# Supplementary material for: Measuring frailty in younger populations: a rapid review of evidence
Source: BMJ Open. 2021 Mar 22;11(3):e047051. doi: 10.1136/bmjopen-2020-047051 (PMC7986767; doi:10.1136/bmjopen-2020-047051)
Supplement: Supplementary data [file bmjopen-2020-047051supp001.pdf]

Supplementary materials

Searches strategy applied to Medline

Searched 17th March 2020; Database(s): **Ovid MEDLINE(R)** 1946 to March Week 1 2020

Key:

/	thesaurus heading
.ti,ab	terms must appear in the title or abstract fields
adj8	the terms either side must be within eight words of each other
*	unlimited truncation
?	single letter truncation

#	Searches	Results
1	Frailty/cl, di, ep [Classification, Diagnosis, Epidemiology]	1007
2	Frailty/	2162
3	"Sensitivity and Specificity"/	343505
4	"Predictive Value of Tests"/	199066
5	ROC Curve/	56249
6	"Diagnostic Techniques and Procedures"/	3243
7	exp Physical Examination/	1349129
8	Symptom Assessment/	4429
9	Psychometrics/	73737
10	Prevalence/	284108
11	or/3-10	2159330
12	2 and 11	631
13	((frail* or prefrail* or pre frail*) adj8 (measur* or assess* or estimat* or define? or definition or classif* or test* or identif* or observ* or screen* or review* or indicat* or criteri* or sign? or symptom? or prevalence*)).ti,ab.	5778
14	((frail* or prefrail* or pre frail*) adj8 (instrument? or tool? or framework* or index* or score? or scale? or psychometric or reliable or reliability or valid* or responsive*)).ti,ab.	2733
15	((frail* or prefrail* or pre frail*) adj8 (associat* or predict*)).ti,ab.	3371
16	or/13-15	7248
17	1 or 12 or 16	7460
18	exp Aged/	3062714
19	adult/ or middle aged/ or young adult/	6433186
20	18 not (18 and 19)	645469
21	17 not 20	2842
22	limit 21 to (english language and humans and yr="2000 -Current")	2511

Supplementary materials table S1. Framework to determine evidence of measure validity (predictive and/or criterion)

Green (evidence of validity)	• Prediction of mortality and/or hospital admissions was statistically significant<br>• Associations and agreement between measures (criterion validity) were judged to be good or strong by study authors
Amber (unclear or inconsistent evidence of validity)	• Evidence of validity was mixed because:<br>o The frailty measure was tested against both outcomes (mortality and hospital admission) but observed a significant association for only one outcome<br>o Prediction was statistically significant for only some sub-groups (e.g. age groups)<br>o Evidence of either, but not both, predictive or criterion validity was demonstrated<br>o Evidence of predictive validity varied depending on when (e.g. 30 day) and where (e.g. hospital or ICU) mortality was measured, the level of frailty tested (e.g. moderate or severe), or how the frailty score was used (e.g. as continuous or binary)<br>o Where individual components of the frailty measure were tested and only some, not all, demonstrated predictive validity<br>• Only differences in mortality rates between frail and not frail were reported, with no test of prediction<br>• Unclear reporting in the paper made it difficult to determine whether the measure was intended to capture frailty or a different concept<br>• Associations and agreement between measures (criterion validity) were judged to be fair or modest by study authors
Red (no evidence of validity)	• Prediction of mortality and/or hospital admissions was not statistically significant<br>• Data were not reported in the publication to verify a claim that the frailty measure predicted mortality and/or hospital admissions<br>• Associations and agreement between measures (criterion validity) were judged to be poor or weak by study authors

Supplementary materials table S2. Number of studies using frailty measures[a]

Measure	Number of studies using measure
Phenotype model (or variation of these criteria)[b]	137
Cumulative deficit model frailty indices[c]	88
CFS	14
Sarcopenia/muscle wasting/muscle size/sarco-osteopenia/morphemic values of muscle (including in combination with weight and mobility)	9
Liver Frailty Index[d]	5
Short Physical Performance Battery	5
Hand grip	3
Walk Test	2
Groningen Frailty Indicator	2
Risk Analysis Index	2
Veterans Aging Cohort Study Index	2
Performance based frailty scale	1
Function Based Frailty scale	1
EQ-5D Item C	1
Comprehensive Frailty Assessment Instrument	1
Tilburg Frailty Indicator	1
Physical Performance Test	1
Blood levels of alanine amino transferase	1
Cervical Endocrine Surgery Risk Index	1
Edmonton Frail Scale	1
Physical Function Scale of SF36	1
Frailty Discriminant Score	1
Frailty Framework among Homeless and Vulnerable Populations	1
Frailty Framework among Vulnerable Populations	1
Morse Fall Risk Score	1
Chair stands	1
International Myeloma Working Group Frailty Score	1
Kidney Disease Quality of Life SF36	1
Frailty Risk Score	1
Composite score from CFS and Montreal Cognitive Assessment	1

Frailty Questionnaire	1
Radiographic frailty markers	1
Vascular disease (CT scan) as indicator of frailty alongside other CT scan indicators of frailty	1
Not reported	1

[a]Numbers are not mutually exclusive as some studies used more than one measure ; [b]Includes the FRAIL scale, the Study of Osteoporotic Fractures Scale, the SHARE Frailty Instrument for Primary care, and the John Hopkins Frailty Indicator, all of which use items that overlap with the phenotype model; [c]Includes frailty indices where the number of index deficits varied between studies, the modified Frailty Indices (mFI 5 and 11 item versions), the European Male Ageing Study Frailty Index, the Spinal Tumor Frailty Index, the Adult Spinal Deformity Frailty Index, and the Systemic Lupus International Collaborating Clinics Frailty Index; [d]Although termed a frailty index, the Liver Frailty Index is not based on the cumulative deficit model.

Supplementary materials table S3. Summary of evidence of predictive validity for frailty measures in younger populations

Paper	Sample size	Sample age Mean/median (SD/IQR)	Sample population	Type of validity PREDICTIVE (MORTALITY AND/OR ADMISSION), CRITERION(CORRELATION OR AGREEMENT))
**Phenotype**				
Ness 2013	2333	Mean: 33.6 (8.1)	Adult survivors of childhood cancer	PREDICTIVE (MORTALITY)
Delgado 2015	812	Median: 52.0 (42-61)	Chronic Kidney disease	PREDICTIVE (MORTALITY)
McAdams-DeMarco 2018	605	Mean: 53.7 (13.5)	End-stage renal disease	PREDICTIVE (MORTALITY)
Fitzpatrick 2019	370	Mean: 54.9	End stage renal disease	PREDICTIVE (MORTALITY)
Chu 2019	569	Mean: 51.7 (14.0)	End stage renal disease	PREDICTIVE (MORTALITY)
Bao 2012	1576	Mean: 59.6 (14.2)	End stage renal disease	PREDICTIVE (MORTALITY AND ADMISSIONS)
Makhani 2017	330	Mean: 58.0 (SD not reported, range 18-89)	Generic surgery	PREDICTIVE (MORTALITY)
Johansen 2016	771	Mean: 57.1 (14.2)	Haemodialysis	PREDICTIVE (MORTALITY)
Johansen 2019	727	Mean: 57.2 (14.2)	Haemodialysis	PREDICTIVE (MORTALITY)
Lee 2017	1658	Mean: 55.2 (11.9)	Dialysis	PREDICTIVE (ADMISSIONS)
Jha 2016[a,b]	156	Mean: 53.0 (12.0[a], 13.0[b])	Heart failure	PREDICTIVE (MORTALITY)
Pamukcuoglu et al 2019	117	Median: 59.0, range 40-73	Hemotopoietic cell transplant	PREDICTIVE (MORTALITY)
Akgun 2014	6515	Mean: 47.6 (8.5)	HIV	PREDICTIVE (MORTALITY AND ADMISSIONS)
Kelly 2019	1016	Median: 51.0 (46.5)	HIV	PREDICTIVE (MORTALITY)

Gustaffson 2017	1385	Mean: 42.6 (8.8)	HIV	PREDICTIVE (MORTALITY)
Verheij 2020	598	Median:<br>Robust: 51.5 (47.6-56.9)<br>Prefail: 53.3 (48.6-60.1)<br>Frail: 55.1 (49.7-60.0)	HIV	PREDICTIVE (MORTALITY)
McAdams-DeMarco 2013	383	Mean: 53.5 (13.9)	Kidney transplant	PREDICTIVE (MORTALITY)
McAdams-DeMarco 2015	537	Mean: 53.0 (14.0)	Kidney transplant	PREDICTIVE (MORTALITY)
Montgomery 2020	100	Mean: 59.0 (7.0)	Lung transplant	PREDICTIVE (MORTALITY)
Singer 2018	299	Median: 59.0 (IQR: 50, 65)	Lung Transplant	PREDICTIVE (MORTALITY)
Ravindrarajah 2013	2929	Mean: 59.9 (10.8)	General population	PREDICTIVE (MORTALITY)
Malmstrom 2014	998	Mean: (at baseline according to tertile): 51.4 (1.1); 56.0 (1.5); 61.5, (2.0).	General population	PREDICTIVE (MORTALITY)
Hope 2017	95	Mean: 57.1, (17.5)	Critical illness/ICU	PREDICTIVE (MORTALITY)<br>*But as a composite outcome combining death with disability outcome.*
Singer 2015	395	Median: 59.0 (IQR: 50–64)	Lung Transplant	PREDICTIVE (MORTALITY)
Moayedi 2018	201	Mean: 53.8 (12.4)	Heart failure	PREDICTIVE (MORTALITY)
McAdams-DeMarco 2017	663	Mean: 53.0 (13.9)	Kidney transplant	PREDICTIVE (MORTALITY)
Joseph 2017	75	Mean: 58.0 (12.0)	End-stage heart failure	PREDICTIVE (MORTALITY)
Rozenberg 2018	221	Cohort 1 mean: 59.0 (9.0)<br>Cohort 2 mean: 55 (43-62)	Lung transplant	PREDICTIVE (MORTALITY)
Johansen 2014	771	Not frail: mean: 53.8 (14.4); self-report: mean: 57.6 (12.1); performance: 51.6 (15.8); self report + performance: mean 62.9, (13.0)	Haemodialysis	CRITERION (AGREEMENT BETWEEN SELF-REPORT AND PERFORMANCE BASED PHENOTYPE CRITERIA)
McDonagh 2020<br>*St Vincent's Frailty Measure (Fried criteria)*	131	Mean: 54.0 (14.0)	Heart Failure	CRITERION (CORRELATION BETWEEN THREE VERSIONS OF PHENOTYPE CRITERIA)

Rozenberg 2018	221	Cohort 1 mean: 59.0 (9.0)<br>Cohort 2 mean: 55.0 (SD not reported)	Lung transplant	CRITERION (AGREEMENT WITH TWO ALTERNATIVE VERSIONS OF PHENOTYPE CRITERIA BASED ON CLINICAL DATA AND A DATASET)
Erlandson 2012	359	Median: 50.8 (IQR: 47.7–55.7)	HIV	CRITERION (AGREEMENT WITH THE SHORT PHYSICAL PERFORMANCE BATTERY AND WALK TEST)
**Frailty indices, where the number of index deficits varied between studies (condition specific indices are in italics)**				
Kane 2017	8555	Mean: 40.3 (13.0) (under 65s group)	People with metabolic syndrome	PREDICTIVE (MORTALITY)
Li 2019	42953	Multiple means reported by sex and twin group. Reader referred to paper for further details.	General population	PREDICTIVE (MORTALITY)
Malmstrom 2014	998	Mean: (at baseline according to tertile): 51.4 (1.1); 56.0 (1.5); 61.5 (2.0).	General population	PREDICTIVE (MORTALITY)
McKenzie 2016	3034	Mean: 53.9 (17.3)	Intellectual & developmental disabilities	PREDICTIVE (ADMISSIONS)
Myers 2014	1521	Grouped on frailty scores ranging from mean 52.0 (8.0) to mean 77.0 (6.0)	Myocardial infarction survivors	PREDICTIVE (MORTALITY)
Rockwood 2011	14713	Mean: 44.0 (18.0)	General population	PREDICTIVE (MORTALITY)
Pena 2014	SHARE: 57546;<br>YALE-PEP: 754;<br>NSHS: 3227	NSHS: mean: 48.1 (19.8)<br>SHARE: mean: 64.2 (10.5)<br>Yale-PEP: mean: 78.4 (5.3)	General population	PREDICTIVE (MORTALITY)
Jayanama 2018	9030	Mean: 46.6 (16.9)	General population	PREDICTIVE (MORTALITY)
Hyde 2016	363	Range: 45-96 years, 53.8% aged <60 years	General population	PREDICTIVE (MORTALITY)
Guaraldi 2017	47	Mean: 51.2 (6.9)	HIV + Liver transplant	PREDICTIVE (MORTALITY)
Guaraldi 2015	2720	Mean: 46.0 (8.0)	HIV	PREDICTIVE (MORTALITY)
Brothers 2017	963	Mean: 46.8 (7.1)	HIV	PREDICTIVE (MORTALITY)
Ravindrarajah 2013	2929	Mean: 59.9 (10.8)	General population	PREDICTIVE (MORTALITY)

Legge 2019 *Systemic Lupus International Collaborating Clinics (SLICC)-FI*	1683	Mean: 35.7 (13.3)	Systemic lupus erythematosus (SLE)	PREDICTIVE (MORTALITY)
Blodgett 2017 (Lab and self report FI)	8888	Mean: 49.4 (19.0)	General population	PREDICTIVE (MORTALITY)
Martin 2018	2893	Mean: 49.5 (17.7)	Intellectual & developmental disabilities	PREDICTIVE (MORTALITY)
Ahmed 2017 *Spinal Tumour Frailty Index*	6727	Median: 47, IQR: 28 - 61	Spinal tumour surgery	PREDICTIVE (MORTALITY)
**Frailty indices: mFI 11**				
Adams 2013	6727	Mean: 54.7 (17.2),	Head and neck surgery	PREDICTIVE (MORTALITY)
Akyar 2018	132765	71.2% aged < 60 years	Emergency surgery	PREDICTIVE (MORTALITY)
Konstantinidis 2017	1171	Median: 55.0, range 19-87	Cytoreductive surgery with intraoperative hyperthermic intraperitoneal chemotherapy	PREDICTIVE (MORTALITY)
McChesney 2020	10048	Mean: 59.9 (5.3)	Radial pelvic surgery patients	PREDICTIVE (MORTALITY)
McIntyre 2020	75	Mean: 55.4 (1.5)	Brain haemorrhage - angiogram-negative subarachnoid haemorrhages (ANSAH)	PREDICTIVE (MORTALITY)
Mosquera 2018	962913	Mean: 53.8 (17.0)	Thoracoabdominal surgery patients	PREDICTIVE (MORTALITY)
Shin 2017	6965	ACDF group – mean: 52.9 (SD not reported)<br>PCF group – mean: 59.8 (SD not reported)	Post Cervical Spinal Fusion	PREDICTIVE (MORTALITY)<br>*But as composite outcome combining death with any post-surgical complications.*

Louwers 2017	10300	Mean: 59.0 (SD not reported)	Hepatectomy patients	PREDICTIVE (MORTALITY)<br>*But as a composite outcome combining death with any post-surgical complications.*
Fang 2017	379	Mean: 59.0 (15.0)	Lower extremity amputation	PREDICTIVE (ADMISSIONS)
McIntyre 2019	217	Mean: 57.6 (1.0)	Brain haemorrhage - aneurysmal subarachnoid haemorrhage (aSAH)	PREDICTIVE (MORTALITY)
Banasek 20	634	Mean: 50.3 (19.8)	Traumatic spinal cord injury	PREDICTIVE (MORTALITY)
**Frailty indices: mFI 5**				
Traven 2020	24477	Mean: 58.4 (SD note reported)	Post Arthroscopic Rotator Cuff Repair	PREDICTIVE (MORTALITY AND ADMISSIONS)
Subramaniam 2018	541485	2012 - 56.84 (16.76)<br>2015 - 56.42 (16.85)	Post surgery (general)	PREDICTIVE (MORTALITY AND ADMISSION)<br>CRITERION (CORRELATION WITH MFI 11)
Balla 2019	97905	Mean 55.9 (SD not reported)	Ventral Hernia Repair	CRITERION (CORRELATION WITH MFI 11)
**Short Physical Performance Battery**				
Courtwright 2018	90	Mean: 53.5 (14.1)	Lung transplant	PREDICTIVE (ADMISSIONS)
Lai 2016	309	Median: 59 (IQR 53-63)	End-stage liver disease	PREDICTIVE (MORTALITY)
Nastasi 2018	719	Mean: 51.6 (14.2)	Kidney transplant (recipients)	PREDICTIVE (MORTALITY)
Singer 2018	299	Median: 59 (IQR: 50, 65)	Lung Transplant	PREDICTIVE (MORTALITY)
Singer 2015	395	Median: 59 (IQR: 50–64)	Lung Transplant	PREDICTIVE (MORTALITY)<br>*But as a composite outcome combining death with transplant delisting.*
Erlandson 2012	359	Median: 50.8 (IQR: 47.7–55.7)	HIV	CRITERION (AGREEMENT WITH WALK TEST AND PHENOTYPE CRITERIA)
**Clinical Frailty Scale**				

Fernando 2019	8110	Not frail<br>Mean: 57.6 (18.1)<br>Frail<br>Mean: 69.2 (12.2)	Critical illness/ICU	PREDICTIVE (MORTALITY)
Bagshaw 2016	197	Mean: 58.5 (4.1)	Critical illness/ICU	PREDICTIVE (MORTALITY AND ADMISSIONS)
Hope 2017	95	Mean: 57.1 (17.5)	Critical illness/ICU	PREDICTIVE (MORTALITY)<br>*But as a composite outcome combining death with disability outcome.*
Ney 2018<br>MoCA-CFS	355	Mean: 55.9 (9.6)	Cirrhosis	PREDICTIVE (MORTALITY)
Kahlon 2015	495	67.3% aged under 60 years	General population	PREDICTIVE (MORTALITY AND ADMISSIONS)
Montgomery 2019	15238	Mean: 58.0 (17.0)	Critical illness/ICU	PREDICTIVE (MORTALITY)
Hewitt 2019	2279	Median: 54.0 (IQR: 36–72)	Emergency surgery	PREDICTIVE (MORTALITY AND ADMISSIONS)
**Frail Scale**				
Griffin 2018	2541	Mean 50.2 (SD not reported)	General population	PREDICTIVE (MORTALITY)
Chode 2016	998	With diabetes<br>Mean: 57.4 (4.4)<br>Without diabetes<br>Mean: 55.9 (4.4)	Diabetes	PREDICTIVE (MORTALITY)
Chao 2018	560,795	Mean: 56.4 (13.8)	Diabetes	PREDICTIVE (MORTALITY AND ADMISSIONS)
Susanto 2017	10412	Range 50–66. Mean age not reported but uses same cohort in another publication (same study team) with a mean age of 52.5 years.	General population	PREDICTIVE (MORTALITY)
Malmstrom 2014	998	Range 49-65	General population	PREDICTIVE (MORTALITY)
Ravindrarajah 2013<br>EMAS Frail Scale	2929	Mean: 59.9 (10.8)	General population	PREDICTIVE (MORTALITY)
Morley 2012	1586	Various analytic groups according to ADL difficult/dependency and frailty level, ranging from mean 56.1 (4.5) to 57.7 (3.9)	General population	PREDICTIVE (MORTALITY)

**Liver Frailty Index**				
Lai 2019	1044	Median: 57 (IQR 49-63)	End-stage liver disease	PREDICTIVE (MORTALITY)
Lai 2017	536	Median: 58 (IQR 50-63)	End-stage liver disease	PREDICTIVE (MORTALITY)
Lai 2018	529	Median: 58 (IQR 50-63)	End-stage liver disease	PREDICTIVE (MORTALITY)
Haugen 2019b	1108	Mean: 55.0 (10.0)	Liver transplant	PREDICTIVE (MORTALITY)
**SOF Frailty Scale and CHS Frailty Scale**				
Malmstrom 2014	998	Mean: (at baseline according to tertile): 51.4 (1.1); 56.0 (1.5); 61.5 (2.0).	General population	PREDICTIVE (MORTALITY)
**John Hopkins Frailty Indicator**				
Asemota 2019	115317	Frail<br>Mean: 57.1 (SD: 16.9)<br>Non-frail<br>Mean: 51.9 (15.8)	Transphenoidal Pituary Surgery	PREDICTIVE (MORTALITY)
**Walk test**				
Erlandson 2012	359	Median: 50.8, IQR: 47.7–55.7	HIV	CRITERION (AGREEMENT WITH PHENOTYPE CRITERIA AND THE SHORT PHYSICAL PERFORMANCE BATTERY)
**Muscle mass/sarcopenia**				
Kelm 2016	36	Median 56 (IQR 49,62)	Lung transplant	PREDICTIVE (MORTALITY)
Heberton 2016	333	Sarcopenic (yes): Mean: 53.0 (14.0)<br>Sarcopenic (no): Mean 57.0 (14.0)	Left ventricular assist device (LVAD) implantation	PREDICTIVE (MORTALITY)
**Hand grip**				
Chung 2014	72	Mean 59.0 (2.0)	Heart failure	PREDICTIVE (MORTALITY)
**CERSI**				

Mascarella 2020	154895	Mean: 56.1 (15.6)	Thyroid & parathyroid surgery patients	PREDICTIVE (MORTALITY)<br>*But as a composite outcome combining death with any adverse event.*

Bibliographic details of included studies [1-227228-268]

1. Abdel-Kader K, Girard TD, Brummel NE, et al. Acute Kidney Injury and Subsequent Frailty Status in Survivors of Critical Illness: A Secondary Analysis. *Crit Care Med* 2018; **46**(5): e380-e8.
2. Adams P, Ghanem T, Stachler R, Hall F, Velanovich V, Rubinfeld I. Frailty as a predictor of morbidity and mortality in inpatient head and neck surgery. *JAMA Otolaryngol Head Neck Surg* 2013; **139**(8): 783-9.
3. Ahmed AK, Goodwin CR, De la Garza-Ramos R, et al. Predicting Short-Term Outcome After Surgery for Primary Spinal Tumors Based on Patient Frailty. *World Neurosurgery*; **108**: 393-8.
4. Aizpuru M, Staley C, Reisman W, Gottschalk MB, Schenker ML. Determinants of Length of Stay After Operative Treatment for Femur Fractures. *J Orthop Trauma* 2018; **32**(4): 161-6.
5. Akgun KM, Tate JP, Crothers K, et al. An adapted frailty-related phenotype and the VACS index as predictors of hospitalization and mortality in HIV-infected and uninfected individuals. *J Acquir Immune Defic Syndr* 2014; **67**(4): 397-404.
6. Akgun KM, Tate JP, Oursler KK, et al. Association of chronic obstructive pulmonary disease with frailty measurements in HIV-infected and uninfected Veterans. *AIDS* 2016; **30**(14): 2185-93.
7. Akyar S, Armenia SJ, Ratnani P, Merchant AM. The Impact of Frailty on Postoperative Cardiopulmonary Complications in the Emergency General Surgery Population. *Surg J (N Y)* 2018; **4**(2): e66-e77.
8. Alshaikh JT, Amdur R, Sidawy A, Trachiotis G, Kaminski HJ. Thymectomy is safe for myasthenia gravis patients: Analysis of the NSQIP database. *Muscle Nerve* 2016; **53**(3): 370-4.
9. Althoff KN, Jacobson LP, Cranston RD, et al. Age, comorbidities, and AIDS predict a frailty phenotype in men who have sex with men. *J Gerontol A Biol Sci Med Sci* 2014; **69**(2): 189-98.
10. Anderson JE, Inui T, Talamini MA, Chang DC. Cholecystostomy offers no survival benefit in patients with acute acalculous cholecystitis and severe sepsis and shock. *J Surg Res* 2014; **190**(2): 517-21.
11. Andrews JS, Trupin L, Wysham KD, Hough CL, Yelin EH, Katz PP. The Impact of Frailty on Changes in Physical Function and Disease Activity Among Adults With Rheumatoid Arthritis. *ACR Open Rheumatol* 2019; **1**(6): 366-72.
12. Andrews JS, Trupin L, Yelin EH, Hough CL, Covinsky KE, Katz PP. Frailty and reduced physical function go hand in hand in adults with rheumatoid arthritis: a US observational cohort study. *Clin Rheumatol* 2017; **36**(5): 1031-9.
13. Asemota AO, Gallia GL. Impact of frailty on short-term outcomes in patients undergoing transsphenoidal pituitary surgery. *J Neurosurg* 2019; **132**(2): 360-70.
14. Ayesta A, Astiz MTV, Masa MJV, Segovia J, Cosio MDG, Martinez-Selles M. Rationale and design of the FELICITAR registry (Frailty Evaluation After List Inclusion, Characteristics and Influence on Transplantation and Results). *Clin Cardiol* 2018; **41**(3): 293-9.

15. Bagshaw M, Majumdar SR, Rolfson DB, Ibrahim Q, McDermid RC, Stelfox HT. A prospective multicenter cohort study of frailty in younger critically ill patients. *Crit Care* 2016; **20**(1): 175.
16. Balla FM, Yheulon CG, Stetler JL, Patel AD, Lin E, Davis SS. Ventral hernia repair outcomes predicted by a 5-item modified frailty index using NSQIP variables. *Hernia* 2019; **23**(5): 891-8.
17. Banaszek D, Inglis T, Marion TE, et al. Effect of Frailty on Outcome after Traumatic Spinal Cord Injury. *J Neurotrauma* 2020; **37**(6): 839-45.
18. Bao Y, Dalrymple L, Chertow GM, Kaysen GA, Johansen KL. Frailty, dialysis initiation, and mortality in end-stage renal disease. *Arch Intern Med* 2012; **172**(14): 1071-7.
19. Bello GA, Lucchini RG, Teitelbaum SL, Shapiro M, Crane MA, Todd AC. Development of a Physiological Frailty Index for the World Trade Center General Responder Cohort. *Curr Gerontol Geriatr Res* 2018; **2018**: 3725926.
20. Bindawas SM, Vennu V, Stubbs B. Longitudinal Relationship Between Knee Pain Status and Incident Frailty: Data from the Osteoarthritis Initiative. *Pain Med* 2018; **19**(11): 2146-53.
21. Blanco JR, Barrio I, Ramalle-Gomara E, et al. Gender differences for frailty in HIV-infected patients on stable antiretroviral therapy and with an undetectable viral load. *PLoS One* 2019; **14**(5): e0215764.
22. Blodgett JM, Theou O, Howlett SE, Rockwood K. A frailty index from common clinical and laboratory tests predicts increased risk of death across the life course. *Geroscience* 2017; **39**(4): 447-55.
23. Blodgett JM, Theou O, Mitnitski A, Howlett SE, Rockwood K. Associations between a laboratory frailty index and adverse health outcomes across age and sex. *Aging Med (Milton)* 2019; **2**(1): 11-7.
24. Bouillon K, Batty GD, Hamer M, et al. Cardiovascular disease risk scores in identifying future frailty: the Whitehall II prospective cohort study. *Heart* 2013; **99**(10): 737-42.
25. Bouillon K, Kivimaki M, Hamer M, et al. Diabetes risk factors, diabetes risk algorithms, and the prediction of future frailty: the Whitehall II prospective cohort study. *J Am Med Dir Assoc* 2013; **14**(11): 851 e1-6.
26. Bregigeon S, Galinier A, Zaegel-Faucher O, et al. Frailty in HIV infected people: a new risk factor for bone mineral density loss. *AIDS* 2017; **31**(11): 1573-7.
27. Brothers TD, Kirkland S, Theou O, et al. Predictors of transitions in frailty severity and mortality among people aging with HIV. *PLoS One* 2017; **12**(10): e0185352.
28. Buganza-Torio E, Mitchell N, Abraldes JG, et al. Depression in cirrhosis - a prospective evaluation of the prevalence, predictors and development of a screening nomogram. *Aliment Pharmacol Ther* 2019; **49**(2): 194-201.
29. Buitrago DH, Gangadharan SP, Majid A, et al. Frailty Characteristics Predict Respiratory Failure in Patients Undergoing Tracheobronchoplasty. *Ann Thorac Surg* 2018; **106**(3): 836-41.
30. Chao CT, Wang J, Chien KL, group COoGNiNs. Both pre-frailty and frailty increase healthcare utilization and adverse health outcomes in patients with type 2 diabetes mellitus. *Cardiovasc Diabetol* 2018; **17**(1): 130.
31. Chen SY, Stem M, Cerullo M, et al. The Effect of Frailty Index on Early Outcomes after Combined Colorectal and Liver Resections. *J Gastrointest Surg* 2018; **22**(4): 640-9.

32. Chiang JM, Kaysen GA, Segal M, Chertow GM, Delgado C, Johansen KL. Low testosterone is associated with frailty, muscle wasting and physical dysfunction among men receiving hemodialysis: a longitudinal analysis. *Nephrol Dial Transplant* 2019; **34**(5): 802-10.
33. Chode S, Malmstrom TK, Miller DK, Morley JE. Frailty, Diabetes, and Mortality in Middle-Aged African Americans. *J Nutr Health Aging* 2016; **20**(8): 854-9.
34. Chu NM, Deng A, Ying H, et al. Dynamic Frailty Before Kidney Transplantation: Time of Measurement Matters. *Transplantation* 2019; **103**(8): 1700-4.
35. Chu NM, Gross AL, Shaffer AA, et al. Frailty and Changes in Cognitive Function after Kidney Transplantation. *J Am Soc Nephrol* 2019; **30**(2): 336-45.
36. Chung CJ, Wu C, Jones M, et al. Reduced handgrip strength as a marker of frailty predicts clinical outcomes in patients with heart failure undergoing ventricular assist device placement. *J Card Fail* 2014; **20**(5): 310-5.
37. Cook MJ, Oldroyd A, Pye SR, et al. Frailty and bone health in European men. *Age Ageing* 2017; **46**(4): 635-41.
38. Courtwright AM, Zaleski D, Gardo L, et al. Causes, Preventability, and Cost of Unplanned Rehospitalizations Within 30 Days of Discharge After Lung Transplantation. *Transplantation* 2018; **102**(5): 838-44.
39. Courtwright AM, Zaleski D, Tevald M, et al. Discharge frailty following lung transplantation. *Clin Transplant* 2019; **33**(10): e13694.
40. Cron DC, Friedman JF, Winder GS, et al. Depression and Frailty in Patients With End-Stage Liver Disease Referred for Transplant Evaluation. *Am J Transplant* 2016; **16**(6): 1805-11.
41. de Haas SCM, de Jonge EAL, Voortman T, et al. Dietary patterns and changes in frailty status: the Rotterdam study. *Eur J Nutr* 2018; **57**(7): 2365-75.
42. Delgado C, Doyle JW, Johansen KL. Association of frailty with body composition among patients on hemodialysis. *J Ren Nutr* 2013; **23**(5): 356-62.
43. Delgado C, Grimes BA, Glidden DV, Shlipak M, Sarnak MJ, Johansen KL. Association of Frailty based on self-reported physical function with directly measured kidney function and mortality. *BMC Nephrol* 2015; **16**: 203.
44. Denfeld QE, Winters-Stone K, Mudd JO, Hiatt SO, Chien CV, Lee CS. Frequency of and Significance of Physical Frailty in Patients With Heart Failure. *Am J Cardiol* 2017; **119**(8): 1243-9.
45. Derck JE, Thelen AE, Cron DC, et al. Quality of life in liver transplant candidates: frailty is a better indicator than severity of liver disease. *Transplantation* 2015; **99**(2): 340-4.
46. Dolgin NH, Smith AJ, Harrington SG, Movahedi B, Martins PNA, Bozorgzadeh A. Association Between Sarcopenia and Functional Status in Liver Transplant Patients. *Exp Clin Transplant* 2019; **17**(5): 653-64.
47. Dos Santos Mantovani M, Coelho de Carvalho N, Archangelo TE, et al. Frailty predicts surgical complications after kidney transplantation. A propensity score matched study. *PLoS One* 2020; **15**(2): e0229531.
48. Dumond JB, Francis O, Cottrell M, et al. Tenofovir/emtricitabine metabolites and endogenous nucleotide exposures are associated with p16(INK4a) expression in subjects on combination therapy. *Antivir Ther* 2016; **21**(5): 441-5.
49. Erlandson KM, Allshouse AA, Jankowski CM, et al. Comparison of functional status instruments in HIV-infected adults on effective antiretroviral therapy. *HIV Clin Trials* 2012; **13**(6): 324-34.
50. Erlandson KM, Bradford Y, Samuels DC, et al. Mitochondrial DNA Haplogroups and Frailty in Adults Living with HIV. *AIDS Res Hum Retroviruses* 2020; **36**(3): 214-9.

51. Erlandson KM, Ng DK, Jacobson LP, et al. Inflammation, Immune Activation, Immunosenescence, and Hormonal Biomarkers in the Frailty-Related Phenotype of Men With or at Risk for HIV Infection. *J Infect Dis* 2017; **215**(2): 228-37.
52. Erlandson KM, Perez J, Abdo M, et al. Frailty, Neurocognitive Impairment, or Both in Predicting Poor Health Outcomes Among Adults Living With Human Immunodeficiency Virus. *Clin Infect Dis* 2019; **68**(1): 131-8.
53. Erlandson KM, Wu K, Koletar SL, et al. Association Between Frailty and Components of the Frailty Phenotype With Modifiable Risk Factors and Antiretroviral Therapy. *J Infect Dis* 2017; **215**(6): 933-7.
54. Escota GV, Patel P, Brooks JT, et al. Short communication: The Veterans Aging Cohort Study Index is an effective tool to assess baseline frailty status in a contemporary cohort of HIV-infected persons. *AIDS Res Hum Retroviruses* 2015; **31**(3): 313-7.
55. Fang ZB, Hu FY, Arya S, Gillespie TW, Rajani RR. Preoperative frailty is predictive of complications after major lower extremity amputation. *J Vasc Surg* 2017; **65**(3): 804-11.
56. Fernando SM, McIsaac DI, Rochwerg B, et al. Frailty and invasive mechanical ventilation: association with outcomes, extubation failure, and tracheostomy. *Intensive Care Med* 2019; **45**(12): 1742-52.
57. Fitzpatrick J, Sozio SM, Jaar BG, et al. Frailty, body composition and the risk of mortality in incident hemodialysis patients: the Predictors of Arrhythmic and Cardiovascular Risk in End Stage Renal Disease study. *Nephrol Dial Transplant* 2019; **34**(2): 346-54.
58. Franconi I, Theou O, Wallace L, et al. Construct validation of a Frailty Index, an HIV Index and a Protective Index from a clinical HIV database. *PLoS One* 2018; **13**(10): e0201394.
59. Garonzik-Wang JM, Govindan P, Grinnan JW, et al. Frailty and delayed graft function in kidney transplant recipients. *Arch Surg* 2012; **147**(2): 190-3.
60. George EM, Burke WM, Hou JY, et al. Measurement and validation of frailty as a predictor of outcomes in women undergoing major gynaecological surgery. *BJOG* 2016; **123**(3): 455-61.
61. Gordon SJ, Baker N, Kidd M, Maeder A, Grimmer KA. Pre-frailty factors in community-dwelling 40-75 year olds: opportunities for successful ageing. *BMC Geriatr* 2020; **20**(1): 96.
62. Griffin FR, Mode NA, Ejiogu N, Zonderman AB, Evans MK. Frailty in a racially and socioeconomically diverse sample of middle-aged Americans in Baltimore. *PLoS One* 2018; **13**(4): e0195637.
63. Groß M, Herr A, Hower M, et al. Unemployment, health, and education of HIV-infected males in Germany. *International Journal of Public Health* 2016; **61**(5): 593-602.
64. Guaraldi G, Brothers TD, Zona S, et al. A frailty index predicts survival and incident multimorbidity independent of markers of HIV disease severity. *AIDS* 2015; **29**(13): 1633-41.
65. Guaraldi G, De Francesco D, Milic J, et al. The Interplay Between Age and Frailty in People Living With HIV: Results From an 11-Year Follow-up Observational Study. *Open Forum Infect Dis* 2019; **6**(5): ofz199.
66. Guaraldi G, Dolci G, Zona S, et al. A frailty index predicts post-liver transplant morbidity and mortality in HIV-positive patients. *AIDS Res Ther* 2017; **14**(1): 37.
67. Guaraldi G, Francesco D, Malagoli A, et al. Compression of frailty in adults living with HIV. *BMC Geriatr* 2019; **19**(1): 229.

68. Guaraldi G, Franconi I, Milic J, et al. Thymus Imaging Detection and Size Is Inversely Associated With Metabolic Syndrome and Frailty in People With HIV. *Open Forum Infect Dis* 2019; **6**(10): ofz435.
69. Guaraldi G, Malagoli A, Theou O, et al. Correlates of frailty phenotype and frailty index and their associations with clinical outcomes. *HIV Med* 2017; **18**(10): 764-71.
70. Guaraldi G, Zona S, Silva AR, et al. The dynamic association between Frailty, CD4 and CD4/CD8 ratio in people aging with HIV. *PLoS One* 2019; **14**(2): e0212283.
71. Gustafson DR, Shi Q, Holman S, et al. Predicting death over 8 years in a prospective cohort of HIV-infected women: the Women's Interagency HIV Study. *BMJ Open* 2017; **7**(6): e013993.
72. Gustafson DR, Shi Q, Thurn M, et al. Frailty and Constellations of Factors in Aging HIV-infected and Uninfected Women--The Women's Interagency HIV Study. *J Frailty Aging* 2016; **5**(1): 43-8.
73. Hadenfeldt CJ, Darabaris M, Aufdenkamp M. Frailty Assessment in Patients Utilizing a Free Clinic. *J Health Care Poor Underserved* 2017; **28**(4): 1423-35.
74. Haider S, Grabovac I, Berner C, et al. Frailty in seropositive rheumatoid arthritis patients of working age: a cross-sectional study. *Clin Exp Rheumatol* 2019; **37**(4): 585-92.
75. Hampton JP, Owodunni OP, Bettick D, et al. Compliance to an enhanced recovery pathway among patients with a high frailty index after major gastrointestinal surgery results in improved 30-day outcomes. *Surgery* 2019; **166**(1): 75-81.
76. Harland TA, Wang M, Gunaydin D, et al. Frailty as a Predictor of Neurosurgical Outcomes in Brain Tumor Patients. *World Neurosurg* 2020; **133**: e813-e8.
77. Haugen CE, Chu NM, Ying H, et al. Frailty and Access to Kidney Transplantation. *Clin J Am Soc Nephrol* 2019; **14**(4): 576-82.
78. Haugen CE, McAdams-DeMarco M, Verna EC, et al. Association Between Liver Transplant Wait-list Mortality and Frailty Based on Body Mass Index. *JAMA Surg* 2019; **154**(12): 1103-9.
79. Haugen CE, Mountford A, Warsame F, et al. Incidence, Risk Factors, and Sequelae of Post-kidney Transplant Delirium. *J Am Soc Nephrol* 2018; **29**(6): 1752-9.
80. Haugen CE, Thomas AG, Chu NM, et al. Prevalence of frailty among kidney transplant candidates and recipients in the United States: Estimates from a National Registry and Multicenter Cohort Study. *Am J Transplant* 2020; **20**(4): 1170-80.
81. Hayek S, Gibson TM, Leisenring WM, et al. Prevalence and Predictors of Frailty in Childhood Cancer Survivors and Siblings: A Report From the Childhood Cancer Survivor Study. *J Clin Oncol* 2020; **38**(3): 232-47.
82. Heberton GA, Nassif M, Bierhals A, et al. Usefulness of Psoas Muscle Area Determined by Computed Tomography to Predict Mortality or Prolonged Length of Hospital Stay in Patients Undergoing Left Ventricular Assist Device Implantation. *Am J Cardiol* 2016; **118**(9): 1363-7.
83. Hewitt J, Carter B, McCarthy K, et al. Frailty predicts mortality in all emergency surgical admissions regardless of age. An observational study. *Age Ageing* 2019; **48**(3): 388-94.
84. Hope AA, Hsieh SJ, Petti A, Hurtado-Sbordoni M, Verghese J, Gong MN. Assessing the Usefulness and Validity of Frailty Markers in Critically Ill Adults. *Ann Am Thorac Soc* 2017; **14**(6): 952-9.

85. Huang SM, Tseng LM, Chien LY, et al. Effects of non-sporting and sporting qigong on frailty and quality of life among breast cancer patients receiving chemotherapy. *Eur J Oncol Nurs* 2016; **21**: 257-65.
86. Hyde Z, Flicker L, Smith K, et al. Prevalence and incidence of frailty in Aboriginal Australians, and associations with mortality and disability. *Maturitas* 2016; **87**: 89-94.
87. Ianas V, Berg E, Mohler MJ, Wendel C, Klotz SA. Antiretroviral therapy protects against frailty in HIV-1 infection. *J Int Assoc Provid AIDS Care* 2013; **12**(1): 62-6.
88. Jayanama K, Theou O, Blodgett JM, Cahill L, Rockwood K. Frailty, nutrition-related parameters, and mortality across the adult age spectrum. *BMC Med* 2018; **16**(1): 188.
89. Jha SR, Hannu MK, Chang S, et al. The Prevalence and Prognostic Significance of Frailty in Patients With Advanced Heart Failure Referred for Heart Transplantation. *Transplantation* 2016; **100**(2): 429-36.
90. Jha SR, Hannu MK, Gore K, et al. Cognitive impairment improves the predictive validity of physical frailty for mortality in patients with advanced heart failure referred for heart transplantation. *J Heart Lung Transplant* 2016; **35**(9): 1092-100.
91. Jha SR, Hannu MK, Newton PJ, et al. Reversibility of Frailty After Bridge-to-Transplant Ventricular Assist Device Implantation or Heart Transplantation. *Transplant Direct* 2017; **3**(7): e167.
92. Johansen KL, Dalrymple LS, Delgado C, et al. Factors Associated with Frailty and Its Trajectory among Patients on Hemodialysis. *Clin J Am Soc Nephrol* 2017; **12**(7): 1100-8.
93. Johansen KL, Dalrymple LS, Delgado C, et al. Association between body composition and frailty among prevalent hemodialysis patients: a US Renal Data System special study. *J Am Soc Nephrol* 2014; **25**(2): 381-9.
94. Johansen KL, Dalrymple LS, Delgado C, et al. Comparison of self-report-based and physical performance-based frailty definitions among patients receiving maintenance hemodialysis. *Am J Kidney Dis* 2014; **64**(4): 600-7.
95. Johansen KL, Dalrymple LS, Glidden D, et al. Association of Performance-Based and Self-Reported Function-Based Definitions of Frailty with Mortality among Patients Receiving Hemodialysis. *Clin J Am Soc Nephrol* 2016; **11**(4): 626-32.
96. Johansen KL, Delgado C, Kaysen GA, et al. Frailty Among Patients Receiving Hemodialysis: Evolution of Components and Associations With Mortality. *J Gerontol A Biol Sci Med Sci* 2019; **74**(3): 380-6.
97. Johansen KL, Painter P, Delgado C, Doyle J. Characterization of physical activity and sitting time among patients on hemodialysis using a new physical activity instrument. *J Ren Nutr* 2015; **25**(1): 25-30.
98. Joseph SM, Manghelli JL, Vader JM, et al. Prospective Assessment of Frailty Using the Fried Criteria in Patients Undergoing Left Ventricular Assist Device Therapy. *Am J Cardiol* 2017; **120**(8): 1349-54.
99. Kahlon S, Pederson J, Majumdar SR, et al. Association between frailty and 30-day outcomes after discharge from hospital. *CMAJ* 2015; **187**(11): 799-804.
100. Kallianpur KJ, Sakoda M, Gangcuangco LM, et al. Frailty Characteristics in Chronic HIV Patients are Markers of White Matter Atrophy Independently of Age and Depressive Symptoms: A Pilot Study. *Open Med J* 2016; **3**: 138-52.

101. Kane AE, Gregson E, Theou O, Rockwood K, Howlett SE. The association between frailty, the metabolic syndrome, and mortality over the lifespan. *Geroscience* 2017; **39**(2): 221-9.
102. Kang SH, Do JY, Jeong HY, Lee SY, Kim JC. The Clinical Significance of Physical Activity in Maintenance Dialysis Patients. *Kidney Blood Press Res* 2017; **42**(3): 575-86.
103. Kang SH, Do JY, Lee SY, Kim JC. Effect of dialysis modality on frailty phenotype, disability, and health-related quality of life in maintenance dialysis patients. *PLoS One* 2017; **12**(5): e0176814.
104. Kanters DM, Griffith LE, Hogan DB, Richardson J, Patterson C, Raina P. Assessing the measurement properties of a Frailty Index across the age spectrum in the Canadian Longitudinal Study on Aging. *J Epidemiol Community Health* 2017; **71**(8): 794-9.
105. Kehler DS, Ferguson T, Stammers AN, et al. Prevalence of frailty in Canadians 18-79 years old in the Canadian Health Measures Survey. *BMC Geriatr* 2017; **17**(1): 28.
106. Keller DS, Bankwitz B, Nobel T, Delaney CP. Using frailty to predict who will fail early discharge after laparoscopic colorectal surgery with an established recovery pathway. *Dis Colon Rectum* 2014; **57**(3): 337-42.
107. Kelly SG, Wu K, Tassiopoulos K, Erlandson KM, Koletar SL, Palella FJ. Frailty Is an Independent Risk Factor for Mortality, Cardiovascular Disease, Bone Disease, and Diabetes Among Aging Adults With Human Immunodeficiency Virus. *Clin Infect Dis* 2019; **69**(8): 1370-6.
108. Kelm DJ, Bonnes SL, Jensen MD, et al. Pre-transplant wasting (as measured by muscle index) is a novel prognostic indicator in lung transplantation. *Clin Transplant* 2016; **30**(3): 247-55.
109. Konel JM, Warsame F, Ying H, et al. Depressive symptoms, frailty, and adverse outcomes among kidney transplant recipients. *Clin Transplant* 2018; **32**(10): e13391.
110. Konstantinidis IT, Chouliaras K, Levine EA, Lee B, Votanopoulos KI. Frailty Correlates with Postoperative Mortality and Major Morbidity After Cytoreductive Surgery with Hyperthermic Intraperitoneal Chemotherapy. *Ann Surg Oncol* 2017; **24**(13): 3825-30.
111. Kooij KW, Wit FW, Schouten J, et al. HIV infection is independently associated with frailty in middle-aged HIV type 1-infected individuals compared with similar but uninfected controls. *AIDS* 2016; **30**(2): 241-50.
112. Korada SKC, Zhao D, Tibuakuu M, et al. Frailty and subclinical coronary atherosclerosis: The Multicenter AIDS Cohort Study (MACS). *Atherosclerosis* 2017; **266**: 240-7.
113. Kulkarni SS, Chen H, Josbeno DA, et al. Gait Speed and Grip Strength Are Associated With Dropping Out of the Liver Transplant Waiting List. *Transplant Proc* 2019; **51**(3): 794-7.
114. Kull M, Kallikorm R, Lember M. Impact of a new sarco-osteopenia definition on health-related quality of life in a population-based cohort in Northern Europe. *J Clin Densitom* 2012; **15**(1): 32-8.
115. Kutner NG, Zhang R, Allman RM, Bowling CB. Correlates of ADL difficulty in a large hemodialysis cohort. *Hemodial Int* 2014; **18**(1): 70-7.
116. Kutner NG, Zhang R, Huang Y, McClellan WM, Soltow QA, Lea J. Risk factors for frailty in a large prevalent cohort of hemodialysis patients. *Am J Med Sci* 2014; **348**(4): 277-82.
117. Kutner NG, Zhang R, Huang Y, Wasse H. Falls among hemodialysis patients: potential opportunities for prevention? *Clin Kidney J* 2014; **7**(3): 257-63.

118. Lai JC, Covinsky KE, Dodge JL, et al. Development of a novel frailty index to predict mortality in patients with end-stage liver disease. *Hepatology* 2017; **66**(2): 564-74.
119. Lai JC, Covinsky KE, McCulloch CE, Feng S. The Liver Frailty Index Improves Mortality Prediction of the Subjective Clinician Assessment in Patients With Cirrhosis. *Am J Gastroenterol* 2018; **113**(2): 235-42.
120. Lai JC, Dodge JL, Sen S, Covinsky K, Feng S. Functional decline in patients with cirrhosis awaiting liver transplantation: Results from the functional assessment in liver transplantation (FrAILT) study. *Hepatology* 2016; **63**(2): 574-80.
121. Lai JC, Rahimi RS, Verna EC, et al. Frailty Associated With Waitlist Mortality Independent of Ascites and Hepatic Encephalopathy in a Multicenter Study. *Gastroenterology* 2019; **156**(6): 1675-82.
122. Lauerman MH, Raithel M, Kufera J, et al. Comparison of individual and composite radiographic markers of frailty in trauma. *Injury* 2019; **50**(1): 149-55.
123. Layton AM, Armstrong HF, Baldwin MR, et al. Frailty and maximal exercise capacity in adult lung transplant candidates. *Respir Med* 2017; **131**: 70-6.
124. Lee SY, Yang DH, Hwang E, et al. The Prevalence, Association, and Clinical Outcomes of Frailty in Maintenance Dialysis Patients. *J Ren Nutr* 2017; **27**(2): 106-12.
125. Legge A, Kirkl, S., et al. Prediction of Damage Accrual in Systemic Lupus Erythematosus Using the Systemic Lupus International Collaborating Clinics Frailty Index. *Arthritis & Rheumatology* 2019.
126. Legge A, Kirkland S, Rockwood K, et al. Evaluating the Properties of a Frailty Index and Its Association With Mortality Risk Among Patients With Systemic Lupus Erythematosus. *Arthritis Rheumatol* 2019; **71**(8): 1297-307.
127. Legge A, Kirkland S, Rockwood K, et al. Construction of a Frailty Index as a Novel Health Measure in Systemic Lupus Erythematosus. *J Rheumatol* 2020; **47**(1): 72-81.
128. Leopold-George NTN, Nethathe GD. Frailty in perioperative patients in three South African academic hospitals. *S Afr Med J* 2019; **109**(7): 535-40.
129. Li G, Chen M, Li X, et al. Frailty and risk of osteoporotic fractures in patients with rheumatoid arthritis: Data from the Ontario Best Practices Research Initiative. *Bone* 2019; **127**: 129-34.
130. Li X, Ploner A, Karlsson IK, et al. The frailty index is a predictor of cause-specific mortality independent of familial effects from midlife onwards: a large cohort study. *BMC Med* 2019; **17**(1): 94.
131. Limberg J, Ullmann TM, Gray KD, et al. Laparoscopic Adrenalectomy Has the Same Operative Risk as Routine Laparoscopic Cholecystectomy. *J Surg Res* 2019; **241**: 228-34.
132. Limpawattana P, Wirasorn K, Sookprasert A, et al. Frailty Syndrome in Biliary Tract Cancer Patients: Prevalence and Associated Factors. *Asian Pac J Cancer Prev* 2019; **20**(5): 1497-501.
133. Lisiecki J, Zhang P, Wang L, et al. Morphomic measurement of the temporalis muscle and zygomatic bone as novel predictors of hospital-based clinical outcomes in patients with mandible fracture. *J Craniofac Surg* 2013; **24**(5): 1577-81.
134. Louwers L, Schnickel G, Rubinfeld I. Use of a simplified frailty index to predict Clavien 4 complications and mortality after hepatectomy: analysis of the National Surgical Quality Improvement Project database. *American Journal of Surgery* 2016; **211**(6): 1071-6.

135. Luckett P, Paul RH, Navid J, et al. Deep Learning Analysis of Cerebral Blood Flow to Identify Cognitive Impairment and Frailty in Persons Living With HIV. *J Acquir Immune Defic Syndr* 2019; **82**(5): 496-502.
136. Lurie I, Myers V, Goldbourt U, Gerber Y. Perceived social support following myocardial infarction and long-term development of frailty. *Eur J Prev Cardiol* 2015; **22**(10): 1346-53.
137. Lutski M, Tanne D, Goldbourt U. Tall stature in coronary heart disease patients is associated with decreased risk of frailty in late life. *Geriatr Gerontol Int* 2017; **17**(9): 1270-7.
138. Maffei VJ, Ferguson TF, Brashear MM, et al. Lifetime alcohol use among persons living with HIV is associated with frailty. *AIDS* 2020; **34**(2): 245-54.
139. Makhani SS, Kim FY, Liu Y, et al. Cognitive Impairment and Overall Survival in Frail Surgical Patients. *J Am Coll Surg* 2017; **225**(5): 590-600 e1.
140. Malmstrom TK, Miller DK, Herning MM, Morley JE. Low appendicular skeletal muscle mass (ASM) with limited mobility and poor health outcomes in middle-aged African Americans. *J Cachexia Sarcopenia Muscle* 2013; **4**(3): 179-86.
141. Malmstrom TK, Miller DK, Morley JE. A comparison of four frailty models. *J Am Geriatr Soc* 2014; **62**(4): 721-6.
142. Margolick JB, Bream JH, Martinez-Maza O, et al. Frailty and Circulating Markers of Inflammation in HIV+ and HIV- Men in the Multicenter AIDS Cohort Study. *J Acquir Immune Defic Syndr* 2017; **74**(4): 407-17.
143. Martin L, McKenzie K, Ouellette-Kuntz H. Once frail, always frail? Frailty transitions in home care users with intellectual and developmental disabilities. *Geriatr Gerontol Int* 2018; **18**(4): 547-53.
144. Mascarella MA, Milad D, Richardson K, et al. Preoperative Risk Index Among Patients Undergoing Thyroid or Parathyroid Surgery. *Jama Otolaryngol* 2020; **146**(1): 7-12.
145. McAdams-DeMarco MA, Isaacs K, Darko L, et al. Changes in Frailty After Kidney Transplantation. *J Am Geriatr Soc* 2015; **63**(10): 2152-7.
146. McAdams-DeMarco MA, Law A, King E, et al. Frailty and mortality in kidney transplant recipients. *Am J Transplant* 2015; **15**(1): 149-54.
147. McAdams-DeMarco MA, Law A, Salter ML, et al. Frailty and early hospital readmission after kidney transplantation. *Am J Transplant* 2013; **13**(8): 2091-5.
148. McAdams-DeMarco MA, Law A, Tan J, et al. Frailty, mycophenolate reduction, and graft loss in kidney transplant recipients. *Transplantation* 2015; **99**(4): 805-10.
149. McAdams-DeMarco MA, Tan J, Salter ML, et al. Frailty and Cognitive Function in Incident Hemodialysis Patients. *Clin J Am Soc Nephrol* 2015; **10**(12): 2181-9.
150. McAdams-DeMarco MA, Ying H, Olorundare I, et al. Frailty and Health-Related Quality of Life in End Stage Renal Disease Patients of All Ages. *J Frailty Aging* 2016; **5**(3): 174-9.
151. McAdams-DeMarco MA, Ying H, Olorundare I, et al. Individual Frailty Components and Mortality in Kidney Transplant Recipients. *Transplantation* 2017; **101**(9): 2126-32.
152. McAdams-DeMarco MA, Ying H, Thomas AG, et al. Frailty, Inflammatory Markers, and Waitlist Mortality Among Patients With End-stage Renal Disease in a Prospective Cohort Study. *Transplantation* 2018; **102**(10): 1740-6.
153. McAlister FA, Youngson E, Eurich DT. Treated glycosylated hemoglobin levels in individuals with diabetes mellitus vary little by health status: A retrospective cohort study. *Medicine (Baltimore)* 2016; **95**(24): e3894.

154. McChesney SL, Canter DJ, Monlezun DJ, Green H, Margolin DA. Modified Frailty Index Predicts Postoperative Outcomes in Patients Undergoing Radical Pelvic Surgery. *Am Surg* 2020; **86**(2): 95-103.
155. McDonagh J, Salamonson Y, Ferguson C, et al. Evaluating the convergent and discriminant validity of three versions of the frailty phenotype in heart failure: results from the FRAME-HF study. *Eur J Cardiovasc Nurs* 2020; **19**(1): 55-63.
156. McIntyre M, Gandhi C, Dragonette J, et al. Increasing Frailty Predicts Worse Outcomes and Increased Complications After Angiogram-Negative Subarachnoid Hemorrhages. *World Neurosurg* 2020; **134**: e181-e8.
157. McIntyre MK, Gandhi C, Long A, et al. Age predicts outcomes better than frailty following aneurysmal subarachnoid hemorrhage: A retrospective cohort analysis. *Clin Neurol Neurosurg* 2019; **187**: 105558.
158. McKenzie K, Ouellette-Kuntz H, Martin L. Using an accumulation of deficits approach to measure frailty in a population of home care users with intellectual and developmental disabilities: an analytical descriptive study. *BMC Geriatr* 2015; **15**: 170.
159. McKenzie K, Ouellette-Kuntz H, Martin L. Frailty as a Predictor of Institutionalization Among Adults With Intellectual and Developmental Disabilities. *Intellect Dev Disabil* 2016; **54**(2): 123-35.
160. McNelly AS, Rawal J, Shrikrishna D, et al. An Exploratory Study of Long-Term Outcome Measures in Critical Illness Survivors: Construct Validity of Physical Activity, Frailty, and Health-Related Quality of Life Measures. *Crit Care Med* 2016; **44**(6): e362-9.
161. Mezuk B, Lohman M, Dumenci L, Lapane KL. Are depression and frailty overlapping syndromes in mid- and late-life? A latent variable analysis. *Am J Geriatr Psychiatry* 2013; **21**(6): 560-9.
162. Miller BS, Ignatoski KM, Daignault S, et al. A quantitative tool to assess degree of sarcopenia objectively in patients with hypercortisolism. *Surgery* 2011; **150**(6): 1178-85.
163. Miller EK, Lenke LG, Neuman BJ, et al. External Validation of the Adult Spinal Deformity (ASD) Frailty Index (ASD-FI) in the Scoli-RISK-1 Patient Database. *Spine (Phila Pa 1976)* 2018; **43**(20): 1426-31.
164. Miller EK, Vila-Casademunt A, Neuman BJ, et al. External validation of the adult spinal deformity (ASD) frailty index (ASD-FI). *Eur Spine J* 2018; **27**(9): 2331-8.
165. Moayedi Y, Duero Posada JG, Foroutan F, et al. The prognostic significance of frailty compared to peak oxygen consumption and B-type natriuretic peptide in patients with advanced heart failure. *Clin Transplant* 2018; **32**(1): 01.
166. Montgomery CL, Zuege DJ, Rolfson DB, et al. Implementation of population-level screening for frailty among patients admitted to adult intensive care in Alberta, Canada. *Can J Anaesth* 2019; **66**(11): 1310-9.
167. Montgomery E, Macdonald PS, Newton PJ, et al. Frailty as a Predictor of Mortality in Patients With Interstitial Lung Disease Referred for Lung Transplantation. *Transplantation* 2020; **104**(4): 864-72.
168. Morgello S, Gensler G, Sherman S, et al. Frailty in medically complex individuals with chronic HIV. *AIDS* 2019; **33**(10): 1603-11.
169. Morley JE, Malmstrom TK, Miller DK. A simple frailty questionnaire (FRAIL) predicts outcomes in middle aged African Americans. *J Nutr Health Aging* 2012; **16**(7): 601-8.
170. Mosquera C, Bermudez JM, Evans JL, Spaniolas K, MacGillivary DC, Fitzgerald TL. Frailty Predicts Failure to Rescue after Thoracoabdominal Operation. *J Am Coll Surg* 2018; **226**(6): 978-86.

171. Myers V, Broday DM, Steinberg DM, Yuval, Drory Y, Gerber Y. Exposure to particulate air pollution and long-term incidence of frailty after myocardial infarction. *Ann Epidemiol* 2013; **23**(7): 395-400.
172. Myers V, Drory Y, Gerber Y, Israel Study Group on First Acute Myocardial I. Clinical relevance of frailty trajectory post myocardial infarction. *Eur J Prev Cardiol* 2014; **21**(6): 758-66.
173. Myers V, Drory Y, Goldbourt U, Gerber Y. Multilevel socioeconomic status and incidence of frailty post myocardial infarction. *Int J Cardiol* 2014; **170**(3): 338-43.
174. Nampoothiri RV, Kasudhan KS, Patil AN, et al. Impact of frailty, melphalan pharmacokinetics, and pharmacogenetics on outcomes post autologous hematopoietic cell transplantation for multiple myeloma. *Bone Marrow Transplant* 2019; **54**(12): 2088-95.
175. Nastasi AJ, McAdams-DeMarco MA, Schrack J, et al. Pre-Kidney Transplant Lower Extremity Impairment and Post-Kidney Transplant Mortality. *Am J Transplant* 2018; **18**(1): 189-96.
176. Ness KK, Krull KR, Jones KE, et al. Physiologic frailty as a sign of accelerated aging among adult survivors of childhood cancer: a report from the St Jude Lifetime cohort study. *J Clin Oncol* 2013; **31**(36): 4496-503.
177. Ney M, Tangri N, Dobbs B, et al. Predicting Hepatic Encephalopathy-Related Hospitalizations Using a Composite Assessment of Cognitive Impairment and Frailty in 355 Patients With Cirrhosis. *Am J Gastroenterol* 2018; **113**(10): 1506-15.
178. Onen NF, Patel P, Baker J, et al. Frailty and Pre-Frailty in a Contemporary Cohort of HIV-Infected Adults. *J Frailty Aging* 2014; **3**(3): 158-65.
179. Onen NF, Shacham E, Stamm KE, Turner Overton E. Sexual behaviors and results of bacterial sexually transmitted infections testing among frail HIV-infected individuals. *J Int Assoc Physicians AIDS Care (Chic)* 2010; **9**(1): 30-3.
180. Oppenheim H, Paolillo EW, Moore RC, et al. Neurocognitive functioning predicts frailty index in HIV. *Neurology* 2018; **91**(2): e162-e70.
181. Oskutis MQ, Lauerman MH, Kufera JA, et al. Are frailty markers associated with serious thoracic and spinal injuries among motor vehicle crash occupants? *J Trauma Acute Care Surg* 2016; **81**(1): 156-61.
182. Painter P, Kuskowski M. A closer look at frailty in ESRD: getting the measure right. *Hemodial Int* 2013; **17**(1): 41-9.
183. Palmer KT, D'Angelo S, Harris EC, et al. Frailty, prefrailty and employment outcomes in Health and Employment After Fifty (HEAF) Study. *Occup Environ Med* 2017; **74**(7): 476-82.
184. Pamukcuoglu M, Bhatia S, Weisdorf DJ, et al. Hematopoietic Cell Transplant-Related Toxicities and Mortality in Frail Recipients. *Biol Blood Marrow Transplant* 2019; **25**(12): 2454-60.
185. Paolillo EW, Saloner R, Montoya JL, et al. Frailty in Comorbid HIV and Lifetime Methamphetamine Use Disorder: Associations with Neurocognitive and Everyday Functioning. *AIDS Res Hum Retroviruses* 2019; **35**(11-12): 1044-53.
186. Passias PG, Bortz CA, Pierce KE, et al. Decreased rates of 30-day perioperative complications following ASD-corrective surgery: A modified Clavien analysis of 3300 patients from 2010 to 2014. *J Clin Neurosci* 2019; **61**: 147-52.
187. Pathai S, Gilbert C, Weiss HA, et al. Frailty in HIV-infected adults in South Africa. *J Acquir Immune Defic Syndr* 2013; **62**(1): 43-51.
188. Pathai S, Lawn SD, Weiss HA, Cook C, Bekker LG, Gilbert CE. Retinal nerve fibre layer thickness and contrast sensitivity in HIV-infected individuals in South Africa: a case-control study. *PLoS One* 2013; **8**(9): e73694.

189. Pathai S, Shiels PG, Weiss HA, et al. Ocular parameters of biological ageing in HIV-infected individuals in South Africa: relationship with chronological age and systemic biomarkers of ageing. *Mech Ageing Dev* 2013; **134**(9): 400-6.
190. Pena FG, Theou O, Wallace L, et al. Comparison of alternate scoring of variables on the performance of the frailty index. *BMC Geriatr* 2014; **14**: 25.
191. Perez Fernandez M, Martinez Miguel P, Ying H, et al. Comorbidity, Frailty, and Waitlist Mortality among Kidney Transplant Candidates of All Ages. *Am J Nephrol* 2019; **49**(2): 103-10.
192. Perez-Zepeda MU, Avila-Funes JA, Gutierrez-Robledo LM, Garcia-Pena C. Frailty Across Age Groups. *J Frailty Aging* 2016; **5**(1): 15-9.
193. Pierce KE, Passias PG, Alas H, et al. Does Patient Frailty Status Influence Recovery Following Spinal Fusion for Adult Spinal Deformity?: An Analysis of Patients With 3-Year Follow-up. *Spine (Phila Pa 1976)* 2020; **45**(7): E397-E405.
194. Piggott DA, Muzaale AD, Varadhan R, et al. Frailty and Cause-Specific Hospitalization Among Persons Aging With HIV Infection and Injection Drug Use. *J Gerontol A Biol Sci Med Sci* 2017; **72**(3): 389-94.
195. Piggott DA, Varadhan R, Mehta SH, et al. Frailty, Inflammation, and Mortality Among Persons Aging With HIV Infection and Injection Drug Use. *J Gerontol A Biol Sci Med Sci* 2015; **70**(12): 1542-7.
196. Prince CS, Noren Hooten N, Mode NA, et al. Frailty in middle age is associated with frailty status and race-specific changes to the transcriptome. *Aging (Albany NY)* 2019; **11**(15): 5518-34.
197. Ramaty E, Maor E, Peltz-Sinvani N, et al. Low ALT blood levels predict long-term all-cause mortality among adults. A historical prospective cohort study. *European Journal of Internal Medicine* 2014; **25**(10): 919-21.
198. Ravindrarajah R, Lee DM, Pye SR, et al. The ability of three different models of frailty to predict all-cause mortality: results from the European Male Aging Study (EMAS). *Arch Gerontol Geriatr* 2013; **57**(3): 360-8.
199. Rees HC, Meister E, Mohler MJ, Klotz SA. HIV-Related Frailty Is Not Characterized by Sarcopenia. *Journal of the International Association of Providers of AIDS Care* 2016; **15**(2): 131-4.
200. Reid DBC, Daniels AH, Ailon T, et al. Frailty and Health-Related Quality of Life Improvement Following Adult Spinal Deformity Surgery. *World Neurosurgery* 2018; **112**: e548-e54.
201. Ribeiro SML, Morley JE, Malmstrom TK, Miller DK. Fruit and vegetable intake and physical activity as predictors of disability risk factors in African-American middle-aged individuals. *J Nutr Health Aging* 2016; **20**(9): 891-6.
202. Richardson AM, McCarthy DJ, hu J, et al. Predictors of Successful Discharge of Patients on Postoperative Day 1 After Craniotomy for Brain Tumor. *World Neurosurgery* 2019; **126**: e869-e77.
203. Rietman ML, Spijkerman AMW, Wong A, et al. Antioxidants linked with physical, cognitive and psychological frailty: Analysis of candidate biomarkers and markers derived from the MARK-AGE study. *Mech Ageing Dev* 2019; **177**: 135-43.
204. Rochira V, Diazzi C, Santi D, et al. Low testosterone is associated with poor health status in men with human immunodeficiency virus infection: a retrospective study. *Andrology* 2015; **3**(2): 298-308.
205. Rockwood K, Song X, Mitnitski A. Changes in relative fitness and frailty across the adult lifespan: evidence from the Canadian National Population Health Survey. *CMAJ Canadian Medical Association Journal* 2011; **183**(8): E487-94.

206. Rockwood MR, MacDonald E, Sutton E, Rockwood K, Baron M, Canadian Scleroderma Research G. Frailty index to measure health status in people with systemic sclerosis. *Journal of Rheumatology* 2014; **41**(4): 698-705.
207. Roshanravan B, Khatri M, Robinson-Cohen C, et al. A prospective study of frailty in nephrology-referred patients with CKD. *American Journal of Kidney Diseases* 2012; **60**(6): 912-21.
208. Rothenberg KA, Stern JR, George EL, et al. Association of Frailty and Postoperative Complications With Unplanned Readmissions After Elective Outpatient Surgery. *JAMA Network Open* 2019; **2**(5): e194330.
209. Rozenberg D, Mathur S, Wickerson L, Chowdhury NA, Singer LG. Frailty and clinical benefits with lung transplantation. *J Heart Lung Transplant* 2018; **37**(10): 1245-53.
210. Rubtsova AA, Marquine MJ, Depp C, et al. Psychosocial Correlates of Frailty Among HIV-Infected and HIV-Uninfected Adults. *Behavioral Medicine* 2018; **45**(3): 210-20.
211. Sabel MS, Lee J, Cai S, Englesbe MJ, Holcombe S, Wang S. Sarcopenia as a prognostic factor among patients with stage III melanoma. *Annals of Surgical Oncology* 2011; **18**(13): 3579-85.
212. Sadiq F, Kronzer VL, Wildes TS, et al. Frailty Phenotypes and Relations With Surgical Outcomes: A Latent Class Analysis. *Anesth Analg* 2018; **127**(4): 1017-27.
213. Sahli Z, Canner JK, Najjar O, et al. Association Between Age and Patient-Reported Changes in Voice and Swallowing After Thyroidectomy. *Laryngoscope* 2019; **129**(2): 519-24.
214. Salem BE. Characterizing Frailty among Homeless Adults: University of California, Los Angeles; 2013.
215. Salem BE, Nyamathi A, Brecht ML, et al. Constructing and identifying predictors of frailty among homeless adults-a latent variable structural equations model approach. *Arch Gerontol Geriatr* 2014; **58**(2): 248-56.
216. Salem BE, Nyamathi AM, Brecht ML, et al. Correlates of frailty among homeless adults. *West J Nurs Res* 2013; **35**(9): 1128-52.
217. Sandkovsky U, Robertson KR, Meza JL, et al. Pilot study of younger and older HIV-infected adults using traditional and novel functional assessments. *HIV Clin Trials* 2013; **14**(4): 165-74.
218. Schaenman J, Castellon L, Liang EC, et al. The Frailty Risk Score predicts length of stay and need for rehospitalization after kidney transplantation in a retrospective cohort: a pilot study. *Pilot Feasibility Stud* 2019; **5**: 144.
219. Schinkel-Ivy A, Mosca I, Mansfield A. Factors Contributing to Unexpected Retirement and Unemployment in Adults Over 50 Years Old in Ireland. *Gerontol Geriatr Med* 2017; **3**: 2333721417722709.
220. Schopmeyer L, El Moumni M, Nieuwenhuijs-Moeke GJ, Berger SP, Bakker SJL, Pol RA. Frailty has a significant influence on postoperative complications after kidney transplantation-a prospective study on short-term outcomes. *Transpl Int* 2019; **32**(1): 66-74.
221. Sharma A, Hoover DR, Shi Q, et al. Frailty as a predictor of falls in HIV-infected and uninfected women. *Antivir Ther* 2019; **24**(1): 51-61.
222. Sharma A, Shi Q, Hoover DR, et al. Frailty predicts fractures among women with and at-risk for HIV. *AIDS* 2019; **33**(3): 455-63.
223. Shin JI, Kothari P, Phan K, et al. Frailty Index as a Predictor of Adverse Postoperative Outcomes in Patients Undergoing Cervical Spinal Fusion. *Spine (Phila Pa 1976)* 2017; **42**(5): 304-10.

224. Singer JP, Diamond JM, Anderson MR, et al. Frailty phenotypes and mortality after lung transplantation: A prospective cohort study. *Am J Transplant* 2018; **18**(8): 1995-2004.
225. Singer JP, Diamond JM, Gries CJ, et al. Frailty Phenotypes, Disability, and Outcomes in Adult Candidates for Lung Transplantation. *Am J Respir Crit Care Med* 2015; **192**(11): 1325-34.
226. Smit E, Wanke C, Dong K, et al. Frailty, Food Insecurity, and Nutritional Status in People Living with Hiv. *J Frailty Aging* 2015; **4**(4): 191-7.
227. Smitherman AB, Anderson C, Lund JL, Bensen JT, Rosenstein DL, Nichols HB. Frailty and Comorbidities Among Survivors of Adolescent and Young Adult Cancer: A Cross-Sectional Examination of a Hospital-Based Survivorship Cohort. *J Adolesc Young Adult Oncol* 2018; **7**(3): 374-83.
228. Soma O, Hatakeyama S, Okamoto T, et al. Clinical implication of a quantitative frailty assessment tool for prognosis in patients with urological cancers. *Oncotarget* 2018; **9**(25): 17396-405.
229. Stanjek-Cichoracka A, Wozniak-Grygiel E, Laszewska A, Zembala M, Ochman M. Assessment of Cytokines, Biochemical Markers of Malnutrition and Frailty Syndrome Patients Considered for Lung Transplantation. *Transplant Proc* 2019; **51**(6): 2009-13.
230. Stenholm S, Strandberg TE, Pitkala K, Sainio P, Heliovaara M, Koskinen S. Midlife obesity and risk of frailty in old age during a 22-year follow-up in men and women: the Mini-Finland Follow-up Survey. *J Gerontol A Biol Sci Med Sci* 2014; **69**(1): 73-8.
231. Subramaniam S, Aalberg JJ, Soriano RP, Divino CM. New 5-Factor Modified Frailty Index Using American College of Surgeons NSQIP Data. *J Am Coll Surg* 2018; **226**(2): 173-81 e8.
232. Susanto M, Hubbard RE, Gardiner PA. Association of 12-Year Trajectories of Sitting Time With Frailty in Middle-Aged Women. *Am J Epidemiol* 2018; **187**(11): 2387-96.
233. Susanto M, Hubbard RE, Gardiner PA. Validity and Responsiveness of the FRAIL Scale in Middle-Aged Women. *J Am Med Dir Assoc* 2018; **19**(1): 65-9.
234. Swiecicka A, Lunt M, Ahern T, et al. Nonandrogenic Anabolic Hormones Predict Risk of Frailty: European Male Ageing Study Prospective Data. *J Clin Endocrinol Metab* 2017; **102**(8): 2798-806.
235. Sy J, McCulloch CE, Johansen KL. Depressive symptoms, frailty, and mortality among dialysis patients. *Hemodial Int* 2019; **23**(2): 239-46.
236. Sy J, Streja E, Grimes B, Johansen KL. The Marginal Cost of Frailty Among Medicare Patients on Hemodialysis. *Kidney Int Rep* 2020; **5**(3): 289-95.
237. Tajar A, O'Connell MD, Mitnitski AB, et al. Frailty in relation to variations in hormone levels of the hypothalamic-pituitary-testicular axis in older men: results from the European male aging study. *J Am Geriatr Soc* 2011; **59**(5): 814-21.
238. Tapper EB, Derstine B, Baki J, Su GL. Bedside Measures of Frailty and Cognitive Function Correlate with Sarcopenia in Patients with Cirrhosis. *Dig Dis Sci* 2019; **64**(12): 3652-9.
239. Tapper EB, Konerman M, Murphy S, Sonnenday CJ. Hepatic encephalopathy impacts the predictive value of the Fried Frailty Index. *Am J Transplant* 2018; **18**(10): 2566-70.
240. Telemi E, Trofymenko O, Venkat R, Pandit V, Pandian TK, Nfonsam VN. Frailty Predicts Morbidity after Colectomy for Ulcerative Colitis. *Am Surg* 2018; **84**(2): 225-9.
241. Traven SA, Horn RW, Reeves RA, Walton ZJ, Woolf SK, Slone HS. The 5-Factor Modified Frailty Index Predicts Complications, Hospital Admission, and Mortality Following Arthroscopic Rotator Cuff Repair. *Arthroscopy* 2020; **36**(2): 383-8.

242. Trung TN, Duoc NVT, Nhat LTH, et al. Functional outcome and muscle wasting in adults with tetanus. *Trans R Soc Trop Med Hyg* 2019; **113**(11): 706-13.
243. Tuttle LJ, Bittel DC, Bittel AJ, Sinacore DR. Early-Onset Physical Frailty in Adults With Diabesity and Peripheral Neuropathy. *Can J Diabetes* 2018; **42**(5): 478-83.
244. Van Pilsum Rasmussen S, Konel J, Warsame F, et al. Engaging clinicians and patients to assess and improve frailty measurement in adults with end stage renal disease. *BMC Nephrol* 2018; **19**(1): 8.
245. Vatanen A, Hou M, Huang T, et al. Clinical and biological markers of premature aging after autologous SCT in childhood cancer. *Bone Marrow Transplant* 2017; **52**(4): 600-5.
246. Venado A, McCulloch C, Greenland JR, et al. Frailty trajectories in adult lung transplantation: A cohort study. *J Heart Lung Transplant* 2019; **38**(7): 699-707.
247. Verheij E, Kirk GD, Wit FW. Frailty is associated with mortality and incident comorbidity among middle-aged HIV-positive and HIV-negative participants. *Care Management* 2020; **26**(1): 22-.
248. Verschoor CP, Tamim H. Frailty Is Inversely Related to Age at Menopause and Elevated in Women Who Have Had a Hysterectomy: An Analysis of the Canadian Longitudinal Study on Aging. *J Gerontol A Biol Sci Med Sci* 2019; **74**(5): 675-82.
249. Wade KF, Lee DM, McBeth J, et al. Chronic widespread pain is associated with worsening frailty in European men. *Age Ageing* 2016; **45**(2): 268-74.
250. Wallace LM, Theou O, Kirkland SA, et al. Accumulation of non-traditional risk factors for coronary heart disease is associated with incident coronary heart disease hospitalization and death. *PLoS One* 2014; **9**(3): e90475.
251. Wang CW, Lebsack A, Chau S, Lai JC. The Range and Reproducibility of the Liver Frailty Index. *Liver Transpl* 2019; **25**(6): 841-7.
252. Wang R, Shlipak MG, Ix JH, et al. Association of Fibroblast Growth Factor-23 (FGF-23) With Incident Frailty in HIV-Infected and HIV-Uninfected Individuals. *J Acquir Immune Defic Syndr* 2019; **80**(1): 118-25.
253. Warsame F, Ying H, Haugen CE, et al. Intradialytic Activities and Health-Related Quality of Life Among Hemodialysis Patients. *Am J Nephrol* 2018; **48**(3): 181-9.
254. Wen Y, Jabir MA, Dosokey EM, et al. Using Modified Frailty Index to Predict Safe Discharge Within 48 Hours of Ileostomy Closure. *Dis Colon Rectum* 2017; **60**(1): 76-80.
255. Wilkes JG, Evans JL, Prato BS, Hess SA, MacGillivray DC, Fitzgerald TL. Frailty Cost: Economic Impact of Frailty in the Elective Surgical Patient. *J Am Coll Surg* 2019; **228**(6): 861-70.
256. Williams DM, Jylhava J, Pedersen NL, Hagg S. A Frailty Index for UK Biobank Participants. *J Gerontol A Biol Sci Med Sci* 2019; **74**(4): 582-7.
257. Wilson CL, Chemaitilly W, Jones KE, et al. Modifiable Factors Associated With Aging Phenotypes Among Adult Survivors of Childhood Acute Lymphoblastic Leukemia. *J Clin Oncol* 2016; **34**(21): 2509-15.
258. Wilson JM, Holzgrefe RE, Staley CA, Schenker ML, Meals C. The Effect of Malnutrition on Postoperative Complications Following Surgery for Distal Radius Fractures. *J Hand Surg Am* 2019; **44**(9): 742-50.

259. Wilson JM, Lunati MP, Grabel ZJ, Staley CA, Schwartz AM, Schenker ML. Hypoalbuminemia Is an Independent Risk Factor for 30-Day Mortality, Postoperative Complications, Readmission, and Reoperation in the Operative Lower Extremity Orthopaedic Trauma Patient. *J Orthop Trauma* 2019; **33**(6): 284-91.

260. Wilson ME, Vakil AP, Kandel P, Undavalli C, Dunlay SM, Kennedy CC. Pretransplant frailty is associated with decreased survival after lung transplantation. *J Heart Lung Transplant* 2016; **35**(2): 173-8.

261. Womack JA, Goulet JL, Gibert C, et al. Physiologic frailty and fragility fracture in HIV-infected male veterans. *Clin Infect Dis* 2013; **56**(10): 1498-504.

262. Wulunggono W, Yunihastuti E, Shatri H, Wahyudi ER, Ophinni Y. Frailty among HIV-1 Infected Adults under Antiretroviral Therapy in Indonesia. *Curr HIV Res* 2019; **17**(3): 204-13.

263. Xu W, Zhang XM, Ke T, Cai HR, Gao X. Modified Frailty Index and Body Mass Index as Predictors of Adverse Surgical Outcomes in Degenerative Spinal Disease. *Turk Neurosurg* 2018; **28**(6): 897-903.

264. Yadla M, John JP, Mummadi M. A study of clinical assessment of frailty in patients on maintenance hemodialysis supported by cashless government scheme. *Saudi J Kidney Dis Transpl* 2017; **28**(1): 15-22.

265. Yagi M, Michikawa T, Hosogane N, et al. Treatment for Frailty Does Not Improve Complication Rates in Corrective Surgery for Adult Spinal Deformity. *Spine (Phila Pa 1976)* 2019; **44**(10): 723-31.

266. Yagi M, Michikawa T, Hosogane N, et al. The 5-Item Modified Frailty Index Is Predictive of Severe Adverse Events in Patients Undergoing Surgery for Adult Spinal Deformity. *Spine (Phila Pa 1976)* 2019; **44**(18): E1083-E91.

267. Yi C, Lin J, Cao P, et al. Prevalence and Prognosis of Coexisting Frailty and Cognitive Impairment in Patients on Continuous Ambulatory Peritoneal Dialysis. *Sci Rep* 2018; **8**(1): 17305.

268. Young AC, Glaser K, Spector TD, Steves CJ. The Identification of Hereditary and Environmental Determinants of Frailty in a Cohort of UK Twins. *Twin Res Hum Genet* 2016; **19**(6): 600-9.
