# Supplementary material for: Measuring frailty in younger populations: a rapid review of evidence
Source: BMJ Open. 2021 Mar 22;11(3):e047051. doi: 10.1136/bmjopen-2020-047051 (PMC7986767; doi:10.1136/bmjopen-2020-047051)
Supplement: Author's manuscript [file bmjopen-2020-047051.draft_revisions.pdf]

BMJ Open

BMJ Open is committed to open peer review. As part of this commitment we make the peer review history of every article we publish publicly available.

When an article is published we post the peer reviewers' comments and the authors' responses online. We also post the versions of the paper that were used during peer review. These are the versions that the peer review comments apply to.

The versions of the paper that follow are the versions that were submitted during the peer review process. They are not the versions of record or the final published versions. They should not be cited or distributed as the published version of this manuscript.

BMJ Open is an open access journal and the full, final, typeset and author-corrected version of record of the manuscript is available on our site with no access controls, subscription charges or pay-per-view fees (http://bmjopen.bmj.com).

If you have any questions on BMJ Open's open peer review process please email info.bmjopen@bmj.com

BMJ Open

Measuring frailty in younger populations: a rapid review of evidence

Journal:	*BMJ Open*
Manuscript ID	bmjopen-2020-047051
Article Type:	Original research
Date Submitted by the Author:	18-Nov-2020
Complete List of Authors:	Spiers, Gemma; Newcastle University, Population & Health Sciences Institute<br>Kunonga, Tafadzwa; Newcastle University, Population & Health Sciences Institute<br>Hall, Alex; University of Manchester, Division of Nursing, Midwifery & Social Work; School of Health Sciences<br>Beyer, Fiona ; Newcastle University,<br>Boulton, Elisabeth; University of Manchester, School of Health Sciences; Manchester Academic Health Science Centre,<br>Parker, Stuart; Newcastle University, Institute of Health and Society / Newcastle University Institute for Ageing<br>Bower, Peter; University of Manchester, NPCRDC<br>Craig, Dawn; Institute of Health & Society, Newcastle University, Health Economics Group, Institute of Health and Society<br>Todd, Chris; University of Manchester, School of Health Sciences<br>Hanratty, Barbara; Newcastle University, Institute of Health and Society / Newcastle University Institute for Ageing
Keywords:	GENERAL MEDICINE (see Internal Medicine), PRIMARY CARE, Health policy < HEALTH SERVICES ADMINISTRATION & MANAGEMENT

SCHOLARONE™
Manuscripts

I, the Submitting Author has the right to grant and does grant on behalf of all authors of the Work (as defined in the below author licence), an exclusive licence and/or a non-exclusive licence for contributions from authors who are: i) UK Crown employees; ii) where BMJ has agreed a CC-BY licence shall apply, and/or iii) in accordance with the terms applicable for US Federal Government officers or employees acting as part of their official duties; on a worldwide, perpetual, irrevocable, royalty-free basis to BMJ Publishing Group Ltd ("BMJ") its licensees and where the relevant Journal is co-owned by BMJ to the co-owners of the Journal, to publish the Work in this journal and any other BMJ products and to exploit all rights, as set out in our licence.

The Submitting Author accepts and understands that any supply made under these terms is made by BMJ to the Submitting Author unless you are acting as an employee on behalf of your employer or a postgraduate student of an affiliated institution which is paying any applicable article publishing charge ("APC") for Open Access articles. Where the Submitting Author wishes to make the Work available on an Open Access basis (and intends to pay the relevant APC), the terms of reuse of such Open Access shall be governed by a Creative Commons licence – details of these licences and which Creative Commons licence will apply to this Work are set out in our licence referred to above.

Other than as permitted in any relevant BMJ Author's Self Archiving Policies, I confirm this Work has not been accepted for publication elsewhere, is not being considered for publication elsewhere and does not duplicate material already published. I confirm all authors consent to publication of this Work and authorise the granting of this licence.

For peer review only

Measuring frailty in younger populations: a rapid review of evidence

Dr Gemma Spiers[a]* gemma-frances.spiers@newcastle.ac.uk
Ms Tafadzwa Patience Kunonga[a]
Dr Alex Hall [b]
Ms Fiona Beyer [a]
Dr Elisabeth Boulton [b]
Professor Stuart Parker [a]
Professor Peter Bower [b]
Professor Dawn Craig [a]
Professor Chris Todd [b]
Professor Barbara Hanratty[a]

a. National Institute for Health Research (NIHR) Older People and Frailty Policy Research Unit, Population Health Sciences Institute, Newcastle University, Newcastle-upon-Tyne, NE4 5PL, UK.

b. National Institute for Health Research (NIHR) Older People and Frailty Policy Research Unit, School of Health Sciences, Faculty of Biology, Medicine and Health, The University of Manchester, Manchester, M13 9PL, UK.

*Corresponding author

For peer review only

Funding & Acknowledgements

This report presents independent research funded by the National Institute for Health Research Policy Research Unit in Older People and Frailty (PR-PRU-1217-21502). The views expressed are those of the author(s) and not necessarily those of the NIHR or the Department of Health and Social Care.

Conflict of interest

No conflicts of interest.

Keywords: Frailty, younger populations, measurement

Words (excluding abstract, tables, figures and references): 3498
Tables and figures: 3 (additional tables and figures in supplementary materials)

Author contributions
GS co-designed the protocol, undertook all stages of the review, and co-wrote the paper. PK co-designed the protocol, undertook all stages of the review and co-wrote the paper. AH co-designed the protocol, undertook all stages of the review, and co-wrote the paper. FB co-designed the protocol, undertook the searches for the review and co-wrote the paper. EB co-designed the protocol and co-wrote the paper. SP co-designed the protocol and co-wrote the paper. PB co-designed the protocol, contributed to the analysis of review data, and co-wrote the paper. DC co-designed the protocol, contributed to the analysis of review data, and co-wrote the paper. CT co-designed the protocol, and co-wrote the paper. BH co-designed the protocol, and co-wrote the paper.

For peer review only - http://bmjopen.bmj.com/site/about/guidelines.xhtml

Measuring frailty in younger populations: a rapid review of evidence

Objectives

Frailty is typically assessed in older populations. Identifying frailty in adults aged under 60 years may also have value, if it supports the delivery of timely care. We sought to identify how frailty is measured in younger populations, including evidence of the impact on patient outcomes and care.

Design

A rapid review of primary studies was conducted. Four databases, three sources of grey literature and reference lists of systematic reviews were searched for studies that measured frailty in populations aged under 60 years using experimental or observational designs, published after 2000 in English. Records were screened against review criteria. Study data were extracted with 20% of records checked for accuracy by a second researcher. Data were synthesised using a narrative approach.

Results

We identified 268 studies that measured frailty in samples that included people aged under 60 years. Of these, 85 studies reported evidence about measure validity. No measures were identified that were designed and validated to identify frailty *exclusively* in younger groups. However, in populations that included people aged over *and* under 60 years, cumulative deficit frailty indices, phenotype measures, the FRAIL scale, the Liver Frailty Index, and the Short Physical Performance Battery all demonstrated predictive validity for mortality and/or hospital admission. Evidence of criterion validity was rare. The extent to which measures possess validity across the younger adult age (18 to 59) spectrum was unclear. There was no evidence about the impact of measuring frailty in younger populations on patient outcomes and care.

Conclusions

Limited evidence suggests that frailty measures have predictive validity in younger populations. Further research is needed to clarify the validity of measures across the adult age spectrum, and explore the utility of measuring frailty in younger groups.

For peer review only - http://bmjopen.bmj.com/site/about/guidelines.xhtml

Strengths and limitations of this study

- Comprehensive and systematic searches captured the core evidence about measuring frailty in younger populations
- Predictive validity was assessed using known outcomes of frailty in older populations as far less is known about the consequences of frailty in younger groups
- The focus on younger groups offers a novel addition to our understanding of frailty measurement

For peer review only

For peer review only - http://bmjopen.bmj.com/site/about/guidelines.xhtml

Background

Frailty is characterised by increased vulnerability to stressors, and has been described as a problematic expression of population ageing.[1, 2] The presence of frailty is associated with a number of adverse outcomes, including lower quality of life and an increased risk of functional decline, admission to hospital or long-term care and mortality.[3-6] Recent estimates place the global incidence of frailty at 43.4 cases per 1000 person-years, although there is substantial variation by country-level income and disease specific populations.[7]

Debates about how to measure and operationalise frailty have led to two dominant models: the phenotype model and the cumulative deficit model. The phenotype model, which was conceptualised through clinical observation and epidemiological study, operationalises frailty as the presence of three or more of the following criteria: exhaustion, weight loss, weakness/loss of muscular strength, reduced gait speed, and reduced energy/physical activity.[8] In contrast, the cumulative deficit model, which was developed through consideration of biological theories of ageing,[9] considers frailty an accumulation of deficits including clinical signs and symptoms, diseases, and disability. In this model, 'the more things somebody has wrong with them, the more likely they are to be frail'.[10] (p.722)

There are numerous tools to measure frailty, which are not necessarily interchangeable.[11] A recent review of measures in older populations identified 51 separate instruments.[12] Although many measures of frailty are informed by the cumulative deficit or phenotype models, there is still much variation in the content of these instruments.[11] In one analysis of 35 frailty measures, there were varying levels of agreement for identifying who was frail between tools.[13] There are further challenges in assessing the risk of frailty in clinical settings, where different approaches exist across primary, secondary and specialist care.[2] For example, the Clinical Frailty Scale, gait speed, PRISMA-7 and timed-up-and-go tests are all validated tools to assess the risk of frailty in older populations in clinical care.[14-16] These numerous instruments present challenges to researchers and clinicians when choosing how to conceptualise, measure and assess frailty.[12]

The prevalence of frailty increases with age and so it is typically assessed in older populations. In 2019, the International Conference of Frailty and Sarcopenia Research Task Force recommended routine frailty screening in populations aged over 65 years.[17] This reflects current clinical guidance in the UK for example, where screening for frailty in populations aged over 65 is routine in primary care, and also forms part of the Comprehensive Geriatric Assessment in secondary care settings.[15] Such routine screening is part of efforts to support people as they age.

For peer review only - http://bmjopen.bmj.com/site/about/guidelines.xhtml

The utility of identifying frailty in adults aged under 60 years also warrants scrutiny. Some vulnerable groups with chronic conditions may be at risk of becoming frail earlier in life, whilst the onset of illness and disability occurs at younger ages for people living in areas of greater deprivation. [18-20] There is, therefore, potential for younger groups to be classed as frail, and identification may aid the delivery of timely care. Furthermore, studies are increasingly measuring frailty prevalence across the life span.[21-23] Yet current debates and evidence about how to measure frailty remain focused on older populations. A number of systematic reviews have examined the validity of various frailty measures, most recently in 2019.[24-26] These reviews focus on the measurement of frailty in older populations, and there remains a lack of clarity about the validity of frailty measures in younger groups.

To address this gap, we aimed to synthesise evidence on how frailty is measured in younger populations (aged < 60 years). Specifically, we sought evidence to address the following questions:

a. Are there any validated tools to identify frailty specifically in younger populations?

b. How are existing tools to identify frailty in older populations used to measure and identify frailty in younger age groups, and are such tools validated in these younger populations?

c. How is frailty identified in younger people with long term or life limiting conditions?

d. In what other ways (other than a validated tool) is frailty in younger populations currently operationalised and identified from existing data in observational and experimental studies?

e. Is there any evidence of impact (on patient outcomes, staff, clinical management or health service utilisation) of identifying frailty in younger populations, including identification of opportunities for intervention?

Methods

We conducted a rapid review of primary studies. Rapid reviews offer a streamlined version of standard systematic review methods in a shorter timeframe, with the resultant output a summary, rather than in-depth synthesis, of evidence.[27] There is no agreed methodology for the conduct of rapid reviews, but for the purposes of this work, rapid methods included: systematic searches, double screening of titles and abstracts for 5% of studies to pilot the eligibility criteria; single researcher screening of full texts; second checking of data extraction for 20% of studies; and, omitting an assessment of study quality.

For peer review only - http://bmjopen.bmj.com/site/about/guidelines.xhtml

Search strategy

A search strategy was developed, piloted and tested, based on three concepts: frailty, measurement and age (see supplementary materials for the strategy applied to MEDLINE). The search strategy excluded studies that were indexed with terms for older people, unless the record also contained indexing terms pertaining to a younger population ('middle aged' or 'young adult').

Searches were carried out in: MEDLINE (OVID); PsycINFO (OVID); CINAHL (EBSCO); Science Citation Index (Web of Knowledge); Social Science Citation (Web of Knowledge). Three sources of grey literature were also searched: OpenGrey,[28] Social Care Online,[29] and the National Institute of Health and Care Excellence.[30] References of relevant systematic reviews were also checked.

Review criteria

Eligible for inclusion were primary research studies that measured frailty in populations aged under 60 years, and published after 2000 in English, using experimental or observational study designs (table 1). The age threshold of below 60 years was selected, as opposed to 65 years, as initial scoping indicated that studies on frailty in older populations typically used samples aged 60 years and over. Studies that included those aged 60 years and over were included only if the majority of sample was aged under 60 years. Where it was not possible to determine this majority, studies were included if the mean or median sample age was between 18 and 59.9 years.

For peer review only

For peer review only - http://bmjopen.bmj.com/site/about/guidelines.xhtml

Table 1. Review inclusion/exclusion criteria

	Include	Exclude
**Population**	Adults aged 18-59.9 years.<br>Studies that include those aged ≥60 years if the majority of sample is aged under 60 years. Where it is not possible to determine if the majority of the sample are aged under 60 years, studies where the mean/median sample age was under 60 years were included.<br><br>Any condition/diagnosis.	Studies of people aged ≥60 years only, or where the majority of the study sample is aged >60 years.
**Exposure/outcome**	Any measure of frailty that is validated, including those currently used in 65+ populations and those developed specifically for younger age groups.<br><br>Any approach to identifying frailty from existing data.<br><br>Frailty may be either the exposure (e.g. if using it to predict other outcomes) or the outcome (e.g. if estimating prevalence).	
**Study design**	Observational or experimental designs.<br><br>Published in English after 2000.	

Study selection

Title and abstract screening was piloted by three researchers (AH, PK, GS) independently on 5% of records. Decisions were compared and discussed to resolve inconsistencies before proceeding. The titles and abstracts of the remaining 95% of records were then screened by single researchers (AH, PK, GS). The full texts of selected records were retrieved and assessed for inclusion against the review criteria. For studies that reported details of a validated frailty measure, the referenced publication was also retrieved and assessed against the review criteria.

Data extraction and synthesis

A data extraction form was developed and piloted. Summary study information was extracted: author and date; country; measure used; sample age (mean, median and range where reported); and long-term condition of sample (if applicable). Included studies were further scrutinised to identify those that reported evidence that indicated whether the measure was validated. To ascertain validity, we adapted criteria used in a previous review of frailty measures in older populations.[31] That is, the validity of a frailty measure was ascertained through evidence that:

For peer review only - http://bmjopen.bmj.com/site/about/guidelines.xhtml

i. the measure predicted mortality and/or hospitalisations (*predictive validity*)

ii. the measure was associated with another validated measure of frailty (*criterion validity*)

Predictive validity indicates whether the measure can predict outcomes of relevance. Mortality and hospital admissions were chosen as outcomes for the test of predictive validity as these are commonly associated with frailty.[32-35] Criterion validity indicates whether the new measure is assessing what it intends to, by comparing it to a validated measure of the same concept.[31, 36] This is achieved by assessing the association between the two measures (e.g. correlation or regression), or the extent to which two measures produce the same results (agreement).

For studies that reported evidence about predictive and/or criterion validity, data were extracted about: the measure (e.g. items, cut off scores used); whether the evidence indicated validity, including prediction estimates; and any evidence about the impact of measuring frailty in younger populations on patient outcomes, staff, clinical management or health service utilisation (e.g. if a measure was trialled or evaluated). Data were extracted by single researchers (AH, PK, GS), with 20% checked by a second for accuracy.

A narrative synthesis was used to summarise what measures of frailty were used and which measures demonstrated evidence of validity (in which populations). To facilitate this, each study that reported evidence about validity was allocated to a colour coded framework using the criteria described in supplementary materials table S1. Finally, we summarised evidence about any reported impact of measuring frailty in younger populations on patient outcomes and care.

Patient and public involvement

It was not possible to involve patients or the public in the design and conduct of this rapid review.

Findings

After screening, 268 publications met the review criteria (figure 1, supplementary materials table S2; full bibliographic details of the included studies are also listed in the supplementary materials).

Figure 1 here

Most studies used samples aged under *and* over 60 years. A minority of studies (n=6) used samples aged entirely below 60 years.[37-42] Just over 70% of studies used study samples with an average age between 51 and 59 years, whilst 24.1% used samples with an average age of between 41 to 50 years.

For peer review only - http://bmjopen.bmj.com/site/about/guidelines.xhtml

Few studies measured frailty in samples with an average age below 40 years (figure 2). Thus, when referring to *younger populations* in these findings, this typically refers to the older end of the 18 to 59 year spectrum.

Figure 2 here

How was frailty measured in younger populations?

Across studies, 41 measures of frailty were identified (supplementary materials table S3). The most common were phenotype measures or cumulative deficit frailty indices. Some studies measured frailty using single, or a combination of, indicators, such as sarcopenia with vascular disease, or muscle mass. A small number of studies claimed to measure frailty but used tools not designed for this purpose (the Katz Index, the ADL Scale, the Karnofsky Performance Scale, and the Braden Scale). The studies reporting these tools were therefore not included in this review.

Validity of frailty measures in younger populations

A subset of 85 studies reported evidence about the predictive and/or criterion validity of their chosen frailty measure (supplementary materials table S4).[21-23, 43-124]

Across these studies, 13 measures were used (32 in total when accounting for different versions of cumulative deficit frailty indices). These measures included cumulative deficit frailty indices, phenotype measures, the FRAIL Scale, the Short Physical Performance Battery, the Clinical Frailty Scale, the Liver Frailty Index, the John Hopkins Frailty Indicator, and the SOF Frailty Scale and CHS Frailty Scale.[21-23, 43-94, 96-124] Although termed a 'Liver Frailty Index', this measure did not comprise items that were specific to liver disease. Three measures were individual markers of frailty (muscle mass, walk test and hand grip), each used in isolation and not as part of a multiple item measure.[56, 59, 69, 82] One measure was a surgical risk index, which incorporated a frailty assessment.[95]

All measures were used in samples aged over and under 60 years; no measures were identified that were designed and validated to identify frailty *exclusively* in younger (18 to 59.9 years) populations.

Supplementary materials table S4 summarises the evidence for each study. Evidence of criterion validity, where a measure was compared to a 'gold standard' validated frailty measure, was sparse. Most studies reported evidence about the association between the measure of frailty and mortality and/or hospital admissions, which we used to infer predictive validity.

There was evidence of predictive validity for cumulative deficit frailty indices and phenotype measures in general populations, diagnostic specific populations (HIV, systemic lupus

For peer review only - http://bmjopen.bmj.com/site/about/guidelines.xhtml

erythematosus, metabolic syndrome, survivors of myocardial infarction, childhood survivors of cancer, chronic kidney or end stage renal disease) and surgical populations (hematopoietic cell transplant, or other non-specified surgery).[22, 23, 43, 46, 51, 52, 55, 58, 62, 65-67, 72, 76, 77, 80, 81, 83, 88-93, 99, 101, 105, 110, 112, 114-116, 118, 121, 123-125] For some populations, evidence of predictive validity was inconsistent for a cumulative deficit frailty index (intellectual disability),[94, 105] and phenotype measures (heart failure and those undergoing lung or kidney transplant).[73, 74, 78, 96-98, 106, 108, 117, 119, 120]

There was also evidence of predictive validity for the FRAIL scale (general and diabetic populations),[53, 54, 64, 93, 109, 116, 122] the Liver Frailty Index (liver disease),[68, 84, 85, 87] and the Short Physical Performance Battery (populations undergoing lung or kidney transplants and those with end stage liver disease).[57, 86, 111, 119, 120]

For the remaining measures, there was either no evidence of predictive validity, or evidence was too inconsistent or uncertain to draw a conclusion.

In terms of whether the validity of a measure varied by age, only two studies stratified their analysis of prediction by age group. One study showed that a cumulative deficit frailty index based exclusively on laboratory test-based indicators of illness predicted mortality only in groups aged 40 to 65 and not those aged 20 to 39 years. In the same study, a cumulative deficit frailty index based entirely on self-reported items predicted mortality in both age groups, but prediction was statistically less certain for those aged 20 to 39 years.[21] In the second study, higher levels of frailty predicted mortality at all ages, but for the younger group (<39 years), differences in survival were only statistically significant between the least and most frail. We further explored the role of age by comparing evidence across studies according to the mean or median sample age (where this was reported). We used this approach for the two most commonly used measures: the frailty indices and the phenotype measures (supplementary materials figures S1 and S2). With very few studies involving participants with an average age below 40 years, there was insufficient evidence to assess how the validity of these measures varied according to the average age of the sample in which the measure was tested.

Evidence of impact of measuring frailty in younger populations

No studies were identified that evaluated the impact of measuring frailty in younger populations on patient outcomes, staff, clinical care, or service utilisation.

Discussion

This review identified no measures that were designed and validated to measure frailty *only* in younger populations. However, in study populations that included people aged under 60 years,

For peer review only - http://bmjopen.bmj.com/site/about/guidelines.xhtml

there was some evidence that cumulative deficit frailty indices, phenotype measures, the FRAIL scale, the Short Physical Performance Battery, and the Liver Frailty Index have predictive validity. These measures demonstrated validity in both general and single diagnosis populations, and those undergoing some types of surgical procedures. Evidence of predictive validity for other measures was either absent or less certain, and evidence of criterion validity was sparse.

Overall the evidence suggests that some measures allow for the identification of frailty in younger populations. However, a more cautious interpretation should consider the role of sample age. Most studies used populations that included people aged over and under 60 years of age. We also know that the prevalence of frailty is higher in older, rather than younger, groups.[126, 127] Therefore, it is possible that any observed predictive validity was due to higher prevalence of frailty among older participants. Only two studies stratified their analysis of prediction by age group: both showed that the ability of the frailty measures to predict mortality was inconsistent between younger and older age groups.[21, 23] We are also aware of another study that showed a phenotype measure does not predict mortality for women aged 37 to 45 years.[128] However, this study was not included in our review as it was not possible to determine if the majority of the sample were aged under 60 years. Furthermore, most study populations were aged, on average, over 40 years. Therefore, when concluding that measures observed validity in younger populations, we are unclear to what extent this reflects validity for entire 18 to 59 year adult age spectrum.

We identified no studies that evaluated the impact of assessing frailty in younger populations. In many of the studies, there was speculation about the proposed benefits of assessing frailty, such as investigation of patient health status;[66, 109, 122] patient prognosis;[54, 61, 64, 70, 129] risk stratification and pre-operative decision-making;[60-63, 65, 68, 84-86, 92, 98, 101, 108] identifying early or preventive intervention;[46, 64, 92, 125] and patient counselling.[43, 44, 48, 60, 92] Despite these speculations, evidence is needed to clearly establish whether assessing frailty in younger groups does indeed make a difference to patient care in the ways suggested. The absence of any evidence about the impact of measuring frailty is not specific to younger populations. Others have noted the need for evidence about the utility of frailty instruments in practice and whether such tools usefully inform clinical decision making in care for older populations.[2]

A final observation about these findings concerns terminology. We identified a small number of studies where frailty was measured using tools designed for quantifying dependency and in one case, risk of pressure ulcers.[129-131] Whilst dependency and frailty are related,[132-134] they are not conceptually equivalent, and the use of these tools was not clearly justified. The use of these measures signals either some conceptual ambiguity about the nature of frailty, or what Bouillon and

For peer review only

colleagues, in their 2013 review of frailty measures in older populations, call 'a lack of terminological rigour' (p.4).[25] The implications for this review were that we were unable to reliably interpret any evidence about the validity of these tools as measures of frailty, and were thus excluded.

Strengths and limitations

The primary aim of this review was to identify how frailty was measured in younger populations, and the validity of such measures. A comprehensive search strategy using electronic databases, grey literature, systematic reviews and publications about measure validation referenced within included studies, has captured the core literature to achieve this aim.

Our conclusions about validity are based on a synthesis of evidence that predominantly concerns the ability of measures to predict mortality and hospital admissions. We chose these outcomes to assess predictive validity as they are known consequences of frailty in older groups.[32-35] We extended this assumption to younger populations but we acknowledge the limitations of this: evidence about outcomes of frailty *specifically* in younger populations is far less established. We also judged predictive validity based on the statistical significance of prediction. This facilitated an efficient summary assessment of evidence for this rapid review. Future work could add further insight into the validity of measures by considering the size and strength of prediction, particularly if more studies that stratify analyses by age group become available. A quality assessment, omitted here to achieve a rapid synthesis, will further distinguish the most robust evidence about predictive validity.

Finally, our focus on frailty measurement in younger populations was operationalised by excluding studies where the majority were aged, or the average sample age was, 60 years and over. We used the age threshold of 60, and not 65, years, as initial scoping indicated that studies on frailty in older populations typically used samples aged 60 years and over. This criterion has inevitably excluded studies where frailty was measured in samples that were, on average, older than 60 years but nonetheless included younger groups. However, this criterion was necessary to focus on evidence about younger groups and to avoid duplicating existing reviews of frailty measurement in older populations. By using this approach, we have produced a novel review and addressed a notable gap in our understanding of frailty measurement.

Implications for practice and future research

Identifying frailty in younger groups may have value if it aids the delivery of care for those who experience age-related illness and disability earlier in life. However, we identified no evidence about the impact of measuring frailty in younger groups, and the utility of doing so remains

For peer review only - http://bmjopen.bmj.com/site/about/guidelines.xhtml

uncertain. Even if frailty assessment in healthcare is extended to those aged under 65, clearer evidence is needed about the validity of frailty measures *across* the younger adult age spectrum.

It is also worth noting that the measures identified in this review were no different to those used with older populations. This may reflect an assumption that frailty is homogenous across older and younger groups, or it may simply be driven by the availability of frailty measures. Without evidence about the impact of measuring frailty in younger populations, it is impossible to judge whether this is appropriate. This also presents a question about construct validity: is frailty – a concept normally associated with ageing – the same, and thus measurable using existing tools, in younger populations? These are important issues to address and clarify should the assessment of frailty in younger populations be pursued in healthcare policy and clinical practice.

Conclusions and implications

Approaches to measuring frailty in younger groups mirrored those used in older populations. Some of these measures of frailty demonstrated predictive validity in samples that *included* those aged under 60 years. However, further evidence is needed to draw clear conclusions about the validity of these measures across the adult age spectrum. Despite the potential utility of identifying frailty in younger populations evidence about the impact on patient outcomes, staff, clinical management and health service utilisation is needed.

For peer review only

For peer review only - http://bmjopen.bmj.com/site/about/guidelines.xhtml

Conflict of interest

No conflicts of interest.

References

1. Clegg, A, Young, J, Iliffe, S, et al. Frailty in elderly people. The Lancet 2013;381(9868):752-762.
2. Hoogendijk, EO, Afilalo, J, Ensrud, KE, et al. Frailty: implications for clinical practice and public health. The Lancet 2019;394(10206):1365-1375.
3. Cunha, AIL, Veronese, N, de Melo Borges, S, et al. Frailty as a predictor of adverse outcomes in hospitalized older adults: A systematic review and meta-analysis. Ageing Res Rev 2019;56:100960.
4. Song, X, Mitnitski, A, Rockwood, K. Prevalence and 10-Year Outcomes of Frailty in Older Adults in Relation to Deficit Accumulation. Journal of the American Geriatrics Society 2010;58(4):681-687.
5. Bagshaw, SM, Stelfox, HT, Johnson, JA, et al. Long-term association between frailty and health-related quality of life among survivors of critical illness: a prospective multicenter cohort study. Crit Care Med 2015;43(5):973-982.
6. Puts, MTE, Lips, P, Ribbe, MW, et al. The effect of frailty on residential/nursing home admission in the Netherlands independent of chronic diseases and functional limitations. European journal of ageing 2005;2(4):264-274.
7. Ofori-Asenso, R, Chin, KL, Mazidi, M, et al. Global Incidence of Frailty and Prefrailty Among Community-Dwelling Older Adults: A Systematic Review and Meta-analysis. JAMA Network Open 2019;2(8):e198398-e198398.
8. Fried, LP, Tangen, CM, Walston, J, et al. Frailty in older adults: evidence for a phenotype. The journals of gerontology Series A, Biological sciences and medical sciences 2001;56(3):M146-156.
9. Mitnitski, AB, Mogilner, AJ, Rockwood, K. Accumulation of deficits as a proxy measure of aging. ScientificWorldJournal 2001;1:323-336.
10. Rockwood, K, Mitnitski, A. Frailty in Relation to the Accumulation of Deficits. The Journals of Gerontology: Series A 2007;62(7):722-727.
11. Walston, J, Buta, B, Xue, QL. Frailty Screening and Interventions: Considerations for Clinical Practice. Clin Geriatr Med 2018;34(1):25-38.
12. Faller, JW, Pereira, DdN, de Souza, S, et al. Instruments for the detection of frailty syndrome in older adults: A systematic review. PloS one 2019;14(4):e0216166-e0216166.
13. Aguayo, GA, Donneau, A-F, Vaillant, MT, et al. Agreement Between 35 Published Frailty Scores in the General Population. American Journal of Epidemiology 2017;186(4):420-434.
14. University of York, Yorkshire & Humber AHSN. Recognising and managing frailty in primary care. University of York; 2017.
15. NHS England. NHS RightCare: Frailty Toolkit. NHS England; 2019.
16. NHS England. Supporting routine frailty identification and frailty through the GP Contract 2017/18. NHS England; 2019.
17. Dent, E, Morley, JE, Cruz-Jentoft, AJ, et al. Physical Frailty: ICFSR International Clinical Practice Guidelines for Identification and Management. The journal of nutrition, health & aging 2019;23(9):771-787.
18. Chamberlain, AM, Finney Rutten, LJ, Wilson, PM, et al. Neighborhood socioeconomic disadvantage is associated with multimorbidity in a geographically-defined community. BMC Public Health 2020;20(1):13.

For peer review only - http://bmjopen.bmj.com/site/about/guidelines.xhtml

19. Matthews, RJ, Smith, LK, Hancock, RM, et al. Socioeconomic factors associated with the onset of disability in older age: a longitudinal study of people aged 75 years and over. Social Science & Medicine 2005;61(7):1567-1575.
20. van Groenou, MIB, Deeg, DJH, Penninx, BWJH. Income differentials in functional disability in old age: Relative risks of onset, recovery, decline, attrition and mortality. Aging Clinical and Experimental Research 2003;15(2):174-183.
21. Blodgett, JM, Theou, O, Howlett, SE, et al. A frailty index from common clinical and laboratory tests predicts increased risk of death across the life course. Geroscience 2017;39(4):447-455.
22. Jayanama, K, Theou, O, Blodgett, JM, et al. Frailty, nutrition-related parameters, and mortality across the adult age spectrum. BMC Med 2018;16(1):188.
23. Rockwood, K, Song, X, Mitnitski, A. Changes in relative fitness and frailty across the adult lifespan: evidence from the Canadian National Population Health Survey. CMAJ Canadian Medical Association Journal 2011;183(8):E487-494.
24. de Vries, NM, Staal, JB, van Ravensberg, CD, et al. Outcome instruments to measure frailty: A systematic review. Ageing Research Reviews 2011;10(1):104-114.
25. Bouillon, K, Kivimaki, M, Hamer, M. Measures of frailty in population-based studies: an overview. BMC Geriatr 2013;13.
26. Faller, JW, Pereira, DdN, de Souza, S, et al. Instruments for the detection of frailty syndrome in older adults: A systematic review. PLOS ONE 2019;14(4):e0216166.
27. Tricco, AC, Antony, J, Zarin, W, et al. A scoping review of rapid review methods. BMC Medicine 2015;13(1):224.
28. Open Grey. http://www.opengrey.eu/.
29. Social Care Institute for Excellence. https://www.scie-socialcareonline.org.uk/.
30. National Institute of Health & Care Excellence. https://www.nice.org.uk/. Accessed.
31. Bouillon, K, Kivimaki, M, Hamer, M, et al. Measures of frailty in population-based studies: an overview. BMC Geriatr 2013;13:64.
32. Chang, S-F, Lin, P-L. Frail phenotype and mortality prediction: A systematic review and meta-analysis of prospective cohort studies. International Journal of Nursing Studies 2015;52(8):1362-1374.
33. Kojima, G, Iliffe, S, Walters, K. Frailty index as a predictor of mortality: a systematic review and meta-analysis. Age and Ageing 2017;47(2):193-200.
34. Salminen, M, Viljanen, A, Eloranta, S, et al. Frailty and mortality: an 18-year follow-up study among Finnish community-dwelling older people. Aging Clinical and Experimental Research 2019.
35. Kojima, G. Frailty as a predictor of hospitalisation among community-dwelling older people: a systematic review and meta-analysis. Journal of Epidemiology and Community Health 2016;70(7):722-729.
36. Bland, JM, Altman, DG. Validating scales and indexes. BMJ 2002;324(7337):606-607.
37. Onen, NF, Patel, P, Baker, J, et al. Frailty and Pre-Frailty in a Contemporary Cohort of HIV-Infected Adults. J Frailty Aging 2014;3(3):158-165.
38. Onen, NF, Shacham, E, Stamm, KE, et al. Sexual behaviors and results of bacterial sexually transmitted infections testing among frail HIV-infected individuals. J Int Assoc Physicians AIDS Care (Chic) 2010;9(1):30-33.
39. Prince, CS, Noren Hooten, N, Mode, NA, et al. Frailty in middle age is associated with frailty status and race-specific changes to the transcriptome. Aging (Albany NY) 2019;11(15):5518-5534.
40. Rochira, V, Diazzi, C, Santi, D, et al. Low testosterone is associated with poor health status in men with human immunodeficiency virus infection: a retrospective study. Andrology 2015;3(2):298-308.

For peer review only - http://bmjopen.bmj.com/site/about/guidelines.xhtml

41. Vatanen, A, Hou, M, Huang, T, et al. Clinical and biological markers of premature aging after autologous SCT in childhood cancer. Bone Marrow Transplant 2017;52(4):600-605.
42. Wilson, CL, Chemaitilly, W, Jones, KE, et al. Modifiable Factors Associated With Aging Phenotypes Among Adult Survivors of Childhood Acute Lymphoblastic Leukemia. J Clin Oncol 2016;34(21):2509-2515.
43. Adams, P, Ghanem, T, Stachler, R, et al. Frailty as a predictor of morbidity and mortality in inpatient head and neck surgery. JAMA Otolaryngol Head Neck Surg 2013;139(8):783-789.
44. Ahmed, AK, Goodwin, CR, De la Garza-Ramos, R, et al. Predicting Short-Term Outcome After Surgery for Primary Spinal Tumors Based on Patient Frailty. World Neurosurgery 108:393-398.
45. Akgun, KM, Tate, JP, Crothers, K, et al. An adapted frailty-related phenotype and the VACS index as predictors of hospitalization and mortality in HIV-infected and uninfected individuals. J Acquir Immune Defic Syndr 2014;67(4):397-404.
46. Akyar, S, Armenia, SJ, Ratnani, P, et al. The Impact of Frailty on Postoperative Cardiopulmonary Complications in the Emergency General Surgery Population. Surg J (N Y) 2018;4(2):e66-e77.
47. Asemota, AO, Gallia, GL. Impact of frailty on short-term outcomes in patients undergoing transsphenoidal pituitary surgery. J Neurosurg 2019;132(2):360-370.
48. Bagshaw, M, Majumdar, SR, Rolfson, DB, et al. A prospective multicenter cohort study of frailty in younger critically ill patients. Crit Care 2016;20(1):175.
49. Balla, FM, Yheulon, CG, Stetler, JL, et al. Ventral hernia repair outcomes predicted by a 5-item modified frailty index using NSQIP variables. Hernia 2019;23(5):891-898.
50. Banaszek, D, Inglis, T, Marion, TE, et al. Effect of Frailty on Outcome after Traumatic Spinal Cord Injury. J Neurotrauma 2020;37(6):839-845.
51. Bao, Y, Dalrymple, L, Chertow, GM, et al. Frailty, dialysis initiation, and mortality in end-stage renal disease. Arch Intern Med 2012;172(14):1071-1077.
52. Brothers, TD, Kirkland, S, Theou, O, et al. Predictors of transitions in frailty severity and mortality among people aging with HIV. PLoS One 2017;12(10):e0185352.
53. Chao, CT, Wang, J, Chien, KL, et al. Both pre-frailty and frailty increase healthcare utilization and adverse health outcomes in patients with type 2 diabetes mellitus. Cardiovasc Diabetol 2018;17(1):130.
54. Chode, S, Malmstrom, TK, Miller, DK, et al. Frailty, Diabetes, and Mortality in Middle-Aged African Americans. J Nutr Health Aging 2016;20(8):854-859.
55. Chu, NM, Deng, A, Ying, H, et al. Dynamic Frailty Before Kidney Transplantation: Time of Measurement Matters. Transplantation 2019;103(8):1700-1704.
56. Chung, CJ, Wu, C, Jones, M, et al. Reduced handgrip strength as a marker of frailty predicts clinical outcomes in patients with heart failure undergoing ventricular assist device placement. J Card Fail 2014;20(5):310-315.
57. Courtwright, AM, Zaleski, D, Gardo, L, et al. Causes, Preventability, and Cost of Unplanned Rehospitalizations Within 30 Days of Discharge After Lung Transplantation. Transplantation 2018;102(5):838-844.
58. Delgado, C, Grimes, BA, Glidden, DV, et al. Association of Frailty based on self-reported physical function with directly measured kidney function and mortality. BMC Nephrol 2015;16:203.
59. Erlandson, KM, Allshouse, AA, Jankowski, CM, et al. Comparison of functional status instruments in HIV-infected adults on effective antiretroviral therapy. HIV Clin Trials 2012;13(6):324-334.
60. Fang, ZB, Hu, FY, Arya, S, et al. Preoperative frailty is predictive of complications after major lower extremity amputation. J Vasc Surg 2017;65(3):804-811.

For peer review only - http://bmjopen.bmj.com/site/about/guidelines.xhtml

61. Fernando, SM, McIsaac, DI, Rochwerg, B, et al. Frailty and invasive mechanical ventilation: association with outcomes, extubation failure, and tracheostomy. Intensive Care Med 2019;45(12):1742-1752.
62. Fitzpatrick, J, Sozio, SM, Jaar, BG, et al. Frailty, body composition and the risk of mortality in incident hemodialysis patients: the Predictors of Arrhythmic and Cardiovascular Risk in End Stage Renal Disease study. Nephrol Dial Transplant 2019;34(2):346-354.
63. George, EM, Burke, WM, Hou, JY, et al. Measurement and validation of frailty as a predictor of outcomes in women undergoing major gynaecological surgery. BJOG 2016;123(3):455-461.
64. Griffin, FR, Mode, NA, Ejiogu, N, et al. Frailty in a racially and socioeconomically diverse sample of middle-aged Americans in Baltimore. PLoS One 2018;13(4):e0195637.
65. Guaraldi, G, Brothers, TD, Zona, S, et al. A frailty index predicts survival and incident multimorbidity independent of markers of HIV disease severity. AIDS 2015;29(13):1633-1641.
66. Guaraldi, G, Dolci, G, Zona, S, et al. A frailty index predicts post-liver transplant morbidity and mortality in HIV-positive patients. AIDS Res Ther 2017;14(1):37.
67. Gustafson, DR, Shi, Q, Holman, S, et al. Predicting death over 8 years in a prospective cohort of HIV-infected women: the Women's Interagency HIV Study. BMJ Open 2017;7(6):e013993.
68. Haugen, CE, McAdams-DeMarco, M, Verna, EC, et al. Association Between Liver Transplant Wait-list Mortality and Frailty Based on Body Mass Index. JAMA Surg 2019;154(12):1103-1109.
69. Heberton, GA, Nassif, M, Bierhals, A, et al. Usefulness of Psoas Muscle Area Determined by Computed Tomography to Predict Mortality or Prolonged Length of Hospital Stay in Patients Undergoing Left Ventricular Assist Device Implantation. Am J Cardiol 2016;118(9):1363-1367.
70. Hewitt, J, Carter, B, McCarthy, K, et al. Frailty predicts mortality in all emergency surgical admissions regardless of age. An observational study. Age Ageing 2019;48(3):388-394.
71. Hope, AA, Hsieh, SJ, Petti, A, et al. Assessing the Usefulness and Validity of Frailty Markers in Critically Ill Adults. Ann Am Thorac Soc 2017;14(6):952-959.
72. Hyde, Z, Flicker, L, Smith, K, et al. Prevalence and incidence of frailty in Aboriginal Australians, and associations with mortality and disability. Maturitas 2016;87:89-94.
73. Jha, SR, Hannu, MK, Chang, S, et al. The Prevalence and Prognostic Significance of Frailty in Patients With Advanced Heart Failure Referred for Heart Transplantation. Transplantation 2016;100(2):429-436.
74. Jha, SR, Hannu, MK, Gore, K, et al. Cognitive impairment improves the predictive validity of physical frailty for mortality in patients with advanced heart failure referred for heart transplantation. J Heart Lung Transplant 2016;35(9):1092-1100.
75. Johansen, KL, Dalrymple, LS, Delgado, C, et al. Comparison of self-report-based and physical performance-based frailty definitions among patients receiving maintenance hemodialysis. Am J Kidney Dis 2014;64(4):600-607.
76. Johansen, KL, Dalrymple, LS, Glidden, D, et al. Association of Performance-Based and Self-Reported Function-Based Definitions of Frailty with Mortality among Patients Receiving Hemodialysis. Clin J Am Soc Nephrol 2016;11(4):626-632.
77. Johansen, KL, Delgado, C, Kaysen, GA, et al. Frailty Among Patients Receiving Hemodialysis: Evolution of Components and Associations With Mortality. J Gerontol A Biol Sci Med Sci 2019;74(3):380-386.
78. Joseph, SM, Manghelli, JL, Vader, JM, et al. Prospective Assessment of Frailty Using the Fried Criteria in Patients Undergoing Left Ventricular Assist Device Therapy. Am J Cardiol 2017;120(8):1349-1354.
79. Kahlon, S, Pederson, J, Majumdar, SR, et al. Association between frailty and 30-day outcomes after discharge from hospital. CMAJ 2015;187(11):799-804.

For peer review only - http://bmjopen.bmj.com/site/about/guidelines.xhtml

80. Kane, AE, Gregson, E, Theou, O, et al. The association between frailty, the metabolic syndrome, and mortality over the lifespan. Geroscience 2017;39(2):221-229.
81. Kelly, SG, Wu, K, Tassiopoulos, K, et al. Frailty Is an Independent Risk Factor for Mortality, Cardiovascular Disease, Bone Disease, and Diabetes Among Aging Adults With Human Immunodeficiency Virus. Clin Infect Dis 2019;69(8):1370-1376.
82. Kelm, DJ, Bonnes, SL, Jensen, MD, et al. Pre-transplant wasting (as measured by muscle index) is a novel prognostic indicator in lung transplantation. Clin Transplant 2016;30(3):247-255.
83. Konstantinidis, IT, Chouliaras, K, Levine, EA, et al. Frailty Correlates with Postoperative Mortality and Major Morbidity After Cytoreductive Surgery with Hyperthermic Intraperitoneal Chemotherapy. Ann Surg Oncol 2017;24(13):3825-3830.
84. Lai, JC, Covinsky, KE, Dodge, JL, et al. Development of a novel frailty index to predict mortality in patients with end-stage liver disease. Hepatology 2017;66(2):564-574.
85. Lai, JC, Covinsky, KE, McCulloch, CE, et al. The Liver Frailty Index Improves Mortality Prediction of the Subjective Clinician Assessment in Patients With Cirrhosis. Am J Gastroenterol 2018;113(2):235-242.
86. Lai, JC, Dodge, JL, Sen, S, et al. Functional decline in patients with cirrhosis awaiting liver transplantation: Results from the functional assessment in liver transplantation (FrAILT) study. Hepatology 2016;63(2):574-580.
87. Lai, JC, Rahimi, RS, Verna, EC, et al. Frailty Associated With Waitlist Mortality Independent of Ascites and Hepatic Encephalopathy in a Multicenter Study. Gastroenterology 2019;156(6):1675-1682.
88. Lee, SY, Yang, DH, Hwang, E, et al. The Prevalence, Association, and Clinical Outcomes of Frailty in Maintenance Dialysis Patients. J Ren Nutr 2017;27(2):106-112.
89. Legge, A, Kirkland, S, Rockwood, K, et al. Evaluating the Properties of a Frailty Index and Its Association With Mortality Risk Among Patients With Systemic Lupus Erythematosus. Arthritis Rheumatol 2019;71(8):1297-1307.
90. Li, X, Ploner, A, Karlsson, IK, et al. The frailty index is a predictor of cause-specific mortality independent of familial effects from midlife onwards: a large cohort study. BMC Med 2019;17(1):94.
91. Louwers, L, Schnickel, G, Rubinfeld, I. Use of a simplified frailty index to predict Clavien 4 complications and mortality after hepatectomy: analysis of the National Surgical Quality Improvement Project database. American Journal of Surgery 2016;211(6):1071-1076.
92. Makhani, SS, Kim, FY, Liu, Y, et al. Cognitive Impairment and Overall Survival in Frail Surgical Patients. J Am Coll Surg 2017;225(5):590-600 e591.
93. Malmstrom, TK, Miller, DK, Morley, JE. A comparison of four frailty models. J Am Geriatr Soc 2014;62(4):721-726.
94. Martin, L, McKenzie, K, Ouellette-Kuntz, H. Once frail, always frail? Frailty transitions in home care users with intellectual and developmental disabilities. Geriatr Gerontol Int 2018;18(4):547-553.
95. Mascarella, MA, Milad, D, Richardson, K, et al. Preoperative Risk Index Among Patients Undergoing Thyroid or Parathyroid Surgery. Jama Otolaryngol 2020;146(1):7-12.
96. McAdams-DeMarco, MA, Law, A, King, E, et al. Frailty and mortality in kidney transplant recipients. Am J Transplant 2015;15(1):149-154.
97. McAdams-DeMarco, MA, Law, A, Salter, ML, et al. Frailty and early hospital readmission after kidney transplantation. Am J Transplant 2013;13(8):2091-2095.
98. McAdams-DeMarco, MA, Ying, H, Olorundare, I, et al. Individual Frailty Components and Mortality in Kidney Transplant Recipients. Transplantation 2017;101(9):2126-2132.
99. McAdams-DeMarco, MA, Ying, H, Thomas, AG, et al. Frailty, Inflammatory Markers, and Waitlist Mortality Among Patients With End-stage Renal Disease in a Prospective Cohort Study. Transplantation 2018;102(10):1740-1746.

For peer review only

100. McAlister, FA, Youngson, E, Eurich, DT. Treated glycosylated hemoglobin levels in individuals with diabetes mellitus vary little by health status: A retrospective cohort study. Medicine (Baltimore) 2016;95(24):e3894.
101. McChesney, SL, Canter, DJ, Monlezun, DJ, et al. Modified Frailty Index Predicts Postoperative Outcomes in Patients Undergoing Radical Pelvic Surgery. Am Surg 2020;86(2):95-103.
102. McDonagh, J, Salamonson, Y, Ferguson, C, et al. Evaluating the convergent and discriminant validity of three versions of the frailty phenotype in heart failure: results from the FRAME-HF study. Eur J Cardiovasc Nurs 2020;19(1):55-63.
103. McIntyre, M, Gandhi, C, Dragonette, J, et al. Increasing Frailty Predicts Worse Outcomes and Increased Complications After Angiogram-Negative Subarachnoid Hemorrhages. World Neurosurg 2020;134:e181-e188.
104. McIntyre, MK, Gandhi, C, Long, A, et al. Age predicts outcomes better than frailty following aneurysmal subarachnoid hemorrhage: A retrospective cohort analysis. Clin Neurol Neurosurg 2019;187:105558.
105. McKenzie, K, Ouellette-Kuntz, H, Martin, L. Frailty as a Predictor of Institutionalization Among Adults With Intellectual and Developmental Disabilities. Intellect Dev Disabil 2016;54(2):123-135.
106. Moayedi, Y, Duero Posada, JG, Foroutan, F, et al. The prognostic significance of frailty compared to peak oxygen consumption and B-type natriuretic peptide in patients with advanced heart failure. Clin Transplant 2018;32(1):01.
107. Montgomery, CL, Zuege, DJ, Rolfson, DB, et al. Implementation of population-level screening for frailty among patients admitted to adult intensive care in Alberta, Canada. Can J Anaesth 2019;66(11):1310-1319.
108. Montgomery, E, Macdonald, PS, Newton, PJ, et al. Frailty as a Predictor of Mortality in Patients With Interstitial Lung Disease Referred for Lung Transplantation. Transplantation 2020;104(4):864-872.
109. Morley, JE, Malmstrom, TK, Miller, DK. A simple frailty questionnaire (FRAIL) predicts outcomes in middle aged African Americans. J Nutr Health Aging 2012;16(7):601-608.
110. Mosquera, C, Bermudez, JM, Evans, JL, et al. Frailty Predicts Failure to Rescue after Thoracoabdominal Operation. J Am Coll Surg 2018;226(6):978-986.
111. Nastasi, AJ, McAdams-DeMarco, MA, Schrack, J, et al. Pre-Kidney Transplant Lower Extremity Impairment and Post-Kidney Transplant Mortality. Am J Transplant 2018;18(1):189-196.
112. Ness, KK, Krull, KR, Jones, KE, et al. Physiologic frailty as a sign of accelerated aging among adult survivors of childhood cancer: a report from the St Jude Lifetime cohort study. J Clin Oncol 2013;31(36):4496-4503.
113. Ney, M, Tangri, N, Dobbs, B, et al. Predicting Hepatic Encephalopathy-Related Hospitalizations Using a Composite Assessment of Cognitive Impairment and Frailty in 355 Patients With Cirrhosis. Am J Gastroenterol 2018;113(10):1506-1515.
114. Pamukcuoglu, M, Bhatia, S, Weisdorf, DJ, et al. Hematopoietic Cell Transplant-Related Toxicities and Mortality in Frail Recipients. Biol Blood Marrow Transplant 2019;25(12):2454-2460.
115. Pena, FG, Theou, O, Wallace, L, et al. Comparison of alternate scoring of variables on the performance of the frailty index. BMC Geriatr 2014;14:25.
116. Ravindrarajah, R, Lee, DM, Pye, SR, et al. The ability of three different models of frailty to predict all-cause mortality: results from the European Male Aging Study (EMAS). Arch Gerontol Geriatr 2013;57(3):360-368.
117. Rozenberg, D, Mathur, S, Wickerson, L, et al. Frailty and clinical benefits with lung transplantation. J Heart Lung Transplant 2018;37(10):1245-1253.

For peer review only - http://bmjopen.bmj.com/site/about/guidelines.xhtml

118. Shin, JI, Kothari, P, Phan, K, et al. Frailty Index as a Predictor of Adverse Postoperative Outcomes in Patients Undergoing Cervical Spinal Fusion. Spine (Phila Pa 1976) 2017;42(5):304-310.
119. Singer, JP, Diamond, JM, Anderson, MR, et al. Frailty phenotypes and mortality after lung transplantation: A prospective cohort study. Am J Transplant 2018;18(8):1995-2004.
120. Singer, JP, Diamond, JM, Gries, CJ, et al. Frailty Phenotypes, Disability, and Outcomes in Adult Candidates for Lung Transplantation. Am J Respir Crit Care Med 2015;192(11):1325-1334.
121. Subramaniam, S, Aalberg, JJ, Soriano, RP, et al. New 5-Factor Modified Frailty Index Using American College of Surgeons NSQIP Data. J Am Coll Surg 2018;226(2):173-181 e178.
122. Susanto, M, Hubbard, RE, Gardiner, PA. Validity and Responsiveness of the FRAIL Scale in Middle-Aged Women. J Am Med Dir Assoc 2018;19(1):65-69.
123. Traven, SA, Horn, RW, Reeves, RA, et al. The 5-Factor Modified Frailty Index Predicts Complications, Hospital Admission, and Mortality Following Arthroscopic Rotator Cuff Repair. Arthroscopy 2020;36(2):383-388.
124. Verheij, E, Kirk, GD, Wit, FW. Frailty is associated with mortality and incident comorbidity among middle-aged HIV-positive and HIV-negative participants. Care Management 2020;26(1):22-22.
125. Myers, V, Drory, Y, Gerber, Y, et al. Clinical relevance of frailty trajectory post myocardial infarction. Eur J Prev Cardiol 2014;21(6):758-766.
126. Rockwood, K, Howlett, SE, MacKnight, C, et al. Prevalence, attributes, and outcomes of fitness and frailty in community-dwelling older adults: report from the Canadian study of health and aging. The journals of gerontology Series A, Biological sciences and medical sciences 2004;59(12):1310-1317.
127. Blodgett, JM, Theou, O, Mitnitski, A, et al. Associations between a laboratory frailty index and adverse health outcomes across age and sex. Aging Med (Milton) 2019;2(1):11-17.
128. Hanlon, P, Nicholl, BI, Jani, BD, et al. Frailty and pre-frailty in middle-aged and older adults and its association with multimorbidity and mortality: a prospective analysis of 493 737 UK Biobank participants. The Lancet Public Health 2018;3(7):e323-e332.
129. Tapper, EB, Finkelstein, D, Mittleman, MA, et al. Standard assessments of frailty are validated predictors of mortality in hospitalized patients with cirrhosis. Hepatology 2015;62(2):584-590.
130. Dolgin, NH, Martins, PN, Movahedi, B, et al. Functional status predicts postoperative mortality after liver transplantation. Clin Transplant 2016;30(11):1403-1410.
131. Zdradzinski, MJ, Phelan, MP, Mace, SE. Impact of Frailty and Sociodemographic Factors on Hospital Admission From an Emergency Department Observation Unit. Am J Med Qual 2017;32(3):299-306.
132. At, J, Bryce, R, Prina, M, et al. Frailty and the prediction of dependence and mortality in low- and middle-income countries: a 10/66 population-based cohort study. BMC Medicine 2015;13(1):138.
133. Bagshaw, SM, Stelfox, HT, McDermid, RC, et al. Association between frailty and short- and long-term outcomes among critically ill patients: a multicentre prospective cohort study. Canadian Medical Association Journal 2014;186(2):E95-E102.
134. Boyd, CM, Xue, Q-L, Simpson, CF, et al. Frailty, hospitalization, and progression of disability in a cohort of disabled older women. The American Journal of Medicine 2005;118(11):1225-1231.

For peer review only - http://bmjopen.bmj.com/site/about/guidelines.xhtml

Figure 1. PRISMA Flowchart

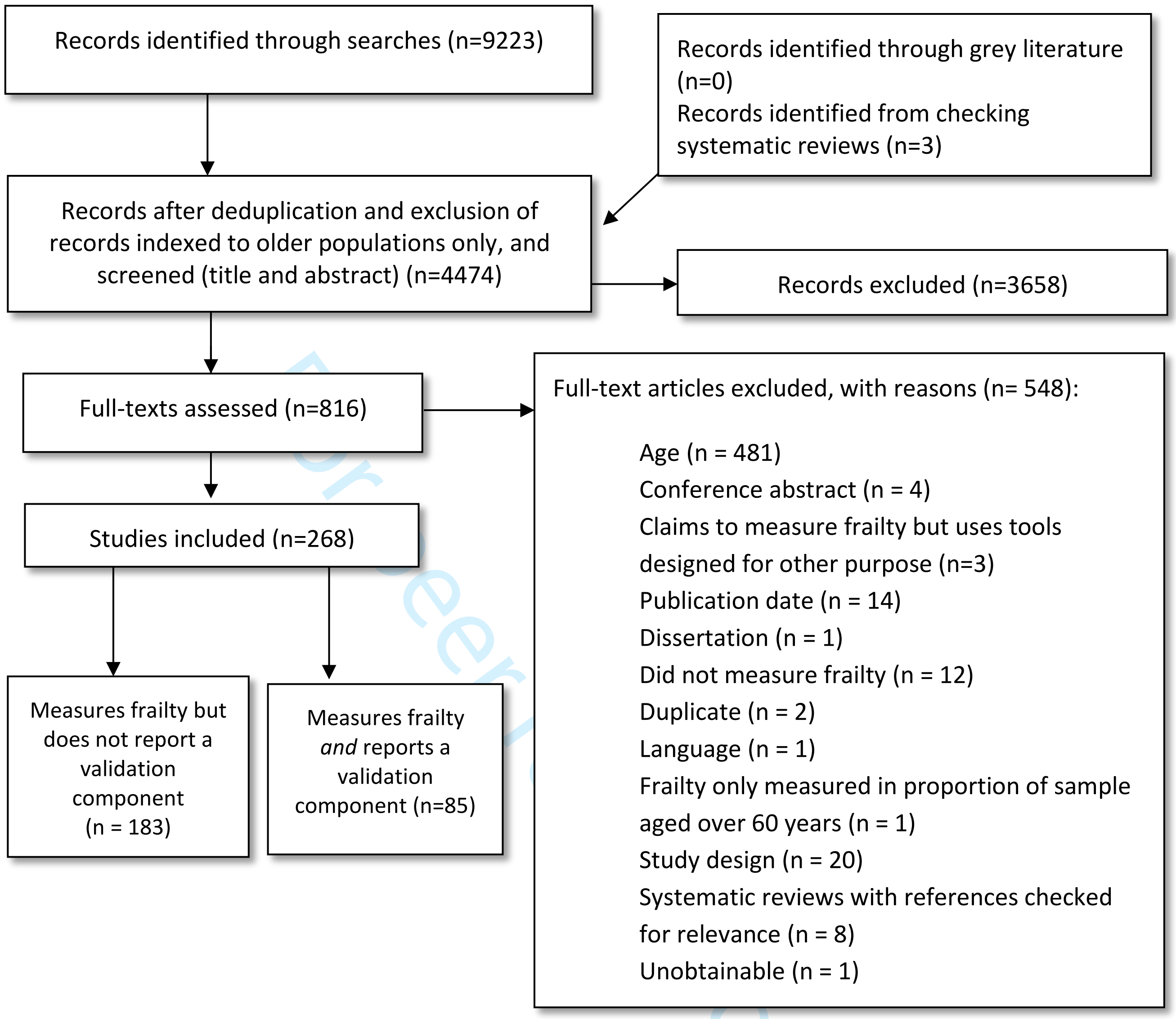

Figure 2. Proportion (%) of studies with different average sample ages [a]

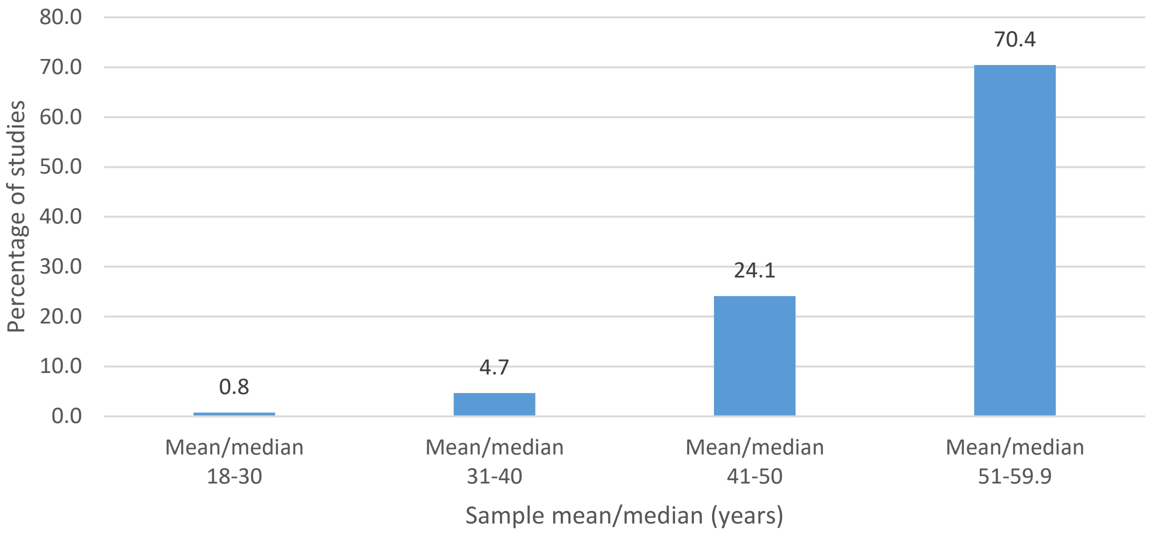

[a]*Calculated from studies where a mean or median sample age was reported (n=257)*

peer review only

Supplementary materials

Searches strategy applied to Medline

Searched 17th March 2020; Database(s): **Ovid MEDLINE(R)** 1946 to March Week 1 2020

Key:

/	thesaurus heading
.ti,ab	terms must appear in the title or abstract fields
adj8	the terms either side must be within eight words of each other
*	unlimited truncation
?	single letter truncation

#	Searches	Results
1	Frailty/cl, di, ep [Classification, Diagnosis, Epidemiology]	1007
2	Frailty/	2162
3	"Sensitivity and Specificity"/	343505
4	"Predictive Value of Tests"/	199066
5	ROC Curve/	56249
6	"Diagnostic Techniques and Procedures"/	3243
7	exp Physical Examination/	1349129
8	Symptom Assessment/	4429
9	Psychometrics/	73737
10	Prevalence/	284108
11	or/3-10	2159330
12	2 and 11	631
13	((frail* or prefrail* or pre frail*) adj8 (measur* or assess* or estimat* or define? or definition or classif* or test* or identif* or observ* or screen* or review* or indicat* or criteri* or sign? or symptom? or prevalence*)).ti,ab.	5778
14	((frail* or prefrail* or pre frail*) adj8 (instrument? or tool? or framework* or index* or score? or scale? or psychometric or reliable or reliability or valid* or responsive*)).ti,ab.	2733
15	((frail* or prefrail* or pre frail*) adj8 (associat* or predict*)).ti,ab.	3371
16	or/13-15	7248
17	1 or 12 or 16	7460
18	exp Aged/	3062714
19	adult/ or middle aged/ or young adult/	6433186
20	18 not (18 and 19)	645469
21	17 not 20	2842
22	limit 21 to (english language and humans and yr="2000 -Current")	2511

For peer review only

Supplementary materials table S1. Framework to determine evidence of measure validity (predictive and/or criterion)

Green (evidence of validity)	• Prediction of mortality and/or hospital admissions was statistically significant<br>• Associations and agreement between measures (criterion validity) were judged to be good or strong by study authors
Amber (unclear or inconsistent evidence of validity)	• Evidence of validity was mixed because:<br>o The frailty measure was tested against both outcomes (mortality and hospital admission) but observed a significant association for only one outcome<br>o Prediction was statistically significant for only some sub-groups (e.g. age groups)<br>o Evidence of either, but not both, predictive or criterion validity was demonstrated<br>o Evidence of predictive validity varied depending on when (e.g. 30 day) and where (e.g. hospital or ICU) mortality was measured, the level of frailty tested (e.g. moderate or severe), or how the frailty score was used (e.g. as continuous or binary)<br>o Where individual components of the frailty measure were tested and only some, not all, demonstrated predictive validity<br>• Only differences in mortality rates between frail and not frail were reported, with no test of prediction<br>• Unclear reporting in the paper made it difficult to determine whether the measure was intended to capture frailty or a different concept<br>• Associations and agreement between measures (criterion validity) were judged to be fair or modest by study authors
Red (no evidence of validity)	• Prediction of mortality and/or hospital admissions was not statistically significant<br>• Data were not reported in the publication to verify a claim that the frailty measure predicted mortality and/or hospital admissions<br>• Associations and agreement between measures (criterion validity) were judged to be poor or weak by study authors

For peer review only

For peer review only

Supplementary materials table S2. Overview of all included studies (n=268) [1-227228-268]

Shaded rows are studies that report a validation component and which were used in the synthesis of measure validity

Study author and date	Country	Mean (SD) / Median (IQR) age<br><br>Where overall sample age is not reported, age details of sub-groups are provided instead.	Is the sample aged entirely under 60 years, or includes those aged under and over 60 years?	Long-term condition group (specify which group) or general population	Measure/approach used
Abdel-Kader 2018	US	Median: 57.0 (IQR not reported)	< and > 60	Acute Kidney Injury	Clinical Frailty Scale
Adams 2013	US	Mean: 54.7 (17.2), range 16-90	< and > 60	Head and neck surgery	mFI (11)
Ahmed 2017	US	Median: 47 (28 – 61)	< and > 60	Spinal tumor surgery	Spinal tumor frailty index (9)
Aizpuru 2018	US	LOS<6 days<br>Mean: 36.9 (19.5)<br>LOS>6 days<br>Mean: 42.1 (19.1)	< and > 60	Femur fractures	mFI (11)
Akgun 2014	US	Mean: 47.6 (8.5)	< and > 60	HIV	Adapted Fried Phenotype (aFRP), using 4/5 criteria (physical shrinking, exhaustion, slowness, and decreased physical activity).
Akgun 2016	US	89.7% of HIV infected <60<br>84.9% of non-infected <60	< and > 60	HIV	Adapted Fried phenotype (aFRP), using 4/5 criteria (physical shrinking, exhaustion, slowness, and decreased physical activity).
Akyar 2018	US	71.2% <60 years	< and > 60	Emergency general surgery	mFI (11)
Alshaikh 2016	US	MG Patients<br>Mean: 45.9 (17.2)<br>Non-MG patients<br>Mean: 53.3 (15.1)	< and > 60	Thymectomy	Frailty Index (no further detail reported)
Althoff 2014	US	Frail<br>Median: 53.8 (47.6-61.3)<br>Non-Frail<br>Median: 50.5 (44.6-56.9)	< and > 60	HIV	Phenotype

Study author and date	Country	Mean (SD) / Median (IQR) age<br><br>Where overall sample age is not reported, age details of sub-groups are provided instead.	Is the sample aged entirely under 60 years, or includes those aged under and over 60 years?	Long-term condition group (specify which group) or general population	Measure/approach used
Anderson 2014	US	Mean: 59.1 (19.7)	< and > 60	Acute acalculous cholecystitis and severe sepsis and shock	NOT REPORTED
Andrews 2017	US	Mean: 58.0 (10.8)	< and > 60	Rheumatoid Arthritis	Phenotype
Andrews 2019	US	Mean: 58.0 (10.8)	< and > 60	Rheumatoid Arthritis	Phenotype
Asemota 2019	US	Frail<br>Mean: 57.14 (16.96)<br>Non-frail<br>Mean: 51.91 (15.88)	< and > 60	Transsphenoidal pituitary surgery	Johns Hopkins Adjusted Clinical Groups (ACG) frailty-defining diagnoses indicator
Ayesta 2018	Spain	Study protocol only - no data collected		Heart transplantation	Phenotype<br>Short Physical Performance Battery
Bagshaw 2016	Canada	Mean: 58.5 (4.1)	< and > 60	Critically ill, ICU	Clinical Frailty Scale
Balla 2019	US	Mean: 55.9 (SD not reported)	< and > 60	Ventral Hernia Repair	mFI (5)
Banasek 2020	Canada	Mean: 50.3 (19.8)	< and > 60	Traumatic Spinal Cord Injury	mFI (11)
Bao 2012	US	Mean: 59.6 (14.2)	< and > 60	End stage renal disease	Phenotype, excluding weight loss as this variable was not available in the data used.
Bello 2018	US	Median: 51 (46–57), range 40–85	< and > 60	9/11/2001 responders exposed to toxic pollutants	Physiological frailty index (FI-Lab)
Bindawas 2018	UK	Mean: 59.9 (68.8)	< and > 60	Knee pain	Phenotype
Blanco 2019	Spain	Robust Mean: 47.7 (8.6)<br>Pre-Frail Mean: 49.6 (11.6)<br>Frail Mean: 59.5 (12.7)	< and > 60	HIV	Phenotype
Blodgett 2017	Canada	Mean: 49.4 (19.0)	< and > 60	General	FI-Lab<br>FI-Self Report
Blodgett 2019	Canada	Mean age not reported. 66% <60, lowest 20	< and > 60	General	Fi-Lab
Bouillion 2013	UK	Mean: 55.2 (5.9)	< and > 60	General	Phenotype
Bouillion 2013	UK	Mean: 55.0 (5.9), range 45-69	< and > 60	Diabetes	Phenotype

For peer review only - http://bmjopen.bmj.com/site/about/guidelines.xhtml

Study author and date	Country	Mean (SD) / Median (IQR) age<br><br>Where overall sample age is not reported, age details of sub-groups are provided instead.	Is the sample aged entirely under 60 years, or includes those aged under and over 60 years?	Long-term condition group (specify which group) or general population	Measure/approach used
Bregigeon 2017	France	Males Median: 56.0 (51.0-64.0)<br>Females Median: 53.0 (50.0-56.0)	< and > 60	HIV	Phenotype
Brothers 2017	Canada	Mean: 46.8 (7.1)	18+, though not clear if includes those aged over 60	HIV	Frailty Index (31)
Buganza-Toria 2019	Canada	Mean: 55.0 (10.0)	< and > 60	Cirrhosis	Clinical Frailty Scale<br>Phenotype
Buitrago 2018	US	Median: 58 (51 – 66)	< and > 60	Tracheobronchoplasty	Frailty index (26)
Chao 2018	Taiwan	Mean: 56.4 (13.8)	< and > 60	Type 2 Diabetes	FRAIL scale
Chen 2018	US	Mean: 58.8 (12.3)	< and > 60	Combined Colorectal and Liver Resections (Surgery)	mFI (5)
Chiang 2019	US	Mean: 56.16 (14.2)	< and > 60	Hemodialysis	Phenotype
Chode 2016	US	With diabetes<br>Mean: 57.43 (4.4)<br>Without diabetes<br>Mean: 55.97 (4.4)	< and > 60	Diabetes	FRAIL Scale<br>Frailty Index (25)<br>Study of Osteoporotic Fractures (SOF) Scale<br>Cardiovascular Health Study (CHS) Scale
Chu 2019	US	Mean: 51.7 (14.0), range 18–85	< and > 60	End stage renal disease	Phenotype
Chu 2019b	US	Mean: 52.0 (SD not reported)	< and > 60	Kidney Transplant	Phenotype
Chung 2014	US	Mean: 59.0 (2.0)	< and > 60	Heart failure	Hand grip as a proxy for frailty
Cook 2017	Italy, Sweden, Belgium, UK, Spain, Poland, Hungary, Estonia	Mean: 59.9 (11.0)	< and > 60	General	Adapted Fried Phenotype<br>Frailty Index (39)
Courtwright 2018	US	Mean: 53.5 (14.1)	< and > 60	Lung transplant	Short Physical Performance Battery

Study author and date	Country	Mean (SD) / Median (IQR) age<br><br>Where overall sample age is not reported, age details of sub-groups are provided instead.	Is the sample aged entirely under 60 years, or includes those aged under and over 60 years?	Long-term condition group (specify which group) or general population	Measure/approach used
Courtwright 2019	US	Mean: 57.0 (44-64)	< and > 60	Lung transplant	Short Physical Performance Battery
Cron 2016	US	Mean: 54.9 (9.9)	< and > 60	End stage liver disease	Phenotype
de Haas 2018	Netherlands	Low Frailty Mean: 55.9 (5.2)<br>High Frailty Mean: 58.1 (7.1)	< and > 60	General	Frailty Index (38)
Delgado 2013	US	Mean: 55.0 (13.0)	< and > 60	CKD, Hemodialysis	Performance-based Frailty (PFP), using gait speed and time required to perform 5 sit-to-stand manoeuvers.<br><br>Function-based frailty (FBF), using physical functioning, exhaustion, and physical activity.
Delgado 2015	US	Median: 52 (42-61)	< and > 60	Chronic Kidney Disease	Adapted Fried Phenotype
Denfield 2017	US	Mean: 57.4 (9.7)	< and > 60	Heart failure	Phenotype
Derck 2015	US	Frail<br>Mean: 55.9 (9.3)<br>Not frail<br>Mean: 54.3 (10.3)	< and > 60	Liver transplant	Phenotype
Dolgin 2019	US	Mean: 55.4 (SD not reported)	< and > 60 years	Liver transplant	Sarcopenia as an 'objective measure of frailty' but no further detail given.
Dos Santos 2020	Brazil	Non-frail<br>Mean: 48.0 (12.0)<br>Frail<br>Mean: 46.0 (13.0)	18+, though not clear if includes those aged over 60	Kidney Transplant	Phenotype
Dumond 2016	US	Median: 48 (22–73)	< and > 60	HIV	Phenotype
Erlandson 2012	US	Median: 50.8 (47.7–55.7)	< and > 60	HIV	Phenotype<br>Short Physical Performance Battery<br>400 metre walking test
Erlandson 2017	US	85% <60	< and > 60	HIV	Phenotype

Study author and date	Country	Mean (SD) / Median (IQR) age<br><br>Where overall sample age is not reported, age details of sub-groups are provided instead.	Is the sample aged entirely under 60 years, or includes those aged under and over 60 years?	Long-term condition group (specify which group) or general population	Measure/approach used
Erlandson 2017b	US	Uninfected men<br>Median: 48.5 (41.5-53.1)<br>Infected + frail<br>Median: 49.1 (41.4-53.9)<br>Infected non-frail<br>Median: 47.7 (40.4-53.0)	< and > 60	HIV	4/5 Phenotype criteria
Erlandson 2019	US	Median: 51 (IQR not reported)	< and > 60	HIV	Phenotype
Erlandson 2020	US	Mean: 51.0 (7.5)	Not clear if sample includes those aged over 60	HIV	Phenotype
Escota 2015	US	Median: 48.0 (42–54)	< and > 60	HIV	Phenotype
Fang 2017	US	Mean 59.0 (15.0)	< and > 60	Lower extremity amputation	mFI (11)
Fernando 2019	Canada	Not Frail Mean: 57.6 (18.1)<br>Frail Mean: 69.2 (12.2)	< and > 60	Ventilation, ICU	Clinical Frailty Scale
Fitzpatrick 2019	Canada	Mean: 54.9 (13.1)	< and > 60	Hemodialysis, End stage renal disease	Phenotype
Franconi 2018	Italy	Mean: 53.1 (8.0)	< and > 60	HIV	Frailty Index (71)
Garonzik-Wang 2012	US	Mean: 53.0 (14.0)	< and > 60	End stage renal disease	Phenotype
Gauraldi 2019d	Italy	Mean: 48.0 (8.2)	< and > 60	HIV	Frailty Index (37)
George 2016	US	70.8% <60	< and > 60	Hysterectomy	mFI (11)
Gordon 2020	Australia	Mean: 59.9 (10.6) 40-75	< and > 60	General	Phenotype
Griffin 2018	US	Mean: 50.2 (SD not reported)<br>Range: 35–64	< and > 60	General	FRAIL Scale

Study author and date	Country	Mean (SD) / Median (IQR) age<br><br>Where overall sample age is not reported, age details of sub-groups are provided instead.	Is the sample aged entirely under 60 years, or includes those aged under and over 60 years?	Long-term condition group (specify which group) or general population	Measure/approach used
Groß 2016	Germany	Range: 18-60	< and > 60	HIV	Item C from EQ-5D (problems with general activities, as Yes/No response)
Guaraldi 2015	Italy	Mean: 46.0 (8.0)	< and > 60	HIV	Frailty Index (37)
Guaraldi 2017	Italy	Median: 51.2 (45.91–54.35)	< and > 60	HIV + liver transplant	Frailty Index (30)
Guaraldi 2017b	Italy	Mean: 53.9 (6.9)	< and > 60	HIV	Phenotype<br>Frailty Index (37)
Guaraldi 2019	Italy	Median: 49 (45–54)	< and > 60	HIV	Frailty Index (37)
Guaraldi 2019a	Italy	Median: 49 (45–54)	< and > 60	HIV	Frailty Index (37)
Guaraldi 2019c	Italy	Mean: 53.11 (7.98)	< and > 60	HIV	Frailty Index (37)
Gustafson 2016	US	Mean: 39.0 (SD not reported)	< and > 60	HIV	Phenotype
Gustafson 2017	US	Mean: 42.6 (8.8)	< and > 60	HIV	Phenotype
Hadenfeldt 2017	US	Mean: 50.0 (6.0)	< and > 60	General	Comprehensive Frailty Assessment Instrument (CFAI). Study team added 7 items to the measure.
Haider 2019	Austria	Mean: 50.9 (9.7)	< and > 60	Rheumatoid Arthritis	Frailty Instrument for Primary Care for the Survey of Health Ageing and Retirement across Europe (SHARE), based on the Phenotype criteria
Hampton 2019	US	Mean: 59.0 (range: 49 - 69)	< and > 60	Major gastrointestinal surgery	mFI (5)
Harland 2020	US	Non frail<br>Mean: 50.1, range: 19 - 78<br>Moderately frail<br>Mean: 56.1, range: 21 - 82<br>Frail<br>Mean: 61.3, range: 40 - 73	< and > 60	Brain timour	Hopkins Frailty Score (HFS)
Haugen 2018	US	Mean: 50.3 (13.7)	< and > 60	Kidney transplant	Phenotype
Haugen 2019	US	Mean: 54.0 (13.0)	< and > 60	End stage renal disease	Phenotype

For peer review only

Study author and date	Country	Mean (SD) / Median (IQR) age<br><br>Where overall sample age is not reported, age details of sub-groups are provided instead.	Is the sample aged entirely under 60 years, or includes those aged under and over 60 years?	Long-term condition group (specify which group) or general population	Measure/approach used
Haugen 2019b	US	Mean: 55.0 (10.0)	< and > 60	Liver transplant	Liver Frailty Index
Haugen 2020	US	Not frail<br>Median: 52.0 (42.0-60.0)<br>Pre frail<br>Median: 54.0 (44.0-62.0)<br>Frail<br>Median: 55.0 (45.0-63.0)	< and > 60	Kidney Transplant	Phenotype
Hayak 2020	US	Mean: 24.3 (8.9)	Not clear if sample includes those aged over 60	Childgood cancer survivors	Phenotype
Heberton 2016	USA	Sarcopenic (yes): 53.0 (14.0)<br>Sarcopenic (no): 57.0 (14.0)	< and > 60	left ventricular assist device (LVAD) implantation	Sarcopenia (psoas muscle area)
Hewitt 2019	UK	Median: 54 (36–72)	< and > 60	Emergency surgery	Clinical Frailty Scale
Hope 2017	US	Mean: 57.1 (17.5)	< and > 60	Critically ill, ICU	Clinical Frailty Scale<br>Frailty markers defined as Phenotype criteria plus cognitive and sensory impairment.
Huang 2016	Taiwan	Mean: 51.4 (SD not reported)	< and > 60	Breast cancer	Edmonton Frail Scale
Hyde 2016	Australia	53.8% <60<br>Range: 45–96	< and > 60	General	Frailty Index (20)
Ianas 2013	US	58.0% <50	< and > 60	HIV	Phenotype
Jayanama 2018	Canada	Mean: 46.6 (16.9)	< and > 60	General	Frailty Index (36)
Jha 2016	Australia	Mean: 53.0 (12.0)	< and > 60	Heart failure	Modification of Phenotype
Jha 2016b	Australia	Mean: 53.0 (13.0)	< and > 60	Heart failure	Modification of Phenotype
Jha 2017	Australia	Mean: 51.0 (13.0)	< and > 60	Heart Failure	Phenotype
Johansen 2014	USA	Not Frail Mean: 53.8 (14.4);	< and > 60	Haemodialysis	Development of modified self-report measure based on Phenotype

Study author and date	Country	Mean (SD) / Median (IQR) age<br><br>Where overall sample age is not reported, age details of sub-groups are provided instead.	Is the sample aged entirely under 60 years, or includes those aged under and over 60 years?	Long-term condition group (specify which group) or general population	Measure/approach used
		Self-report Mean: 57.6 (12.1); Performance Mean 51.6 (15.8); Self report + Performance Mean: 62.9 (13)			
Johansen 2014	USA	Mean: 56.8 (14.3)	< and > 60	Haemodialysis	Phenotype
Johansen 2015	USA	Mean: 59.0 (14.0)	< and > 60	Haemodialysis	Phenotype
Johansen 2016	USA	Mean: 57.1 (14.2)	< and > 60	Haemodialysis	Phenotype; substitution of patient-reported measures available on the Medical Outcomes Study 36-Item Short Form (SF-36)
Johansen 2017	USA	Mean: 57.2 (14.2)	< and > 60	Haemodialysis	Phenotype
Johansen 2019	USA	Mean: 57.2 (14.2)	< and > 60	Haemodialysis	Phenotype
Joseph 2017	USA	Mean: 58.0 (12.0)	< and > 60	End-stage heart failure	Phenotype (adapted)
Kahlon 2015	Canada	67.3% aged <60 years	< and > 60	General	Clinical Frailty Scale
Kallianpur 2016	USA	Mean: 50.6 (6.8)	< and > 60	HIV	Phenotype
Kane 2017	USA	Mean: 40.3 (13) (under 65s group)	< and > 60	The metabolic syndrome	Frailty Index (41)
Kang 2017	Korea	Inactive Group Mean: 55.0 (14.0); Intermediate Group Mean: 56.1 (12.2); Active Group Mean: 57.3 (11.6)	< and > 60	Dialysis patients	Modified Phenotype including slowness and poor endurance (PF scale and VT scale of the SF36); physical inactivity; unintentional weight loss
Kang 2017	Korea	Haemodialysis Group Mean: 56.4 (13.2); Peritoneal Dialysis Group Mean: 54.1 (11.9)	< and > 60	Dialysis patients	Modified Phenotype including slowness and poor endurance (PF scale and VT scale of the SF36); physical inactivity; unintentional weight loss
Kanters 2017	Canada	Range 45-85; 21% under 54, 58% under 65	< and > 60	General	Frailty Index (90)

Study author and date	Country	Mean (SD) / Median (IQR) age<br><br>Where overall sample age is not reported, age details of sub-groups are provided instead.	Is the sample aged entirely under 60 years, or includes those aged under and over 60 years?	Long-term condition group (specify which group) or general population	Measure/approach used
Kehler 2017	Canada	Cycle 1 Mean: 44.58 (0.66); Cycle 2 Mean 45.0 (0.56); Cycle 3 Mean: 45.52 (0.63)	< and > 60	General	Frailty Index (23); Phenotype
Keller 2014	USA	Discharge ≤3 days: Mean: 58.87 (15.65); Discharge > 3 days Mean: 60.93 (19.56)	< and > 60	Laparoscopic colorectal surgery	mFI (11)
Kelly 2019	USA	Median: 51 (46,56)	< and > 60	HIV	Phenotype
Kelm 2016	USA	Median: 56 (49,62)	< and > 60	Lung transplant (recipients)	Muscle wasting measured by CT. "Frailty Phenotype is attractive to researchers as the discrete frailty parameters - such as muscle wasting - can be studied and targeted for potential interventions."
Konel 2018	USA	Mean: 54.0 (14.0), range 19-86	< and > 60	Kidney transplant (recipients)	Phenotype
Konstantinidis 2017	USA	Median: (55), range 19-87	< and > 60	Cytoreductive surgery with intraoperative hyperthermic intraperitoneal chemotherapy	mFI (11)
Kooij 2016	Netherlands	Median: 52.8 (48.2,59.5)	< and > 60	HIV	Phenotype
Korada 2017	USA	Mean: 54.0 (7.0)	< and > 60	HIV	Phenotype
Kulkarni 2019	USA	Mean: 57.1, range 20-74	< and > 60	Liver transplant (waiting list)	5-metre walk test; hand grip strength
Kull 2012	Estonia	Young adults group: Mean: 32.8 (4.2) (men), Mean: 33.0 (4.2) (women); Adults group: Mean: 53.7 (8.1) (men), Mean: 55.8 (8.6) (women), range 40-70	< and > 60 but separated	General	Sarco-osteopenia: bone mineral density, muscle mass, grip strength
Kutner 2014	USA	Mean: 57.1 (14.1)	< and > 60	Haemodialysis	Phenotype
Kutner 2014	USA	Mean: 57.1 (14.1) range 20-92	< and > 60	Haemodialysis	Phenotype
Kutner 2014	USA	Mean: 57.2 (14.1)	< and > 60	Haemodialysis	Phenotype

**Study author and date**	**Country**	**Mean (SD) / Median (IQR) age**<br><br>**Where overall sample age is not reported, age details of sub-groups are provided instead.**	**Is the sample aged entirely under 60 years, or includes those aged under and over 60 years?**	**Long-term condition group (specify which group) or general population**	**Measure/approach used**
Lai 2016	USA	Median: 59 (53-63)	< and > 60	End-stage liver disease	4 measures of physical function: grip strength, gait speed, chair stands, balance
Lai 2017	USA	Median: 58 (50-63)	< and > 60	End-stage liver disease	Liver Frailty Index (various candidate models 2-6 items tested)
Lai 2018	USA	Median: 58 (50-63)	< and > 60	End-stage liver disease	Liver Frailty Index (3)
Lai 2019	USA	Median: 57 (49-63)	< and > 60	End-stage liver disease	Liver Frailty Index (3)
Lauerman 2019	USA	61% under 60	< and > 60	Trauma patients (motor vehicle crash)	Radiographic measurements of muscle mass/area; development of composite frailty score
Layton 2017	USA	Mean: 57.0 (11.0)	< and > 60	Lung transplant (potential)	Phenotype
Lee 2017	Korea	Mean: 55.2 (11.9)	< and > 60	Dialysis patients	Kidney Disease Quality of Life SF36
Legge 2019	USA	Mean: 35.7 (13.4)	Not clear	Systemic lupus erythematosus (SLE)	Systemic Lupus International Collaborating Clinics (SLICC) Frailty Index (48)
Legge 2019	USA	Mean: 35.7 (13.3)	Not clear	Systemic lupus erythematosus (SLE)	Systemic Lupus International Collaborating Clinics (SLICC) Frailty Index (48)
Legge 2020	USA	Mean: 35.7 (13.4)	Not clear	Systemic lupus erythematosus (SLE)	Systemic Lupus International Collaborating Clinics (SLICC) Frailty Index (48)
Leopold-George 2019	South Africa	Mean: 50.6 (15.8)	< and > 60	Generic perioperative patients	Clinical Frailty Scale
Li, G 2019	Canada	Mean: 57.7 (12.7)	< and > 60	Rheumatoid arthritis	Frailty Index (32)
Li, X 2019	Sweden	Males: Mean: 58.7 (10.6) (single); Mean: 57.4 (9.6) (DZ twins); Mean: 56.9 (9.2) (MZ twins); Females: Mean: 59.6 (11.2) (single); Mean: 58.5 (10.3) (DZ twins); Mean: 57.8 (10.2) (MZ twins). (Figures at baseline; length of follow up 20 years)	< and > 60	General	Frailty Index (44)

Study author and date	Country	Mean (SD) / Median (IQR) age<br><br>Where overall sample age is not reported, age details of sub-groups are provided instead.	Is the sample aged entirely under 60 years, or includes those aged under and over 60 years?	Long-term condition group (specify which group) or general population	Measure/approach used
Limberg 2019	USA	Laparoscopic cholecystectomy group: Median: 46 (33-59); Laparoscopic adrenalectomy group: Median: 53 (43-62)	< and > 60	Adrenal tumours	mFI (5)
Limpwattana 2019	Thailand	Frail group (n=9) Mean: 64.22 (8.42); Not frail group (n=66) Mean: 58.56 (8.56)	< and > 60	Biliary tract cancer	FRAIL scale
Lisiecki 2013	USA	Mean: 47.1, range 30-71	< and > 60	Trauma patients (mandible fracture)	CT scans to measure morphomic values of muscle as a quantitative marker of frailty
Louwers 2016	USA	Mean: 59.0, range 17-90	< and > 60	Hepatectomy patients	mFI (11)
Luckett 2019	USA	Mean: 51.4 (11.4)	< and > 60	HIV	Phenotype
Lurie 2015	Israel	Mean: 52.0 (8.6)	< and > 60	Myocardial infarction survivors	Frailty Index (40 item)
Lutski 2017	Israel	Mean: 57.0 (6.0 (at baseline))	< and > 60	Coronary heart disease survivors	Phenotype
Maffei 2020	USA	Male: Mean: 48.4 (10.6) Female: Mean: 48.0 (9.8)	< and > 60	HIV	Frailty Index (58); Phenotype
Makhani 2017	USA	Mean: 58, range 18-89	< and > 60	Generic surgery patients	4-level composite scoring system created via combination of Phenotype + Emory Clock Draw Test
Malmstrom 2013	USA	Mean: 59.17 (4.4)	< and > 60	General	FRAIL scale
Malmstrom 2014	USA	Means (at baseline according to tertile): 51.4 (1.1); 56.0 (1.5); 61.5 (2.0). Range 49-65. Follow up at 3 and 9 years.	< and > 60	General	FRAIL scale; Study of Osteoporotic Fractures scale (SOF); Cardiovascular Health Study scale (CHS); Frailty Index (25)
Margolick 2017	USA	HIV-: Nonfrail Mean: 52.3 (10.3); Frail Mean 55.1 (8.9); HIV+: Nonfrail Mean: 49.9 (8.7); Frail Mean: 53.9 (7.8)	< and > 60	HIV	Phenotype

For peer review only

Study author and date	Country	Mean (SD) / Median (IQR) age<br><br>Where overall sample age is not reported, age details of sub-groups are provided instead.	Is the sample aged entirely under 60 years, or includes those aged under and over 60 years?	Long-term condition group (specify which group) or general population	Measure/approach used
Martin 2018	Canada	Mean: 49.5 (17.7)	< and > 60	Intellectual & developmental disabilities	Frailty Index (42)
Mascarella 2020	USA	60.4% under 60: 15-29 (6.6%), 30-44 (21.4%), 45-59 (32.4%).	< and > 60	Thyroid & parathyroid surgery patients	Cervical Endocrine Surgery Risk Index (CERSI)
McAdams-DeMarco 2013	USA	Mean: 53.5 (13.9)	< and > 60	Kidney transplant (recipients)	Phenotype
McAdams-DeMarco 2015	USA	Mean: 53.0 (14.0)	< and > 60	Kidney transplant (recipients)	Phenotype
McAdams-DeMarco 2015	USA	Mean: 54.8 (13.3)	< and > 60	Haemodialysis	Phenotype
McAdams-DeMarco 2015	USA	Mean: 53.3 (14.2)	< and > 60	Kidney transplant (recipients)	Phenotype
McAdams-DeMarco 2015	USA	Mean: 53.0 (14.0)	< and > 60	Kidney transplant (recipients)	Phenotype
McAdams-DeMarco 2016	USA	Mean: 53.3 (14.0) range 18-82	< and > 60	End-stage renal disease	Phenotype
McAdams-DeMarco 2017	USA	Mean: 53.0 (13.9) range 18.7-83.0	< and > 60	Kidney transplant (recipients)	Phenotype
McAdams-DeMarco 2018	USA	Mean: 53.7 (13.5) range 18.3-85.7	< and > 60	End-stage renal disease	Phenotype
McAlister 2016	Canada	Mean: 54.0 (9.9)	< and > 60	Type 2 diabetes	Johns Hopkins Adjusted Clinical Groups (ACG) frailty marker
McChesney 2020	USA	Mean: 59.94 (15.34)	< and > 60	Radial pelvic surgery patients	mFI (11)
McDonagh 2020	Australia	Mean 54.0 (14.0)	< and > 60	Heart failure	3 versions of Phenotype: original criteria; SHARE-FI; St Vincent's Frailty (SVF);
McIntyre 2019	USA	Mean: 57.6 (1.0) range 14-98	< and > 60	Brain haemorrhage - aneurysmal subarachnoid hemorrhage (aSAH)	mFI (11)

Study author and date	Country	Mean (SD) / Median (IQR) age<br><br>Where overall sample age is not reported, age details of sub-groups are provided instead.	Is the sample aged entirely under 60 years, or includes those aged under and over 60 years?	Long-term condition group (specify which group) or general population	Measure/approach used
McIntyre 2020	USA	Mean: 55.4 (1.5)	< and > 60	Brain haemorrhage - angiogram-negative subarachnoid hemorrhages (ANSAH)	mFI (11)
McKenzie 2015	Canada	Mean: 56.2	Not clear	Intellectual & developmental disabilities	Frailty Index (42)
McKenzie 2016	Canada	Mean: 53.9 (17.3)	< and > 60	Intellectual & developmental disabilities	Frailty Index (42)
McNelly 2016	UK	Mean: 55.3, range 48.3-62.3	< and > 60	Critical illness (ICU) patients	Clinical Frailty Scale
Mezuk 2013	USA	Mean: 56.77 (11.1)	< and > 60	General	Phenotype (adapted)
Miller 2011	USA	Mean: 46.0, range 14-80	< and > 60	Hypercortisolism	psoas muscle area and psoas muscle density
Miller 2018	Europe	Not Frail Mean: 43.0 (1.7); Frail Mean: 57.0 (1.8); Severely Frail Mean: 62.0 (2.6)	< and > 60	Spinal deformity	Adult Spinal Deformity Frailty Index (ASD-FI) (36)
Miller 2018	Europe, Asia, North America	Mean: 57.0 (15.0)	< and > 60	Spinal deformity	Adult Spinal Deformity Frailty Index (ASD-FI) (36)
Moayedi 2018	USA	Mean: 53.8 (12.4)	< and > 60	Heart failure	Phenotype
Montgomery 2019	Canada	Mean: 58.0 (17.0)	< and > 60	ICU patients	Clinical Frailty Scale
Montgomery 2020	Australia	Mean: 59.0 (7.0)	< and > 60	Lung transplant (waiting list)	Phenotype
Morgello 2019	USA	Mean: 59.7 (8.9)	< and > 60	HIV	Phenotype
Morley 2012	USA	Various analytic groups according to ADL difficult/dependency and frailty level, ranging from Mean: 56.10 (4.5) - 57.79 (3.9)	< and > 60	General	FRAIL scale

Study author and date	Country	Mean (SD) / Median (IQR) age<br><br>Where overall sample age is not reported, age details of sub-groups are provided instead.	Is the sample aged entirely under 60 years, or includes those aged under and over 60 years?	Long-term condition group (specify which group) or general population	Measure/approach used
Mosquera 2018	USA	Mean: 53.8 (17.09);<br>Median: 54 (18-89)	< and > 60	Thoracoabdominal surgery patients	mFI (11)
Myers 2013	Israel	Mean: 53.2 (8.2)	< and > 60	Myocardial infarction survivors	Frailty Index (40)
Myers 2014	Israel	Grouped on frailty scores ranging from Mean: 52 (8) - 77 (6)	< and > 60	Myocardial infarction survivors	Frailty Index (32)
Myers 2014	Israel	Mean: 58.0 (8.0)	< and > 60	Myocardial infarction survivors	Frailty Index (40)
Nampoothiri 2019	India	Median: 50.5, range 33-64	< and > 60	Myeloma	International Myeloma Working Group (IMWG) frailty score
Nastasi 2018	USA	Mean: 51.6 (14.2), range 18.7-86.0	< and > 60	Kidney transplant (recipients)	Short Physical Performance Battery Phenotype
Ness 2013	USA	Mean: 33.6 (8.1)	< and > 60	Adult survivors of childhood cancer	Phenotype
Ney 2018	Canada	Mean: 55.9 (9.6)	< and > 60	Cirrhosis	MoCA- Clinical Frailty Scale: composite score developed from the Montreal Cognitive Assessment (MoCA) and the Clinical Frailty Scale
Onen 2010	USA	Median: 43, range 33-49	< 60	HIV	Phenotype
Onen 2014	USA	Median: 47 (non-frail median: 46 (40-52); pre-frail median: 48 (43-54); frail median: 53 (45-54)	< 60	HIV	Phenotype
Oppenheim 2018	USA	Mean: 44.6 (11.2), range 18-79	< and > 60	HIV	Frailty Index (26)
Oskutis 2016	USA	Mean: 58.5 (12.8)	< and > 60	Trauma patients (motor vehicle crash)	CT markers of frailty: sarcopenia (psoas muscle mass area); osteopenia (Hounsfield units of trabecular bone); vascular disease (aortic calcification); correlated with injury

Study author and date	Country	Mean (SD) / Median (IQR) age<br><br>Where overall sample age is not reported, age details of sub-groups are provided instead.	Is the sample aged entirely under 60 years, or includes those aged under and over 60 years?	Long-term condition group (specify which group) or general population	Measure/approach used
					severity (Maximum Abbreviated Injury Scale)
Painter 2013	USA	Mean: 55.7 (15.3)	< and > 60	Haemodialysis	FRAIL scale and the Physical Function Scale of the SF36
Palmer 2017	England	Mean: 58.7 (4.4), range 50-65	< and > 60	General primary care group: Health and Employment After Fifty (HEAF) cohort	Phenotype (adapted: self-reported difficulties as replacement for direct assessment)
Pamukcuoglu 2019	USA	Median: 59.0, range 40-73	< and > 60	Hemotopoietic cell transplant	Phenotype
Paolillo 2019	USA	Mean: 50.8 (7.97), range 35-65	< and > 60	HIV	Frailty Index (27)
Passias 2019	USA	Mean: 57.0 (15.1)	< and > 60	Adult spinal deformity (ASD)	mFI (5)
Pathai 2013a	South Africa	HIV-infected and HIV-seronegative groups Mean: 41.1±7.9 and 42.6±9.6 respectively	Not clear	HIV	Phenotype
Pathai 2013b	South Africa	Median of both groups was similar (41.2 vs. 41.9, p= 0.37)	Not clear	HIV	Phenotype
Pathai 2013c	South Africa	Median: 40 (35–46)	Not clear	HIV	Phenotype
Pena 2014	Canada, USA, Europe	NSHS Mean: 48.1 (19.8)<br>SHARE Mean: 64.2 (10.5)<br>Yale-PEP Mean: 78.4 (5.3)	< and > 60	General	Frailty Index (FI-dichotomous)<br><br>Frailty Index (FI-ordinal)
Perez Fernandez 2019	USA	Mean: 53.8 (13.5)	< and > 60	End-stage renal disease (ESRD)	Phenotype
Perez-Zepeda 2016	Mexico	Mean: 44.05 (15.64)	< and > 60	General	Frailty Index (30)
Pierce 2020	USA	Mean: 59.0 (12.0)	< and > 60	adult spinal deformity (ASD) patients	ASD frailty index (ASD-FI)
Piggott 2015	USA	Median: 48	Not clear	HIV	Phenotype

Study author and date	Country	Mean (SD) / Median (IQR) age<br><br>Where overall sample age is not reported, age details of sub-groups are provided instead.	Is the sample aged entirely under 60 years, or includes those aged under and over 60 years?	Long-term condition group (specify which group) or general population	Measure/approach used
Piggott 2017	USA	Mean: 48.0	Not clear	HIV	Phenotype
Prince 2019	USA	Mean:<br>Non-frail - 48.09 (1.21)<br>Pre-frail - 47.85 (1.84)	< 60 only	General	FRAIL scale
Ramaty 2014	Israel	Mean: 48.0 (11.0)	< and > 60	Liver disease	Blood levels of alanine amino transferase
Ravindrarajah 2013	Europe (Multicentre)	Mean: 59.9 (10.8)	< and > 60	General	EMAS Frailty Index<br>EMAS Phenotype<br>EMAS Frailty Scale
Rees 2016	USA	53% < 50 years	< and > 60	HIV	Phenotype
Reid 2018	USA	Mean: 56.7 (14.8)	< and > 60	Adult Spinal Deformity	Adult Spinal Deformity Frailty Index score
Ribeiro 2016	USA	Mean: 59.2 (4.4)	< and > 60	General	FRAIL scale
Richardson 2019	USA	Mean: 57.0 (15.2)	< and > 60	Brain Tumour	mFI (11)
Rietman 2019	Europe (Multicentre)	Not frail mean: 53.4 (11.0)<br>Physically frail mean: 62.8 (10.6)<br>Cognitively frail mean: 64.3 (8.5)<br>Psychologically frail: 57.8 (12.5)	< and > 60	General	4 criteria Phenotype and Tilburg Frailty Indicator
Rochira 2015	Italy	Median: 45.0 (41.0–49.0)	< 60 only	HIV	38-item multimorbidity frailty index
Rockwood 2011	Canada	Mean: 44.0 (18.0)	< and > 60	General	Frailty Index (42)
Rockwood 2014	USA	Limited and Diffuse Groups Mean: 58.5 (11.5) and 54.6 (12.1) respectively	< and > 60	Systemic sclerosis	Canadian Scleroderma Research Group - Frailty Index
Roshanravan 2012	USA	Mean: 59.0	< and > 60	Chronic kidney disease	Phenotype
Rothenberg 2019	USA	Mean: 36.3 (16.3)	< and > 60	Postoperative complications	Risk Analysis Index
Rozenberg 2018	Canada	Cohort 1 : 59.0 (9.0)<br>Cohort 2: 55.0 (D not reported, range: 43-62)	< and > 60	Lung transplant	Phenotype index and 2 alternate phenotypic frailty indices (Index 1: Clinical and Index 2: Dataset)

For peer review only

Study author and date	Country	Mean (SD) / Median (IQR) age<br><br>Where overall sample age is not reported, age details of sub-groups are provided instead.	Is the sample aged entirely under 60 years, or includes those aged under and over 60 years?	Long-term condition group (specify which group) or general population	Measure/approach used
Rubtsova 2018	USA	Mean: 51.0 (SD not reported)	< and > 60	HIV	Phenotype
Sabel 2011	USA	Mean: 52.8 (SD not reported, range: 18–87)	< and > 60	Melanoma	Core muscle size
Sadiq 2018	USA	Mean: 58.0 (14.2)	< and > 60	Post surgery	32 heterogeneous markers capturing limitations in physical, functional, emotional, and social activity domains to indicate severity of frailty
Sahli 2019	Canada	Mean: 51.1 (SD not reported, range 6.6 – 90.2)	< and > 60	Post thyroidectomy	mFI (11)
Salem 2013a	USA	Mean: 52.4 (6.80)	< and > 60	Homeless adults	Frailty Framework among Homeless and Vulnerable Populations (FFHVP)
Salem 2013b	USA	Mean: 52.4 (6.80)	< and > 60	Homeless adults	Frailty Index
Salem 2014	USA	Average: 52.3 (6.8)	< and > 60	Homeless adults	Frailty Framework among Vulnerable Populations (FFVP)
Sandkovsky 2013	USA	Twenty-one participants were 20 to 40 (younger; mean: 31.5), and 20 were more than 50 (older; mean: 56.5)	< and > 60	HIV	Phenotype
Schaenman 2019	USA	Median: for younger (<60) and older (>60) was 43 (34–51) and 67 (60–80) respectively	< and > 60	End-stage renal disease (ESRD)	Frailty Risk Score (FRS)
Schinkel-Ivy 2017	Ireland	Employed mean: 55.6 (5.3)<br>Unexpected retirement mean: 62.1 (6.7)	< and > 60	General	Phenotype
Schopmeyer 2018	Netherlands	Mean: 51.8 (14.5)	< and > 60	Post kidney transplantation	Groningen Frailty Indicator (GFI)
Sharma 2019a	USA	HIV infected and uninfected women Median: 42 and 39 respectively (IQR not reported)	Not clear	HIV	Phenotype

Study author and date	Country	Mean (SD) / Median (IQR) age<br><br>Where overall sample age is not reported, age details of sub-groups are provided instead.	Is the sample aged entirely under 60 years, or includes those aged under and over 60 years?	Long-term condition group (specify which group) or general population	Measure/approach used
Sharma 2019b	USA	HIV infected and uninfected women Median: 42 and 39 respectively	Not clear	HIV	Phenotype
Shin 2017	USA	Average for anterior cervical discectomy and fusion (ACDF) and posterior cervical fusion (PCF) patients: 52.9 and 59.8 respectively	< and > 60	Post Cervical Spinal Fusion	mFI (11)
Singer 2015	USA	Median: 59 (50–64)	< and > 60	Lung transplant	Phenotype
Singer 2018	USA	Median: 59 (50, 65)	< and > 60	Lung transplant	Phenotype
Smit 2015	USA	Mean: 57.0 (6.3)	< and > 60	HIV	Phenotype
Smitherman 2018	USA	57% were 30–39, 27% were >40	Not clear	Cancer	FRAILTY Scale
Soma 2018	Japan	Mean:<br>Control 55.0 (15.0)<br>Urological cancers 70.0 (8.7)	< and > 60	Urological cancers	Frailty discriminant score (FDS)
Stanjek-Cichoracka 2019	Poland	Mean: 49.2 (9.4)	Not clear	End-stage lung disease	Canadian Study of Health and Aging (CSHA) Clinical Frailty Scale
Stenholm 2014	Finland	Mean: 43.6 (9.7)	< and > 60	Obesity	Frailty Index
Subramaniam 2018	USA	2012 - 56.84 (16.76)<br>2015 - 56.42 (16.85)	< and > 60	Post-surgery	mFI (5)
Susanto 2017	Australia	Range 50 - 66 (mean not reported but uses same dataset and cohort as below)	< and > 60	General middle aged women	FRAIL scale
Susanto 2018	Australia	Mean: 52.5	< and > 60	Not reported	FRAIL scale
Swiecicka 2017	Europe (Multicentre)	Mean: 59.0 (SD not reported)	< and > 60	Non-androgenic anabolic hormones	EMAS FP
Sy 2019	USA	Mean: 57.2 (14.2)	< and > 60	End-stage renal disease (ESRD)	Phenotype

Study author and date	Country	Mean (SD) / Median (IQR) age<br><br>Where overall sample age is not reported, age details of sub-groups are provided instead.	Is the sample aged entirely under 60 years, or includes those aged under and over 60 years?	Long-term condition group (specify which group) or general population	Measure/approach used
Sy 2020	USA	Mean: 56.0 (13.0)	< and > 60	Obesity in End-stage renal disease (ESRD)	Phenotype
Tajar 2011	Europe (Multicentre)	Mean: 59.7 (11.0)	< and > 60	General	EMAS FI
Tapper 2018	USA	Mean: 54.0 (10.3)	< and > 60	End-Stage Liver Disease	Frailty Index
Tapper 2019	USA	Mean: 56.0 (13.0)	< and > 60	Cirrhosis	Hand grip and chair stands
Telemi 2018	USA	Median: 46 (33–59)	< and > 60	Colectomy for Ulcerative Colitis	mFI (11)
Traven 2020	USA	Mean: 58.4 (SD not reported)	< and > 60	Post Arthroscopic Rotator Cuff Repair	mFI (5)
Trung 2019	Vietnam	Median: 49 (35–59.5)	< and > 60	Tetanus	Clinical Frailty Score
Tuttle 2018	USA	Mean:<br>DMPN - 57 (12)<br>DM - 53 (9)<br>Control - 67 (7)	< and > 60	Diabesity and Peripheral Neuropathy	9-item, modified Physical Performance Test
Van Pilsum Rasmussen 2018	USA	Mean: 54.4 (14.14)	< and > 60	End-stage renal disease (ESRD)	Phenotype
Vatanen 2017	Finland	Median: 22, range 16–30	< 60 only	high-risk neuroblastoma (HR NBL)	Frailty was defined as ⩾3 of the following conditions: low muscle mass, low energy expenditure, slow running and weakness.
Venado 2019	USA	Median at transplant: 59 (49–66)	< and > 60	Lung transplant	Phenotype
Verheij 2020	Netherlands	Median:<br>Robust = 51.5 (47.6-56.9)<br>Prefrail = 53.3 (48.6-60.1)<br>Frail = 55.1 (49.7-60.0)	< and > 60	HIV	Phenotype
Verschoor 2019	Canada	Premature (30–39; n = 298), early (40–45; n = 1,213), normal	< and > 60	Menopausal women	Frailty measure combining the Frailty Index (14) and Phenotype

For peer review only

Study author and date	Country	Mean (SD) / Median (IQR) age<br><br>Where overall sample age is not reported, age details of sub-groups are provided instead.	Is the sample aged entirely under 60 years, or includes those aged under and over 60 years?	Long-term condition group (specify which group) or general population	Measure/approach used
		(46–54; n = 4,747), and late (55–62; n = 1,121)			
Wade 2016	Europe (Multicentre)	Mean: 59.2 (10.6)	< and > 60	Chronic Widespread pain	European Male Ageing Study Frailty Index (39)
Wallace 2014	Canada	Mean: 46.9 +/-18.7	< and > 60	Chronic Heart Disease	Frailty Index (25)
Wang 2019a	USA	Non-liver disease cohort mean: 50.0 (34-63)<br>Liver disease cohort mean: 56.0 (43-65)<br>FrAILT Cohort mean: 60 (53-65)	< and > 60	End-stage Liver Disease	Liver Frailty Index
Wang 2019b	USA	Mean: 49.8 (7.1)	Not clear	HIV	Phenotype
Warsame 2018	USA	Mean: 54.0 (13.0), range: 20–83	< and > 60	End stage renal disease	Phenotype
Wen 2017	USA	Mean <2 days: 55.67 (17.21)<br>Mean >2 days: 57.85 (16.81)	< and > 60	Ileostomy closure	mFI (11)
Wilkes 2019	USA	Mean: 58.0 (16.0)	< and > 60	Elective Surgical Patient	Risk Analysis Index (RAI)
Williams 2019	UK	Median: 58 (13)	< and > 60	Biobank data	Frailty Index (49)
Wilson 2016a	USA	Median: 31.3, range 18.4-59.7	< 60 only	Survivors of childhood acute lymphoblastic leukemia (ALL)	Phenotype
Wilson 2016b	USA	Median: 57 (51–62.3)	< and > 60	Adult lung transplant recipients	Frailty Deficit Index (32)
Wilson 2019	USA	Mean: 56.0 (SD not reported, range 18-90)	< and > 60	Surgery for Distal Radius Fractures	mFI (5)
Wilson 2019b	USA	Mean: 46.69 (13.62)	< and > 60	Lower Extremity Orthopaedic Trauma Patient	mFI (11)
Womack 2103	USA	Mean: 46.0 (10.0)	Not clear	HIV	Veterans Aging Cohort Study (VACS) Index
Wulunggono 2019	Indonesia	Median: 40.5	Not clear	HIV	Phenotype
Xu 2018	China	Mean: 58.1 (SD not reported)	< and > 60	Degenerative Spinal Disease	mFI (11)

Study author and date	Country	Mean (SD) / Median (IQR) age<br><br>Where overall sample age is not reported, age details of sub-groups are provided instead.	Is the sample aged entirely under 60 years, or includes those aged under and over 60 years?	Long-term condition group (specify which group) or general population	Measure/approach used
Yadla 2018	India	Mean: 44.95 (13.27)	Not clear	hemodialysis	Phenotype
Yagi 2019a	Japan	Mean: 58.0 (17.0)	< and > 60	Adult spinal deformity	mFI (not specified)
Yagi 2019b	Japan	Mean: 55.0 (19.0)	< and > 60	Surgery for Adult Spinal Deformity	mFI (5) and mFI (11)
Yi 2018	China	Mean: 48.8 (14.6)	< and > 60	Continuous Ambulatory Peritoneal Dialysis	Clinical Frailty Scale
Young 2016	UK	Mean: 59.12 (9.3)	< and > 60	General	Frailty Index (39)

For peer review only

For peer review only

Supplementary materials table S3. Number of studies using frailty measures[a]

Measure	Number of studies using measure
Phenotype model (or variation of these criteria)[b]	137
Cumulative deficit model frailty indices[c]	88
CFS	14
Sarcopenia/muscle wasting/muscle size/sarco-osteopenia/morphemic values of muscle (including in combination with weight and mobility)	9
Liver Frailty Index[d]	5
Short Physical Performance Battery	5
Hand grip	3
Walk Test	2
Groningen Frailty Indicator	2
Risk Analysis Index	2
Veterans Aging Cohort Study Index	2
Performance based frailty scale	1
Function Based Frailty scale	1
EQ-5D Item C	1
Comprehensive Frailty Assessment Instrument	1
Tilburg Frailty Indicator	1
Physical Performance Test	1
Blood levels of alanine amino transferase	1
Cervical Endocrine Surgery Risk Index	1
Edmonton Frail Scale	1
Physical Function Scale of SF36	1
Frailty Discriminant Score	1
Frailty Framework among Homeless and Vulnerable Populations	1
Frailty Framework among Vulnerable Populations	1
Morse Fall Risk Score	1
Chair stands	1
International Myeloma Working Group Frailty Score	1
Kidney Disease Quality of Life SF36	1
Frailty Risk Score	1
Composite score from CFS and Montreal Cognitive Assessment	1

For peer review only

Frailty Questionnaire	1
Radiographic frailty markers	1
Vascular disease (CT scan) as indicator of frailty alongside other CT scan indicators of frailty	1
Not reported	1

[a]Numbers are not mutually exclusive as some studies used more than one measure; [b]Includes the FRAIL scale, the Study of Osteoporotic Fractures Scale, the SHARE Frailty Instrument for Primary care, and the John Hopkins Frailty Indicator, all of which use items that overlap with the phenotype model; [c]Includes frailty indices where the number of index deficits varied between studies, the modified Frailty Indices (mFI 5 and 11 item versions), the European Male Ageing Study Frailty Index, the Spinal Tumor Frailty Index, the Adult Spinal Deformity Frailty Index, and the Systemic Lupus International Collaborating Clinics Frailty Index; [d]Although termed a frailty index, the Liver Frailty Index is not based on the cumulative deficit model.

For peer review only

Supplementary materials table S4. Summary of evidence of predictive validity for frailty measures in younger populations

Paper	Sample age Mean/median (SD/IQR)	Sample population	Type of validity PREDICTIVE (MORTALITY AND/OR ADMISSION), CRITERION(CORRELATION OR AGREEMENT))
**Phenotype**			
Ness 2013	Mean: 33.6 (8.1)	Adult survivors of childhood cancer	PREDICTIVE (MORTALITY)
Delgado 2015	Median: 52.0 (42-61)	Chronic Kidney disease	PREDICTIVE (MORTALITY)
McAdams-DeMarco 2018	Mean: 53.7 (13.5)	End-stage renal disease	PREDICTIVE (MORTALITY)
Fitzpatrick 2019	Mean: 54.9	End stage renal disease	PREDICTIVE (MORTALITY)
Chu 2019	Mean: 51.7 (14.0)	End stage renal disease	PREDICTIVE (MORTALITY)
Bao 2012	Mean: 59.6 (14.2)	End stage renal disease	PREDICTIVE (MORTALITY AND ADMISSIONS)
Makhani 2017	Mean: 58.0 (SD not reported, range 18-89)	Generic surgery	PREDICTIVE (MORTALITY)
Johansen 2016	Mean: 57.1 (14.2)	Haemodialysis	PREDICTIVE (MORTALITY)
Johansen 2019	Mean: 57.2 (14.2)	Haemodialysis	PREDICTIVE (MORTALITY)
Lee 2017	Mean: 55.2 (11.9)	Dialysis	PREDICTIVE (ADMISSIONS)
Jha 2016[a,b]	Mean: 53.0 (12.0[a], 13.0[b])	Heart failure	PREDICTIVE (MORTALITY)
Pamukcuoglu et al 2019	Median: 59.0, range 40-73	Hemotopoietic cell transplant	PREDICTIVE (MORTALITY)
Akgun 2014	Mean: 47.6 (8.5)	HIV	PREDICTIVE (MORTALITY AND ADMISSIONS)
Kelly 2019	Median: 51.0 (46.5)	HIV	PREDICTIVE (MORTALITY)
Gustaffson 2017	Mean: 42.6 (8.8)	HIV	PREDICTIVE (MORTALITY)
Verheij 2020	Median:<br>Robust: 51.5 (47.6-56.9)<br>Prefail: 53.3 (48.6-60.1)<br>Frail: 55.1 (49.7-60.0)	HIV	PREDICTIVE (MORTALITY)
McAdams-DeMarco 2013	Mean: 53.5 (13.9)	Kidney transplant	PREDICTIVE (MORTALITY)
McAdams-DeMarco 2015	Mean: 53.0 (14.0)	Kidney transplant	PREDICTIVE (MORTALITY)

Montgomery 2020	Mean: 59.0 (7.0)	Lung transplant	PREDICTIVE (MORTALITY)
Singer 2018	Median: 59.0 (IQR: 50, 65)	Lung Transplant	PREDICTIVE (MORTALITY)
Ravindrarajah 2013	Mean: 59.9 (10.8)	General population	PREDICTIVE (MORTALITY)
Malmstrom 2014	Mean: (at baseline according to tertile): 51.4 (1.1); 56.0 (1.5); 61.5, (2.0).	General population	PREDICTIVE (MORTALITY)
Hope 2017	Mean: 57.1, (17.5)	Critical illness/ICU	PREDICTIVE (MORTALITY) *But as a composite outcome combining death with disability outcome.*
Singer 2015	Median: 59.0 (IQR: 50–64)	Lung Transplant	PREDICTIVE (MORTALITY)
Moayedi 2018	Mean: 53.8 (12.4)	Heart failure	PREDICTIVE (MORTALITY)
McAdams-DeMarco 2017	Mean: 53.0 (13.9)	Kidney transplant	PREDICTIVE (MORTALITY)
Joseph 2017	Mean: 58.0 (12.0)	End-stage heart failure	PREDICTIVE (MORTALITY)
Rozenberg 2018	Cohort 1 mean: 59.0 (9.0)<br>Cohort 2 mean: 55 (43-62)	Lung transplant	PREDICTIVE (MORTALITY)
Johansen 2014	Not frail: mean: 53.8 (14.4); self-report: mean: 57.6 (12.1); performance: 51.6 (15.8); self report + performance: mean 62.9, (13.0)	Haemodialysis	CRITERION (AGREEMENT BETWEEN SELF-REPORT AND PERFORMANCE BASED PHENOTYPE CRITERIA)
McDonagh 2020 *St Vincent's Frailty Measure (Fried criteria)*	Mean: 54.0 (14.0)	Heart Failure	CRITERION (CORRELATION BETWEEN THREE VERSIONS OF PHENOTYPE CRITERIA)
Rozenberg 2018	Cohort 1 mean: 59.0 (9.0)<br>Cohort 2 mean: 55.0 (SD not reported)	Lung transplant	CRITERION (AGREEMENT WITH TWO ALTERNATIVE VERSIONS OF PHENOTYPE CRITERIA BASED ON CLINICAL DATA AND A DATASET)
Erlandson 2012	Median: 50.8 (IQR: 47.7–55.7)	HIV	CRITERION (AGREEMENT WITH THE SHORT PHYSICAL PERFORMANCE BATTERY AND WALK TEST)
**Frailty Indices where the number of index deficits varied between studies (condition specific indices are noted in italics)[a]**			
Kane 2017	Mean: 40.3 (13.0) (under 65s group)	People with metabolic syndrome	PREDICTIVE (MORTALITY)
Li 2019	Multiple means reported by sex and twin group. Reader referred to paper for further details.	General population	PREDICTIVE (MORTALITY)

For peer review only - http://bmjopen.bmj.com/site/about/guidelines.xhtml

Malmstrom 2014	Mean: (at baseline according to tertile): 51.4 (1.1); 56.0 (1.5); 61.5 (2.0).	General population	PREDICTIVE (MORTALITY)
McKenzie 2016	Mean: 53.9 (17.3)	Intellectual & developmental disabilities	PREDICTIVE (ADMISSIONS)
Myers 2014	Grouped on frailty scores ranging from mean 52.0 (8.0) to mean 77.0 (6.0)	Myocardial infarction survivors	PREDICTIVE (MORTALITY)
Rockwood 2011	Mean: 44.0 (18.0)	General population	PREDICTIVE (MORTALITY)
Pena 2014	NSHS: mean: 48.1 (19.8)<br>SHARE: mean: 64.2 (10.5)<br>Yale-PEP: mean: 78.4 (5.3)	General population	PREDICTIVE (MORTALITY)
Jayanama 2018	Mean: 46.6 (16.9)	General population	PREDICTIVE (MORTALITY)
Hyde 2016	Range: 45-96 years, 53.8% aged <60 years	General population	PREDICTIVE (MORTALITY)
Guaraldi 2017	Mean: 51.2 (6.9)	HIV + Liver transplant	PREDICTIVE (MORTALITY)
Guaraldi 2015	Mean: 46.0 (8.0)	HIV	PREDICTIVE (MORTALITY)
Brothers 2017	Mean: 46.8 (7.1)	HIV	PREDICTIVE (MORTALITY)
Ravindrarajah 2013	Mean: 59.9 (10.8)	General population	PREDICTIVE (MORTALITY)
Legge 2019<br>*Systemic Lupus International Collaborating Clinics (SLICC)-FI*	Mean: 35.7 (13.3)	Systemic lupus erythematosus (SLE)	PREDICTIVE (MORTALITY)
Blodgett 2017 (Lab and self report FI)	Mean: 49.4 (19.0)	General population	PREDICTIVE (MORTALITY)
Martin 2018	Mean: 49.5 (17.7)	Intellectual & developmental disabilities	PREDICTIVE (MORTALITY)
Ahmed 2017<br>*Spinal Tumour Frailty Index*	Median: 47, IQR: 28 - 61	Spinal tumour surgery	PREDICTIVE (MORTALITY)
**Frailty Indices: mFI 11**			
Adams 2013	Mean: 54.7 (17.2),	Head and neck surgery	PREDICTIVE (MORTALITY)
Akyar 2018	71.2% aged < 60 years	Emergency surgery	PREDICTIVE (MORTALITY)
Konstantinidis 2017	Median: 55.0, range 19-87	Cytoreductive surgery with intraoperative hyperthermic	PREDICTIVE (MORTALITY)

		intraperitoneal chemotherapy	
McChesney 2020	Mean: 59.9 (5.3)	Radial pelvic surgery patients	PREDICTIVE (MORTALITY)
McIntyre 2020	Mean: 55.4 (1.5)	Brain haemorrhage - angiogram-negative subarachnoid haemorrhages (ANSAH)	PREDICTIVE (MORTALITY)
Mosquera 2018	Mean: 53.8 (17.0)	Thoracoabdominal surgery patients	PREDICTIVE (MORTALITY)
Shin 2017	ACDF group – mean: 52.9 (SD not reported)<br>PCF group – mean: 59.8 (SD not reported)	Post Cervical Spinal Fusion	PREDICTIVE (MORTALITY)<br>*But as composite outcome combining death with any post-surgical complications.*
Louwers 2017	Mean: 59.0 (SD not reported)	Hepatectomy patients	PREDICTIVE (MORTALITY)<br>*But as a composite outcome combining death with any post-surgical complications.*
Fang 2017	Mean: 59.0 (15.0)	Lower extremity amputation	PREDICTIVE (ADMISSIONS)
McIntyre 2019	Mean: 57.6 (1.0)	Brain haemorrhage - aneurysmal subarachnoid haemorrhage (aSAH)	PREDICTIVE (MORTALITY)
Banasek 20	Mean: 50.3 (19.8)	Traumatic spinal cord injury	PREDICTIVE (MORTALITY)
**Frailty Indices: mFI 5**			
Traven 2020	Mean: 58.4 (SD note reported)	Post Arthroscopic Rotator Cuff Repair	PREDICTIVE (MORTALITY AND ADMISSIONS)
Subramaniam 2018	2012 - 56.84 (16.76)<br>2015 - 56.42 (16.85)	Post surgery (general)	PREDICTIVE (MORTALITY AND ADMISSION)<br>CRITERION (CORRELATION WITH MFI 11)
Balla 2019	Mean 55.9 (SD not reported)	Ventral Hernia Repair	CRITERION (CORRELATION WITH MFI 11)
**Short Physical Performance Battery**			
Courtwright 2018	Mean: 53.5 (14.1)	Lung transplant	PREDICTIVE (ADMISSIONS)
Lai 2016	Median: 59 (IQR 53-63)	End-stage liver disease	PREDICTIVE (MORTALITY)

Nastasi 2018	Mean: 51.6 (14.2)	Kidney transplant (recipients)	PREDICTIVE (MORTALITY)
Singer 2018	Median: 59 (IQR: 50, 65)	Lung Transplant	PREDICTIVE (MORTALITY)
Singer 2015	Median: 59 (IQR: 50–64)	Lung Transplant	PREDICTIVE (MORTALITY) *But as a composite outcome combining death with transplant delisting.*
Erlandson 2012	Median: 50.8 (IQR: 47.7–55.7)	HIV	CRITERION (AGREEMENT WITH WALK TEST AND PHENOTYPE CRITERIA)
**Clinical Frailty Scale**			
Fernando 2019	Not frail Mean: 57.6 (18.1) Frail Mean: 69.2 (12.2)	Critical illness/ICU	PREDICTIVE (MORTALITY)
Bagshaw 2016	Mean: 58.5 (4.1)	Critical illness/ICU	PREDICTIVE (MORTALITY AND ADMISSIONS)
Hope 2017	Mean: 57.1 (17.5)	Critical illness/ICU	PREDICTIVE (MORTALITY) *But as a composite outcome combining death with disability outcome.*
Ney 2018 MoCA-CFS	Mean: 55.9 (9.6)	Cirrhosis	PREDICTIVE (MORTALITY)
Kahlon 2015	67.3% aged under 60 years	General population	PREDICTIVE (MORTALITY AND ADMISSIONS)
Montgomery 2019	Mean: 58.0 (17.0)	Critical illness/ICU	PREDICTIVE (MORTALITY)
Hewitt 2019	Median: 54.0 (IQR: 36–72)	Emergency surgery	PREDICTIVE (MORTALITY AND ADMISSIONS)
**FRAIL Scale**			
Griffin 2018	Mean 50.2 (SD not reported)	General population	PREDICTIVE (MORTALITY)
Chode 2016	With diabetes Mean: 57.4 (4.4) Without diabetes Mean: 55.9 (4.4)	Diabetes	PREDICTIVE (MORTALITY)
Chao 2018	Mean: 56.4 (13.8)	Diabetes	PREDICTIVE (MORTALITY AND ADMISSIONS)
Susanto 2017	Range 50–66. Mean age not reported but uses same cohort in another publication (same study team) with a mean age of 52.5 years.	General population	PREDICTIVE (MORTALITY)
Malmstrom 2014	Range 49-65	General population	PREDICTIVE (MORTALITY)

Ravindrarajah 2013<br>EMAS Frail Scale	Mean: 59.9 (10.8)	General population	PREDICTIVE (MORTALITY)
Morley 2012	Various analytic groups according to ADL difficult/dependency and frailty level, ranging from mean 56.1 (4.5) to 57.7 (3.9)	General population	PREDICTIVE (MORTALITY)
**Liver Frailty Index**			
Lai 2019	Median: 57 (IQR 49-63)	End-stage liver disease	PREDICTIVE (MORTALITY)
Lai 2017	Median: 58 (IQR 50-63)	End-stage liver disease	PREDICTIVE (MORTALITY)
Lai 2018	Median: 58 (IQR 50-63)	End-stage liver disease	PREDICTIVE (MORTALITY)
Haugen 2019b	Mean: 55.0 (10.0)	Liver transplant	PREDICTIVE (MORTALITY)
**SOF Frailty Scale and CHS Frailty Scale**			
Malmstrom 2014	Mean: (at baseline according to tertile): 51.4 (1.1); 56.0 (1.5); 61.5 (2.0).	General population	PREDICTIVE (MORTALITY)
**John Hopkins Frailty Indicator**			
Asemota 2019	Frail<br>Mean: 57.1 (SD: 16.9)<br>Non-frail<br>Mean: 51.9 (15.8)	Transphenoidal Pituary Surgery	PREDICTIVE (MORTALITY)
**Walk tests**			
Erlandson 2012	Median: 50.8, IQR: 47.7–55.7	HIV	CRITERION (AGREEMENT WITH PHENOTYPE CRITERIA AND THE SHORT PHYSICAL PERFORMANCE BATTERY)
**Muscle mass/sarcopenia**			
Kelm 2016	Median 56 (IQR 49,62)	Lung transplant	PREDICTIVE (MORTALITY)
Heberton 2016	Sarcopenic (yes): Mean: 53.0 (14.0)<br>Sarcopenic (no): Mean 57.0 (14.0)	Left ventricular assist device (LVAD) implantation	PREDICTIVE (MORTALITY)
**Hand grip**			
Chung 2014	Mean 59.0 (2.0)	Heart failure	PREDICTIVE (MORTALITY)
**CERSI**			
Mascarella 2020	Mean: 56.1 (15.6)	Thyroid & parathyroid surgery patients	PREDICTIVE (MORTALITY)<br>*But as a composite outcome combining death with any adverse event.*

For peer review only

Supplementary materials figure S1. Evidence of validity for cumulative deficit frailty indices by study sample mean/median age (years)

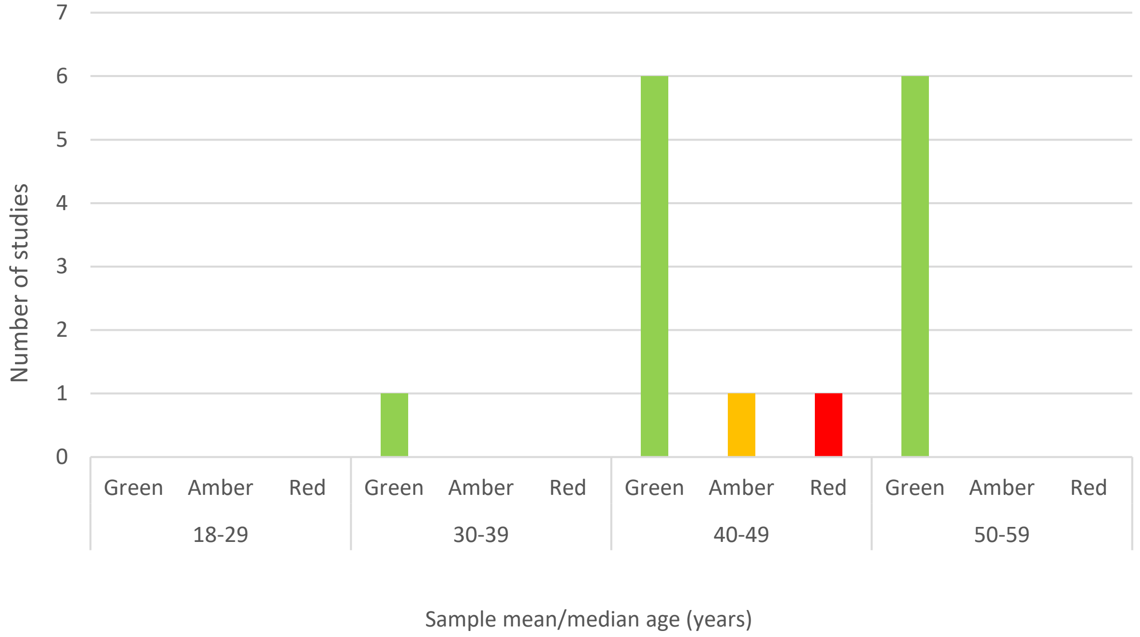

Evidence of validity (green); mixed or uncertain evidence of validity (amber); no evidence of validity (red)

Supplementary materials figure S2. Evidence of validity for phenotype measures by study sample mean/median age (years)

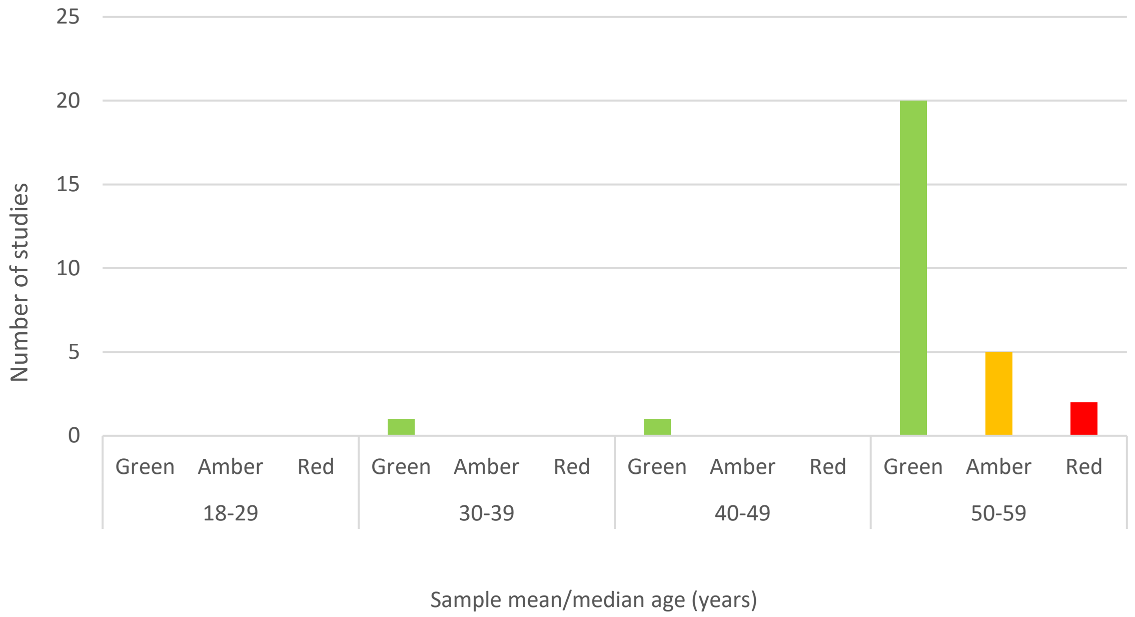

Evidence of validity (green); mixed or uncertain evidence of validity (amber); no evidence of validity (red)

Bibliographic details of included studies

1. Abdel-Kader K, Girard TD, Brummel NE, et al. Acute Kidney Injury and Subsequent Frailty Status in Survivors of Critical Illness: A Secondary Analysis. *Crit Care Med* 2018; **46**(5): e380-e8.
2. Adams P, Ghanem T, Stachler R, Hall F, Velanovich V, Rubinfeld I. Frailty as a predictor of morbidity and mortality in inpatient head and neck surgery. *JAMA Otolaryngol Head Neck Surg* 2013; **139**(8): 783-9.
3. Ahmed AK, Goodwin CR, De la Garza-Ramos R, et al. Predicting Short-Term Outcome After Surgery for Primary Spinal Tumors Based on Patient Frailty. *World Neurosurgery*; **108**: 393-8.
4. Aizpuru M, Staley C, Reisman W, Gottschalk MB, Schenker ML. Determinants of Length of Stay After Operative Treatment for Femur Fractures. *J Orthop Trauma* 2018; **32**(4): 161-6.
5. Akgun KM, Tate JP, Crothers K, et al. An adapted frailty-related phenotype and the VACS index as predictors of hospitalization and mortality in HIV-infected and uninfected individuals. *J Acquir Immune Defic Syndr* 2014; **67**(4): 397-404.
6. Akgun KM, Tate JP, Oursler KK, et al. Association of chronic obstructive pulmonary disease with frailty measurements in HIV-infected and uninfected Veterans. *AIDS* 2016; **30**(14): 2185-93.
7. Akyar S, Armenia SJ, Ratnani P, Merchant AM. The Impact of Frailty on Postoperative Cardiopulmonary Complications in the Emergency General Surgery Population. *Surg J (N Y)* 2018; **4**(2): e66-e77.
8. Alshaikh JT, Amdur R, Sidawy A, Trachiotis G, Kaminski HJ. Thymectomy is safe for myasthenia gravis patients: Analysis of the NSQIP database. *Muscle Nerve* 2016; **53**(3): 370-4.
9. Althoff KN, Jacobson LP, Cranston RD, et al. Age, comorbidities, and AIDS predict a frailty phenotype in men who have sex with men. *J Gerontol A Biol Sci Med Sci* 2014; **69**(2): 189-98.
10. Anderson JE, Inui T, Talamini MA, Chang DC. Cholecystostomy offers no survival benefit in patients with acute acalculous cholecystitis and severe sepsis and shock. *J Surg Res* 2014; **190**(2): 517-21.
11. Andrews JS, Trupin L, Wysham KD, Hough CL, Yelin EH, Katz PP. The Impact of Frailty on Changes in Physical Function and Disease Activity Among Adults With Rheumatoid Arthritis. *ACR Open Rheumatol* 2019; **1**(6): 366-72.
12. Andrews JS, Trupin L, Yelin EH, Hough CL, Covinsky KE, Katz PP. Frailty and reduced physical function go hand in hand in adults with rheumatoid arthritis: a US observational cohort study. *Clin Rheumatol* 2017; **36**(5): 1031-9.
13. Asemota AO, Gallia GL. Impact of frailty on short-term outcomes in patients undergoing transsphenoidal pituitary surgery. *J Neurosurg* 2019; **132**(2): 360-70.
14. Ayesta A, Astiz MTV, Masa MJV, Segovia J, Cosio MDG, Martinez-Selles M. Rationale and design of the FELICITAR registry (Frailty Evaluation After List Inclusion, Characteristics and Influence on Transplantation and Results). *Clin Cardiol* 2018; **41**(3): 293-9.
15. Bagshaw M, Majumdar SR, Rolfson DB, Ibrahim Q, McDermid RC, Stelfox HT. A prospective multicenter cohort study of frailty in younger critically ill patients. *Crit Care* 2016; **20**(1): 175.

16. Balla FM, Yheulon CG, Stetler JL, Patel AD, Lin E, Davis SS. Ventral hernia repair outcomes predicted by a 5-item modified frailty index using NSQIP variables. *Hernia* 2019; **23**(5): 891-8.
17. Banaszek D, Inglis T, Marion TE, et al. Effect of Frailty on Outcome after Traumatic Spinal Cord Injury. *J Neurotrauma* 2020; **37**(6): 839-45.
18. Bao Y, Dalrymple L, Chertow GM, Kaysen GA, Johansen KL. Frailty, dialysis initiation, and mortality in end-stage renal disease. *Arch Intern Med* 2012; **172**(14): 1071-7.
19. Bello GA, Lucchini RG, Teitelbaum SL, Shapiro M, Crane MA, Todd AC. Development of a Physiological Frailty Index for the World Trade Center General Responder Cohort. *Curr Gerontol Geriatr Res* 2018; **2018**: 3725926.
20. Bindawas SM, Vennu V, Stubbs B. Longitudinal Relationship Between Knee Pain Status and Incident Frailty: Data from the Osteoarthritis Initiative. *Pain Med* 2018; **19**(11): 2146-53.
21. Blanco JR, Barrio I, Ramalle-Gomara E, et al. Gender differences for frailty in HIV-infected patients on stable antiretroviral therapy and with an undetectable viral load. *PLoS One* 2019; **14**(5): e0215764.
22. Blodgett JM, Theou O, Howlett SE, Rockwood K. A frailty index from common clinical and laboratory tests predicts increased risk of death across the life course. *Geroscience* 2017; **39**(4): 447-55.
23. Blodgett JM, Theou O, Mitnitski A, Howlett SE, Rockwood K. Associations between a laboratory frailty index and adverse health outcomes across age and sex. *Aging Med (Milton)* 2019; **2**(1): 11-7.
24. Bouillon K, Batty GD, Hamer M, et al. Cardiovascular disease risk scores in identifying future frailty: the Whitehall II prospective cohort study. *Heart* 2013; **99**(10): 737-42.
25. Bouillon K, Kivimaki M, Hamer M, et al. Diabetes risk factors, diabetes risk algorithms, and the prediction of future frailty: the Whitehall II prospective cohort study. *J Am Med Dir Assoc* 2013; **14**(11): 851 e1-6.
26. Bregigeon S, Galinier A, Zaegel-Faucher O, et al. Frailty in HIV infected people: a new risk factor for bone mineral density loss. *AIDS* 2017; **31**(11): 1573-7.
27. Brothers TD, Kirkland S, Theou O, et al. Predictors of transitions in frailty severity and mortality among people aging with HIV. *PLoS One* 2017; **12**(10): e0185352.
28. Buganza-Torio E, Mitchell N, Abraldes JG, et al. Depression in cirrhosis - a prospective evaluation of the prevalence, predictors and development of a screening nomogram. *Aliment Pharmacol Ther* 2019; **49**(2): 194-201.
29. Buitrago DH, Gangadharan SP, Majid A, et al. Frailty Characteristics Predict Respiratory Failure in Patients Undergoing Tracheobronchoplasty. *Ann Thorac Surg* 2018; **106**(3): 836-41.
30. Chao CT, Wang J, Chien KL, group COoGNiNs. Both pre-frailty and frailty increase healthcare utilization and adverse health outcomes in patients with type 2 diabetes mellitus. *Cardiovasc Diabetol* 2018; **17**(1): 130.
31. Chen SY, Stem M, Cerullo M, et al. The Effect of Frailty Index on Early Outcomes after Combined Colorectal and Liver Resections. *J Gastrointest Surg* 2018; **22**(4): 640-9.
32. Chiang JM, Kaysen GA, Segal M, Chertow GM, Delgado C, Johansen KL. Low testosterone is associated with frailty, muscle wasting and physical dysfunction among men receiving hemodialysis: a longitudinal analysis. *Nephrol Dial Transplant* 2019; **34**(5): 802-10.

For peer review only

33. Chode S, Malmstrom TK, Miller DK, Morley JE. Frailty, Diabetes, and Mortality in Middle-Aged African Americans. *J Nutr Health Aging* 2016; **20**(8): 854-9.
34. Chu NM, Deng A, Ying H, et al. Dynamic Frailty Before Kidney Transplantation: Time of Measurement Matters. *Transplantation* 2019; **103**(8): 1700-4.
35. Chu NM, Gross AL, Shaffer AA, et al. Frailty and Changes in Cognitive Function after Kidney Transplantation. *J Am Soc Nephrol* 2019; **30**(2): 336-45.
36. Chung CJ, Wu C, Jones M, et al. Reduced handgrip strength as a marker of frailty predicts clinical outcomes in patients with heart failure undergoing ventricular assist device placement. *J Card Fail* 2014; **20**(5): 310-5.
37. Cook MJ, Oldroyd A, Pye SR, et al. Frailty and bone health in European men. *Age Ageing* 2017; **46**(4): 635-41.
38. Courtwright AM, Zaleski D, Gardo L, et al. Causes, Preventability, and Cost of Unplanned Rehospitalizations Within 30 Days of Discharge After Lung Transplantation. *Transplantation* 2018; **102**(5): 838-44.
39. Courtwright AM, Zaleski D, Tevald M, et al. Discharge frailty following lung transplantation. *Clin Transplant* 2019; **33**(10): e13694.
40. Cron DC, Friedman JF, Winder GS, et al. Depression and Frailty in Patients With End-Stage Liver Disease Referred for Transplant Evaluation. *Am J Transplant* 2016; **16**(6): 1805-11.
41. de Haas SCM, de Jonge EAL, Voortman T, et al. Dietary patterns and changes in frailty status: the Rotterdam study. *Eur J Nutr* 2018; **57**(7): 2365-75.
42. Delgado C, Doyle JW, Johansen KL. Association of frailty with body composition among patients on hemodialysis. *J Ren Nutr* 2013; **23**(5): 356-62.
43. Delgado C, Grimes BA, Glidden DV, Shlipak M, Sarnak MJ, Johansen KL. Association of Frailty based on self-reported physical function with directly measured kidney function and mortality. *BMC Nephrol* 2015; **16**: 203.
44. Denfeld QE, Winters-Stone K, Mudd JO, Hiatt SO, Chien CV, Lee CS. Frequency of and Significance of Physical Frailty in Patients With Heart Failure. *Am J Cardiol* 2017; **119**(8): 1243-9.
45. Derck JE, Thelen AE, Cron DC, et al. Quality of life in liver transplant candidates: frailty is a better indicator than severity of liver disease. *Transplantation* 2015; **99**(2): 340-4.
46. Dolgin NH, Smith AJ, Harrington SG, Movahedi B, Martins PNA, Bozorgzadeh A. Association Between Sarcopenia and Functional Status in Liver Transplant Patients. *Exp Clin Transplant* 2019; **17**(5): 653-64.
47. Dos Santos Mantovani M, Coelho de Carvalho N, Archangelo TE, et al. Frailty predicts surgical complications after kidney transplantation. A propensity score matched study. *PLoS One* 2020; **15**(2): e0229531.
48. Dumond JB, Francis O, Cottrell M, et al. Tenofovir/emtricitabine metabolites and endogenous nucleotide exposures are associated with p16(INK4a) expression in subjects on combination therapy. *Antivir Ther* 2016; **21**(5): 441-5.
49. Erlandson KM, Allshouse AA, Jankowski CM, et al. Comparison of functional status instruments in HIV-infected adults on effective antiretroviral therapy. *HIV Clin Trials* 2012; **13**(6): 324-34.
50. Erlandson KM, Bradford Y, Samuels DC, et al. Mitochondrial DNA Haplogroups and Frailty in Adults Living with HIV. *AIDS Res Hum Retroviruses* 2020; **36**(3): 214-9.
51. Erlandson KM, Ng DK, Jacobson LP, et al. Inflammation, Immune Activation, Immunosenescence, and Hormonal Biomarkers in the Frailty-Related Phenotype of Men With or at Risk for HIV Infection. *J Infect Dis* 2017; **215**(2): 228-37.

For peer review only

52. Erlandson KM, Perez J, Abdo M, et al. Frailty, Neurocognitive Impairment, or Both in Predicting Poor Health Outcomes Among Adults Living With Human Immunodeficiency Virus. *Clin Infect Dis* 2019; **68**(1): 131-8.
53. Erlandson KM, Wu K, Koletar SL, et al. Association Between Frailty and Components of the Frailty Phenotype With Modifiable Risk Factors and Antiretroviral Therapy. *J Infect Dis* 2017; **215**(6): 933-7.
54. Escota GV, Patel P, Brooks JT, et al. Short communication: The Veterans Aging Cohort Study Index is an effective tool to assess baseline frailty status in a contemporary cohort of HIV-infected persons. *AIDS Res Hum Retroviruses* 2015; **31**(3): 313-7.
55. Fang ZB, Hu FY, Arya S, Gillespie TW, Rajani RR. Preoperative frailty is predictive of complications after major lower extremity amputation. *J Vasc Surg* 2017; **65**(3): 804-11.
56. Fernando SM, McIsaac DI, Rochwerg B, et al. Frailty and invasive mechanical ventilation: association with outcomes, extubation failure, and tracheostomy. *Intensive Care Med* 2019; **45**(12): 1742-52.
57. Fitzpatrick J, Sozio SM, Jaar BG, et al. Frailty, body composition and the risk of mortality in incident hemodialysis patients: the Predictors of Arrhythmic and Cardiovascular Risk in End Stage Renal Disease study. *Nephrol Dial Transplant* 2019; **34**(2): 346-54.
58. Franconi I, Theou O, Wallace L, et al. Construct validation of a Frailty Index, an HIV Index and a Protective Index from a clinical HIV database. *PLoS One* 2018; **13**(10): e0201394.
59. Garonzik-Wang JM, Govindan P, Grinnan JW, et al. Frailty and delayed graft function in kidney transplant recipients. *Arch Surg* 2012; **147**(2): 190-3.
60. George EM, Burke WM, Hou JY, et al. Measurement and validation of frailty as a predictor of outcomes in women undergoing major gynaecological surgery. *BJOG* 2016; **123**(3): 455-61.
61. Gordon SJ, Baker N, Kidd M, Maeder A, Grimmer KA. Pre-frailty factors in community-dwelling 40-75 year olds: opportunities for successful ageing. *BMC Geriatr* 2020; **20**(1): 96.
62. Griffin FR, Mode NA, Ejiogu N, Zonderman AB, Evans MK. Frailty in a racially and socioeconomically diverse sample of middle-aged Americans in Baltimore. *PLoS One* 2018; **13**(4): e0195637.
63. Groß M, Herr A, Hower M, et al. Unemployment, health, and education of HIV-infected males in Germany. *International Journal of Public Health* 2016; **61**(5): 593-602.
64. Guaraldi G, Brothers TD, Zona S, et al. A frailty index predicts survival and incident multimorbidity independent of markers of HIV disease severity. *AIDS* 2015; **29**(13): 1633-41.
65. Guaraldi G, De Francesco D, Milic J, et al. The Interplay Between Age and Frailty in People Living With HIV: Results From an 11-Year Follow-up Observational Study. *Open Forum Infect Dis* 2019; **6**(5): ofz199.
66. Guaraldi G, Dolci G, Zona S, et al. A frailty index predicts post-liver transplant morbidity and mortality in HIV-positive patients. *AIDS Res Ther* 2017; **14**(1): 37.
67. Guaraldi G, Francesco D, Malagoli A, et al. Compression of frailty in adults living with HIV. *BMC Geriatr* 2019; **19**(1): 229.
68. Guaraldi G, Franconi I, Milic J, et al. Thymus Imaging Detection and Size Is Inversely Associated With Metabolic Syndrome and Frailty in People With HIV. *Open Forum Infect Dis* 2019; **6**(10): ofz435.

For peer review only

69. Guaraldi G, Malagoli A, Theou O, et al. Correlates of frailty phenotype and frailty index and their associations with clinical outcomes. *HIV Med* 2017; **18**(10): 764-71.
70. Guaraldi G, Zona S, Silva AR, et al. The dynamic association between Frailty, CD4 and CD4/CD8 ratio in people aging with HIV. *PLoS One* 2019; **14**(2): e0212283.
71. Gustafson DR, Shi Q, Holman S, et al. Predicting death over 8 years in a prospective cohort of HIV-infected women: the Women's Interagency HIV Study. *BMJ Open* 2017; **7**(6): e013993.
72. Gustafson DR, Shi Q, Thurn M, et al. Frailty and Constellations of Factors in Aging HIV-infected and Uninfected Women--The Women's Interagency HIV Study. *J Frailty Aging* 2016; **5**(1): 43-8.
73. Hadenfeldt CJ, Darabaris M, Aufdenkamp M. Frailty Assessment in Patients Utilizing a Free Clinic. *J Health Care Poor Underserved* 2017; **28**(4): 1423-35.
74. Haider S, Grabovac I, Berner C, et al. Frailty in seropositive rheumatoid arthritis patients of working age: a cross-sectional study. *Clin Exp Rheumatol* 2019; **37**(4): 585-92.
75. Hampton JP, Owodunni OP, Bettick D, et al. Compliance to an enhanced recovery pathway among patients with a high frailty index after major gastrointestinal surgery results in improved 30-day outcomes. *Surgery* 2019; **166**(1): 75-81.
76. Harland TA, Wang M, Gunaydin D, et al. Frailty as a Predictor of Neurosurgical Outcomes in Brain Tumor Patients. *World Neurosurg* 2020; **133**: e813-e8.
77. Haugen CE, Chu NM, Ying H, et al. Frailty and Access to Kidney Transplantation. *Clin J Am Soc Nephrol* 2019; **14**(4): 576-82.
78. Haugen CE, McAdams-DeMarco M, Verna EC, et al. Association Between Liver Transplant Wait-list Mortality and Frailty Based on Body Mass Index. *JAMA Surg* 2019; **154**(12): 1103-9.
79. Haugen CE, Mountford A, Warsame F, et al. Incidence, Risk Factors, and Sequelae of Post-kidney Transplant Delirium. *J Am Soc Nephrol* 2018; **29**(6): 1752-9.
80. Haugen CE, Thomas AG, Chu NM, et al. Prevalence of frailty among kidney transplant candidates and recipients in the United States: Estimates from a National Registry and Multicenter Cohort Study. *Am J Transplant* 2020; **20**(4): 1170-80.
81. Hayek S, Gibson TM, Leisenring WM, et al. Prevalence and Predictors of Frailty in Childhood Cancer Survivors and Siblings: A Report From the Childhood Cancer Survivor Study. *J Clin Oncol* 2020; **38**(3): 232-47.
82. Heberton GA, Nassif M, Bierhals A, et al. Usefulness of Psoas Muscle Area Determined by Computed Tomography to Predict Mortality or Prolonged Length of Hospital Stay in Patients Undergoing Left Ventricular Assist Device Implantation. *Am J Cardiol* 2016; **118**(9): 1363-7.
83. Hewitt J, Carter B, McCarthy K, et al. Frailty predicts mortality in all emergency surgical admissions regardless of age. An observational study. *Age Ageing* 2019; **48**(3): 388-94.
84. Hope AA, Hsieh SJ, Petti A, Hurtado-Sbordoni M, Verghese J, Gong MN. Assessing the Usefulness and Validity of Frailty Markers in Critically Ill Adults. *Ann Am Thorac Soc* 2017; **14**(6): 952-9.
85. Huang SM, Tseng LM, Chien LY, et al. Effects of non-sporting and sporting qigong on frailty and quality of life among breast cancer patients receiving chemotherapy. *Eur J Oncol Nurs* 2016; **21**: 257-65.

For peer review only

86. Hyde Z, Flicker L, Smith K, et al. Prevalence and incidence of frailty in Aboriginal Australians, and associations with mortality and disability. *Maturitas* 2016; **87**: 89-94.
87. Ianas V, Berg E, Mohler MJ, Wendel C, Klotz SA. Antiretroviral therapy protects against frailty in HIV-1 infection. *J Int Assoc Provid AIDS Care* 2013; **12**(1): 62-6.
88. Jayanama K, Theou O, Blodgett JM, Cahill L, Rockwood K. Frailty, nutrition-related parameters, and mortality across the adult age spectrum. *BMC Med* 2018; **16**(1): 188.
89. Jha SR, Hannu MK, Chang S, et al. The Prevalence and Prognostic Significance of Frailty in Patients With Advanced Heart Failure Referred for Heart Transplantation. *Transplantation* 2016; **100**(2): 429-36.
90. Jha SR, Hannu MK, Gore K, et al. Cognitive impairment improves the predictive validity of physical frailty for mortality in patients with advanced heart failure referred for heart transplantation. *J Heart Lung Transplant* 2016; **35**(9): 1092-100.
91. Jha SR, Hannu MK, Newton PJ, et al. Reversibility of Frailty After Bridge-to-Transplant Ventricular Assist Device Implantation or Heart Transplantation. *Transplant Direct* 2017; **3**(7): e167.
92. Johansen KL, Dalrymple LS, Delgado C, et al. Factors Associated with Frailty and Its Trajectory among Patients on Hemodialysis. *Clin J Am Soc Nephrol* 2017; **12**(7): 1100-8.
93. Johansen KL, Dalrymple LS, Delgado C, et al. Association between body composition and frailty among prevalent hemodialysis patients: a US Renal Data System special study. *J Am Soc Nephrol* 2014; **25**(2): 381-9.
94. Johansen KL, Dalrymple LS, Delgado C, et al. Comparison of self-report-based and physical performance-based frailty definitions among patients receiving maintenance hemodialysis. *Am J Kidney Dis* 2014; **64**(4): 600-7.
95. Johansen KL, Dalrymple LS, Glidden D, et al. Association of Performance-Based and Self-Reported Function-Based Definitions of Frailty with Mortality among Patients Receiving Hemodialysis. *Clin J Am Soc Nephrol* 2016; **11**(4): 626-32.
96. Johansen KL, Delgado C, Kaysen GA, et al. Frailty Among Patients Receiving Hemodialysis: Evolution of Components and Associations With Mortality. *J Gerontol A Biol Sci Med Sci* 2019; **74**(3): 380-6.
97. Johansen KL, Painter P, Delgado C, Doyle J. Characterization of physical activity and sitting time among patients on hemodialysis using a new physical activity instrument. *J Ren Nutr* 2015; **25**(1): 25-30.
98. Joseph SM, Manghelli JL, Vader JM, et al. Prospective Assessment of Frailty Using the Fried Criteria in Patients Undergoing Left Ventricular Assist Device Therapy. *Am J Cardiol* 2017; **120**(8): 1349-54.
99. Kahlon S, Pederson J, Majumdar SR, et al. Association between frailty and 30-day outcomes after discharge from hospital. *CMAJ* 2015; **187**(11): 799-804.
100. Kallianpur KJ, Sakoda M, Gangcuangco LM, et al. Frailty Characteristics in Chronic HIV Patients are Markers of White Matter Atrophy Independently of Age and Depressive Symptoms: A Pilot Study. *Open Med J* 2016; **3**: 138-52.
101. Kane AE, Gregson E, Theou O, Rockwood K, Howlett SE. The association between frailty, the metabolic syndrome, and mortality over the lifespan. *Geroscience* 2017; **39**(2): 221-9.

For peer review only

102. Kang SH, Do JY, Jeong HY, Lee SY, Kim JC. The Clinical Significance of Physical Activity in Maintenance Dialysis Patients. *Kidney Blood Press Res* 2017; **42**(3): 575-86.
103. Kang SH, Do JY, Lee SY, Kim JC. Effect of dialysis modality on frailty phenotype, disability, and health-related quality of life in maintenance dialysis patients. *PLoS One* 2017; **12**(5): e0176814.
104. Kanters DM, Griffith LE, Hogan DB, Richardson J, Patterson C, Raina P. Assessing the measurement properties of a Frailty Index across the age spectrum in the Canadian Longitudinal Study on Aging. *J Epidemiol Community Health* 2017; **71**(8): 794-9.
105. Kehler DS, Ferguson T, Stammers AN, et al. Prevalence of frailty in Canadians 18-79 years old in the Canadian Health Measures Survey. *BMC Geriatr* 2017; **17**(1): 28.
106. Keller DS, Bankwitz B, Nobel T, Delaney CP. Using frailty to predict who will fail early discharge after laparoscopic colorectal surgery with an established recovery pathway. *Dis Colon Rectum* 2014; **57**(3): 337-42.
107. Kelly SG, Wu K, Tassiopoulos K, Erlandson KM, Koletar SL, Palella FJ. Frailty Is an Independent Risk Factor for Mortality, Cardiovascular Disease, Bone Disease, and Diabetes Among Aging Adults With Human Immunodeficiency Virus. *Clin Infect Dis* 2019; **69**(8): 1370-6.
108. Kelm DJ, Bonnes SL, Jensen MD, et al. Pre-transplant wasting (as measured by muscle index) is a novel prognostic indicator in lung transplantation. *Clin Transplant* 2016; **30**(3): 247-55.
109. Konel JM, Warsame F, Ying H, et al. Depressive symptoms, frailty, and adverse outcomes among kidney transplant recipients. *Clin Transplant* 2018; **32**(10): e13391.
110. Konstantinidis IT, Chouliaras K, Levine EA, Lee B, Votanopoulos KI. Frailty Correlates with Postoperative Mortality and Major Morbidity After Cytoreductive Surgery with Hyperthermic Intraperitoneal Chemotherapy. *Ann Surg Oncol* 2017; **24**(13): 3825-30.
111. Kooij KW, Wit FW, Schouten J, et al. HIV infection is independently associated with frailty in middle-aged HIV type 1-infected individuals compared with similar but uninfected controls. *AIDS* 2016; **30**(2): 241-50.
112. Korada SKC, Zhao D, Tibuakuu M, et al. Frailty and subclinical coronary atherosclerosis: The Multicenter AIDS Cohort Study (MACS). *Atherosclerosis* 2017; **266**: 240-7.
113. Kulkarni SS, Chen H, Josbeno DA, et al. Gait Speed and Grip Strength Are Associated With Dropping Out of the Liver Transplant Waiting List. *Transplant Proc* 2019; **51**(3): 794-7.
114. Kull M, Kallikorm R, Lember M. Impact of a new sarco-osteopenia definition on health-related quality of life in a population-based cohort in Northern Europe. *J Clin Densitom* 2012; **15**(1): 32-8.
115. Kutner NG, Zhang R, Allman RM, Bowling CB. Correlates of ADL difficulty in a large hemodialysis cohort. *Hemodial Int* 2014; **18**(1): 70-7.
116. Kutner NG, Zhang R, Huang Y, McClellan WM, Soltow QA, Lea J. Risk factors for frailty in a large prevalent cohort of hemodialysis patients. *Am J Med Sci* 2014; **348**(4): 277-82.
117. Kutner NG, Zhang R, Huang Y, Wasse H. Falls among hemodialysis patients: potential opportunities for prevention? *Clin Kidney J* 2014; **7**(3): 257-63.
118. Lai JC, Covinsky KE, Dodge JL, et al. Development of a novel frailty index to predict mortality in patients with end-stage liver disease. *Hepatology* 2017; **66**(2): 564-74.

For peer review only

119. Lai JC, Covinsky KE, McCulloch CE, Feng S. The Liver Frailty Index Improves Mortality Prediction of the Subjective Clinician Assessment in Patients With Cirrhosis. *Am J Gastroenterol* 2018; **113**(2): 235-42.
120. Lai JC, Dodge JL, Sen S, Covinsky K, Feng S. Functional decline in patients with cirrhosis awaiting liver transplantation: Results from the functional assessment in liver transplantation (FrAILT) study. *Hepatology* 2016; **63**(2): 574-80.
121. Lai JC, Rahimi RS, Verna EC, et al. Frailty Associated With Waitlist Mortality Independent of Ascites and Hepatic Encephalopathy in a Multicenter Study. *Gastroenterology* 2019; **156**(6): 1675-82.
122. Lauerman MH, Raithel M, Kufera J, et al. Comparison of individual and composite radiographic markers of frailty in trauma. *Injury* 2019; **50**(1): 149-55.
123. Layton AM, Armstrong HF, Baldwin MR, et al. Frailty and maximal exercise capacity in adult lung transplant candidates. *Respir Med* 2017; **131**: 70-6.
124. Lee SY, Yang DH, Hwang E, et al. The Prevalence, Association, and Clinical Outcomes of Frailty in Maintenance Dialysis Patients. *J Ren Nutr* 2017; **27**(2): 106-12.
125. Legge A, Kirkl, S., et al. Prediction of Damage Accrual in Systemic Lupus Erythematosus Using the Systemic Lupus International Collaborating Clinics Frailty Index. *Arthritis & Rheumatology* 2019.
126. Legge A, Kirkland S, Rockwood K, et al. Evaluating the Properties of a Frailty Index and Its Association With Mortality Risk Among Patients With Systemic Lupus Erythematosus. *Arthritis Rheumatol* 2019; **71**(8): 1297-307.
127. Legge A, Kirkland S, Rockwood K, et al. Construction of a Frailty Index as a Novel Health Measure in Systemic Lupus Erythematosus. *J Rheumatol* 2020; **47**(1): 72-81.
128. Leopold-George NTN, Nethathe GD. Frailty in perioperative patients in three South African academic hospitals. *S Afr Med J* 2019; **109**(7): 535-40.
129. Li G, Chen M, Li X, et al. Frailty and risk of osteoporotic fractures in patients with rheumatoid arthritis: Data from the Ontario Best Practices Research Initiative. *Bone* 2019; **127**: 129-34.
130. Li X, Ploner A, Karlsson IK, et al. The frailty index is a predictor of cause-specific mortality independent of familial effects from midlife onwards: a large cohort study. *BMC Med* 2019; **17**(1): 94.
131. Limberg J, Ullmann TM, Gray KD, et al. Laparoscopic Adrenalectomy Has the Same Operative Risk as Routine Laparoscopic Cholecystectomy. *J Surg Res* 2019; **241**: 228-34.
132. Limpawattana P, Wirasorn K, Sookprasert A, et al. Frailty Syndrome in Biliary Tract Cancer Patients: Prevalence and Associated Factors. *Asian Pac J Cancer Prev* 2019; **20**(5): 1497-501.
133. Lisiecki J, Zhang P, Wang L, et al. Morphomic measurement of the temporalis muscle and zygomatic bone as novel predictors of hospital-based clinical outcomes in patients with mandible fracture. *J Craniofac Surg* 2013; **24**(5): 1577-81.
134. Louwers L, Schnickel G, Rubinfeld I. Use of a simplified frailty index to predict Clavien 4 complications and mortality after hepatectomy: analysis of the National Surgical Quality Improvement Project database. *American Journal of Surgery* 2016; **211**(6): 1071-6.
135. Luckett P, Paul RH, Navid J, et al. Deep Learning Analysis of Cerebral Blood Flow to Identify Cognitive Impairment and Frailty in Persons Living With HIV. *J Acquir Immune Defic Syndr* 2019; **82**(5): 496-502.

For peer review only

136. Lurie I, Myers V, Goldbourt U, Gerber Y. Perceived social support following myocardial infarction and long-term development of frailty. *Eur J Prev Cardiol* 2015; **22**(10): 1346-53.
137. Lutski M, Tanne D, Goldbourt U. Tall stature in coronary heart disease patients is associated with decreased risk of frailty in late life. *Geriatr Gerontol Int* 2017; **17**(9): 1270-7.
138. Maffei VJ, Ferguson TF, Brashear MM, et al. Lifetime alcohol use among persons living with HIV is associated with frailty. *AIDS* 2020; **34**(2): 245-54.
139. Makhani SS, Kim FY, Liu Y, et al. Cognitive Impairment and Overall Survival in Frail Surgical Patients. *J Am Coll Surg* 2017; **225**(5): 590-600 e1.
140. Malmstrom TK, Miller DK, Herning MM, Morley JE. Low appendicular skeletal muscle mass (ASM) with limited mobility and poor health outcomes in middle-aged African Americans. *J Cachexia Sarcopenia Muscle* 2013; **4**(3): 179-86.
141. Malmstrom TK, Miller DK, Morley JE. A comparison of four frailty models. *J Am Geriatr Soc* 2014; **62**(4): 721-6.
142. Margolick JB, Bream JH, Martinez-Maza O, et al. Frailty and Circulating Markers of Inflammation in HIV+ and HIV- Men in the Multicenter AIDS Cohort Study. *J Acquir Immune Defic Syndr* 2017; **74**(4): 407-17.
143. Martin L, McKenzie K, Ouellette-Kuntz H. Once frail, always frail? Frailty transitions in home care users with intellectual and developmental disabilities. *Geriatr Gerontol Int* 2018; **18**(4): 547-53.
144. Mascarella MA, Milad D, Richardson K, et al. Preoperative Risk Index Among Patients Undergoing Thyroid or Parathyroid Surgery. *Jama Otolaryngol* 2020; **146**(1): 7-12.
145. McAdams-DeMarco MA, Isaacs K, Darko L, et al. Changes in Frailty After Kidney Transplantation. *J Am Geriatr Soc* 2015; **63**(10): 2152-7.
146. McAdams-DeMarco MA, Law A, King E, et al. Frailty and mortality in kidney transplant recipients. *Am J Transplant* 2015; **15**(1): 149-54.
147. McAdams-DeMarco MA, Law A, Salter ML, et al. Frailty and early hospital readmission after kidney transplantation. *Am J Transplant* 2013; **13**(8): 2091-5.
148. McAdams-DeMarco MA, Law A, Tan J, et al. Frailty, mycophenolate reduction, and graft loss in kidney transplant recipients. *Transplantation* 2015; **99**(4): 805-10.
149. McAdams-DeMarco MA, Tan J, Salter ML, et al. Frailty and Cognitive Function in Incident Hemodialysis Patients. *Clin J Am Soc Nephrol* 2015; **10**(12): 2181-9.
150. McAdams-DeMarco MA, Ying H, Olorundare I, et al. Frailty and Health-Related Quality of Life in End Stage Renal Disease Patients of All Ages. *J Frailty Aging* 2016; **5**(3): 174-9.
151. McAdams-DeMarco MA, Ying H, Olorundare I, et al. Individual Frailty Components and Mortality in Kidney Transplant Recipients. *Transplantation* 2017; **101**(9): 2126-32.
152. McAdams-DeMarco MA, Ying H, Thomas AG, et al. Frailty, Inflammatory Markers, and Waitlist Mortality Among Patients With End-stage Renal Disease in a Prospective Cohort Study. *Transplantation* 2018; **102**(10): 1740-6.
153. McAlister FA, Youngson E, Eurich DT. Treated glycosylated hemoglobin levels in individuals with diabetes mellitus vary little by health status: A retrospective cohort study. *Medicine (Baltimore)* 2016; **95**(24): e3894.
154. McChesney SL, Canter DJ, Monlezun DJ, Green H, Margolin DA. Modified Frailty Index Predicts Postoperative Outcomes in Patients Undergoing Radical Pelvic Surgery. *Am Surg* 2020; **86**(2): 95-103.

For peer review only

155. McDonagh J, Salamonson Y, Ferguson C, et al. Evaluating the convergent and discriminant validity of three versions of the frailty phenotype in heart failure: results from the FRAME-HF study. *Eur J Cardiovasc Nurs* 2020; **19**(1): 55-63.
156. McIntyre M, Gandhi C, Dragonette J, et al. Increasing Frailty Predicts Worse Outcomes and Increased Complications After Angiogram-Negative Subarachnoid Hemorrhages. *World Neurosurg* 2020; **134**: e181-e8.
157. McIntyre MK, Gandhi C, Long A, et al. Age predicts outcomes better than frailty following aneurysmal subarachnoid hemorrhage: A retrospective cohort analysis. *Clin Neurol Neurosurg* 2019; **187**: 105558.
158. McKenzie K, Ouellette-Kuntz H, Martin L. Using an accumulation of deficits approach to measure frailty in a population of home care users with intellectual and developmental disabilities: an analytical descriptive study. *BMC Geriatr* 2015; **15**: 170.
159. McKenzie K, Ouellette-Kuntz H, Martin L. Frailty as a Predictor of Institutionalization Among Adults With Intellectual and Developmental Disabilities. *Intellect Dev Disabil* 2016; **54**(2): 123-35.
160. McNelly AS, Rawal J, Shrikrishna D, et al. An Exploratory Study of Long-Term Outcome Measures in Critical Illness Survivors: Construct Validity of Physical Activity, Frailty, and Health-Related Quality of Life Measures. *Crit Care Med* 2016; **44**(6): e362-9.
161. Mezuk B, Lohman M, Dumenci L, Lapane KL. Are depression and frailty overlapping syndromes in mid- and late-life? A latent variable analysis. *Am J Geriatr Psychiatry* 2013; **21**(6): 560-9.
162. Miller BS, Ignatoski KM, Daignault S, et al. A quantitative tool to assess degree of sarcopenia objectively in patients with hypercortisolism. *Surgery* 2011; **150**(6): 1178-85.
163. Miller EK, Lenke LG, Neuman BJ, et al. External Validation of the Adult Spinal Deformity (ASD) Frailty Index (ASD-FI) in the Scoli-RISK-1 Patient Database. *Spine (Phila Pa 1976)* 2018; **43**(20): 1426-31.
164. Miller EK, Vila-Casademunt A, Neuman BJ, et al. External validation of the adult spinal deformity (ASD) frailty index (ASD-FI). *Eur Spine J* 2018; **27**(9): 2331-8.
165. Moayedi Y, Duero Posada JG, Foroutan F, et al. The prognostic significance of frailty compared to peak oxygen consumption and B-type natriuretic peptide in patients with advanced heart failure. *Clin Transplant* 2018; **32**(1): 01.
166. Montgomery CL, Zuege DJ, Rolfson DB, et al. Implementation of population-level screening for frailty among patients admitted to adult intensive care in Alberta, Canada. *Can J Anaesth* 2019; **66**(11): 1310-9.
167. Montgomery E, Macdonald PS, Newton PJ, et al. Frailty as a Predictor of Mortality in Patients With Interstitial Lung Disease Referred for Lung Transplantation. *Transplantation* 2020; **104**(4): 864-72.
168. Morgello S, Gensler G, Sherman S, et al. Frailty in medically complex individuals with chronic HIV. *AIDS* 2019; **33**(10): 1603-11.
169. Morley JE, Malmstrom TK, Miller DK. A simple frailty questionnaire (FRAIL) predicts outcomes in middle aged African Americans. *J Nutr Health Aging* 2012; **16**(7): 601-8.
170. Mosquera C, Bermudez JM, Evans JL, Spaniolas K, MacGillivary DC, Fitzgerald TL. Frailty Predicts Failure to Rescue after Thoracoabdominal Operation. *J Am Coll Surg* 2018; **226**(6): 978-86.
171. Myers V, Broday DM, Steinberg DM, Yuval, Drory Y, Gerber Y. Exposure to particulate air pollution and long-term incidence of frailty after myocardial infarction. *Ann Epidemiol* 2013; **23**(7): 395-400.

For peer review only

172. Myers V, Drory Y, Gerber Y, Israel Study Group on First Acute Myocardial I. Clinical relevance of frailty trajectory post myocardial infarction. *Eur J Prev Cardiol* 2014; **21**(6): 758-66.
173. Myers V, Drory Y, Goldbourt U, Gerber Y. Multilevel socioeconomic status and incidence of frailty post myocardial infarction. *Int J Cardiol* 2014; **170**(3): 338-43.
174. Nampoothiri RV, Kasudhan KS, Patil AN, et al. Impact of frailty, melphalan pharmacokinetics, and pharmacogenetics on outcomes post autologous hematopoietic cell transplantation for multiple myeloma. *Bone Marrow Transplant* 2019; **54**(12): 2088-95.
175. Nastasi AJ, McAdams-DeMarco MA, Schrack J, et al. Pre-Kidney Transplant Lower Extremity Impairment and Post-Kidney Transplant Mortality. *Am J Transplant* 2018; **18**(1): 189-96.
176. Ness KK, Krull KR, Jones KE, et al. Physiologic frailty as a sign of accelerated aging among adult survivors of childhood cancer: a report from the St Jude Lifetime cohort study. *J Clin Oncol* 2013; **31**(36): 4496-503.
177. Ney M, Tangri N, Dobbs B, et al. Predicting Hepatic Encephalopathy-Related Hospitalizations Using a Composite Assessment of Cognitive Impairment and Frailty in 355 Patients With Cirrhosis. *Am J Gastroenterol* 2018; **113**(10): 1506-15.
178. Onen NF, Patel P, Baker J, et al. Frailty and Pre-Frailty in a Contemporary Cohort of HIV-Infected Adults. *J Frailty Aging* 2014; **3**(3): 158-65.
179. Onen NF, Shacham E, Stamm KE, Turner Overton E. Sexual behaviors and results of bacterial sexually transmitted infections testing among frail HIV-infected individuals. *J Int Assoc Physicians AIDS Care (Chic)* 2010; **9**(1): 30-3.
180. Oppenheim H, Paolillo EW, Moore RC, et al. Neurocognitive functioning predicts frailty index in HIV. *Neurology* 2018; **91**(2): e162-e70.
181. Oskutis MQ, Lauerman MH, Kufera JA, et al. Are frailty markers associated with serious thoracic and spinal injuries among motor vehicle crash occupants? *J Trauma Acute Care Surg* 2016; **81**(1): 156-61.
182. Painter P, Kuskowski M. A closer look at frailty in ESRD: getting the measure right. *Hemodial Int* 2013; **17**(1): 41-9.
183. Palmer KT, D'Angelo S, Harris EC, et al. Frailty, prefrailty and employment outcomes in Health and Employment After Fifty (HEAF) Study. *Occup Environ Med* 2017; **74**(7): 476-82.
184. Pamukcuoglu M, Bhatia S, Weisdorf DJ, et al. Hematopoietic Cell Transplant-Related Toxicities and Mortality in Frail Recipients. *Biol Blood Marrow Transplant* 2019; **25**(12): 2454-60.
185. Paolillo EW, Saloner R, Montoya JL, et al. Frailty in Comorbid HIV and Lifetime Methamphetamine Use Disorder: Associations with Neurocognitive and Everyday Functioning. *AIDS Res Hum Retroviruses* 2019; **35**(11-12): 1044-53.
186. Passias PG, Bortz CA, Pierce KE, et al. Decreased rates of 30-day perioperative complications following ASD-corrective surgery: A modified Clavien analysis of 3300 patients from 2010 to 2014. *J Clin Neurosci* 2019; **61**: 147-52.
187. Pathai S, Gilbert C, Weiss HA, et al. Frailty in HIV-infected adults in South Africa. *J Acquir Immune Defic Syndr* 2013; **62**(1): 43-51.
188. Pathai S, Lawn SD, Weiss HA, Cook C, Bekker LG, Gilbert CE. Retinal nerve fibre layer thickness and contrast sensitivity in HIV-infected individuals in South Africa: a case-control study. *PLoS One* 2013; **8**(9): e73694.
189. Pathai S, Shiels PG, Weiss HA, et al. Ocular parameters of biological ageing in HIV-infected individuals in South Africa: relationship with chronological age and systemic biomarkers of ageing. *Mech Ageing Dev* 2013; **134**(9): 400-6.
190. Pena FG, Theou O, Wallace L, et al. Comparison of alternate scoring of variables on the performance of the frailty index. *BMC Geriatr* 2014; **14**: 25.

For peer review only

191. Perez Fernandez M, Martinez Miguel P, Ying H, et al. Comorbidity, Frailty, and Waitlist Mortality among Kidney Transplant Candidates of All Ages. *Am J Nephrol* 2019; **49**(2): 103-10.
192. Perez-Zepeda MU, Avila-Funes JA, Gutierrez-Robledo LM, Garcia-Pena C. Frailty Across Age Groups. *J Frailty Aging* 2016; **5**(1): 15-9.
193. Pierce KE, Passias PG, Alas H, et al. Does Patient Frailty Status Influence Recovery Following Spinal Fusion for Adult Spinal Deformity?: An Analysis of Patients With 3-Year Follow-up. *Spine (Phila Pa 1976)* 2020; **45**(7): E397-E405.
194. Piggott DA, Muzaale AD, Varadhan R, et al. Frailty and Cause-Specific Hospitalization Among Persons Aging With HIV Infection and Injection Drug Use. *J Gerontol A Biol Sci Med Sci* 2017; **72**(3): 389-94.
195. Piggott DA, Varadhan R, Mehta SH, et al. Frailty, Inflammation, and Mortality Among Persons Aging With HIV Infection and Injection Drug Use. *J Gerontol A Biol Sci Med Sci* 2015; **70**(12): 1542-7.
196. Prince CS, Noren Hooten N, Mode NA, et al. Frailty in middle age is associated with frailty status and race-specific changes to the transcriptome. *Aging (Albany NY)* 2019; **11**(15): 5518-34.
197. Ramaty E, Maor E, Peltz-Sinvani N, et al. Low ALT blood levels predict long-term all-cause mortality among adults. A historical prospective cohort study. *European Journal of Internal Medicine* 2014; **25**(10): 919-21.
198. Ravindrarajah R, Lee DM, Pye SR, et al. The ability of three different models of frailty to predict all-cause mortality: results from the European Male Aging Study (EMAS). *Arch Gerontol Geriatr* 2013; **57**(3): 360-8.
199. Rees HC, Meister E, Mohler MJ, Klotz SA. HIV-Related Frailty Is Not Characterized by Sarcopenia. *Journal of the International Association of Providers of AIDS Care* 2016; **15**(2): 131-4.
200. Reid DBC, Daniels AH, Ailon T, et al. Frailty and Health-Related Quality of Life Improvement Following Adult Spinal Deformity Surgery. *World Neurosurgery* 2018; **112**: e548-e54.
201. Ribeiro SML, Morley JE, Malmstrom TK, Miller DK. Fruit and vegetable intake and physical activity as predictors of disability risk factors in African-American middle-aged individuals. *J Nutr Health Aging* 2016; **20**(9): 891-6.
202. Richardson AM, McCarthy DJ, hu J, et al. Predictors of Successful Discharge of Patients on Postoperative Day 1 After Craniotomy for Brain Tumor. *World Neurosurgery* 2019; **126**: e869-e77.
203. Rietman ML, Spijkerman AMW, Wong A, et al. Antioxidants linked with physical, cognitive and psychological frailty: Analysis of candidate biomarkers and markers derived from the MARK-AGE study. *Mech Ageing Dev* 2019; **177**: 135-43.
204. Rochira V, Diazzi C, Santi D, et al. Low testosterone is associated with poor health status in men with human immunodeficiency virus infection: a retrospective study. *Andrology* 2015; **3**(2): 298-308.
205. Rockwood K, Song X, Mitnitski A. Changes in relative fitness and frailty across the adult lifespan: evidence from the Canadian National Population Health Survey. *CMAJ Canadian Medical Association Journal* 2011; **183**(8): E487-94.
206. Rockwood MR, MacDonald E, Sutton E, Rockwood K, Baron M, Canadian Scleroderma Research G. Frailty index to measure health status in people with systemic sclerosis. *Journal of Rheumatology* 2014; **41**(4): 698-705.
207. Roshanravan B, Khatri M, Robinson-Cohen C, et al. A prospective study of frailty in nephrology-referred patients with CKD. *American Journal of Kidney Diseases* 2012; **60**(6): 912-21.

For peer review only

208. Rothenberg KA, Stern JR, George EL, et al. Association of Frailty and Postoperative Complications With Unplanned Readmissions After Elective Outpatient Surgery. *JAMA Network Open* 2019; **2**(5): e194330.
209. Rozenberg D, Mathur S, Wickerson L, Chowdhury NA, Singer LG. Frailty and clinical benefits with lung transplantation. *J Heart Lung Transplant* 2018; **37**(10): 1245-53.
210. Rubtsova AA, Marquine MJ, Depp C, et al. Psychosocial Correlates of Frailty Among HIV-Infected and HIV-Uninfected Adults. *Behavioral Medicine* 2018; **45**(3): 210-20.
211. Sabel MS, Lee J, Cai S, Englesbe MJ, Holcombe S, Wang S. Sarcopenia as a prognostic factor among patients with stage III melanoma. *Annals of Surgical Oncology* 2011; **18**(13): 3579-85.
212. Sadiq F, Kronzer VL, Wildes TS, et al. Frailty Phenotypes and Relations With Surgical Outcomes: A Latent Class Analysis. *Anesth Analg* 2018; **127**(4): 1017-27.
213. Sahli Z, Canner JK, Najjar O, et al. Association Between Age and Patient-Reported Changes in Voice and Swallowing After Thyroidectomy. *Laryngoscope* 2019; **129**(2): 519-24.
214. Salem BE. Characterizing Frailty among Homeless Adults: University of California, Los Angeles; 2013.
215. Salem BE, Nyamathi A, Brecht ML, et al. Constructing and identifying predictors of frailty among homeless adults-a latent variable structural equations model approach. *Arch Gerontol Geriatr* 2014; **58**(2): 248-56.
216. Salem BE, Nyamathi AM, Brecht ML, et al. Correlates of frailty among homeless adults. *West J Nurs Res* 2013; **35**(9): 1128-52.
217. Sandkovsky U, Robertson KR, Meza JL, et al. Pilot study of younger and older HIV-infected adults using traditional and novel functional assessments. *HIV Clin Trials* 2013; **14**(4): 165-74.
218. Schaenman J, Castellon L, Liang EC, et al. The Frailty Risk Score predicts length of stay and need for rehospitalization after kidney transplantation in a retrospective cohort: a pilot study. *Pilot Feasibility Stud* 2019; **5**: 144.
219. Schinkel-Ivy A, Mosca I, Mansfield A. Factors Contributing to Unexpected Retirement and Unemployment in Adults Over 50 Years Old in Ireland. *Gerontol Geriatr Med* 2017; **3**: 2333721417722709.
220. Schopmeyer L, El Moumni M, Nieuwenhuijs-Moeke GJ, Berger SP, Bakker SJL, Pol RA. Frailty has a significant influence on postoperative complications after kidney transplantation-a prospective study on short-term outcomes. *Transpl Int* 2019; **32**(1): 66-74.
221. Sharma A, Hoover DR, Shi Q, et al. Frailty as a predictor of falls in HIV-infected and uninfected women. *Antivir Ther* 2019; **24**(1): 51-61.
222. Sharma A, Shi Q, Hoover DR, et al. Frailty predicts fractures among women with and at-risk for HIV. *AIDS* 2019; **33**(3): 455-63.
223. Shin JI, Kothari P, Phan K, et al. Frailty Index as a Predictor of Adverse Postoperative Outcomes in Patients Undergoing Cervical Spinal Fusion. *Spine (Phila Pa 1976)* 2017; **42**(5): 304-10.
224. Singer JP, Diamond JM, Anderson MR, et al. Frailty phenotypes and mortality after lung transplantation: A prospective cohort study. *Am J Transplant* 2018; **18**(8): 1995-2004.
225. Singer JP, Diamond JM, Gries CJ, et al. Frailty Phenotypes, Disability, and Outcomes in Adult Candidates for Lung Transplantation. *Am J Respir Crit Care Med* 2015; **192**(11): 1325-34.
226. Smit E, Wanke C, Dong K, et al. Frailty, Food Insecurity, and Nutritional Status in People Living with Hiv. *J Frailty Aging* 2015; **4**(4): 191-7.

For peer review only

227. Smitherman AB, Anderson C, Lund JL, Bensen JT, Rosenstein DL, Nichols HB. Frailty and Comorbidities Among Survivors of Adolescent and Young Adult Cancer: A Cross-Sectional Examination of a Hospital-Based Survivorship Cohort. *J Adolesc Young Adult Oncol* 2018; **7**(3): 374-83.
228. Soma O, Hatakeyama S, Okamoto T, et al. Clinical implication of a quantitative frailty assessment tool for prognosis in patients with urological cancers. *Oncotarget* 2018; **9**(25): 17396-405.
229. Stanjek-Cichoracka A, Wozniak-Grygiel E, Laszewska A, Zembala M, Ochman M. Assessment of Cytokines, Biochemical Markers of Malnutrition and Frailty Syndrome Patients Considered for Lung Transplantation. *Transplant Proc* 2019; **51**(6): 2009-13.
230. Stenholm S, Strandberg TE, Pitkala K, Sainio P, Heliovaara M, Koskinen S. Midlife obesity and risk of frailty in old age during a 22-year follow-up in men and women: the Mini-Finland Follow-up Survey. *J Gerontol A Biol Sci Med Sci* 2014; **69**(1): 73-8.
231. Subramaniam S, Aalberg JJ, Soriano RP, Divino CM. New 5-Factor Modified Frailty Index Using American College of Surgeons NSQIP Data. *J Am Coll Surg* 2018; **226**(2): 173-81 e8.
232. Susanto M, Hubbard RE, Gardiner PA. Association of 12-Year Trajectories of Sitting Time With Frailty in Middle-Aged Women. *Am J Epidemiol* 2018; **187**(11): 2387-96.
233. Susanto M, Hubbard RE, Gardiner PA. Validity and Responsiveness of the FRAIL Scale in Middle-Aged Women. *J Am Med Dir Assoc* 2018; **19**(1): 65-9.
234. Swiecicka A, Lunt M, Ahern T, et al. Nonandrogenic Anabolic Hormones Predict Risk of Frailty: European Male Ageing Study Prospective Data. *J Clin Endocrinol Metab* 2017; **102**(8): 2798-806.
235. Sy J, McCulloch CE, Johansen KL. Depressive symptoms, frailty, and mortality among dialysis patients. *Hemodial Int* 2019; **23**(2): 239-46.
236. Sy J, Streja E, Grimes B, Johansen KL. The Marginal Cost of Frailty Among Medicare Patients on Hemodialysis. *Kidney Int Rep* 2020; **5**(3): 289-95.
237. Tajar A, O'Connell MD, Mitnitski AB, et al. Frailty in relation to variations in hormone levels of the hypothalamic-pituitary-testicular axis in older men: results from the European male aging study. *J Am Geriatr Soc* 2011; **59**(5): 814-21.
238. Tapper EB, Derstine B, Baki J, Su GL. Bedside Measures of Frailty and Cognitive Function Correlate with Sarcopenia in Patients with Cirrhosis. *Dig Dis Sci* 2019; **64**(12): 3652-9.
239. Tapper EB, Konerman M, Murphy S, Sonnenday CJ. Hepatic encephalopathy impacts the predictive value of the Fried Frailty Index. *Am J Transplant* 2018; **18**(10): 2566-70.
240. Telemi E, Trofymenko O, Venkat R, Pandit V, Pandian TK, Nfonsam VN. Frailty Predicts Morbidity after Colectomy for Ulcerative Colitis. *Am Surg* 2018; **84**(2): 225-9.
241. Traven SA, Horn RW, Reeves RA, Walton ZJ, Woolf SK, Slone HS. The 5-Factor Modified Frailty Index Predicts Complications, Hospital Admission, and Mortality Following Arthroscopic Rotator Cuff Repair. *Arthroscopy* 2020; **36**(2): 383-8.
242. Trung TN, Duoc NVT, Nhat LTH, et al. Functional outcome and muscle wasting in adults with tetanus. *Trans R Soc Trop Med Hyg* 2019; **113**(11): 706-13.
243. Tuttle LJ, Bittel DC, Bittel AJ, Sinacore DR. Early-Onset Physical Frailty in Adults With Diabesity and Peripheral Neuropathy. *Can J Diabetes* 2018; **42**(5): 478-83.
244. Van Pilsum Rasmussen S, Konel J, Warsame F, et al. Engaging clinicians and patients to assess and improve frailty measurement in adults with end stage renal disease. *BMC Nephrol* 2018; **19**(1): 8.

For peer review only

245. Vatanen A, Hou M, Huang T, et al. Clinical and biological markers of premature aging after autologous SCT in childhood cancer. *Bone Marrow Transplant* 2017; **52**(4): 600-5.
246. Venado A, McCulloch C, Greenland JR, et al. Frailty trajectories in adult lung transplantation: A cohort study. *J Heart Lung Transplant* 2019; **38**(7): 699-707.
247. Verheij E, Kirk GD, Wit FW. Frailty is associated with mortality and incident comorbidity among middle-aged HIV-positive and HIV-negative participants. *Care Management* 2020; **26**(1): 22-.
248. Verschoor CP, Tamim H. Frailty Is Inversely Related to Age at Menopause and Elevated in Women Who Have Had a Hysterectomy: An Analysis of the Canadian Longitudinal Study on Aging. *J Gerontol A Biol Sci Med Sci* 2019; **74**(5): 675-82.
249. Wade KF, Lee DM, McBeth J, et al. Chronic widespread pain is associated with worsening frailty in European men. *Age Ageing* 2016; **45**(2): 268-74.
250. Wallace LM, Theou O, Kirkland SA, et al. Accumulation of non-traditional risk factors for coronary heart disease is associated with incident coronary heart disease hospitalization and death. *PLoS One* 2014; **9**(3): e90475.
251. Wang CW, Lebsack A, Chau S, Lai JC. The Range and Reproducibility of the Liver Frailty Index. *Liver Transpl* 2019; **25**(6): 841-7.
252. Wang R, Shlipak MG, Ix JH, et al. Association of Fibroblast Growth Factor-23 (FGF-23) With Incident Frailty in HIV-Infected and HIV-Uninfected Individuals. *J Acquir Immune Defic Syndr* 2019; **80**(1): 118-25.
253. Warsame F, Ying H, Haugen CE, et al. Intradialytic Activities and Health-Related Quality of Life Among Hemodialysis Patients. *Am J Nephrol* 2018; **48**(3): 181-9.
254. Wen Y, Jabir MA, Dosokey EM, et al. Using Modified Frailty Index to Predict Safe Discharge Within 48 Hours of Ileostomy Closure. *Dis Colon Rectum* 2017; **60**(1): 76-80.
255. Wilkes JG, Evans JL, Prato BS, Hess SA, MacGillivray DC, Fitzgerald TL. Frailty Cost: Economic Impact of Frailty in the Elective Surgical Patient. *J Am Coll Surg* 2019; **228**(6): 861-70.
256. Williams DM, Jylhava J, Pedersen NL, Hagg S. A Frailty Index for UK Biobank Participants. *J Gerontol A Biol Sci Med Sci* 2019; **74**(4): 582-7.
257. Wilson CL, Chemaitilly W, Jones KE, et al. Modifiable Factors Associated With Aging Phenotypes Among Adult Survivors of Childhood Acute Lymphoblastic Leukemia. *J Clin Oncol* 2016; **34**(21): 2509-15.
258. Wilson JM, Holzgrefe RE, Staley CA, Schenker ML, Meals C. The Effect of Malnutrition on Postoperative Complications Following Surgery for Distal Radius Fractures. *J Hand Surg Am* 2019; **44**(9): 742-50.
259. Wilson JM, Lunati MP, Grabel ZJ, Staley CA, Schwartz AM, Schenker ML. Hypoalbuminemia Is an Independent Risk Factor for 30-Day Mortality, Postoperative Complications, Readmission, and Reoperation in the Operative Lower Extremity Orthopaedic Trauma Patient. *J Orthop Trauma* 2019; **33**(6): 284-91.
260. Wilson ME, Vakil AP, Kandel P, Undavalli C, Dunlay SM, Kennedy CC. Pretransplant frailty is associated with decreased survival after lung transplantation. *J Heart Lung Transplant* 2016; **35**(2): 173-8.
261. Womack JA, Goulet JL, Gibert C, et al. Physiologic frailty and fragility fracture in HIV-infected male veterans. *Clin Infect Dis* 2013; **56**(10): 1498-504.
262. Wulunggono W, Yunihastuti E, Shatri H, Wahyudi ER, Ophinni Y. Frailty among HIV-1 Infected Adults under Antiretroviral Therapy in Indonesia. *Curr HIV Res* 2019; **17**(3): 204-13.

For peer review only

263. Xu W, Zhang XM, Ke T, Cai HR, Gao X. Modified Frailty Index and Body Mass Index as Predictors of Adverse Surgical Outcomes in Degenerative Spinal Disease. *Turk Neurosurg* 2018; **28**(6): 897-903.

264. Yadla M, John JP, Mummadi M. A study of clinical assessment of frailty in patients on maintenance hemodialysis supported by cashless government scheme. *Saudi J Kidney Dis Transpl* 2017; **28**(1): 15-22.

265. Yagi M, Michikawa T, Hosogane N, et al. Treatment for Frailty Does Not Improve Complication Rates in Corrective Surgery for Adult Spinal Deformity. *Spine (Phila Pa 1976)* 2019; **44**(10): 723-31.

266. Yagi M, Michikawa T, Hosogane N, et al. The 5-Item Modified Frailty Index Is Predictive of Severe Adverse Events in Patients Undergoing Surgery for Adult Spinal Deformity. *Spine (Phila Pa 1976)* 2019; **44**(18): E1083-E91.

267. Yi C, Lin J, Cao P, et al. Prevalence and Prognosis of Coexisting Frailty and Cognitive Impairment in Patients on Continuous Ambulatory Peritoneal Dialysis. *Sci Rep* 2018; **8**(1): 17305.

268. Young AC, Glaser K, Spector TD, Steves CJ. The Identification of Hereditary and Environmental Determinants of Frailty in a Cohort of UK Twins. *Twin Res Hum Genet* 2016; **19**(6): 600-9.

For peer review only

PRISMA 2009 Checklist

Section/topic	#	Checklist item	Reported on page #
**TITLE**			
Title	1	Identify the report as a systematic review, meta-analysis, or both.	1
**ABSTRACT**			
Structured summary	2	Provide a structured summary including, as applicable: background; objectives; data sources; study eligibility criteria, participants, and interventions; study appraisal and synthesis methods; results; limitations; conclusions and implications of key findings; systematic review registration number.	2
**INTRODUCTION**			
Rationale	3	Describe the rationale for the review in the context of what is already known.	3-4
Objectives	4	Provide an explicit statement of questions being addressed with reference to participants, interventions, comparisons, outcomes, and study design (PICOS).	4
**METHODS**			
Protocol and registration	5	Indicate if a review protocol exists, if and where it can be accessed (e.g., Web address), and, if available, provide registration information including registration number.	NA
Eligibility criteria	6	Specify study characteristics (e.g., PICOS, length of follow-up) and report characteristics (e.g., years considered, language, publication status) used as criteria for eligibility, giving rationale.	5-6
Information sources	7	Describe all information sources (e.g., databases with dates of coverage, contact with study authors to identify additional studies) in the search and date last searched.	5
Search	8	Present full electronic search strategy for at least one database, including any limits used, such that it could be repeated.	Supplementary materials
Study selection	9	State the process for selecting studies (i.e., screening, eligibility, included in systematic review, and, if applicable, included in the meta-analysis).	6
Data collection process	10	Describe method of data extraction from reports (e.g., piloted forms, independently, in duplicate) and any processes for obtaining and confirming data from investigators.	6-7
Data items	11	List and define all variables for which data were sought (e.g., PICOS, funding sources) and any assumptions and simplifications made.	7
Risk of bias in individual studies	12	Describe methods used for assessing risk of bias of individual studies (including specification of whether this was done at the study or outcome level), and how this information is to be used in any data synthesis.	NA
Summary measures	13	State the principal summary measures (e.g., risk ratio, difference in means).	NA
Synthesis of results	14	Describe the methods of handling data and combining results of studies, if done, including measures of consistency (e.g., I^2) for each meta-analysis.	NA

For peer review only - http://bmjopen.bmj.com/site/about/guidelines.xhtml

PRISMA 2009 Checklist

Section/topic	#	Checklist item	Reported on page #
Risk of bias across studies	15	Specify any assessment of risk of bias that may affect the cumulative evidence (e.g., publication bias, selective reporting within studies).	NA
Additional analyses	16	Describe methods of additional analyses (e.g., sensitivity or subgroup analyses, meta-regression), if done, indicating which were pre-specified.	NA
**RESULTS**			
Study selection	17	Give numbers of studies screened, assessed for eligibility, and included in the review, with reasons for exclusions at each stage, ideally with a flow diagram.	8, and figure 1
Study characteristics	18	For each study, present characteristics for which data were extracted (e.g., study size, PICOS, follow-up period) and provide the citations.	Supplementary materials table S2
Risk of bias within studies	19	Present data on risk of bias of each study and, if available, any outcome level assessment (see item 12).	NA
Results of individual studies	20	For all outcomes considered (benefits or harms), present, for each study: (a) simple summary data for each intervention group (b) effect estimates and confidence intervals, ideally with a forest plot.	NA
Synthesis of results	21	Present results of each meta-analysis done, including confidence intervals and measures of consistency.	9-11, supplementary materials tables S3, S4, and figures S1 and S2
Risk of bias across studies	22	Present results of any assessment of risk of bias across studies (see Item 15).	NA
Additional analysis	23	Give results of additional analyses, if done (e.g., sensitivity or subgroup analyses, meta-regression [see Item 16]).	NA
**DISCUSSION**			
Summary of evidence	24	Summarize the main findings including the strength of evidence for each main outcome; consider their relevance to key groups (e.g., healthcare providers, users, and policy makers).	11-12
Limitations	25	Discuss limitations at study and outcome level (e.g., risk of bias), and at review-level (e.g., incomplete retrieval of identified research, reporting bias).	12-13
Conclusions	26	Provide a general interpretation of the results in the context of other evidence, and implications for future research.	11-12
**FUNDING**			

For peer review only - http://bmjopen.bmj.com/site/about/guidelines.xhtml

PRISMA 2009 Checklist

Funding	27	Describe sources of funding for the systematic review and other support (e.g., supply of data); role of funders for the systematic review.	Title page

From: Moher D, Liberati A, Tetzlaff J, Altman DG, The PRISMA Group (2009). Preferred Reporting Items for Systematic Reviews and Meta-Analyses: The PRISMA Statement. PLoS Med 6(7): e1000097. doi:10.1371/journal.pmed1000097

For more information, visit: www.prisma-statement.org.

Page 2 of 2

For peer review only

Measuring frailty in younger populations: a rapid review of evidence

Journal:	*BMJ Open*
Manuscript ID	bmjopen-2020-047051.R1
Article Type:	Original research
Date Submitted by the Author:	11-Feb-2021

Complete List of Authors:	Spiers, Gemma; Newcastle University, Population & Health Sciences Institute<br>Kunonga, Tafadzwa; Newcastle University, Population & Health Sciences Institute<br>Hall, Alex; University of Manchester, Division of Nursing, Midwifery & Social Work; School of Health Sciences<br>Beyer, Fiona ; Newcastle University,<br>Boulton, Elisabeth; University of Manchester, School of Health Sciences; Manchester Academic Health Science Centre,<br>Parker, Stuart; Newcastle University, Institute of Health and Society / Newcastle University Institute for Ageing<br>Bower, Peter; University of Manchester, NPCRDC<br>Craig, Dawn; Institute of Health & Society, Newcastle University, Health Economics Group, Institute of Health and Society<br>Todd, Chris; University of Manchester, School of Health Sciences<br>Hanratty, Barbara; Newcastle University, Institute of Health and Society / Newcastle University Institute for Ageing

**Primary Subject Heading**:	Health policy
Secondary Subject Heading:	Research methods
Keywords:	GENERAL MEDICINE (see Internal Medicine), PRIMARY CARE, Health policy < HEALTH SERVICES ADMINISTRATION & MANAGEMENT

SCHOLARONE™ Manuscripts

I, the Submitting Author has the right to grant and does grant on behalf of all authors of the Work (as defined in the below author licence), an exclusive licence and/or a non-exclusive licence for contributions from authors who are: i) UK Crown employees; ii) where BMJ has agreed a CC-BY licence shall apply, and/or iii) in accordance with the terms applicable for US Federal Government officers or employees acting as part of their official duties; on a worldwide, perpetual, irrevocable, royalty-free basis to BMJ Publishing Group Ltd ("BMJ") its licensees and where the relevant Journal is co-owned by BMJ to the co-owners of the Journal, to publish the Work in this journal and any other BMJ products and to exploit all rights, as set out in our licence.

The Submitting Author accepts and understands that any supply made under these terms is made by BMJ to the Submitting Author unless you are acting as an employee on behalf of your employer or a postgraduate student of an affiliated institution which is paying any applicable article publishing charge ("APC") for Open Access articles. Where the Submitting Author wishes to make the Work available on an Open Access basis (and intends to pay the relevant APC), the terms of reuse of such Open Access shall be governed by a Creative Commons licence – details of these licences and which Creative Commons licence will apply to this Work are set out in our licence referred to above.

Other than as permitted in any relevant BMJ Author's Self Archiving Policies, I confirm this Work has not been accepted for publication elsewhere, is not being considered for publication elsewhere and does not duplicate material already published. I confirm all authors consent to publication of this Work and authorise the granting of this licence.

For peer review only

Measuring frailty in younger populations: a rapid review of evidence

Dr Gemma Spiers[a*] gemma-frances.spiers@newcastle.ac.uk
Ms Tafadzwa Patience Kunonga[a]
Dr Alex Hall [b]
Ms Fiona Beyer [a]
Dr Elisabeth Boulton [b]
Professor Stuart Parker [a]
Professor Peter Bower [b]
Professor Dawn Craig [a]
Professor Chris Todd [b]
Professor Barbara Hanratty[a]

a. National Institute for Health Research (NIHR) Older People and Frailty Policy Research Unit, Population Health Sciences Institute, Newcastle University, Newcastle-upon-Tyne, NE4 5PL, UK.

b. National Institute for Health Research (NIHR) Older People and Frailty Policy Research Unit, School of Health Sciences, Faculty of Biology, Medicine and Health, The University of Manchester, Manchester, M13 9PL, UK.

*Corresponding author

Funding & Acknowledgements

This report presents independent research funded by the National Institute for Health Research Policy Research Unit in Older People and Frailty (PR-PRU-1217-21502). The views expressed are those of the authors and not necessarily those of the NIHR or the Department of Health and Social Care.

Conflict of interest

No conflicts of interest.

Data statement

All data relevant to the study are included in the article or uploaded as supplementary information

Keywords: Frailty, younger populations, measurement

Words (excluding abstract, tables, figures and references): 4137
Tables and figures: 3 (additional tables and figures in supplementary materials)

Author contributions

GS co-designed the protocol, undertook all stages of the review, and co-wrote the paper. PK co-designed the protocol, undertook all stages of the review and co-wrote the paper. AH co-designed the protocol, undertook all stages of the review, and co-wrote the paper. FB co-designed the protocol, undertook the searches for the review and co-wrote the paper. EB co-designed the protocol and co-wrote the paper. SP co-designed the protocol and co-wrote

For peer review only - http://bmjopen.bmj.com/site/about/guidelines.xhtml

the paper. PB co-designed the protocol, contributed to the analysis of review data, and co-wrote the paper. DC co-designed the protocol, contributed to the analysis of review data, and co-wrote the paper. CT co-designed the protocol, and co-wrote the paper. BH co-designed the protocol, and co-wrote the paper.

For peer review only

For peer review only - http://bmjopen.bmj.com/site/about/guidelines.xhtml

Measuring frailty in younger populations: a rapid review of evidence

Objectives

Frailty is typically assessed in older populations. Identifying frailty in adults aged under 60 years may also have value, if it supports the delivery of timely care. We sought to identify how frailty is measured in younger populations, including evidence of the impact on patient outcomes and care.

Design

A rapid review of primary studies was conducted.

Data sources

Four databases, three sources of grey literature and reference lists of systematic reviews were searched, in March 2020.

Eligibility criteria

Eligible studies measured frailty in populations aged under 60 years using experimental or observational designs, published after 2000 in English.

Data extraction and synthesis

Records were screened against review criteria. Study data were extracted with 20% of records checked for accuracy by a second researcher. Data were synthesised using a narrative approach.

Results

We identified 268 studies that measured frailty in samples that included people aged under 60 years. Of these, 85 studies reported evidence about measure validity. No measures were identified that were designed and validated to identify frailty *exclusively* in younger groups. However, in populations that included people aged over *and* under 60 years, cumulative deficit frailty indices, phenotype measures, the FRAIL scale, the Liver Frailty Index, and the Short Physical Performance Battery all demonstrated predictive validity for mortality and/or hospital admission. Evidence of criterion validity was rare. The extent to which measures possess validity across the younger adult age (18 to 59) spectrum was unclear. There was no evidence about the impact of measuring frailty in younger populations on patient outcomes and care.

Conclusions

Limited evidence suggests that frailty measures have predictive validity in younger populations. Further research is needed to clarify the validity of measures across the adult age spectrum, and explore the utility of measuring frailty in younger groups.

For peer review only - http://bmjopen.bmj.com/site/about/guidelines.xhtml

Strengths and limitations of this study

- Comprehensive and systematic searches captured the core evidence about measuring frailty in younger populations
- We were only able to judge predictive validity based on frailty outcomes for older populations and not younger populations
- The focus on younger groups offers a novel addition to our understanding of frailty measurement
- Our exclusion of studies with samples aged, on average, over 60 years means we have omitted some studies that include younger participants.

For peer review only

For peer review only - http://bmjopen.bmj.com/site/about/guidelines.xhtml

Background

Frailty is characterised by increased vulnerability to stressors, and has been described as a problematic expression of population ageing.[1, 2] The presence of frailty is associated with a number of adverse outcomes, including lower quality of life and an increased risk of functional decline, admission to hospital or long-term care and mortality.[3-6] Recent estimates place the global incidence of frailty at 43.4 cases per 1000 person-years, although there is substantial variation by country-level income and disease specific populations.[7]

Debates about how to measure and operationalise frailty have led to two dominant models: the phenotype model and the cumulative deficit model. The phenotype model, which was conceptualised through clinical observation and epidemiological study, operationalises frailty as the presence of three or more of the following criteria: exhaustion, weight loss, weakness/loss of muscular strength, reduced gait speed, and reduced energy/physical activity.[8] In contrast, the cumulative deficit model, which was developed through consideration of biological theories of ageing,[9] considers frailty an accumulation of deficits including clinical signs and symptoms, diseases, and disability. In this model, 'the more things somebody has wrong with them, the more likely they are to be frail'.[10] (p.722)

There are numerous tools to measure frailty, which are not necessarily interchangeable.[11] A recent review of measures in older populations identified 51 separate instruments.[12] Although many measures of frailty are informed by the cumulative deficit or phenotype models, there is still much variation in the content of these instruments.[11] In one analysis of 35 frailty measures, there were varying levels of agreement for identifying who was frail between tools.[13] There are further challenges in assessing the risk of frailty in clinical settings, where different approaches exist across primary, secondary and specialist care.[2] For example, the Clinical Frailty Scale, gait speed, PRISMA-7 and timed-up-and-go tests are all validated tools to assess the risk of frailty in older populations in clinical care.[14-16] These numerous instruments present challenges to researchers and clinicians when choosing how to conceptualise, measure and assess frailty.[12]

The prevalence of frailty increases with age and so it is typically assessed in older populations. In 2019, the International Conference of Frailty and Sarcopenia Research Task Force recommended routine frailty screening in populations aged over 65 years.[17] This reflects current clinical guidance in the UK for example, where screening for frailty in populations aged over 65 is routine in primary care, and also forms part of the Comprehensive Geriatric Assessment in secondary care settings.[15] Such routine screening is part of efforts to support people as they age.

For peer review only - http://bmjopen.bmj.com/site/about/guidelines.xhtml

The utility of identifying frailty in adults aged under 60 years also warrants scrutiny. Some vulnerable groups with chronic conditions may be at risk of becoming frail earlier in life, whilst the onset of illness and disability occurs at younger ages for people living in areas of greater deprivation. [18-20] There is, therefore, potential for younger groups to be classed as frail, and identification may aid the delivery of timely care. Furthermore, studies are increasingly measuring frailty prevalence across the life span.[21-23] Yet current debates and evidence about how to measure frailty remain focused on older populations. A number of systematic reviews have examined the validity of various frailty measures, most recently in 2019.[24-26] These reviews focus on the measurement of frailty in older populations, and there remains a lack of clarity about the validity of frailty measures in younger groups.

To address this gap, we aimed to synthesise evidence on how frailty is measured in younger populations (aged < 60 years). Specifically, we sought evidence to address the following questions:

a. Are there any validated tools to identify frailty specifically in younger populations?
b. How are existing tools to identify frailty in older populations used to measure and identify frailty in younger age groups, and are such tools validated in these younger populations?
c. How is frailty identified in younger people with long term or life limiting conditions?
d. In what other ways (other than a validated tool) is frailty in younger populations currently operationalised and identified from existing data in observational and experimental studies?
e. Is there any evidence of impact (on patient outcomes, staff, clinical management or health service utilisation) of identifying frailty in younger populations, including identification of opportunities for intervention?

Methods

We conducted a rapid review of primary studies. Rapid reviews offer a streamlined version of standard systematic review methods in a shorter timeframe, with the resultant output a summary, rather than in-depth synthesis, of evidence.[27] There is no agreed methodology for the conduct of rapid reviews, but for the purposes of this work, rapid methods included: systematic searches, double screening of titles and abstracts for 5% of studies to pilot the eligibility criteria; single researcher screening of full texts; second checking of data extraction for 20% of studies; and,

For peer review only - http://bmjopen.bmj.com/site/about/guidelines.xhtml

omitting an assessment of study quality, an approach that has been used in other reviews of frailty measures.[25]

Search strategy

A search strategy was developed, piloted and tested, based on three concepts: frailty, measurement and age (see supplementary materials for the strategy applied to MEDLINE). The search strategy excluded studies that were indexed with terms for older people, unless the record also contained indexing terms pertaining to a younger population (‘middle aged’ or ‘young adult’).

Searches were carried out in: MEDLINE (OVID) (1946 to March week 1 2020); PsycINFO (OVID) (1967 to March week 2 2020); CINAHL (EBSCO) (1982 to March 2020); Science Citation Index (Web of Knowledge) (1970 to March 2020); Social Science Citation (Web of Knowledge) (1970 to March 2020). Three sources of grey literature were also searched: OpenGrey,[28] Social Care Online,[29] and the National Institute of Health and Care Excellence.[30] References of relevant systematic reviews were also checked. Searches were carried out in March 2020.

Review criteria

Eligible for inclusion were primary research studies that measured frailty in populations aged under 60 years, and published after 2000 in English, using experimental or observational study designs (table 1). The age threshold of below 60 years was selected, as opposed to 65 years, as initial scoping indicated that studies on frailty in older populations typically used samples aged 60 years and over. Studies that included those aged 60 years and over were included only if the majority of sample was aged under 60 years. Where it was not possible to determine this majority, studies were included if the mean or median sample age was between 18 and 59.9 years. As the concept of frailty began to develop from around 2001,[31] we excluded studies before 2000 to prioritise the most relevant and contemporary evidence.

For peer review only - http://bmjopen.bmj.com/site/about/guidelines.xhtml

Table 1. Review inclusion/exclusion criteria

	Include	Exclude
**Population**	Adults aged 18-59.9 years.<br>Studies that include those aged ≥60 years if the majority of sample is aged under 60 years. Where it is not possible to determine if the majority of the sample are aged under 60 years, studies where the mean/median sample age was under 60 years were included.<br><br>Any condition/diagnosis.	Studies of people aged ≥60 years only, or where the majority of the study sample is aged >60 years.
**Exposure/outcome**	Any measure of frailty that is validated, including those currently used in 65+ populations and those developed specifically for younger age groups.<br><br>Any approach to identifying frailty from existing data.<br><br>Frailty may be either the exposure (e.g. if using it to predict other outcomes) or the outcome (e.g. if estimating prevalence).	
**Study design**	Observational or experimental designs.<br><br>Published in English after 2000.	

Study selection

Title and abstract screening was piloted by three researchers (AH, PK, GS) independently on 5% of records. Decisions were compared and discussed to resolve inconsistencies before proceeding. The titles and abstracts of the remaining 95% of records were then screened by single researchers (AH, PK, GS). The full texts of selected records were retrieved and assessed for inclusion against the review criteria. For studies that reported details of a validated frailty measure, the referenced publication was also retrieved and assessed against the review criteria.

Data extraction and synthesis

A data extraction form was developed and piloted. Summary study information was extracted: author and date; country; measure used; sample age (mean, median and range where reported); and long-term condition of sample (if applicable). Included studies were further scrutinised to identify those that reported evidence that indicated whether the measure was validated. To ascertain validity, we adapted criteria used in a previous review of frailty measures in older populations.[32] That is, the validity of a frailty measure was ascertained through evidence that:

For peer review only - http://bmjopen.bmj.com/site/about/guidelines.xhtml

i. the measure predicted mortality and/or hospitalisations (*predictive validity*)

ii. the measure was associated with another validated measure of frailty (*criterion validity*)

Predictive validity indicates whether the measure can predict outcomes of relevance. Mortality and hospital admissions were chosen as outcomes for the test of predictive validity as these are commonly associated with frailty.[33-36] Criterion validity indicates whether the new measure is assessing what it intends to, by comparing it to a validated measure of the same concept.[32, 37] This is achieved by assessing the association between the two measures (e.g. correlation or regression), or the extent to which two measures produce the same results (agreement).

For studies that reported evidence about predictive and/or criterion validity, data were extracted about: the measure (e.g. items, cut off scores used); whether the evidence indicated validity, including prediction estimates; and any evidence about the impact of measuring frailty in younger populations on patient outcomes, staff, clinical management or health service utilisation (e.g. if a measure was trialled or evaluated). Data were extracted by single researchers (AH, PK, GS), with 20% checked by a second for accuracy.

A narrative synthesis was used to summarise what measures of frailty were used and which measures demonstrated evidence of validity (in which populations). To facilitate this, each study that reported evidence about validity was allocated to a colour coded framework (supplementary materials table S1). Studies coded green demonstrated evidence of validity (a statistically significant prediction of mortality and/or hospital admissions, or authors described associations or agreement between measures as good or strong). Studies coded amber reported inconsistent or unclear evidence of validity, or authors described associations or agreement between measures as fair or modest. Studies coded red demonstrated no evidence of validity (the prediction of mortality and/or hospital admissions was not statistically significant, authors described associations or agreement between measures as weak or poor, or data were not reported in the publication to verify a claim of an association). Finally, we summarised evidence about any reported impact of measuring frailty in younger populations on patient outcomes and care.

Patient and public involvement

This work was a rapid response to a request by the UK Department of Health and Social Care with a limited timeframe available for completion. Involving patients effectively in such rapid responsive work can be challenging, and in this case we did not feel that we could effectively engage with

For peer review only - http://bmjopen.bmj.com/site/about/guidelines.xhtml

patients in the time available. However, the work was undertaken in response to a policy imperative.

Results

After screening, 268 publications met the review criteria (figure 1; full bibliographic details of the included studies are also listed in the supplementary materials).

Figure 1 here

Most studies used samples aged under *and* over 60 years. A minority of studies (n=6) used samples aged entirely below 60 years.[38-43] Just over 70% of studies used study samples with an average age between 51 and 59 years, whilst 24.1% used samples with an average age of between 41 to 50 years. Few studies measured frailty in samples with an average age below 40 years (figure 2). Thus, when referring to *younger populations* in these findings, this typically refers to the older end of the 18 to 59 year spectrum.

Figure 2 here

Are there any validated tools to identify frailty specifically in younger populations?

No measures were identified that were designed and validated to identify frailty *exclusively* in younger (18 to 59.9 years) populations.

How are existing tools to identify frailty in older populations used to measure and identify frailty in younger age groups, and are such tools validated in these younger populations?

Across studies, 41 measures of frailty were identified (supplementary materials table S2). The most common were phenotype measures or cumulative deficit frailty indices. Some studies measured frailty using single, or a combination of, indicators, such as sarcopenia with vascular disease, or muscle mass. A small number of studies claimed to measure frailty but used tools not designed for this purpose (the Katz Index, the ADL Scale, the Karnofsky Performance Scale, and the Braden Scale). The studies reporting these tools were therefore not included in this review.

A subset of 85 studies, of the 268 that met the review criteria, reported evidence about the predictive and/or criterion validity of their chosen frailty measure (supplementary materials table S3).[21-23, 44-125]

Across these studies, 13 measures were used (32 in total when accounting for different versions of cumulative deficit frailty indices). These measures included cumulative deficit frailty indices, phenotype measures, the FRAIL Scale, the Short Physical Performance Battery, the Clinical Frailty

For peer review only - http://bmjopen.bmj.com/site/about/guidelines.xhtml

Scale, the Liver Frailty Index, the John Hopkins Frailty Indicator, and the SOF Frailty Scale and CHS Frailty Scale.[21-23, 44-95, 97-125] Although termed a 'Liver Frailty Index', this measure did not comprise items that were specific to liver disease. Three measures were individual markers of frailty (muscle mass, walk test and hand grip), each used in isolation and not as part of a multiple item measure.[57, 60, 70, 83] One measure was a surgical risk index, which incorporated a frailty assessment.[96]

Supplementary materials table S3 summarises the evidence for each study. Evidence of criterion validity, where a measure was compared to a 'gold standard' validated frailty measure, was sparse. Most studies reported evidence about the association between the measure of frailty and mortality and/or hospital admissions, which we used to infer predictive validity.

There was evidence of predictive validity for cumulative deficit frailty indices and phenotype measures in general populations, diagnostic specific populations (HIV, systemic lupus erythematosus, metabolic syndrome, survivors of myocardial infarction, childhood survivors of cancer, chronic kidney or end stage renal disease) and surgical populations (hematopoietic cell transplant, or other non-specified surgery).[22, 23, 44, 47, 52, 53, 56, 59, 63, 66-68, 73, 77, 78, 81, 82, 84, 89-94, 100, 102, 106, 111, 113, 115-117, 119, 122, 124-126] For some populations, evidence of predictive validity was inconsistent for a cumulative deficit frailty index (intellectual disability),[95, 106] and phenotype measures (heart failure and those undergoing lung or kidney transplant).[74, 75, 79, 97-99, 107, 109, 118, 120, 121]

There was also evidence of predictive validity for the FRAIL scale (general and diabetic populations),[54, 55, 65, 94, 110, 117, 123] the Liver Frailty Index (liver disease),[69, 85, 86, 88] and the Short Physical Performance Battery (populations undergoing lung or kidney transplants and those with end stage liver disease).[58, 87, 112, 120, 121]

For the remaining measures, there was either no evidence of predictive validity, or evidence was too inconsistent or uncertain to draw a conclusion.

In terms of whether the validity of a measure varied by age, only two studies stratified their analysis of prediction by age group. One study showed that a cumulative deficit frailty index based exclusively on laboratory test-based indicators of illness predicted mortality only in groups aged 40 to 65 and not those aged 20 to 39 years. In the same study, a cumulative deficit frailty index based entirely on self-reported items predicted mortality in both age groups, but prediction was statistically less certain for those aged 20 to 39 years.[21] In the second study, higher levels of frailty predicted mortality at all ages, but for the younger group (<39 years), differences in survival were only statistically significant between the least and most frail. We further explored the role of age by comparing evidence across studies according to the mean or median sample age (where this was

For peer review only - http://bmjopen.bmj.com/site/about/guidelines.xhtml

reported). We used this approach for the two most commonly used measures: the frailty indices and the phenotype measures (figures 3a and 3b). With very few studies involving participants with an average age below 40 years, there was insufficient evidence to assess how the validity of these measures varied according to the average age of the sample in which the measure was tested.

Figures 3 a and 3b here

How is frailty identified in younger people with long term or life limiting conditions?

Most studies used frailty measures in a clinical sub-group and *not* a generic population (Supplementary materials Table S3). Younger populations of people with life limiting or long-term conditions, in which frailty measures demonstrated evidence of predictive validity, included: HIV (Frailty Index); chronic or end stage kidney disease (Frailty Index, phenotype model, the Short Physical Performance Battery); diabetes (FRAIL Scale); and end stage liver disease (Liver Frailty Index, the Short Physical Performance Battery).

In what other ways (other than a validated tool) is frailty in younger populations currently operationalised and identified from existing data in observational and experimental studies?

Typically, studies operationalised and measured frailty using existing tools, the majority of which were cumulative deficit frailty indices or phenotype measures. In four studies, frailty was identified from single indicators (a walk test and grip strength, one study each, and muscle mass, two studies), which were not part of a multiple item measure.[127]

Is there any evidence of impact of measuring frailty in younger populations?

No studies were identified that evaluated the impact of measuring frailty in younger populations on patient outcomes, staff, clinical care, or service utilisation.

Discussion

This review identified no measures that were designed and validated to measure frailty *only* in younger populations. However, in study populations that included people aged under 60 years, there was some evidence that cumulative deficit frailty indices, phenotype measures, the FRAIL scale, the Short Physical Performance Battery, and the Liver Frailty Index have predictive validity. These measures demonstrated validity in both general and single diagnosis populations, and those

For peer review only - http://bmjopen.bmj.com/site/about/guidelines.xhtml

undergoing some types of surgical procedures. Evidence of predictive validity for other measures was either absent or less certain, and evidence of criterion validity was sparse.

Overall the evidence suggests that some measures allow for the identification of frailty in younger populations. However, a more cautious interpretation should consider the role of sample age. Most studies used populations that included both people aged over and under 60 years of age. We also know that the prevalence of frailty is higher in older, rather than younger, groups.[128, 129] Therefore, it is possible that any observed predictive validity was due to higher prevalence of frailty among older participants. Only two studies stratified their analysis of prediction by age group: both showed that the ability of the frailty measures to predict mortality was inconsistent between younger and older age groups.[21, 23] We are also aware of another study that showed a phenotype measure does not predict mortality for women aged 37 to 45 years.[130] However, this study was not included in our review as it was not possible to determine if the majority of the sample were aged under 60 years. Furthermore, most study populations were aged, on average, over 40 years. Therefore, when concluding that measures observed validity in younger populations, we are unclear to what extent this reflects validity for entire 18 to 59 year adult age spectrum.

We identified no studies that evaluated the impact of assessing frailty in younger populations. In many of the studies, there was speculation about the proposed benefits of assessing frailty, such as investigation of patient health status;[67, 110, 123] patient prognosis;[55, 62, 65, 71, 131] risk stratification and pre-operative decision-making;[61-64, 66, 69, 85-87, 93, 99, 102, 109] identifying early or preventive intervention;[47, 65, 93, 126] and patient counselling.[44, 45, 49, 61, 93] Despite these speculations, evidence is needed to clearly establish whether assessing frailty in younger groups does indeed make a difference to patient care in the ways suggested. The absence of any evidence about the impact of measuring frailty is not specific to younger populations. Others have noted the need for evidence about the utility of frailty instruments in practice and whether such tools usefully inform clinical decision making in care for older populations.[2]

A final observation about these findings concerns terminology. We identified a small number of studies where frailty was measured using tools designed for quantifying dependency and in one case, risk of pressure ulcers.[131-133] Whilst dependency and frailty are related,[134-136] they are not conceptually equivalent, and the use of these tools was not clearly justified. The use of these measures signals either some conceptual ambiguity about the nature of frailty, or what Bouillon and colleagues, in their 2013 review of frailty measures in older populations, call ‘a lack of terminological rigour’ (p.4).[25] The implications for this review were that we were unable to reliably interpret any evidence about the validity of these tools as measures of frailty, and were thus excluded.

For peer review only - http://bmjopen.bmj.com/site/about/guidelines.xhtml

Strengths and limitations

The primary aim of this review was to identify how frailty was measured in younger populations, and the validity of such measures. A comprehensive search strategy using electronic databases, grey literature, systematic reviews and publications about measure validation referenced within included studies, has captured the core literature to achieve this aim.

Our conclusions about validity are based on a synthesis of evidence that predominantly concerns the ability of measures to predict mortality and hospital admissions. We chose these outcomes to assess predictive validity as they are known consequences of frailty in older groups.[33-36] We extended this assumption to younger populations but we acknowledge the limitations of this: evidence about outcomes of frailty *specifically* in younger populations is far less established. We also judged predictive validity based on the statistical significance of prediction. This facilitated an efficient summary assessment of evidence for this rapid review. Future work could add further insight into the validity of measures by considering the size and strength of prediction, particularly if more studies that stratify analyses by age group become available.

We did not undertake a quality assessment due to the rapid nature of this review. Whilst an assessment of study quality can identify the most robust studies, we do not believe such assessment would change the conclusions of this review. A key finding is that we are uncertain to what extent the predictive validity of frailty measures in younger populations is driven by the presence of older sample participants, among whom frailty is more prevalent. Given this limitation of the evidence, identifying the most robust studies would not enhance our certainty about the validity of measures in younger groups.

Finally, our focus on frailty measurement in younger populations was operationalised by excluding studies where the majority were aged, or the average sample age was, 60 years and over. We used the age threshold of 60, and not 65, years, as initial scoping indicated that studies on frailty in older populations typically used samples aged 60 years and over. This criterion has inevitably excluded studies where frailty was measured in samples that were, on average, older than 60 years but nonetheless included younger groups. However, this criterion was necessary to focus on evidence about younger groups and to avoid duplicating existing reviews of frailty measurement in older populations. By using this approach, we have produced a novel review and addressed a notable gap in our understanding of frailty measurement.

Implications for practice and future research

For peer review only - http://bmjopen.bmj.com/site/about/guidelines.xhtml

Identifying frailty in younger groups may have value if it aids the delivery of care for those who experience age-related illness and disability earlier in life. However, we identified no evidence about the impact of measuring frailty in younger groups, and the utility of doing so remains uncertain. Even if frailty assessment in healthcare is extended to those aged under 65, clearer evidence is needed about the validity of frailty measures *across* the younger adult age spectrum.

It is also worth noting that the measures identified in this review were no different to those used with older populations. This may reflect an assumption that frailty is homogenous across older and younger groups, or it may simply be driven by the availability of frailty measures. Without evidence about the impact of measuring frailty in younger populations, it is impossible to judge whether this is appropriate. This also presents a question about construct validity: is frailty – a concept normally associated with ageing – the same, and thus measurable using existing tools, in younger populations? These are important issues to address and clarify should the assessment of frailty in younger populations be pursued in healthcare policy and clinical practice.

Conclusions and implications

Approaches to measuring frailty in younger groups mirrored those used in older populations. Some of these measures of frailty demonstrated predictive validity in samples that *included* those aged under 60 years. However, further evidence is needed to draw clear conclusions about the validity of these measures across the adult age spectrum. Despite the potential utility of identifying frailty in younger populations evidence about the impact on patient outcomes, staff, clinical management and health service utilisation is needed.

For peer review only - http://bmjopen.bmj.com/site/about/guidelines.xhtml

Conflict of interest

No conflicts of interest.

References

1. Clegg, A, Young, J, Iliffe, S, et al. Frailty in elderly people. The Lancet 2013;381(9868):752-762.
2. Hoogendijk, EO, Afilalo, J, Ensrud, KE, et al. Frailty: implications for clinical practice and public health. The Lancet 2019;394(10206):1365-1375.
3. Cunha, AIL, Veronese, N, de Melo Borges, S, et al. Frailty as a predictor of adverse outcomes in hospitalized older adults: A systematic review and meta-analysis. Ageing Res Rev 2019;56:100960.
4. Song, X, Mitnitski, A, Rockwood, K. Prevalence and 10-Year Outcomes of Frailty in Older Adults in Relation to Deficit Accumulation. Journal of the American Geriatrics Society 2010;58(4):681-687.
5. Bagshaw, SM, Stelfox, HT, Johnson, JA, et al. Long-term association between frailty and health-related quality of life among survivors of critical illness: a prospective multicenter cohort study. Crit Care Med 2015;43(5):973-982.
6. Puts, MTE, Lips, P, Ribbe, MW, et al. The effect of frailty on residential/nursing home admission in the Netherlands independent of chronic diseases and functional limitations. European journal of ageing 2005;2(4):264-274.
7. Ofori-Asenso, R, Chin, KL, Mazidi, M, et al. Global Incidence of Frailty and Prefrailty Among Community-Dwelling Older Adults: A Systematic Review and Meta-analysis. JAMA Network Open 2019;2(8):e198398-e198398.
8. Fried, LP, Tangen, CM, Walston, J, et al. Frailty in older adults: evidence for a phenotype. The journals of gerontology Series A, Biological sciences and medical sciences 2001;56(3):M146-156.
9. Mitnitski, AB, Mogilner, AJ, Rockwood, K. Accumulation of deficits as a proxy measure of aging. ScientificWorldJournal 2001;1:323-336.
10. Rockwood, K, Mitnitski, A. Frailty in Relation to the Accumulation of Deficits. The Journals of Gerontology: Series A 2007;62(7):722-727.
11. Walston, J, Buta, B, Xue, QL. Frailty Screening and Interventions: Considerations for Clinical Practice. Clin Geriatr Med 2018;34(1):25-38.
12. Faller, JW, Pereira, DdN, de Souza, S, et al. Instruments for the detection of frailty syndrome in older adults: A systematic review. PloS one 2019;14(4):e0216166-e0216166.
13. Aguayo, GA, Donneau, A-F, Vaillant, MT, et al. Agreement Between 35 Published Frailty Scores in the General Population. American Journal of Epidemiology 2017;186(4):420-434.
14. University of York, Yorkshire & Humber AHSN. Recognising and managing frailty in primary care. University of York; 2017.
15. NHS England. NHS RightCare: Frailty Toolkit. NHS England; 2019.
16. NHS England. Supporting routine frailty identification and frailty through the GP Contract 2017/18. NHS England; 2019.
17. Dent, E, Morley, JE, Cruz-Jentoft, AJ, et al. Physical Frailty: ICFSR International Clinical Practice Guidelines for Identification and Management. The journal of nutrition, health & aging 2019;23(9):771-787.
18. Chamberlain, AM, Finney Rutten, LJ, Wilson, PM, et al. Neighborhood socioeconomic disadvantage is associated with multimorbidity in a geographically-defined community. BMC Public Health 2020;20(1):13.

For peer review only - http://bmjopen.bmj.com/site/about/guidelines.xhtml

19. Matthews, RJ, Smith, LK, Hancock, RM, et al. Socioeconomic factors associated with the onset of disability in older age: a longitudinal study of people aged 75 years and over. Social Science & Medicine 2005;61(7):1567-1575.
20. van Groenou, MIB, Deeg, DJH, Penninx, BWJH. Income differentials in functional disability in old age: Relative risks of onset, recovery, decline, attrition and mortality. Aging Clinical and Experimental Research 2003;15(2):174-183.
21. Blodgett, JM, Theou, O, Howlett, SE, et al. A frailty index from common clinical and laboratory tests predicts increased risk of death across the life course. Geroscience 2017;39(4):447-455.
22. Jayanama, K, Theou, O, Blodgett, JM, et al. Frailty, nutrition-related parameters, and mortality across the adult age spectrum. BMC Med 2018;16(1):188.
23. Rockwood, K, Song, X, Mitnitski, A. Changes in relative fitness and frailty across the adult lifespan: evidence from the Canadian National Population Health Survey. CMAJ Canadian Medical Association Journal 2011;183(8):E487-494.
24. de Vries, NM, Staal, JB, van Ravensberg, CD, et al. Outcome instruments to measure frailty: A systematic review. Ageing Research Reviews 2011;10(1):104-114.
25. Bouillon, K, Kivimaki, M, Hamer, M. Measures of frailty in population-based studies: an overview. BMC Geriatr 2013;13.
26. Faller, JW, Pereira, DdN, de Souza, S, et al. Instruments for the detection of frailty syndrome in older adults: A systematic review. PLOS ONE 2019;14(4):e0216166.
27. Tricco, AC, Antony, J, Zarin, W, et al. A scoping review of rapid review methods. BMC Medicine 2015;13(1):224.
28. Open Grey. http://www.opengrey.eu/.
29. Social Care Institute for Excellence. https://www.scie-socialcareonline.org.uk/.
30. National Institute of Health & Care Excellence. https://www.nice.org.uk/. Accessed.
31. Rockwood, K, Howlett, SE. Fifteen years of progress in understanding frailty and health in aging. BMC Medicine 2018;16(1):220.
32. Bouillon, K, Kivimaki, M, Hamer, M, et al. Measures of frailty in population-based studies: an overview. BMC Geriatr 2013;13:64.
33. Chang, S-F, Lin, P-L. Frail phenotype and mortality prediction: A systematic review and meta-analysis of prospective cohort studies. International Journal of Nursing Studies 2015;52(8):1362-1374.
34. Kojima, G, Iliffe, S, Walters, K. Frailty index as a predictor of mortality: a systematic review and meta-analysis. Age and Ageing 2017;47(2):193-200.
35. Salminen, M, Viljanen, A, Eloranta, S, et al. Frailty and mortality: an 18-year follow-up study among Finnish community-dwelling older people. Aging Clinical and Experimental Research 2019.
36. Kojima, G. Frailty as a predictor of hospitalisation among community-dwelling older people: a systematic review and meta-analysis. Journal of Epidemiology and Community Health 2016;70(7):722-729.
37. Bland, JM, Altman, DG. Validating scales and indexes. BMJ 2002;324(7337):606-607.
38. Onen, NF, Patel, P, Baker, J, et al. Frailty and Pre-Frailty in a Contemporary Cohort of HIV-Infected Adults. J Frailty Aging 2014;3(3):158-165.
39. Onen, NF, Shacham, E, Stamm, KE, et al. Sexual behaviors and results of bacterial sexually transmitted infections testing among frail HIV-infected individuals. J Int Assoc Physicians AIDS Care (Chic) 2010;9(1):30-33.
40. Prince, CS, Noren Hooten, N, Mode, NA, et al. Frailty in middle age is associated with frailty status and race-specific changes to the transcriptome. Aging (Albany NY) 2019;11(15):5518-5534.

For peer review only - http://bmjopen.bmj.com/site/about/guidelines.xhtml

41. Rochira, V, Diazzi, C, Santi, D, et al. Low testosterone is associated with poor health status in men with human immunodeficiency virus infection: a retrospective study. Andrology 2015;3(2):298-308.
42. Vatanen, A, Hou, M, Huang, T, et al. Clinical and biological markers of premature aging after autologous SCT in childhood cancer. Bone Marrow Transplant 2017;52(4):600-605.
43. Wilson, CL, Chemaitilly, W, Jones, KE, et al. Modifiable Factors Associated With Aging Phenotypes Among Adult Survivors of Childhood Acute Lymphoblastic Leukemia. J Clin Oncol 2016;34(21):2509-2515.
44. Adams, P, Ghanem, T, Stachler, R, et al. Frailty as a predictor of morbidity and mortality in inpatient head and neck surgery. JAMA Otolaryngol Head Neck Surg 2013;139(8):783-789.
45. Ahmed, AK, Goodwin, CR, De la Garza-Ramos, R, et al. Predicting Short-Term Outcome After Surgery for Primary Spinal Tumors Based on Patient Frailty. World Neurosurgery 108:393-398.
46. Akgun, KM, Tate, JP, Crothers, K, et al. An adapted frailty-related phenotype and the VACS index as predictors of hospitalization and mortality in HIV-infected and uninfected individuals. J Acquir Immune Defic Syndr 2014;67(4):397-404.
47. Akyar, S, Armenia, SJ, Ratnani, P, et al. The Impact of Frailty on Postoperative Cardiopulmonary Complications in the Emergency General Surgery Population. Surg J (N Y) 2018;4(2):e66-e77.
48. Asemota, AO, Gallia, GL. Impact of frailty on short-term outcomes in patients undergoing transsphenoidal pituitary surgery. J Neurosurg 2019;132(2):360-370.
49. Bagshaw, M, Majumdar, SR, Rolfson, DB, et al. A prospective multicenter cohort study of frailty in younger critically ill patients. Crit Care 2016;20(1):175.
50. Balla, FM, Yheulon, CG, Stetler, JL, et al. Ventral hernia repair outcomes predicted by a 5-item modified frailty index using NSQIP variables. Hernia 2019;23(5):891-898.
51. Banaszek, D, Inglis, T, Marion, TE, et al. Effect of Frailty on Outcome after Traumatic Spinal Cord Injury. J Neurotrauma 2020;37(6):839-845.
52. Bao, Y, Dalrymple, L, Chertow, GM, et al. Frailty, dialysis initiation, and mortality in end-stage renal disease. Arch Intern Med 2012;172(14):1071-1077.
53. Brothers, TD, Kirkland, S, Theou, O, et al. Predictors of transitions in frailty severity and mortality among people aging with HIV. PLoS One 2017;12(10):e0185352.
54. Chao, CT, Wang, J, Chien, KL, et al. Both pre-frailty and frailty increase healthcare utilization and adverse health outcomes in patients with type 2 diabetes mellitus. Cardiovasc Diabetol 2018;17(1):130.
55. Chode, S, Malmstrom, TK, Miller, DK, et al. Frailty, Diabetes, and Mortality in Middle-Aged African Americans. J Nutr Health Aging 2016;20(8):854-859.
56. Chu, NM, Deng, A, Ying, H, et al. Dynamic Frailty Before Kidney Transplantation: Time of Measurement Matters. Transplantation 2019;103(8):1700-1704.
57. Chung, CJ, Wu, C, Jones, M, et al. Reduced handgrip strength as a marker of frailty predicts clinical outcomes in patients with heart failure undergoing ventricular assist device placement. J Card Fail 2014;20(5):310-315.
58. Courtwright, AM, Zaleski, D, Gardo, L, et al. Causes, Preventability, and Cost of Unplanned Rehospitalizations Within 30 Days of Discharge After Lung Transplantation. Transplantation 2018;102(5):838-844.
59. Delgado, C, Grimes, BA, Glidden, DV, et al. Association of Frailty based on self-reported physical function with directly measured kidney function and mortality. BMC Nephrol 2015;16:203.
60. Erlandson, KM, Allshouse, AA, Jankowski, CM, et al. Comparison of functional status instruments in HIV-infected adults on effective antiretroviral therapy. HIV Clin Trials 2012;13(6):324-334.

For peer review only

For peer review only - http://bmjopen.bmj.com/site/about/guidelines.xhtml

61. Fang, ZB, Hu, FY, Arya, S, et al. Preoperative frailty is predictive of complications after major lower extremity amputation. J Vasc Surg 2017;65(3):804-811.
62. Fernando, SM, McIsaac, DI, Rochwerg, B, et al. Frailty and invasive mechanical ventilation: association with outcomes, extubation failure, and tracheostomy. Intensive Care Med 2019;45(12):1742-1752.
63. Fitzpatrick, J, Sozio, SM, Jaar, BG, et al. Frailty, body composition and the risk of mortality in incident hemodialysis patients: the Predictors of Arrhythmic and Cardiovascular Risk in End Stage Renal Disease study. Nephrol Dial Transplant 2019;34(2):346-354.
64. George, EM, Burke, WM, Hou, JY, et al. Measurement and validation of frailty as a predictor of outcomes in women undergoing major gynaecological surgery. BJOG 2016;123(3):455-461.
65. Griffin, FR, Mode, NA, Ejiogu, N, et al. Frailty in a racially and socioeconomically diverse sample of middle-aged Americans in Baltimore. PLoS One 2018;13(4):e0195637.
66. Guaraldi, G, Brothers, TD, Zona, S, et al. A frailty index predicts survival and incident multimorbidity independent of markers of HIV disease severity. AIDS 2015;29(13):1633-1641.
67. Guaraldi, G, Dolci, G, Zona, S, et al. A frailty index predicts post-liver transplant morbidity and mortality in HIV-positive patients. AIDS Res Ther 2017;14(1):37.
68. Gustafson, DR, Shi, Q, Holman, S, et al. Predicting death over 8 years in a prospective cohort of HIV-infected women: the Women's Interagency HIV Study. BMJ Open 2017;7(6):e013993.
69. Haugen, CE, McAdams-DeMarco, M, Verna, EC, et al. Association Between Liver Transplant Wait-list Mortality and Frailty Based on Body Mass Index. JAMA Surg 2019;154(12):1103-1109.
70. Heberton, GA, Nassif, M, Bierhals, A, et al. Usefulness of Psoas Muscle Area Determined by Computed Tomography to Predict Mortality or Prolonged Length of Hospital Stay in Patients Undergoing Left Ventricular Assist Device Implantation. Am J Cardiol 2016;118(9):1363-1367.
71. Hewitt, J, Carter, B, McCarthy, K, et al. Frailty predicts mortality in all emergency surgical admissions regardless of age. An observational study. Age Ageing 2019;48(3):388-394.
72. Hope, AA, Hsieh, SJ, Petti, A, et al. Assessing the Usefulness and Validity of Frailty Markers in Critically Ill Adults. Ann Am Thorac Soc 2017;14(6):952-959.
73. Hyde, Z, Flicker, L, Smith, K, et al. Prevalence and incidence of frailty in Aboriginal Australians, and associations with mortality and disability. Maturitas 2016;87:89-94.
74. Jha, SR, Hannu, MK, Chang, S, et al. The Prevalence and Prognostic Significance of Frailty in Patients With Advanced Heart Failure Referred for Heart Transplantation. Transplantation 2016;100(2):429-436.
75. Jha, SR, Hannu, MK, Gore, K, et al. Cognitive impairment improves the predictive validity of physical frailty for mortality in patients with advanced heart failure referred for heart transplantation. J Heart Lung Transplant 2016;35(9):1092-1100.
76. Johansen, KL, Dalrymple, LS, Delgado, C, et al. Comparison of self-report-based and physical performance-based frailty definitions among patients receiving maintenance hemodialysis. Am J Kidney Dis 2014;64(4):600-607.
77. Johansen, KL, Dalrymple, LS, Glidden, D, et al. Association of Performance-Based and Self-Reported Function-Based Definitions of Frailty with Mortality among Patients Receiving Hemodialysis. Clin J Am Soc Nephrol 2016;11(4):626-632.
78. Johansen, KL, Delgado, C, Kaysen, GA, et al. Frailty Among Patients Receiving Hemodialysis: Evolution of Components and Associations With Mortality. J Gerontol A Biol Sci Med Sci 2019;74(3):380-386.
79. Joseph, SM, Manghelli, JL, Vader, JM, et al. Prospective Assessment of Frailty Using the Fried Criteria in Patients Undergoing Left Ventricular Assist Device Therapy. Am J Cardiol 2017;120(8):1349-1354.

For peer review only - http://bmjopen.bmj.com/site/about/guidelines.xhtml

80. Kahlon, S, Pederson, J, Majumdar, SR, et al. Association between frailty and 30-day outcomes after discharge from hospital. CMAJ 2015;187(11):799-804.
81. Kane, AE, Gregson, E, Theou, O, et al. The association between frailty, the metabolic syndrome, and mortality over the lifespan. Geroscience 2017;39(2):221-229.
82. Kelly, SG, Wu, K, Tassiopoulos, K, et al. Frailty Is an Independent Risk Factor for Mortality, Cardiovascular Disease, Bone Disease, and Diabetes Among Aging Adults With Human Immunodeficiency Virus. Clin Infect Dis 2019;69(8):1370-1376.
83. Kelm, DJ, Bonnes, SL, Jensen, MD, et al. Pre-transplant wasting (as measured by muscle index) is a novel prognostic indicator in lung transplantation. Clin Transplant 2016;30(3):247-255.
84. Konstantinidis, IT, Chouliaras, K, Levine, EA, et al. Frailty Correlates with Postoperative Mortality and Major Morbidity After Cytoreductive Surgery with Hyperthermic Intraperitoneal Chemotherapy. Ann Surg Oncol 2017;24(13):3825-3830.
85. Lai, JC, Covinsky, KE, Dodge, JL, et al. Development of a novel frailty index to predict mortality in patients with end-stage liver disease. Hepatology 2017;66(2):564-574.
86. Lai, JC, Covinsky, KE, McCulloch, CE, et al. The Liver Frailty Index Improves Mortality Prediction of the Subjective Clinician Assessment in Patients With Cirrhosis. Am J Gastroenterol 2018;113(2):235-242.
87. Lai, JC, Dodge, JL, Sen, S, et al. Functional decline in patients with cirrhosis awaiting liver transplantation: Results from the functional assessment in liver transplantation (FrAILT) study. Hepatology 2016;63(2):574-580.
88. Lai, JC, Rahimi, RS, Verna, EC, et al. Frailty Associated With Waitlist Mortality Independent of Ascites and Hepatic Encephalopathy in a Multicenter Study. Gastroenterology 2019;156(6):1675-1682.
89. Lee, SY, Yang, DH, Hwang, E, et al. The Prevalence, Association, and Clinical Outcomes of Frailty in Maintenance Dialysis Patients. J Ren Nutr 2017;27(2):106-112.
90. Legge, A, Kirkland, S, Rockwood, K, et al. Evaluating the Properties of a Frailty Index and Its Association With Mortality Risk Among Patients With Systemic Lupus Erythematosus. Arthritis Rheumatol 2019;71(8):1297-1307.
91. Li, X, Ploner, A, Karlsson, IK, et al. The frailty index is a predictor of cause-specific mortality independent of familial effects from midlife onwards: a large cohort study. BMC Med 2019;17(1):94.
92. Louwers, L, Schnickel, G, Rubinfeld, I. Use of a simplified frailty index to predict Clavien 4 complications and mortality after hepatectomy: analysis of the National Surgical Quality Improvement Project database. American Journal of Surgery 2016;211(6):1071-1076.
93. Makhani, SS, Kim, FY, Liu, Y, et al. Cognitive Impairment and Overall Survival in Frail Surgical Patients. J Am Coll Surg 2017;225(5):590-600 e591.
94. Malmstrom, TK, Miller, DK, Morley, JE. A comparison of four frailty models. J Am Geriatr Soc 2014;62(4):721-726.
95. Martin, L, McKenzie, K, Ouellette-Kuntz, H. Once frail, always frail? Frailty transitions in home care users with intellectual and developmental disabilities. Geriatr Gerontol Int 2018;18(4):547-553.
96. Mascarella, MA, Milad, D, Richardson, K, et al. Preoperative Risk Index Among Patients Undergoing Thyroid or Parathyroid Surgery. Jama Otolaryngol 2020;146(1):7-12.
97. McAdams-DeMarco, MA, Law, A, King, E, et al. Frailty and mortality in kidney transplant recipients. Am J Transplant 2015;15(1):149-154.
98. McAdams-DeMarco, MA, Law, A, Salter, ML, et al. Frailty and early hospital readmission after kidney transplantation. Am J Transplant 2013;13(8):2091-2095.
99. McAdams-DeMarco, MA, Ying, H, Olorundare, I, et al. Individual Frailty Components and Mortality in Kidney Transplant Recipients. Transplantation 2017;101(9):2126-2132.

For peer review only - http://bmjopen.bmj.com/site/about/guidelines.xhtml

100. McAdams-DeMarco, MA, Ying, H, Thomas, AG, et al. Frailty, Inflammatory Markers, and Waitlist Mortality Among Patients With End-stage Renal Disease in a Prospective Cohort Study. Transplantation 2018;102(10):1740-1746.
101. McAlister, FA, Youngson, E, Eurich, DT. Treated glycosylated hemoglobin levels in individuals with diabetes mellitus vary little by health status: A retrospective cohort study. Medicine (Baltimore) 2016;95(24):e3894.
102. McChesney, SL, Canter, DJ, Monlezun, DJ, et al. Modified Frailty Index Predicts Postoperative Outcomes in Patients Undergoing Radical Pelvic Surgery. Am Surg 2020;86(2):95-103.
103. McDonagh, J, Salamonson, Y, Ferguson, C, et al. Evaluating the convergent and discriminant validity of three versions of the frailty phenotype in heart failure: results from the FRAME-HF study. Eur J Cardiovasc Nurs 2020;19(1):55-63.
104. McIntyre, M, Gandhi, C, Dragonette, J, et al. Increasing Frailty Predicts Worse Outcomes and Increased Complications After Angiogram-Negative Subarachnoid Hemorrhages. World Neurosurg 2020;134:e181-e188.
105. McIntyre, MK, Gandhi, C, Long, A, et al. Age predicts outcomes better than frailty following aneurysmal subarachnoid hemorrhage: A retrospective cohort analysis. Clin Neurol Neurosurg 2019;187:105558.
106. McKenzie, K, Ouellette-Kuntz, H, Martin, L. Frailty as a Predictor of Institutionalization Among Adults With Intellectual and Developmental Disabilities. Intellect Dev Disabil 2016;54(2):123-135.
107. Moayedi, Y, Duero Posada, JG, Foroutan, F, et al. The prognostic significance of frailty compared to peak oxygen consumption and B-type natriuretic peptide in patients with advanced heart failure. Clin Transplant 2018;32(1):01.
108. Montgomery, CL, Zuege, DJ, Rolfson, DB, et al. Implementation of population-level screening for frailty among patients admitted to adult intensive care in Alberta, Canada. Can J Anaesth 2019;66(11):1310-1319.
109. Montgomery, E, Macdonald, PS, Newton, PJ, et al. Frailty as a Predictor of Mortality in Patients With Interstitial Lung Disease Referred for Lung Transplantation. Transplantation 2020;104(4):864-872.
110. Morley, JE, Malmstrom, TK, Miller, DK. A simple frailty questionnaire (FRAIL) predicts outcomes in middle aged African Americans. J Nutr Health Aging 2012;16(7):601-608.
111. Mosquera, C, Bermudez, JM, Evans, JL, et al. Frailty Predicts Failure to Rescue after Thoracoabdominal Operation. J Am Coll Surg 2018;226(6):978-986.
112. Nastasi, AJ, McAdams-DeMarco, MA, Schrack, J, et al. Pre-Kidney Transplant Lower Extremity Impairment and Post-Kidney Transplant Mortality. Am J Transplant 2018;18(1):189-196.
113. Ness, KK, Krull, KR, Jones, KE, et al. Physiologic frailty as a sign of accelerated aging among adult survivors of childhood cancer: a report from the St Jude Lifetime cohort study. J Clin Oncol 2013;31(36):4496-4503.
114. Ney, M, Tangri, N, Dobbs, B, et al. Predicting Hepatic Encephalopathy-Related Hospitalizations Using a Composite Assessment of Cognitive Impairment and Frailty in 355 Patients With Cirrhosis. Am J Gastroenterol 2018;113(10):1506-1515.
115. Pamukcuoglu, M, Bhatia, S, Weisdorf, DJ, et al. Hematopoietic Cell Transplant-Related Toxicities and Mortality in Frail Recipients. Biol Blood Marrow Transplant 2019;25(12):2454-2460.
116. Pena, FG, Theou, O, Wallace, L, et al. Comparison of alternate scoring of variables on the performance of the frailty index. BMC Geriatr 2014;14:25.
117. Ravindrarajah, R, Lee, DM, Pye, SR, et al. The ability of three different models of frailty to predict all-cause mortality: results from the European Male Aging Study (EMAS). Arch Gerontol Geriatr 2013;57(3):360-368.

For peer review only - http://bmjopen.bmj.com/site/about/guidelines.xhtml

118. Rozenberg, D, Mathur, S, Wickerson, L, et al. Frailty and clinical benefits with lung transplantation. J Heart Lung Transplant 2018;37(10):1245-1253.
119. Shin, JI, Kothari, P, Phan, K, et al. Frailty Index as a Predictor of Adverse Postoperative Outcomes in Patients Undergoing Cervical Spinal Fusion. Spine (Phila Pa 1976) 2017;42(5):304-310.
120. Singer, JP, Diamond, JM, Anderson, MR, et al. Frailty phenotypes and mortality after lung transplantation: A prospective cohort study. Am J Transplant 2018;18(8):1995-2004.
121. Singer, JP, Diamond, JM, Gries, CJ, et al. Frailty Phenotypes, Disability, and Outcomes in Adult Candidates for Lung Transplantation. Am J Respir Crit Care Med 2015;192(11):1325-1334.
122. Subramaniam, S, Aalberg, JJ, Soriano, RP, et al. New 5-Factor Modified Frailty Index Using American College of Surgeons NSQIP Data. J Am Coll Surg 2018;226(2):173-181 e178.
123. Susanto, M, Hubbard, RE, Gardiner, PA. Validity and Responsiveness of the FRAIL Scale in Middle-Aged Women. J Am Med Dir Assoc 2018;19(1):65-69.
124. Traven, SA, Horn, RW, Reeves, RA, et al. The 5-Factor Modified Frailty Index Predicts Complications, Hospital Admission, and Mortality Following Arthroscopic Rotator Cuff Repair. Arthroscopy 2020;36(2):383-388.
125. Verheij, E, Kirk, GD, Wit, FW. Frailty is associated with mortality and incident comorbidity among middle-aged HIV-positive and HIV-negative participants. Care Management 2020;26(1):22-22.
126. Myers, V, Drory, Y, Gerber, Y, et al. Clinical relevance of frailty trajectory post myocardial infarction. Eur J Prev Cardiol 2014;21(6):758-766.
127. !!! INVALID CITATION !!! 56, 59, 69, 82.
128. Rockwood, K, Howlett, SE, MacKnight, C, et al. Prevalence, attributes, and outcomes of fitness and frailty in community-dwelling older adults: report from the Canadian study of health and aging. The journals of gerontology Series A, Biological sciences and medical sciences 2004;59(12):1310-1317.
129. Blodgett, JM, Theou, O, Mitnitski, A, et al. Associations between a laboratory frailty index and adverse health outcomes across age and sex. Aging Med (Milton) 2019;2(1):11-17.
130. Hanlon, P, Nicholl, BI, Jani, BD, et al. Frailty and pre-frailty in middle-aged and older adults and its association with multimorbidity and mortality: a prospective analysis of 493 737 UK Biobank participants. The Lancet Public Health 2018;3(7):e323-e332.
131. Tapper, EB, Finkelstein, D, Mittleman, MA, et al. Standard assessments of frailty are validated predictors of mortality in hospitalized patients with cirrhosis. Hepatology 2015;62(2):584-590.
132. Dolgin, NH, Martins, PN, Movahedi, B, et al. Functional status predicts postoperative mortality after liver transplantation. Clin Transplant 2016;30(11):1403-1410.
133. Zdradzinski, MJ, Phelan, MP, Mace, SE. Impact of Frailty and Sociodemographic Factors on Hospital Admission From an Emergency Department Observation Unit. Am J Med Qual 2017;32(3):299-306.
134. At, J, Bryce, R, Prina, M, et al. Frailty and the prediction of dependence and mortality in low- and middle-income countries: a 10/66 population-based cohort study. BMC Medicine 2015;13(1):138.
135. Bagshaw, SM, Stelfox, HT, McDermid, RC, et al. Association between frailty and short- and long-term outcomes among critically ill patients: a multicentre prospective cohort study. Canadian Medical Association Journal 2014;186(2):E95-E102.
136. Boyd, CM, Xue, Q-L, Simpson, CF, et al. Frailty, hospitalization, and progression of disability in a cohort of disabled older women. The American Journal of Medicine 2005;118(11):1225-1231.

For peer review only - http://bmjopen.bmj.com/site/about/guidelines.xhtml

Figure legends:

Figure 1. PRISMA Flowchart

Figure 2. Proportion (%) of studies with different average sample ages

Figure 3a. Evidence of validity for cumulative deficit frailty indices by study sample mean/median age (years)

Figure 3b. Evidence of validity for phenotype measures by study sample mean/median age (years)

For peer review only

Figure 1. PRISMA Flowchart

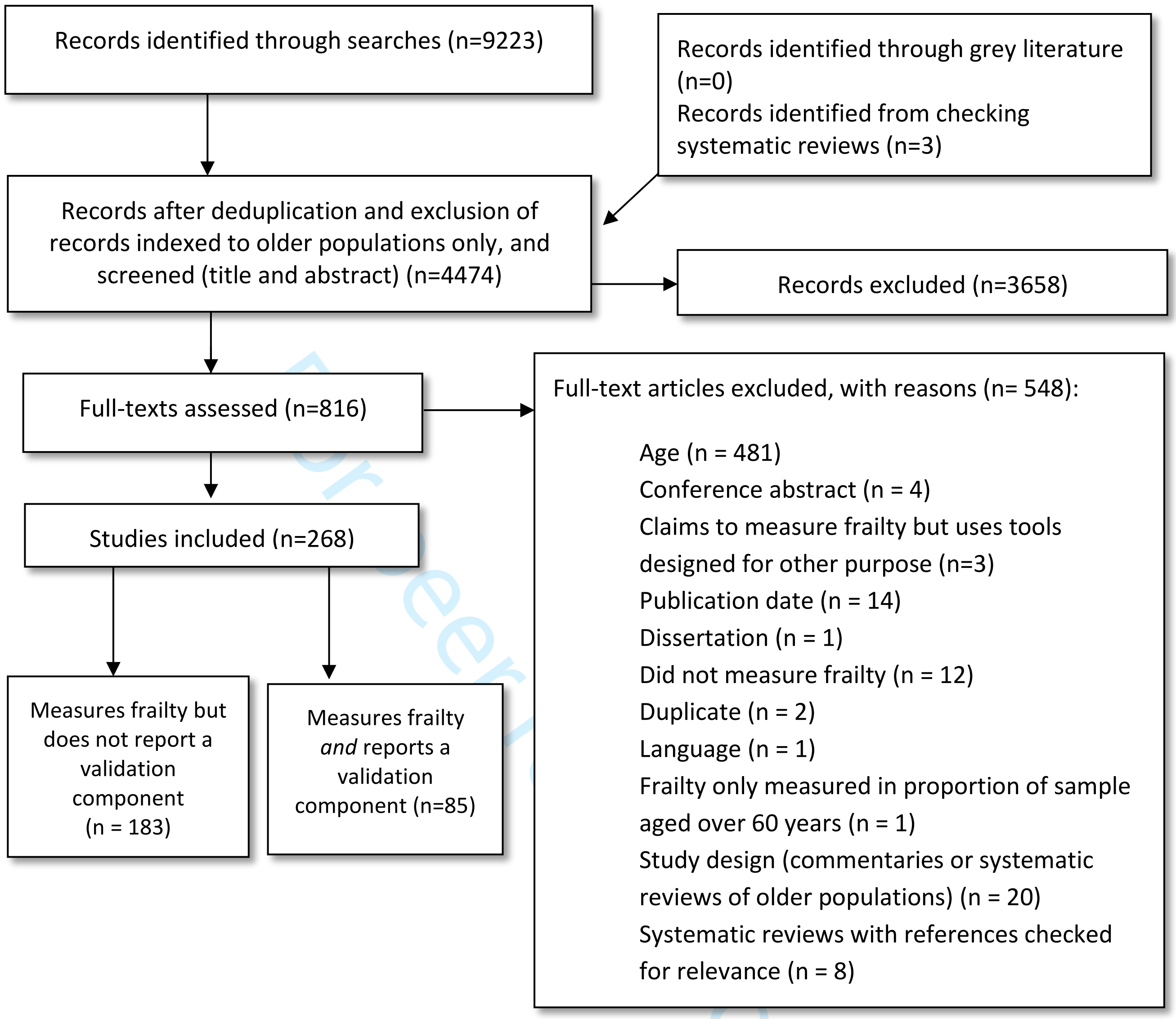

Figure 2. Proportion (%) of studies with different average sample ages [a]

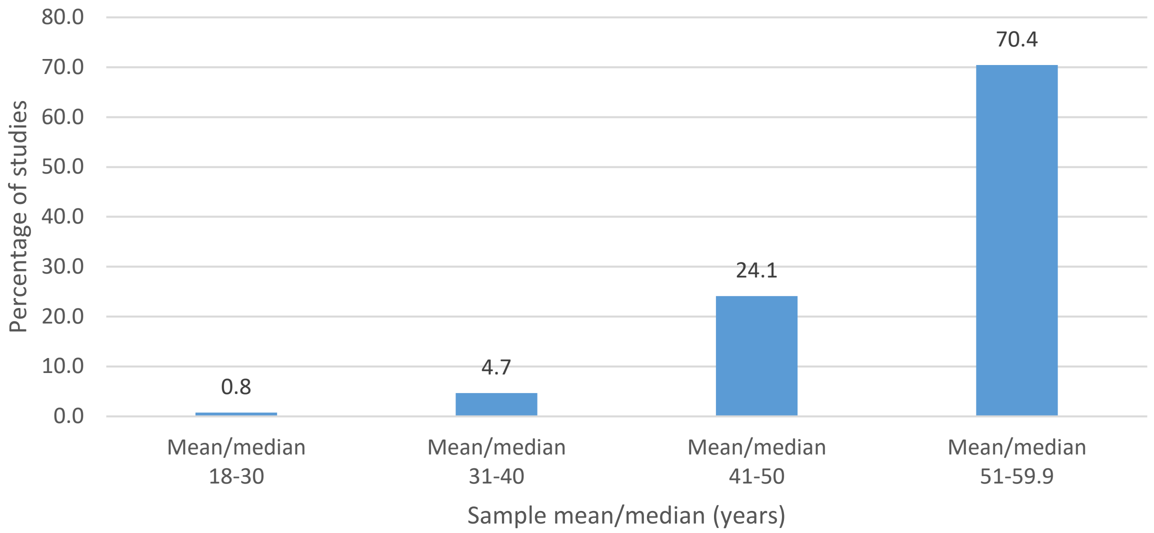

[a]*Calculated from studies where a mean or median sample age was reported (n=257)*

peer review only

Figure 3a. Evidence of validity for cumulative deficit frailty indices by study sample mean/median age (years)

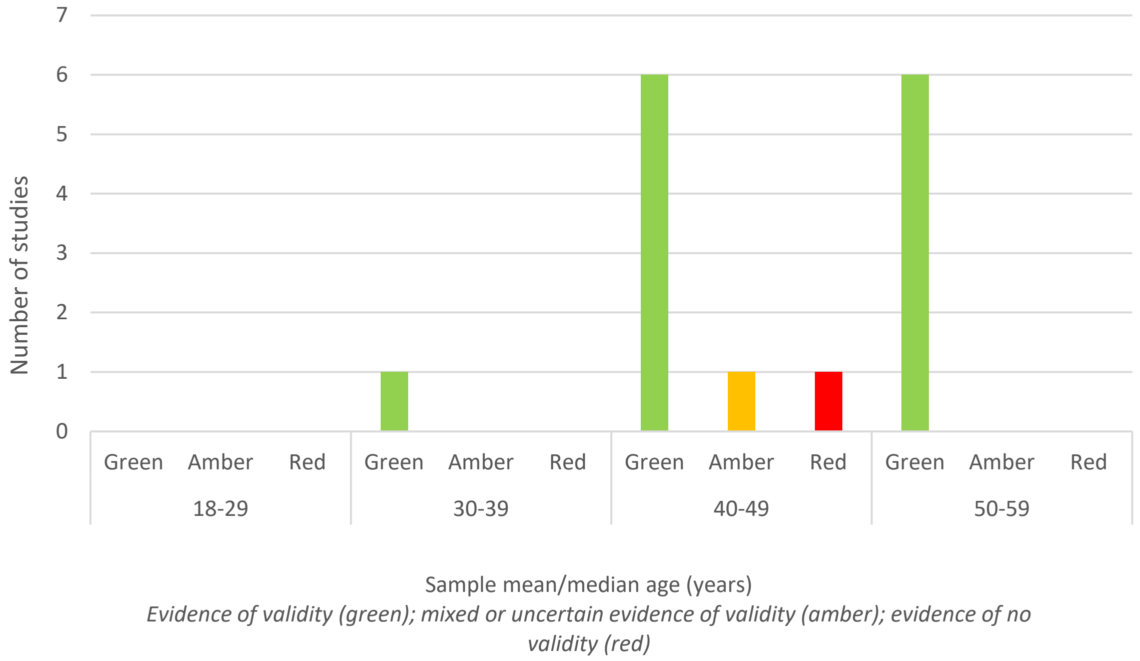

The reader is referred to Table S3 for details of the studies using cumulative deficit frailty indices and their corresponding evidence classification.

Figure 3b. Evidence of validity for phenotype measures by study sample mean/median age (years)

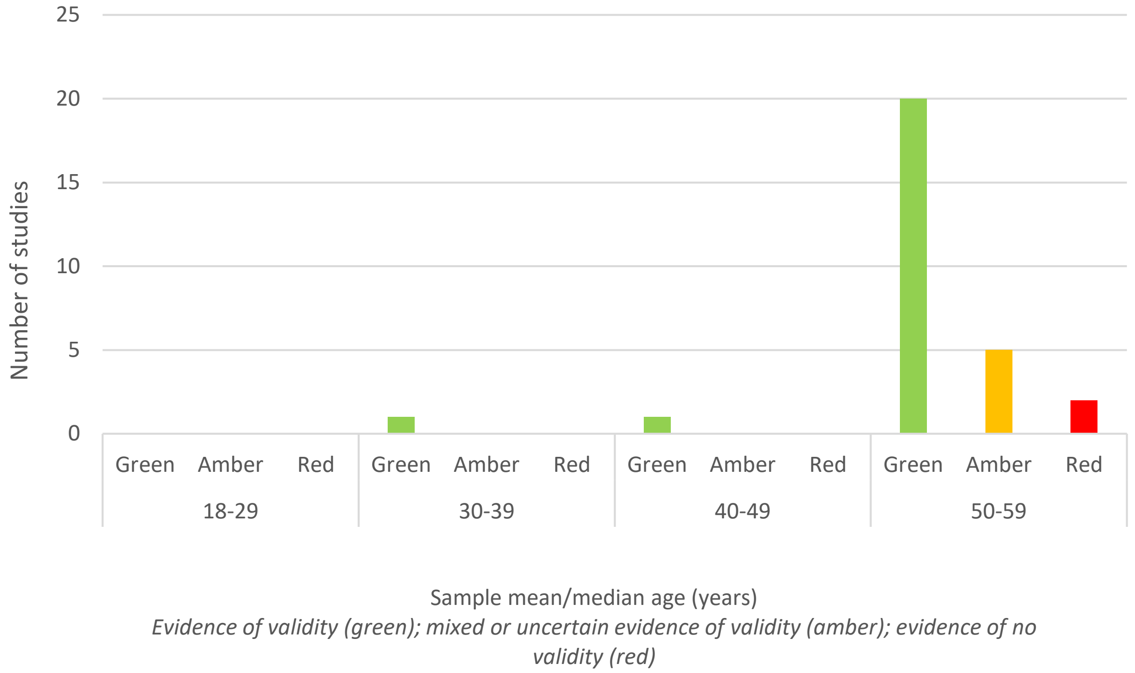

The reader is referred to Table S3 for details of the studies using phenotype measures and their corresponding evidence classification.

Supplementary materials

Searches strategy applied to Medline

Searched 17th March 2020; Database(s): **Ovid MEDLINE(R)** 1946 to March Week 1 2020

Key:

/	thesaurus heading
.ti,ab	terms must appear in the title or abstract fields
adj8	the terms either side must be within eight words of each other
*	unlimited truncation
?	single letter truncation

#	Searches	Results
1	Frailty/cl, di, ep [Classification, Diagnosis, Epidemiology]	1007
2	Frailty/	2162
3	"Sensitivity and Specificity"/	343505
4	"Predictive Value of Tests"/	199066
5	ROC Curve/	56249
6	"Diagnostic Techniques and Procedures"/	3243
7	exp Physical Examination/	1349129
8	Symptom Assessment/	4429
9	Psychometrics/	73737
10	Prevalence/	284108
11	or/3-10	2159330
12	2 and 11	631
13	((frail* or prefrail* or pre frail*) adj8 (measur* or assess* or estimat* or define? or definition or classif* or test* or identif* or observ* or screen* or review* or indicat* or criteri* or sign? or symptom? or prevalence*)).ti,ab.	5778
14	((frail* or prefrail* or pre frail*) adj8 (instrument? or tool? or framework* or index* or score? or scale? or psychometric or reliable or reliability or valid* or responsive*)).ti,ab.	2733
15	((frail* or prefrail* or pre frail*) adj8 (associat* or predict*)).ti,ab.	3371
16	or/13-15	7248
17	1 or 12 or 16	7460
18	exp Aged/	3062714
19	adult/ or middle aged/ or young adult/	6433186
20	18 not (18 and 19)	645469
21	17 not 20	2842
22	limit 21 to (english language and humans and yr="2000 -Current")	2511

For peer review only

Supplementary materials table S1. Framework to determine evidence of measure validity (predictive and/or criterion)

Green (evidence of validity)	• Prediction of mortality and/or hospital admissions was statistically significant<br>• Associations and agreement between measures (criterion validity) were judged to be good or strong by study authors
Amber (unclear or inconsistent evidence of validity)	• Evidence of validity was mixed because:<br>o The frailty measure was tested against both outcomes (mortality and hospital admission) but observed a significant association for only one outcome<br>o Prediction was statistically significant for only some sub-groups (e.g. age groups)<br>o Evidence of either, but not both, predictive or criterion validity was demonstrated<br>o Evidence of predictive validity varied depending on when (e.g. 30 day) and where (e.g. hospital or ICU) mortality was measured, the level of frailty tested (e.g. moderate or severe), or how the frailty score was used (e.g. as continuous or binary)<br>o Where individual components of the frailty measure were tested and only some, not all, demonstrated predictive validity<br>• Only differences in mortality rates between frail and not frail were reported, with no test of prediction<br>• Unclear reporting in the paper made it difficult to determine whether the measure was intended to capture frailty or a different concept<br>• Associations and agreement between measures (criterion validity) were judged to be fair or modest by study authors
Red (no evidence of validity)	• Prediction of mortality and/or hospital admissions was not statistically significant<br>• Data were not reported in the publication to verify a claim that the frailty measure predicted mortality and/or hospital admissions<br>• Associations and agreement between measures (criterion validity) were judged to be poor or weak by study authors

For peer review only

Supplementary materials table S2. Number of studies using frailty measures[a]

Measure	Number of studies using measure
Phenotype model (or variation of these criteria)[b]	137
Cumulative deficit model frailty indices[c]	88
CFS	14
Sarcopenia/muscle wasting/muscle size/sarco-osteopenia/morphemic values of muscle (including in combination with weight and mobility)	9
Liver Frailty Index[d]	5
Short Physical Performance Battery	5
Hand grip	3
Walk Test	2
Groningen Frailty Indicator	2
Risk Analysis Index	2
Veterans Aging Cohort Study Index	2
Performance based frailty scale	1
Function Based Frailty scale	1
EQ-5D Item C	1
Comprehensive Frailty Assessment Instrument	1
Tilburg Frailty Indicator	1
Physical Performance Test	1
Blood levels of alanine amino transferase	1
Cervical Endocrine Surgery Risk Index	1
Edmonton Frail Scale	1
Physical Function Scale of SF36	1
Frailty Discriminant Score	1
Frailty Framework among Homeless and Vulnerable Populations	1
Frailty Framework among Vulnerable Populations	1
Morse Fall Risk Score	1
Chair stands	1
International Myeloma Working Group Frailty Score	1
Kidney Disease Quality of Life SF36	1
Frailty Risk Score	1
Composite score from CFS and Montreal Cognitive Assessment	1

For peer review only

Frailty Questionnaire	1
Radiographic frailty markers	1
Vascular disease (CT scan) as indicator of frailty alongside other CT scan indicators of frailty	1
Not reported	1

[a]Numbers are not mutually exclusive as some studies used more than one measure ; [b]Includes the FRAIL scale, the Study of Osteoporotic Fractures Scale, the SHARE Frailty Instrument for Primary care, and the John Hopkins Frailty Indicator, all of which use items that overlap with the phenotype model; [c]Includes frailty indices where the number of index deficits varied between studies, the modified Frailty Indices (mFI 5 and 11 item versions), the European Male Ageing Study Frailty Index, the Spinal Tumor Frailty Index, the Adult Spinal Deformity Frailty Index, and the Systemic Lupus International Collaborating Clinics Frailty Index; [d]Although termed a frailty index, the Liver Frailty Index is not based on the cumulative deficit model.

For peer review only

Supplementary materials table S3. Summary of evidence of predictive validity for frailty measures in younger populations

**Paper**	**Sample size**	**Sample age Mean/median (SD/IQR)**	**Sample population**	Type of validity PREDICTIVE (MORTALITY AND/OR ADMISSION), CRITERION(CORRELATION OR AGREEMENT))
**Phenotype**				
Ness 2013	2333	Mean: 33.6 (8.1)	Adult survivors of childhood cancer	PREDICTIVE (MORTALITY)
Delgado 2015	812	Median: 52.0 (42-61)	Chronic Kidney disease	PREDICTIVE (MORTALITY)
McAdams-DeMarco 2018	605	Mean: 53.7 (13.5)	End-stage renal disease	PREDICTIVE (MORTALITY)
Fitzpatrick 2019	370	Mean: 54.9	End stage renal disease	PREDICTIVE (MORTALITY)
Chu 2019	569	Mean: 51.7 (14.0)	End stage renal disease	PREDICTIVE (MORTALITY)
Bao 2012	1576	Mean: 59.6 (14.2)	End stage renal disease	PREDICTIVE (MORTALITY AND ADMISSIONS)
Makhani 2017	330	Mean: 58.0 (SD not reported, range 18-89)	Generic surgery	PREDICTIVE (MORTALITY)
Johansen 2016	771	Mean: 57.1 (14.2)	Haemodialysis	PREDICTIVE (MORTALITY)
Johansen 2019	727	Mean: 57.2 (14.2)	Haemodialysis	PREDICTIVE (MORTALITY)
Lee 2017	1658	Mean: 55.2 (11.9)	Dialysis	PREDICTIVE (ADMISSIONS)
Jha 2016[a,b]	156	Mean: 53.0 (12.0[a], 13.0[b])	Heart failure	PREDICTIVE (MORTALITY)
Pamukcuoglu et al 2019	117	Median: 59.0, range 40-73	Hemotopoietic cell transplant	PREDICTIVE (MORTALITY)
Akgun 2014	6515	Mean: 47.6 (8.5)	HIV	PREDICTIVE (MORTALITY AND ADMISSIONS)
Kelly 2019	1016	Median: 51.0 (46.5)	HIV	PREDICTIVE (MORTALITY)

For peer review only

Gustaffson 2017	1385	Mean: 42.6 (8.8)	HIV	PREDICTIVE (MORTALITY)
Verheij 2020	598	Median:<br>Robust: 51.5 (47.6-56.9)<br>Prefail: 53.3 (48.6-60.1)<br>Frail: 55.1 (49.7-60.0)	HIV	PREDICTIVE (MORTALITY)
McAdams-DeMarco 2013	383	Mean: 53.5 (13.9)	Kidney transplant	PREDICTIVE (MORTALITY)
McAdams-DeMarco 2015	537	Mean: 53.0 (14.0)	Kidney transplant	PREDICTIVE (MORTALITY)
Montgomery 2020	100	Mean: 59.0 (7.0)	Lung transplant	PREDICTIVE (MORTALITY)
Singer 2018	299	Median: 59.0 (IQR: 50, 65)	Lung Transplant	PREDICTIVE (MORTALITY)
Ravindrarajah 2013	2929	Mean: 59.9 (10.8)	General population	PREDICTIVE (MORTALITY)
Malmstrom 2014	998	Mean: (at baseline according to tertile): 51.4 (1.1); 56.0 (1.5); 61.5, (2.0).	General population	PREDICTIVE (MORTALITY)
Hope 2017	95	Mean: 57.1, (17.5)	Critical illness/ICU	PREDICTIVE (MORTALITY)<br>*But as a composite outcome combining death with disability outcome.*
Singer 2015	395	Median: 59.0 (IQR: 50–64)	Lung Transplant	PREDICTIVE (MORTALITY)
Moayedi 2018	201	Mean: 53.8 (12.4)	Heart failure	PREDICTIVE (MORTALITY)
McAdams-DeMarco 2017	663	Mean: 53.0 (13.9)	Kidney transplant	PREDICTIVE (MORTALITY)
Joseph 2017	75	Mean: 58.0 (12.0)	End-stage heart failure	PREDICTIVE (MORTALITY)
Rozenberg 2018	221	Cohort 1 mean: 59.0 (9.0)<br>Cohort 2 mean: 55 (43-62)	Lung transplant	PREDICTIVE (MORTALITY)
Johansen 2014	771	Not frail: mean: 53.8 (14.4); self-report: mean: 57.6 (12.1); performance: 51.6 (15.8); self report + performance: mean 62.9, (13.0)	Haemodialysis	CRITERION (AGREEMENT BETWEEN SELF-REPORT AND PERFORMANCE BASED PHENOTYPE CRITERIA)
McDonagh 2020<br>*St Vincent's Frailty Measure (Fried criteria)*	131	Mean: 54.0 (14.0)	Heart Failure	CRITERION (CORRELATION BETWEEN THREE VERSIONS OF PHENOTYPE CRITERIA)

For peer review only

Rozenberg 2018	221	Cohort 1 mean: 59.0 (9.0)<br>Cohort 2 mean: 55.0 (SD not reported)	Lung transplant	CRITERION (AGREEMENT WITH TWO ALTERNATIVE VERSIONS OF PHENOTYPE CRITERIA BASED ON CLINICAL DATA AND A DATASET)
Erlandson 2012	359	Median: 50.8 (IQR: 47.7–55.7)	HIV	CRITERION (AGREEMENT WITH THE SHORT PHYSICAL PERFORMANCE BATTERY AND WALK TEST)
**Frailty indices, where the number of index deficits varied between studies (condition specific indices are in italics)**				
Kane 2017	8555	Mean: 40.3 (13.0) (under 65s group)	People with metabolic syndrome	PREDICTIVE (MORTALITY)
Li 2019	42953	Multiple means reported by sex and twin group. Reader referred to paper for further details.	General population	PREDICTIVE (MORTALITY)
Malmstrom 2014	998	Mean: (at baseline according to tertile): 51.4 (1.1); 56.0 (1.5); 61.5 (2.0).	General population	PREDICTIVE (MORTALITY)
McKenzie 2016	3034	Mean: 53.9 (17.3)	Intellectual & developmental disabilities	PREDICTIVE (ADMISSIONS)
Myers 2014	1521	Grouped on frailty scores ranging from mean 52.0 (8.0) to mean 77.0 (6.0)	Myocardial infarction survivors	PREDICTIVE (MORTALITY)
Rockwood 2011	14713	Mean: 44.0 (18.0)	General population	PREDICTIVE (MORTALITY)
Pena 2014	SHARE: 57546;<br>YALE-PEP: 754;<br>NSHS: 3227	NSHS: mean: 48.1 (19.8)<br>SHARE: mean: 64.2 (10.5)<br>Yale-PEP: mean: 78.4 (5.3)	General population	PREDICTIVE (MORTALITY)
Jayanama 2018	9030	Mean: 46.6 (16.9)	General population	PREDICTIVE (MORTALITY)
Hyde 2016	363	Range: 45-96 years, 53.8% aged <60 years	General population	PREDICTIVE (MORTALITY)
Guaraldi 2017	47	Mean: 51.2 (6.9)	HIV + Liver transplant	PREDICTIVE (MORTALITY)
Guaraldi 2015	2720	Mean: 46.0 (8.0)	HIV	PREDICTIVE (MORTALITY)
Brothers 2017	963	Mean: 46.8 (7.1)	HIV	PREDICTIVE (MORTALITY)
Ravindrarajah 2013	2929	Mean: 59.9 (10.8)	General population	PREDICTIVE (MORTALITY)

For peer review only - http://bmjopen.bmj.com/site/about/guidelines.xhtml

Legge 2019<br>*Systemic Lupus International Collaborating Clinics (SLICC)-FI*	1683	Mean: 35.7 (13.3)	Systemic lupus erythematosus (SLE)	PREDICTIVE (MORTALITY)
Blodgett 2017 (Lab and self report FI)	8888	Mean: 49.4 (19.0)	General population	PREDICTIVE (MORTALITY)
Martin 2018	2893	Mean: 49.5 (17.7)	Intellectual & developmental disabilities	PREDICTIVE (MORTALITY)
Ahmed 2017<br>*Spinal Tumour Frailty Index*	6727	Median: 47, IQR: 28 - 61	Spinal tumour surgery	PREDICTIVE (MORTALITY)
**Frailty indices: mFI 11**				
Adams 2013	6727	Mean: 54.7 (17.2),	Head and neck surgery	PREDICTIVE (MORTALITY)
Akyar 2018	132765	71.2% aged < 60 years	Emergency surgery	PREDICTIVE (MORTALITY)
Konstantinidis 2017	1171	Median: 55.0, range 19-87	Cytoreductive surgery with intraoperative hyperthermic intraperitoneal chemotherapy	PREDICTIVE (MORTALITY)
McChesney 2020	10048	Mean: 59.9 (5.3)	Radial pelvic surgery patients	PREDICTIVE (MORTALITY)
McIntyre 2020	75	Mean: 55.4 (1.5)	Brain haemorrhage - angiogram-negative subarachnoid haemorrhages (ANSAH)	PREDICTIVE (MORTALITY)
Mosquera 2018	962913	Mean: 53.8 (17.0)	Thoracoabdominal surgery patients	PREDICTIVE (MORTALITY)
Shin 2017	6965	ACDF group – mean: 52.9 (SD not reported)<br>PCF group – mean: 59.8 (SD not reported)	Post Cervical Spinal Fusion	PREDICTIVE (MORTALITY)<br>*But as composite outcome combining death with any post-surgical complications.*

Louwers 2017	10300	Mean: 59.0 (SD not reported)	Hepatectomy patients	PREDICTIVE (MORTALITY)<br>*But as a composite outcome combining death with any post-surgical complications.*
Fang 2017	379	Mean: 59.0 (15.0)	Lower extremity amputation	PREDICTIVE (ADMISSIONS)
McIntyre 2019	217	Mean: 57.6 (1.0)	Brain haemorrhage - aneurysmal subarachnoid haemorrhage (aSAH)	PREDICTIVE (MORTALITY)
Banasek 20	634	Mean: 50.3 (19.8)	Traumatic spinal cord injury	PREDICTIVE (MORTALITY)
**Frailty indices: mFI 5**				
Traven 2020	24477	Mean: 58.4 (SD note reported)	Post Arthroscopic Rotator Cuff Repair	PREDICTIVE (MORTALITY AND ADMISSIONS)
Subramaniam 2018	541485	2012 - 56.84 (16.76)<br>2015 - 56.42 (16.85)	Post surgery (general)	PREDICTIVE (MORTALITY AND ADMISSION)<br><br>CRITERION (CORRELATION WITH MFI 11)
Balla 2019	97905	Mean 55.9 (SD not reported)	Ventral Hernia Repair	CRITERION (CORRELATION WITH MFI 11)
**Short Physical Performance Battery**				
Courtwright 2018	90	Mean: 53.5 (14.1)	Lung transplant	PREDICTIVE (ADMISSIONS)
Lai 2016	309	Median: 59 (IQR 53-63)	End-stage liver disease	PREDICTIVE (MORTALITY)
Nastasi 2018	719	Mean: 51.6 (14.2)	Kidney transplant (recipients)	PREDICTIVE (MORTALITY)
Singer 2018	299	Median: 59 (IQR: 50, 65)	Lung Transplant	PREDICTIVE (MORTALITY)
Singer 2015	395	Median: 59 (IQR: 50–64)	Lung Transplant	PREDICTIVE (MORTALITY)<br>*But as a composite outcome combining death with transplant delisting.*
Erlandson 2012	359	Median: 50.8 (IQR: 47.7–55.7)	HIV	CRITERION (AGREEMENT WITH WALK TEST AND PHENOTYPE CRITERIA)
**Clinical Frailty Scale**				

Fernando 2019	8110	Not frail<br>Mean: 57.6 (18.1)<br>Frail<br>Mean: 69.2 (12.2)	Critical illness/ICU	PREDICTIVE (MORTALITY)
Bagshaw 2016	197	Mean: 58.5 (4.1)	Critical illness/ICU	PREDICTIVE (MORTALITY AND ADMISSIONS)
Hope 2017	95	Mean: 57.1 (17.5)	Critical illness/ICU	PREDICTIVE (MORTALITY)<br>*But as a composite outcome combining death with disability outcome.*
Ney 2018<br>MoCA-CFS	355	Mean: 55.9 (9.6)	Cirrhosis	PREDICTIVE (MORTALITY)
Kahlon 2015	495	67.3% aged under 60 years	General population	PREDICTIVE (MORTALITY AND ADMISSIONS)
Montgomery 2019	15238	Mean: 58.0 (17.0)	Critical illness/ICU	PREDICTIVE (MORTALITY)
Hewitt 2019	2279	Median: 54.0 (IQR: 36–72)	Emergency surgery	PREDICTIVE (MORTALITY AND ADMISSIONS)
**Frail Scale**				
Griffin 2018	2541	Mean 50.2 (SD not reported)	General population	PREDICTIVE (MORTALITY)
Chode 2016	998	With diabetes<br>Mean: 57.4 (4.4)<br>Without diabetes<br>Mean: 55.9 (4.4)	Diabetes	PREDICTIVE (MORTALITY)
Chao 2018	560,795	Mean: 56.4 (13.8)	Diabetes	PREDICTIVE (MORTALITY AND ADMISSIONS)
Susanto 2017	10412	Range 50–66. Mean age not reported but uses same cohort in another publication (same study team) with a mean age of 52.5 years.	General population	PREDICTIVE (MORTALITY)
Malmstrom 2014	998	Range 49-65	General population	PREDICTIVE (MORTALITY)
Ravindrarajah 2013<br>EMAS Frail Scale	2929	Mean: 59.9 (10.8)	General population	PREDICTIVE (MORTALITY)
Morley 2012	1586	Various analytic groups according to ADL difficult/dependency and frailty level, ranging from mean 56.1 (4.5) to 57.7 (3.9)	General population	PREDICTIVE (MORTALITY)

**Liver Frailty Index**				
Lai 2019	1044	Median: 57 (IQR 49-63)	End-stage liver disease	PREDICTIVE (MORTALITY)
Lai 2017	536	Median: 58 (IQR 50-63)	End-stage liver disease	PREDICTIVE (MORTALITY)
Lai 2018	529	Median: 58 (IQR 50-63)	End-stage liver disease	PREDICTIVE (MORTALITY)
Haugen 2019b	1108	Mean: 55.0 (10.0)	Liver transplant	PREDICTIVE (MORTALITY)
**SOF Frailty Scale and CHS Frailty Scale**				
Malmstrom 2014	998	Mean: (at baseline according to tertile): 51.4 (1.1); 56.0 (1.5); 61.5 (2.0).	General population	PREDICTIVE (MORTALITY)
**John Hopkins Frailty Indicator**				
Asemota 2019	115317	Frail<br>Mean: 57.1 (SD: 16.9)<br>Non-frail<br>Mean: 51.9 (15.8)	Transphenoidal Pituary Surgery	PREDICTIVE (MORTALITY)
**Walk test**				
Erlandson 2012	359	Median: 50.8, IQR: 47.7–55.7	HIV	CRITERION (AGREEMENT WITH PHENOTYPE CRITERIA AND THE SHORT PHYSICAL PERFORMANCE BATTERY)
**Muscle mass/sarcopenia**				
Kelm 2016	36	Median 56 (IQR 49,62)	Lung transplant	PREDICTIVE (MORTALITY)
Heberton 2016	333	Sarcopenic (yes): Mean: 53.0 (14.0)<br>Sarcopenic (no): Mean 57.0 (14.0)	Left ventricular assist device (LVAD) implantation	PREDICTIVE (MORTALITY)
**Hand grip**				
Chung 2014	72	Mean 59.0 (2.0)	Heart failure	PREDICTIVE (MORTALITY)
**CERSI**				

Mascarella 2020	154895	Mean: 56.1 (15.6)	Thyroid & parathyroid surgery patients	PREDICTIVE (MORTALITY)<br>*But as a composite outcome combining death with any adverse event.*

For peer review only

For peer review only

Bibliographic details of included studies [1-227228-268]

1. Abdel-Kader K, Girard TD, Brummel NE, et al. Acute Kidney Injury and Subsequent Frailty Status in Survivors of Critical Illness: A Secondary Analysis. *Crit Care Med* 2018; **46**(5): e380-e8.
2. Adams P, Ghanem T, Stachler R, Hall F, Velanovich V, Rubinfeld I. Frailty as a predictor of morbidity and mortality in inpatient head and neck surgery. *JAMA Otolaryngol Head Neck Surg* 2013; **139**(8): 783-9.
3. Ahmed AK, Goodwin CR, De la Garza-Ramos R, et al. Predicting Short-Term Outcome After Surgery for Primary Spinal Tumors Based on Patient Frailty. *World Neurosurgery*; **108**: 393-8.
4. Aizpuru M, Staley C, Reisman W, Gottschalk MB, Schenker ML. Determinants of Length of Stay After Operative Treatment for Femur Fractures. *J Orthop Trauma* 2018; **32**(4): 161-6.
5. Akgun KM, Tate JP, Crothers K, et al. An adapted frailty-related phenotype and the VACS index as predictors of hospitalization and mortality in HIV-infected and uninfected individuals. *J Acquir Immune Defic Syndr* 2014; **67**(4): 397-404.
6. Akgun KM, Tate JP, Oursler KK, et al. Association of chronic obstructive pulmonary disease with frailty measurements in HIV-infected and uninfected Veterans. *AIDS* 2016; **30**(14): 2185-93.
7. Akyar S, Armenia SJ, Ratnani P, Merchant AM. The Impact of Frailty on Postoperative Cardiopulmonary Complications in the Emergency General Surgery Population. *Surg J (N Y)* 2018; **4**(2): e66-e77.
8. Alshaikh JT, Amdur R, Sidawy A, Trachiotis G, Kaminski HJ. Thymectomy is safe for myasthenia gravis patients: Analysis of the NSQIP database. *Muscle Nerve* 2016; **53**(3): 370-4.
9. Althoff KN, Jacobson LP, Cranston RD, et al. Age, comorbidities, and AIDS predict a frailty phenotype in men who have sex with men. *J Gerontol A Biol Sci Med Sci* 2014; **69**(2): 189-98.
10. Anderson JE, Inui T, Talamini MA, Chang DC. Cholecystostomy offers no survival benefit in patients with acute acalculous cholecystitis and severe sepsis and shock. *J Surg Res* 2014; **190**(2): 517-21.
11. Andrews JS, Trupin L, Wysham KD, Hough CL, Yelin EH, Katz PP. The Impact of Frailty on Changes in Physical Function and Disease Activity Among Adults With Rheumatoid Arthritis. *ACR Open Rheumatol* 2019; **1**(6): 366-72.
12. Andrews JS, Trupin L, Yelin EH, Hough CL, Covinsky KE, Katz PP. Frailty and reduced physical function go hand in hand in adults with rheumatoid arthritis: a US observational cohort study. *Clin Rheumatol* 2017; **36**(5): 1031-9.
13. Asemota AO, Gallia GL. Impact of frailty on short-term outcomes in patients undergoing transsphenoidal pituitary surgery. *J Neurosurg* 2019; **132**(2): 360-70.
14. Ayesta A, Astiz MTV, Masa MJV, Segovia J, Cosio MDG, Martinez-Selles M. Rationale and design of the FELICITAR registry (Frailty Evaluation After List Inclusion, Characteristics and Influence on Transplantation and Results). *Clin Cardiol* 2018; **41**(3): 293-9.

For peer review only

15. Bagshaw M, Majumdar SR, Rolfson DB, Ibrahim Q, McDermid RC, Stelfox HT. A prospective multicenter cohort study of frailty in younger critically ill patients. *Crit Care* 2016; **20**(1): 175.
16. Balla FM, Yheulon CG, Stetler JL, Patel AD, Lin E, Davis SS. Ventral hernia repair outcomes predicted by a 5-item modified frailty index using NSQIP variables. *Hernia* 2019; **23**(5): 891-8.
17. Banaszek D, Inglis T, Marion TE, et al. Effect of Frailty on Outcome after Traumatic Spinal Cord Injury. *J Neurotrauma* 2020; **37**(6): 839-45.
18. Bao Y, Dalrymple L, Chertow GM, Kaysen GA, Johansen KL. Frailty, dialysis initiation, and mortality in end-stage renal disease. *Arch Intern Med* 2012; **172**(14): 1071-7.
19. Bello GA, Lucchini RG, Teitelbaum SL, Shapiro M, Crane MA, Todd AC. Development of a Physiological Frailty Index for the World Trade Center General Responder Cohort. *Curr Gerontol Geriatr Res* 2018; **2018**: 3725926.
20. Bindawas SM, Vennu V, Stubbs B. Longitudinal Relationship Between Knee Pain Status and Incident Frailty: Data from the Osteoarthritis Initiative. *Pain Med* 2018; **19**(11): 2146-53.
21. Blanco JR, Barrio I, Ramalle-Gomara E, et al. Gender differences for frailty in HIV-infected patients on stable antiretroviral therapy and with an undetectable viral load. *PLoS One* 2019; **14**(5): e0215764.
22. Blodgett JM, Theou O, Howlett SE, Rockwood K. A frailty index from common clinical and laboratory tests predicts increased risk of death across the life course. *Geroscience* 2017; **39**(4): 447-55.
23. Blodgett JM, Theou O, Mitnitski A, Howlett SE, Rockwood K. Associations between a laboratory frailty index and adverse health outcomes across age and sex. *Aging Med (Milton)* 2019; **2**(1): 11-7.
24. Bouillon K, Batty GD, Hamer M, et al. Cardiovascular disease risk scores in identifying future frailty: the Whitehall II prospective cohort study. *Heart* 2013; **99**(10): 737-42.
25. Bouillon K, Kivimaki M, Hamer M, et al. Diabetes risk factors, diabetes risk algorithms, and the prediction of future frailty: the Whitehall II prospective cohort study. *J Am Med Dir Assoc* 2013; **14**(11): 851 e1-6.
26. Bregigeon S, Galinier A, Zaegel-Faucher O, et al. Frailty in HIV infected people: a new risk factor for bone mineral density loss. *AIDS* 2017; **31**(11): 1573-7.
27. Brothers TD, Kirkland S, Theou O, et al. Predictors of transitions in frailty severity and mortality among people aging with HIV. *PLoS One* 2017; **12**(10): e0185352.
28. Buganza-Torio E, Mitchell N, Abraldes JG, et al. Depression in cirrhosis - a prospective evaluation of the prevalence, predictors and development of a screening nomogram. *Aliment Pharmacol Ther* 2019; **49**(2): 194-201.
29. Buitrago DH, Gangadharan SP, Majid A, et al. Frailty Characteristics Predict Respiratory Failure in Patients Undergoing Tracheobronchoplasty. *Ann Thorac Surg* 2018; **106**(3): 836-41.
30. Chao CT, Wang J, Chien KL, group COoGNiNs. Both pre-frailty and frailty increase healthcare utilization and adverse health outcomes in patients with type 2 diabetes mellitus. *Cardiovasc Diabetol* 2018; **17**(1): 130.
31. Chen SY, Stem M, Cerullo M, et al. The Effect of Frailty Index on Early Outcomes after Combined Colorectal and Liver Resections. *J Gastrointest Surg* 2018; **22**(4): 640-9.

For peer review only

32. Chiang JM, Kaysen GA, Segal M, Chertow GM, Delgado C, Johansen KL. Low testosterone is associated with frailty, muscle wasting and physical dysfunction among men receiving hemodialysis: a longitudinal analysis. *Nephrol Dial Transplant* 2019; **34**(5): 802-10.
33. Chode S, Malmstrom TK, Miller DK, Morley JE. Frailty, Diabetes, and Mortality in Middle-Aged African Americans. *J Nutr Health Aging* 2016; **20**(8): 854-9.
34. Chu NM, Deng A, Ying H, et al. Dynamic Frailty Before Kidney Transplantation: Time of Measurement Matters. *Transplantation* 2019; **103**(8): 1700-4.
35. Chu NM, Gross AL, Shaffer AA, et al. Frailty and Changes in Cognitive Function after Kidney Transplantation. *J Am Soc Nephrol* 2019; **30**(2): 336-45.
36. Chung CJ, Wu C, Jones M, et al. Reduced handgrip strength as a marker of frailty predicts clinical outcomes in patients with heart failure undergoing ventricular assist device placement. *J Card Fail* 2014; **20**(5): 310-5.
37. Cook MJ, Oldroyd A, Pye SR, et al. Frailty and bone health in European men. *Age Ageing* 2017; **46**(4): 635-41.
38. Courtwright AM, Zaleski D, Gardo L, et al. Causes, Preventability, and Cost of Unplanned Rehospitalizations Within 30 Days of Discharge After Lung Transplantation. *Transplantation* 2018; **102**(5): 838-44.
39. Courtwright AM, Zaleski D, Tevald M, et al. Discharge frailty following lung transplantation. *Clin Transplant* 2019; **33**(10): e13694.
40. Cron DC, Friedman JF, Winder GS, et al. Depression and Frailty in Patients With End-Stage Liver Disease Referred for Transplant Evaluation. *Am J Transplant* 2016; **16**(6): 1805-11.
41. de Haas SCM, de Jonge EAL, Voortman T, et al. Dietary patterns and changes in frailty status: the Rotterdam study. *Eur J Nutr* 2018; **57**(7): 2365-75.
42. Delgado C, Doyle JW, Johansen KL. Association of frailty with body composition among patients on hemodialysis. *J Ren Nutr* 2013; **23**(5): 356-62.
43. Delgado C, Grimes BA, Glidden DV, Shlipak M, Sarnak MJ, Johansen KL. Association of Frailty based on self-reported physical function with directly measured kidney function and mortality. *BMC Nephrol* 2015; **16**: 203.
44. Denfeld QE, Winters-Stone K, Mudd JO, Hiatt SO, Chien CV, Lee CS. Frequency of and Significance of Physical Frailty in Patients With Heart Failure. *Am J Cardiol* 2017; **119**(8): 1243-9.
45. Derck JE, Thelen AE, Cron DC, et al. Quality of life in liver transplant candidates: frailty is a better indicator than severity of liver disease. *Transplantation* 2015; **99**(2): 340-4.
46. Dolgin NH, Smith AJ, Harrington SG, Movahedi B, Martins PNA, Bozorgzadeh A. Association Between Sarcopenia and Functional Status in Liver Transplant Patients. *Exp Clin Transplant* 2019; **17**(5): 653-64.
47. Dos Santos Mantovani M, Coelho de Carvalho N, Archangelo TE, et al. Frailty predicts surgical complications after kidney transplantation. A propensity score matched study. *PLoS One* 2020; **15**(2): e0229531.
48. Dumond JB, Francis O, Cottrell M, et al. Tenofovir/emtricitabine metabolites and endogenous nucleotide exposures are associated with p16(INK4a) expression in subjects on combination therapy. *Antivir Ther* 2016; **21**(5): 441-5.
49. Erlandson KM, Allshouse AA, Jankowski CM, et al. Comparison of functional status instruments in HIV-infected adults on effective antiretroviral therapy. *HIV Clin Trials* 2012; **13**(6): 324-34.
50. Erlandson KM, Bradford Y, Samuels DC, et al. Mitochondrial DNA Haplogroups and Frailty in Adults Living with HIV. *AIDS Res Hum Retroviruses* 2020; **36**(3): 214-9.

For peer review only

51. Erlandson KM, Ng DK, Jacobson LP, et al. Inflammation, Immune Activation, Immunosenescence, and Hormonal Biomarkers in the Frailty-Related Phenotype of Men With or at Risk for HIV Infection. *J Infect Dis* 2017; **215**(2): 228-37.
52. Erlandson KM, Perez J, Abdo M, et al. Frailty, Neurocognitive Impairment, or Both in Predicting Poor Health Outcomes Among Adults Living With Human Immunodeficiency Virus. *Clin Infect Dis* 2019; **68**(1): 131-8.
53. Erlandson KM, Wu K, Koletar SL, et al. Association Between Frailty and Components of the Frailty Phenotype With Modifiable Risk Factors and Antiretroviral Therapy. *J Infect Dis* 2017; **215**(6): 933-7.
54. Escota GV, Patel P, Brooks JT, et al. Short communication: The Veterans Aging Cohort Study Index is an effective tool to assess baseline frailty status in a contemporary cohort of HIV-infected persons. *AIDS Res Hum Retroviruses* 2015; **31**(3): 313-7.
55. Fang ZB, Hu FY, Arya S, Gillespie TW, Rajani RR. Preoperative frailty is predictive of complications after major lower extremity amputation. *J Vasc Surg* 2017; **65**(3): 804-11.
56. Fernando SM, McIsaac DI, Rochwerg B, et al. Frailty and invasive mechanical ventilation: association with outcomes, extubation failure, and tracheostomy. *Intensive Care Med* 2019; **45**(12): 1742-52.
57. Fitzpatrick J, Sozio SM, Jaar BG, et al. Frailty, body composition and the risk of mortality in incident hemodialysis patients: the Predictors of Arrhythmic and Cardiovascular Risk in End Stage Renal Disease study. *Nephrol Dial Transplant* 2019; **34**(2): 346-54.
58. Franconi I, Theou O, Wallace L, et al. Construct validation of a Frailty Index, an HIV Index and a Protective Index from a clinical HIV database. *PLoS One* 2018; **13**(10): e0201394.
59. Garonzik-Wang JM, Govindan P, Grinnan JW, et al. Frailty and delayed graft function in kidney transplant recipients. *Arch Surg* 2012; **147**(2): 190-3.
60. George EM, Burke WM, Hou JY, et al. Measurement and validation of frailty as a predictor of outcomes in women undergoing major gynaecological surgery. *BJOG* 2016; **123**(3): 455-61.
61. Gordon SJ, Baker N, Kidd M, Maeder A, Grimmer KA. Pre-frailty factors in community-dwelling 40-75 year olds: opportunities for successful ageing. *BMC Geriatr* 2020; **20**(1): 96.
62. Griffin FR, Mode NA, Ejiogu N, Zonderman AB, Evans MK. Frailty in a racially and socioeconomically diverse sample of middle-aged Americans in Baltimore. *PLoS One* 2018; **13**(4): e0195637.
63. Groß M, Herr A, Hower M, et al. Unemployment, health, and education of HIV-infected males in Germany. *International Journal of Public Health* 2016; **61**(5): 593-602.
64. Guaraldi G, Brothers TD, Zona S, et al. A frailty index predicts survival and incident multimorbidity independent of markers of HIV disease severity. *AIDS* 2015; **29**(13): 1633-41.
65. Guaraldi G, De Francesco D, Milic J, et al. The Interplay Between Age and Frailty in People Living With HIV: Results From an 11-Year Follow-up Observational Study. *Open Forum Infect Dis* 2019; **6**(5): ofz199.
66. Guaraldi G, Dolci G, Zona S, et al. A frailty index predicts post-liver transplant morbidity and mortality in HIV-positive patients. *AIDS Res Ther* 2017; **14**(1): 37.
67. Guaraldi G, Francesco D, Malagoli A, et al. Compression of frailty in adults living with HIV. *BMC Geriatr* 2019; **19**(1): 229.

For peer review only

68. Guaraldi G, Franconi I, Milic J, et al. Thymus Imaging Detection and Size Is Inversely Associated With Metabolic Syndrome and Frailty in People With HIV. *Open Forum Infect Dis* 2019; **6**(10): ofz435.
69. Guaraldi G, Malagoli A, Theou O, et al. Correlates of frailty phenotype and frailty index and their associations with clinical outcomes. *HIV Med* 2017; **18**(10): 764-71.
70. Guaraldi G, Zona S, Silva AR, et al. The dynamic association between Frailty, CD4 and CD4/CD8 ratio in people aging with HIV. *PLoS One* 2019; **14**(2): e0212283.
71. Gustafson DR, Shi Q, Holman S, et al. Predicting death over 8 years in a prospective cohort of HIV-infected women: the Women's Interagency HIV Study. *BMJ Open* 2017; **7**(6): e013993.
72. Gustafson DR, Shi Q, Thurn M, et al. Frailty and Constellations of Factors in Aging HIV-infected and Uninfected Women--The Women's Interagency HIV Study. *J Frailty Aging* 2016; **5**(1): 43-8.
73. Hadenfeldt CJ, Darabaris M, Aufdenkamp M. Frailty Assessment in Patients Utilizing a Free Clinic. *J Health Care Poor Underserved* 2017; **28**(4): 1423-35.
74. Haider S, Grabovac I, Berner C, et al. Frailty in seropositive rheumatoid arthritis patients of working age: a cross-sectional study. *Clin Exp Rheumatol* 2019; **37**(4): 585-92.
75. Hampton JP, Owodunni OP, Bettick D, et al. Compliance to an enhanced recovery pathway among patients with a high frailty index after major gastrointestinal surgery results in improved 30-day outcomes. *Surgery* 2019; **166**(1): 75-81.
76. Harland TA, Wang M, Gunaydin D, et al. Frailty as a Predictor of Neurosurgical Outcomes in Brain Tumor Patients. *World Neurosurg* 2020; **133**: e813-e8.
77. Haugen CE, Chu NM, Ying H, et al. Frailty and Access to Kidney Transplantation. *Clin J Am Soc Nephrol* 2019; **14**(4): 576-82.
78. Haugen CE, McAdams-DeMarco M, Verna EC, et al. Association Between Liver Transplant Wait-list Mortality and Frailty Based on Body Mass Index. *JAMA Surg* 2019; **154**(12): 1103-9.
79. Haugen CE, Mountford A, Warsame F, et al. Incidence, Risk Factors, and Sequelae of Post-kidney Transplant Delirium. *J Am Soc Nephrol* 2018; **29**(6): 1752-9.
80. Haugen CE, Thomas AG, Chu NM, et al. Prevalence of frailty among kidney transplant candidates and recipients in the United States: Estimates from a National Registry and Multicenter Cohort Study. *Am J Transplant* 2020; **20**(4): 1170-80.
81. Hayek S, Gibson TM, Leisenring WM, et al. Prevalence and Predictors of Frailty in Childhood Cancer Survivors and Siblings: A Report From the Childhood Cancer Survivor Study. *J Clin Oncol* 2020; **38**(3): 232-47.
82. Heberton GA, Nassif M, Bierhals A, et al. Usefulness of Psoas Muscle Area Determined by Computed Tomography to Predict Mortality or Prolonged Length of Hospital Stay in Patients Undergoing Left Ventricular Assist Device Implantation. *Am J Cardiol* 2016; **118**(9): 1363-7.
83. Hewitt J, Carter B, McCarthy K, et al. Frailty predicts mortality in all emergency surgical admissions regardless of age. An observational study. *Age Ageing* 2019; **48**(3): 388-94.
84. Hope AA, Hsieh SJ, Petti A, Hurtado-Sbordoni M, Verghese J, Gong MN. Assessing the Usefulness and Validity of Frailty Markers in Critically Ill Adults. *Ann Am Thorac Soc* 2017; **14**(6): 952-9.

For peer review only

85. Huang SM, Tseng LM, Chien LY, et al. Effects of non-sporting and sporting qigong on frailty and quality of life among breast cancer patients receiving chemotherapy. *Eur J Oncol Nurs* 2016; **21**: 257-65.
86. Hyde Z, Flicker L, Smith K, et al. Prevalence and incidence of frailty in Aboriginal Australians, and associations with mortality and disability. *Maturitas* 2016; **87**: 89-94.
87. Ianas V, Berg E, Mohler MJ, Wendel C, Klotz SA. Antiretroviral therapy protects against frailty in HIV-1 infection. *J Int Assoc Provid AIDS Care* 2013; **12**(1): 62-6.
88. Jayanama K, Theou O, Blodgett JM, Cahill L, Rockwood K. Frailty, nutrition-related parameters, and mortality across the adult age spectrum. *BMC Med* 2018; **16**(1): 188.
89. Jha SR, Hannu MK, Chang S, et al. The Prevalence and Prognostic Significance of Frailty in Patients With Advanced Heart Failure Referred for Heart Transplantation. *Transplantation* 2016; **100**(2): 429-36.
90. Jha SR, Hannu MK, Gore K, et al. Cognitive impairment improves the predictive validity of physical frailty for mortality in patients with advanced heart failure referred for heart transplantation. *J Heart Lung Transplant* 2016; **35**(9): 1092-100.
91. Jha SR, Hannu MK, Newton PJ, et al. Reversibility of Frailty After Bridge-to-Transplant Ventricular Assist Device Implantation or Heart Transplantation. *Transplant Direct* 2017; **3**(7): e167.
92. Johansen KL, Dalrymple LS, Delgado C, et al. Factors Associated with Frailty and Its Trajectory among Patients on Hemodialysis. *Clin J Am Soc Nephrol* 2017; **12**(7): 1100-8.
93. Johansen KL, Dalrymple LS, Delgado C, et al. Association between body composition and frailty among prevalent hemodialysis patients: a US Renal Data System special study. *J Am Soc Nephrol* 2014; **25**(2): 381-9.
94. Johansen KL, Dalrymple LS, Delgado C, et al. Comparison of self-report-based and physical performance-based frailty definitions among patients receiving maintenance hemodialysis. *Am J Kidney Dis* 2014; **64**(4): 600-7.
95. Johansen KL, Dalrymple LS, Glidden D, et al. Association of Performance-Based and Self-Reported Function-Based Definitions of Frailty with Mortality among Patients Receiving Hemodialysis. *Clin J Am Soc Nephrol* 2016; **11**(4): 626-32.
96. Johansen KL, Delgado C, Kaysen GA, et al. Frailty Among Patients Receiving Hemodialysis: Evolution of Components and Associations With Mortality. *J Gerontol A Biol Sci Med Sci* 2019; **74**(3): 380-6.
97. Johansen KL, Painter P, Delgado C, Doyle J. Characterization of physical activity and sitting time among patients on hemodialysis using a new physical activity instrument. *J Ren Nutr* 2015; **25**(1): 25-30.
98. Joseph SM, Manghelli JL, Vader JM, et al. Prospective Assessment of Frailty Using the Fried Criteria in Patients Undergoing Left Ventricular Assist Device Therapy. *Am J Cardiol* 2017; **120**(8): 1349-54.
99. Kahlon S, Pederson J, Majumdar SR, et al. Association between frailty and 30-day outcomes after discharge from hospital. *CMAJ* 2015; **187**(11): 799-804.
100. Kallianpur KJ, Sakoda M, Gangcuangco LM, et al. Frailty Characteristics in Chronic HIV Patients are Markers of White Matter Atrophy Independently of Age and Depressive Symptoms: A Pilot Study. *Open Med J* 2016; **3**: 138-52.

For peer review only

101. Kane AE, Gregson E, Theou O, Rockwood K, Howlett SE. The association between frailty, the metabolic syndrome, and mortality over the lifespan. *Geroscience* 2017; **39**(2): 221-9.
102. Kang SH, Do JY, Jeong HY, Lee SY, Kim JC. The Clinical Significance of Physical Activity in Maintenance Dialysis Patients. *Kidney Blood Press Res* 2017; **42**(3): 575-86.
103. Kang SH, Do JY, Lee SY, Kim JC. Effect of dialysis modality on frailty phenotype, disability, and health-related quality of life in maintenance dialysis patients. *PLoS One* 2017; **12**(5): e0176814.
104. Kanters DM, Griffith LE, Hogan DB, Richardson J, Patterson C, Raina P. Assessing the measurement properties of a Frailty Index across the age spectrum in the Canadian Longitudinal Study on Aging. *J Epidemiol Community Health* 2017; **71**(8): 794-9.
105. Kehler DS, Ferguson T, Stammers AN, et al. Prevalence of frailty in Canadians 18-79 years old in the Canadian Health Measures Survey. *BMC Geriatr* 2017; **17**(1): 28.
106. Keller DS, Bankwitz B, Nobel T, Delaney CP. Using frailty to predict who will fail early discharge after laparoscopic colorectal surgery with an established recovery pathway. *Dis Colon Rectum* 2014; **57**(3): 337-42.
107. Kelly SG, Wu K, Tassiopoulos K, Erlandson KM, Koletar SL, Palella FJ. Frailty Is an Independent Risk Factor for Mortality, Cardiovascular Disease, Bone Disease, and Diabetes Among Aging Adults With Human Immunodeficiency Virus. *Clin Infect Dis* 2019; **69**(8): 1370-6.
108. Kelm DJ, Bonnes SL, Jensen MD, et al. Pre-transplant wasting (as measured by muscle index) is a novel prognostic indicator in lung transplantation. *Clin Transplant* 2016; **30**(3): 247-55.
109. Konel JM, Warsame F, Ying H, et al. Depressive symptoms, frailty, and adverse outcomes among kidney transplant recipients. *Clin Transplant* 2018; **32**(10): e13391.
110. Konstantinidis IT, Chouliaras K, Levine EA, Lee B, Votanopoulos KI. Frailty Correlates with Postoperative Mortality and Major Morbidity After Cytoreductive Surgery with Hyperthermic Intraperitoneal Chemotherapy. *Ann Surg Oncol* 2017; **24**(13): 3825-30.
111. Kooij KW, Wit FW, Schouten J, et al. HIV infection is independently associated with frailty in middle-aged HIV type 1-infected individuals compared with similar but uninfected controls. *AIDS* 2016; **30**(2): 241-50.
112. Korada SKC, Zhao D, Tibuakuu M, et al. Frailty and subclinical coronary atherosclerosis: The Multicenter AIDS Cohort Study (MACS). *Atherosclerosis* 2017; **266**: 240-7.
113. Kulkarni SS, Chen H, Josbeno DA, et al. Gait Speed and Grip Strength Are Associated With Dropping Out of the Liver Transplant Waiting List. *Transplant Proc* 2019; **51**(3): 794-7.
114. Kull M, Kallikorm R, Lember M. Impact of a new sarco-osteopenia definition on health-related quality of life in a population-based cohort in Northern Europe. *J Clin Densitom* 2012; **15**(1): 32-8.
115. Kutner NG, Zhang R, Allman RM, Bowling CB. Correlates of ADL difficulty in a large hemodialysis cohort. *Hemodial Int* 2014; **18**(1): 70-7.
116. Kutner NG, Zhang R, Huang Y, McClellan WM, Soltow QA, Lea J. Risk factors for frailty in a large prevalent cohort of hemodialysis patients. *Am J Med Sci* 2014; **348**(4): 277-82.
117. Kutner NG, Zhang R, Huang Y, Wasse H. Falls among hemodialysis patients: potential opportunities for prevention? *Clin Kidney J* 2014; **7**(3): 257-63.

For peer review only

118. Lai JC, Covinsky KE, Dodge JL, et al. Development of a novel frailty index to predict mortality in patients with end-stage liver disease. *Hepatology* 2017; **66**(2): 564-74.
119. Lai JC, Covinsky KE, McCulloch CE, Feng S. The Liver Frailty Index Improves Mortality Prediction of the Subjective Clinician Assessment in Patients With Cirrhosis. *Am J Gastroenterol* 2018; **113**(2): 235-42.
120. Lai JC, Dodge JL, Sen S, Covinsky K, Feng S. Functional decline in patients with cirrhosis awaiting liver transplantation: Results from the functional assessment in liver transplantation (FrAILT) study. *Hepatology* 2016; **63**(2): 574-80.
121. Lai JC, Rahimi RS, Verna EC, et al. Frailty Associated With Waitlist Mortality Independent of Ascites and Hepatic Encephalopathy in a Multicenter Study. *Gastroenterology* 2019; **156**(6): 1675-82.
122. Lauerman MH, Raithel M, Kufera J, et al. Comparison of individual and composite radiographic markers of frailty in trauma. *Injury* 2019; **50**(1): 149-55.
123. Layton AM, Armstrong HF, Baldwin MR, et al. Frailty and maximal exercise capacity in adult lung transplant candidates. *Respir Med* 2017; **131**: 70-6.
124. Lee SY, Yang DH, Hwang E, et al. The Prevalence, Association, and Clinical Outcomes of Frailty in Maintenance Dialysis Patients. *J Ren Nutr* 2017; **27**(2): 106-12.
125. Legge A, Kirkl, S., et al. Prediction of Damage Accrual in Systemic Lupus Erythematosus Using the Systemic Lupus International Collaborating Clinics Frailty Index. *Arthritis & Rheumatology* 2019.
126. Legge A, Kirkland S, Rockwood K, et al. Evaluating the Properties of a Frailty Index and Its Association With Mortality Risk Among Patients With Systemic Lupus Erythematosus. *Arthritis Rheumatol* 2019; **71**(8): 1297-307.
127. Legge A, Kirkland S, Rockwood K, et al. Construction of a Frailty Index as a Novel Health Measure in Systemic Lupus Erythematosus. *J Rheumatol* 2020; **47**(1): 72-81.
128. Leopold-George NTN, Nethathe GD. Frailty in perioperative patients in three South African academic hospitals. *S Afr Med J* 2019; **109**(7): 535-40.
129. Li G, Chen M, Li X, et al. Frailty and risk of osteoporotic fractures in patients with rheumatoid arthritis: Data from the Ontario Best Practices Research Initiative. *Bone* 2019; **127**: 129-34.
130. Li X, Ploner A, Karlsson IK, et al. The frailty index is a predictor of cause-specific mortality independent of familial effects from midlife onwards: a large cohort study. *BMC Med* 2019; **17**(1): 94.
131. Limberg J, Ullmann TM, Gray KD, et al. Laparoscopic Adrenalectomy Has the Same Operative Risk as Routine Laparoscopic Cholecystectomy. *J Surg Res* 2019; **241**: 228-34.
132. Limpawattana P, Wirasorn K, Sookprasert A, et al. Frailty Syndrome in Biliary Tract Cancer Patients: Prevalence and Associated Factors. *Asian Pac J Cancer Prev* 2019; **20**(5): 1497-501.
133. Lisiecki J, Zhang P, Wang L, et al. Morphomic measurement of the temporalis muscle and zygomatic bone as novel predictors of hospital-based clinical outcomes in patients with mandible fracture. *J Craniofac Surg* 2013; **24**(5): 1577-81.
134. Louwers L, Schnickel G, Rubinfeld I. Use of a simplified frailty index to predict Clavien 4 complications and mortality after hepatectomy: analysis of the National Surgical Quality Improvement Project database. *American Journal of Surgery* 2016; **211**(6): 1071-6.

For peer review only

135. Luckett P, Paul RH, Navid J, et al. Deep Learning Analysis of Cerebral Blood Flow to Identify Cognitive Impairment and Frailty in Persons Living With HIV. *J Acquir Immune Defic Syndr* 2019; **82**(5): 496-502.
136. Lurie I, Myers V, Goldbourt U, Gerber Y. Perceived social support following myocardial infarction and long-term development of frailty. *Eur J Prev Cardiol* 2015; **22**(10): 1346-53.
137. Lutski M, Tanne D, Goldbourt U. Tall stature in coronary heart disease patients is associated with decreased risk of frailty in late life. *Geriatr Gerontol Int* 2017; **17**(9): 1270-7.
138. Maffei VJ, Ferguson TF, Brashear MM, et al. Lifetime alcohol use among persons living with HIV is associated with frailty. *AIDS* 2020; **34**(2): 245-54.
139. Makhani SS, Kim FY, Liu Y, et al. Cognitive Impairment and Overall Survival in Frail Surgical Patients. *J Am Coll Surg* 2017; **225**(5): 590-600 e1.
140. Malmstrom TK, Miller DK, Herning MM, Morley JE. Low appendicular skeletal muscle mass (ASM) with limited mobility and poor health outcomes in middle-aged African Americans. *J Cachexia Sarcopenia Muscle* 2013; **4**(3): 179-86.
141. Malmstrom TK, Miller DK, Morley JE. A comparison of four frailty models. *J Am Geriatr Soc* 2014; **62**(4): 721-6.
142. Margolick JB, Bream JH, Martinez-Maza O, et al. Frailty and Circulating Markers of Inflammation in HIV+ and HIV- Men in the Multicenter AIDS Cohort Study. *J Acquir Immune Defic Syndr* 2017; **74**(4): 407-17.
143. Martin L, McKenzie K, Ouellette-Kuntz H. Once frail, always frail? Frailty transitions in home care users with intellectual and developmental disabilities. *Geriatr Gerontol Int* 2018; **18**(4): 547-53.
144. Mascarella MA, Milad D, Richardson K, et al. Preoperative Risk Index Among Patients Undergoing Thyroid or Parathyroid Surgery. *Jama Otolaryngol* 2020; **146**(1): 7-12.
145. McAdams-DeMarco MA, Isaacs K, Darko L, et al. Changes in Frailty After Kidney Transplantation. *J Am Geriatr Soc* 2015; **63**(10): 2152-7.
146. McAdams-DeMarco MA, Law A, King E, et al. Frailty and mortality in kidney transplant recipients. *Am J Transplant* 2015; **15**(1): 149-54.
147. McAdams-DeMarco MA, Law A, Salter ML, et al. Frailty and early hospital readmission after kidney transplantation. *Am J Transplant* 2013; **13**(8): 2091-5.
148. McAdams-DeMarco MA, Law A, Tan J, et al. Frailty, mycophenolate reduction, and graft loss in kidney transplant recipients. *Transplantation* 2015; **99**(4): 805-10.
149. McAdams-DeMarco MA, Tan J, Salter ML, et al. Frailty and Cognitive Function in Incident Hemodialysis Patients. *Clin J Am Soc Nephrol* 2015; **10**(12): 2181-9.
150. McAdams-DeMarco MA, Ying H, Olorundare I, et al. Frailty and Health-Related Quality of Life in End Stage Renal Disease Patients of All Ages. *J Frailty Aging* 2016; **5**(3): 174-9.
151. McAdams-DeMarco MA, Ying H, Olorundare I, et al. Individual Frailty Components and Mortality in Kidney Transplant Recipients. *Transplantation* 2017; **101**(9): 2126-32.
152. McAdams-DeMarco MA, Ying H, Thomas AG, et al. Frailty, Inflammatory Markers, and Waitlist Mortality Among Patients With End-stage Renal Disease in a Prospective Cohort Study. *Transplantation* 2018; **102**(10): 1740-6.
153. McAlister FA, Youngson E, Eurich DT. Treated glycosylated hemoglobin levels in individuals with diabetes mellitus vary little by health status: A retrospective cohort study. *Medicine (Baltimore)* 2016; **95**(24): e3894.

For peer review only

154. McChesney SL, Canter DJ, Monlezun DJ, Green H, Margolin DA. Modified Frailty Index Predicts Postoperative Outcomes in Patients Undergoing Radical Pelvic Surgery. *Am Surg* 2020; **86**(2): 95-103.
155. McDonagh J, Salamonson Y, Ferguson C, et al. Evaluating the convergent and discriminant validity of three versions of the frailty phenotype in heart failure: results from the FRAME-HF study. *Eur J Cardiovasc Nurs* 2020; **19**(1): 55-63.
156. McIntyre M, Gandhi C, Dragonette J, et al. Increasing Frailty Predicts Worse Outcomes and Increased Complications After Angiogram-Negative Subarachnoid Hemorrhages. *World Neurosurg* 2020; **134**: e181-e8.
157. McIntyre MK, Gandhi C, Long A, et al. Age predicts outcomes better than frailty following aneurysmal subarachnoid hemorrhage: A retrospective cohort analysis. *Clin Neurol Neurosurg* 2019; **187**: 105558.
158. McKenzie K, Ouellette-Kuntz H, Martin L. Using an accumulation of deficits approach to measure frailty in a population of home care users with intellectual and developmental disabilities: an analytical descriptive study. *BMC Geriatr* 2015; **15**: 170.
159. McKenzie K, Ouellette-Kuntz H, Martin L. Frailty as a Predictor of Institutionalization Among Adults With Intellectual and Developmental Disabilities. *Intellect Dev Disabil* 2016; **54**(2): 123-35.
160. McNelly AS, Rawal J, Shrikrishna D, et al. An Exploratory Study of Long-Term Outcome Measures in Critical Illness Survivors: Construct Validity of Physical Activity, Frailty, and Health-Related Quality of Life Measures. *Crit Care Med* 2016; **44**(6): e362-9.
161. Mezuk B, Lohman M, Dumenci L, Lapane KL. Are depression and frailty overlapping syndromes in mid- and late-life? A latent variable analysis. *Am J Geriatr Psychiatry* 2013; **21**(6): 560-9.
162. Miller BS, Ignatoski KM, Daignault S, et al. A quantitative tool to assess degree of sarcopenia objectively in patients with hypercortisolism. *Surgery* 2011; **150**(6): 1178-85.
163. Miller EK, Lenke LG, Neuman BJ, et al. External Validation of the Adult Spinal Deformity (ASD) Frailty Index (ASD-FI) in the Scoli-RISK-1 Patient Database. *Spine (Phila Pa 1976)* 2018; **43**(20): 1426-31.
164. Miller EK, Vila-Casademunt A, Neuman BJ, et al. External validation of the adult spinal deformity (ASD) frailty index (ASD-FI). *Eur Spine J* 2018; **27**(9): 2331-8.
165. Moayedi Y, Duero Posada JG, Foroutan F, et al. The prognostic significance of frailty compared to peak oxygen consumption and B-type natriuretic peptide in patients with advanced heart failure. *Clin Transplant* 2018; **32**(1): 01.
166. Montgomery CL, Zuege DJ, Rolfson DB, et al. Implementation of population-level screening for frailty among patients admitted to adult intensive care in Alberta, Canada. *Can J Anaesth* 2019; **66**(11): 1310-9.
167. Montgomery E, Macdonald PS, Newton PJ, et al. Frailty as a Predictor of Mortality in Patients With Interstitial Lung Disease Referred for Lung Transplantation. *Transplantation* 2020; **104**(4): 864-72.
168. Morgello S, Gensler G, Sherman S, et al. Frailty in medically complex individuals with chronic HIV. *AIDS* 2019; **33**(10): 1603-11.
169. Morley JE, Malmstrom TK, Miller DK. A simple frailty questionnaire (FRAIL) predicts outcomes in middle aged African Americans. *J Nutr Health Aging* 2012; **16**(7): 601-8.
170. Mosquera C, Bermudez JM, Evans JL, Spaniolas K, MacGillivary DC, Fitzgerald TL. Frailty Predicts Failure to Rescue after Thoracoabdominal Operation. *J Am Coll Surg* 2018; **226**(6): 978-86.

For peer review only

171. Myers V, Broday DM, Steinberg DM, Yuval, Drory Y, Gerber Y. Exposure to particulate air pollution and long-term incidence of frailty after myocardial infarction. *Ann Epidemiol* 2013; **23**(7): 395-400.
172. Myers V, Drory Y, Gerber Y, Israel Study Group on First Acute Myocardial I. Clinical relevance of frailty trajectory post myocardial infarction. *Eur J Prev Cardiol* 2014; **21**(6): 758-66.
173. Myers V, Drory Y, Goldbourt U, Gerber Y. Multilevel socioeconomic status and incidence of frailty post myocardial infarction. *Int J Cardiol* 2014; **170**(3): 338-43.
174. Nampoothiri RV, Kasudhan KS, Patil AN, et al. Impact of frailty, melphalan pharmacokinetics, and pharmacogenetics on outcomes post autologous hematopoietic cell transplantation for multiple myeloma. *Bone Marrow Transplant* 2019; **54**(12): 2088-95.
175. Nastasi AJ, McAdams-DeMarco MA, Schrack J, et al. Pre-Kidney Transplant Lower Extremity Impairment and Post-Kidney Transplant Mortality. *Am J Transplant* 2018; **18**(1): 189-96.
176. Ness KK, Krull KR, Jones KE, et al. Physiologic frailty as a sign of accelerated aging among adult survivors of childhood cancer: a report from the St Jude Lifetime cohort study. *J Clin Oncol* 2013; **31**(36): 4496-503.
177. Ney M, Tangri N, Dobbs B, et al. Predicting Hepatic Encephalopathy-Related Hospitalizations Using a Composite Assessment of Cognitive Impairment and Frailty in 355 Patients With Cirrhosis. *Am J Gastroenterol* 2018; **113**(10): 1506-15.
178. Onen NF, Patel P, Baker J, et al. Frailty and Pre-Frailty in a Contemporary Cohort of HIV-Infected Adults. *J Frailty Aging* 2014; **3**(3): 158-65.
179. Onen NF, Shacham E, Stamm KE, Turner Overton E. Sexual behaviors and results of bacterial sexually transmitted infections testing among frail HIV-infected individuals. *J Int Assoc Physicians AIDS Care (Chic)* 2010; **9**(1): 30-3.
180. Oppenheim H, Paolillo EW, Moore RC, et al. Neurocognitive functioning predicts frailty index in HIV. *Neurology* 2018; **91**(2): e162-e70.
181. Oskutis MQ, Lauerman MH, Kufera JA, et al. Are frailty markers associated with serious thoracic and spinal injuries among motor vehicle crash occupants? *J Trauma Acute Care Surg* 2016; **81**(1): 156-61.
182. Painter P, Kuskowski M. A closer look at frailty in ESRD: getting the measure right. *Hemodial Int* 2013; **17**(1): 41-9.
183. Palmer KT, D'Angelo S, Harris EC, et al. Frailty, prefrailty and employment outcomes in Health and Employment After Fifty (HEAF) Study. *Occup Environ Med* 2017; **74**(7): 476-82.
184. Pamukcuoglu M, Bhatia S, Weisdorf DJ, et al. Hematopoietic Cell Transplant-Related Toxicities and Mortality in Frail Recipients. *Biol Blood Marrow Transplant* 2019; **25**(12): 2454-60.
185. Paolillo EW, Saloner R, Montoya JL, et al. Frailty in Comorbid HIV and Lifetime Methamphetamine Use Disorder: Associations with Neurocognitive and Everyday Functioning. *AIDS Res Hum Retroviruses* 2019; **35**(11-12): 1044-53.
186. Passias PG, Bortz CA, Pierce KE, et al. Decreased rates of 30-day perioperative complications following ASD-corrective surgery: A modified Clavien analysis of 3300 patients from 2010 to 2014. *J Clin Neurosci* 2019; **61**: 147-52.
187. Pathai S, Gilbert C, Weiss HA, et al. Frailty in HIV-infected adults in South Africa. *J Acquir Immune Defic Syndr* 2013; **62**(1): 43-51.
188. Pathai S, Lawn SD, Weiss HA, Cook C, Bekker LG, Gilbert CE. Retinal nerve fibre layer thickness and contrast sensitivity in HIV-infected individuals in South Africa: a case-control study. *PLoS One* 2013; **8**(9): e73694.

For peer review only

189. Pathai S, Shiels PG, Weiss HA, et al. Ocular parameters of biological ageing in HIV-infected individuals in South Africa: relationship with chronological age and systemic biomarkers of ageing. *Mech Ageing Dev* 2013; **134**(9): 400-6.
190. Pena FG, Theou O, Wallace L, et al. Comparison of alternate scoring of variables on the performance of the frailty index. *BMC Geriatr* 2014; **14**: 25.
191. Perez Fernandez M, Martinez Miguel P, Ying H, et al. Comorbidity, Frailty, and Waitlist Mortality among Kidney Transplant Candidates of All Ages. *Am J Nephrol* 2019; **49**(2): 103-10.
192. Perez-Zepeda MU, Avila-Funes JA, Gutierrez-Robledo LM, Garcia-Pena C. Frailty Across Age Groups. *J Frailty Aging* 2016; **5**(1): 15-9.
193. Pierce KE, Passias PG, Alas H, et al. Does Patient Frailty Status Influence Recovery Following Spinal Fusion for Adult Spinal Deformity?: An Analysis of Patients With 3-Year Follow-up. *Spine (Phila Pa 1976)* 2020; **45**(7): E397-E405.
194. Piggott DA, Muzaale AD, Varadhan R, et al. Frailty and Cause-Specific Hospitalization Among Persons Aging With HIV Infection and Injection Drug Use. *J Gerontol A Biol Sci Med Sci* 2017; **72**(3): 389-94.
195. Piggott DA, Varadhan R, Mehta SH, et al. Frailty, Inflammation, and Mortality Among Persons Aging With HIV Infection and Injection Drug Use. *J Gerontol A Biol Sci Med Sci* 2015; **70**(12): 1542-7.
196. Prince CS, Noren Hooten N, Mode NA, et al. Frailty in middle age is associated with frailty status and race-specific changes to the transcriptome. *Aging (Albany NY)* 2019; **11**(15): 5518-34.
197. Ramaty E, Maor E, Peltz-Sinvani N, et al. Low ALT blood levels predict long-term all-cause mortality among adults. A historical prospective cohort study. *European Journal of Internal Medicine* 2014; **25**(10): 919-21.
198. Ravindrarajah R, Lee DM, Pye SR, et al. The ability of three different models of frailty to predict all-cause mortality: results from the European Male Aging Study (EMAS). *Arch Gerontol Geriatr* 2013; **57**(3): 360-8.
199. Rees HC, Meister E, Mohler MJ, Klotz SA. HIV-Related Frailty Is Not Characterized by Sarcopenia. *Journal of the International Association of Providers of AIDS Care* 2016; **15**(2): 131-4.
200. Reid DBC, Daniels AH, Ailon T, et al. Frailty and Health-Related Quality of Life Improvement Following Adult Spinal Deformity Surgery. *World Neurosurgery* 2018; **112**: e548-e54.
201. Ribeiro SML, Morley JE, Malmstrom TK, Miller DK. Fruit and vegetable intake and physical activity as predictors of disability risk factors in African-American middle-aged individuals. *J Nutr Health Aging* 2016; **20**(9): 891-6.
202. Richardson AM, McCarthy DJ, hu J, et al. Predictors of Successful Discharge of Patients on Postoperative Day 1 After Craniotomy for Brain Tumor. *World Neurosurgery* 2019; **126**: e869-e77.
203. Rietman ML, Spijkerman AMW, Wong A, et al. Antioxidants linked with physical, cognitive and psychological frailty: Analysis of candidate biomarkers and markers derived from the MARK-AGE study. *Mech Ageing Dev* 2019; **177**: 135-43.
204. Rochira V, Diazzi C, Santi D, et al. Low testosterone is associated with poor health status in men with human immunodeficiency virus infection: a retrospective study. *Andrology* 2015; **3**(2): 298-308.
205. Rockwood K, Song X, Mitnitski A. Changes in relative fitness and frailty across the adult lifespan: evidence from the Canadian National Population Health Survey. *CMAJ Canadian Medical Association Journal* 2011; **183**(8): E487-94.

For peer review only

206. Rockwood MR, MacDonald E, Sutton E, Rockwood K, Baron M, Canadian Scleroderma Research G. Frailty index to measure health status in people with systemic sclerosis. *Journal of Rheumatology* 2014; **41**(4): 698-705.
207. Roshanravan B, Khatri M, Robinson-Cohen C, et al. A prospective study of frailty in nephrology-referred patients with CKD. *American Journal of Kidney Diseases* 2012; **60**(6): 912-21.
208. Rothenberg KA, Stern JR, George EL, et al. Association of Frailty and Postoperative Complications With Unplanned Readmissions After Elective Outpatient Surgery. *JAMA Network Open* 2019; **2**(5): e194330.
209. Rozenberg D, Mathur S, Wickerson L, Chowdhury NA, Singer LG. Frailty and clinical benefits with lung transplantation. *J Heart Lung Transplant* 2018; **37**(10): 1245-53.
210. Rubtsova AA, Marquine MJ, Depp C, et al. Psychosocial Correlates of Frailty Among HIV-Infected and HIV-Uninfected Adults. *Behavioral Medicine* 2018; **45**(3): 210-20.
211. Sabel MS, Lee J, Cai S, Englesbe MJ, Holcombe S, Wang S. Sarcopenia as a prognostic factor among patients with stage III melanoma. *Annals of Surgical Oncology* 2011; **18**(13): 3579-85.
212. Sadiq F, Kronzer VL, Wildes TS, et al. Frailty Phenotypes and Relations With Surgical Outcomes: A Latent Class Analysis. *Anesth Analg* 2018; **127**(4): 1017-27.
213. Sahli Z, Canner JK, Najjar O, et al. Association Between Age and Patient-Reported Changes in Voice and Swallowing After Thyroidectomy. *Laryngoscope* 2019; **129**(2): 519-24.
214. Salem BE. Characterizing Frailty among Homeless Adults: University of California, Los Angeles; 2013.
215. Salem BE, Nyamathi A, Brecht ML, et al. Constructing and identifying predictors of frailty among homeless adults-a latent variable structural equations model approach. *Arch Gerontol Geriatr* 2014; **58**(2): 248-56.
216. Salem BE, Nyamathi AM, Brecht ML, et al. Correlates of frailty among homeless adults. *West J Nurs Res* 2013; **35**(9): 1128-52.
217. Sandkovsky U, Robertson KR, Meza JL, et al. Pilot study of younger and older HIV-infected adults using traditional and novel functional assessments. *HIV Clin Trials* 2013; **14**(4): 165-74.
218. Schaenman J, Castellon L, Liang EC, et al. The Frailty Risk Score predicts length of stay and need for rehospitalization after kidney transplantation in a retrospective cohort: a pilot study. *Pilot Feasibility Stud* 2019; **5**: 144.
219. Schinkel-Ivy A, Mosca I, Mansfield A. Factors Contributing to Unexpected Retirement and Unemployment in Adults Over 50 Years Old in Ireland. *Gerontol Geriatr Med* 2017; **3**: 2333721417722709.
220. Schopmeyer L, El Moumni M, Nieuwenhuijs-Moeke GJ, Berger SP, Bakker SJL, Pol RA. Frailty has a significant influence on postoperative complications after kidney transplantation-a prospective study on short-term outcomes. *Transpl Int* 2019; **32**(1): 66-74.
221. Sharma A, Hoover DR, Shi Q, et al. Frailty as a predictor of falls in HIV-infected and uninfected women. *Antivir Ther* 2019; **24**(1): 51-61.
222. Sharma A, Shi Q, Hoover DR, et al. Frailty predicts fractures among women with and at-risk for HIV. *AIDS* 2019; **33**(3): 455-63.
223. Shin JI, Kothari P, Phan K, et al. Frailty Index as a Predictor of Adverse Postoperative Outcomes in Patients Undergoing Cervical Spinal Fusion. *Spine (Phila Pa 1976)* 2017; **42**(5): 304-10.

For peer review only

224. Singer JP, Diamond JM, Anderson MR, et al. Frailty phenotypes and mortality after lung transplantation: A prospective cohort study. *Am J Transplant* 2018; **18**(8): 1995-2004.
225. Singer JP, Diamond JM, Gries CJ, et al. Frailty Phenotypes, Disability, and Outcomes in Adult Candidates for Lung Transplantation. *Am J Respir Crit Care Med* 2015; **192**(11): 1325-34.
226. Smit E, Wanke C, Dong K, et al. Frailty, Food Insecurity, and Nutritional Status in People Living with Hiv. *J Frailty Aging* 2015; **4**(4): 191-7.
227. Smitherman AB, Anderson C, Lund JL, Bensen JT, Rosenstein DL, Nichols HB. Frailty and Comorbidities Among Survivors of Adolescent and Young Adult Cancer: A Cross-Sectional Examination of a Hospital-Based Survivorship Cohort. *J Adolesc Young Adult Oncol* 2018; **7**(3): 374-83.
228. Soma O, Hatakeyama S, Okamoto T, et al. Clinical implication of a quantitative frailty assessment tool for prognosis in patients with urological cancers. *Oncotarget* 2018; **9**(25): 17396-405.
229. Stanjek-Cichoracka A, Wozniak-Grygiel E, Laszewska A, Zembala M, Ochman M. Assessment of Cytokines, Biochemical Markers of Malnutrition and Frailty Syndrome Patients Considered for Lung Transplantation. *Transplant Proc* 2019; **51**(6): 2009-13.
230. Stenholm S, Strandberg TE, Pitkala K, Sainio P, Heliovaara M, Koskinen S. Midlife obesity and risk of frailty in old age during a 22-year follow-up in men and women: the Mini-Finland Follow-up Survey. *J Gerontol A Biol Sci Med Sci* 2014; **69**(1): 73-8.
231. Subramaniam S, Aalberg JJ, Soriano RP, Divino CM. New 5-Factor Modified Frailty Index Using American College of Surgeons NSQIP Data. *J Am Coll Surg* 2018; **226**(2): 173-81 e8.
232. Susanto M, Hubbard RE, Gardiner PA. Association of 12-Year Trajectories of Sitting Time With Frailty in Middle-Aged Women. *Am J Epidemiol* 2018; **187**(11): 2387-96.
233. Susanto M, Hubbard RE, Gardiner PA. Validity and Responsiveness of the FRAIL Scale in Middle-Aged Women. *J Am Med Dir Assoc* 2018; **19**(1): 65-9.
234. Swiecicka A, Lunt M, Ahern T, et al. Nonandrogenic Anabolic Hormones Predict Risk of Frailty: European Male Ageing Study Prospective Data. *J Clin Endocrinol Metab* 2017; **102**(8): 2798-806.
235. Sy J, McCulloch CE, Johansen KL. Depressive symptoms, frailty, and mortality among dialysis patients. *Hemodial Int* 2019; **23**(2): 239-46.
236. Sy J, Streja E, Grimes B, Johansen KL. The Marginal Cost of Frailty Among Medicare Patients on Hemodialysis. *Kidney Int Rep* 2020; **5**(3): 289-95.
237. Tajar A, O'Connell MD, Mitnitski AB, et al. Frailty in relation to variations in hormone levels of the hypothalamic-pituitary-testicular axis in older men: results from the European male aging study. *J Am Geriatr Soc* 2011; **59**(5): 814-21.
238. Tapper EB, Derstine B, Baki J, Su GL. Bedside Measures of Frailty and Cognitive Function Correlate with Sarcopenia in Patients with Cirrhosis. *Dig Dis Sci* 2019; **64**(12): 3652-9.
239. Tapper EB, Konerman M, Murphy S, Sonnenday CJ. Hepatic encephalopathy impacts the predictive value of the Fried Frailty Index. *Am J Transplant* 2018; **18**(10): 2566-70.
240. Telemi E, Trofymenko O, Venkat R, Pandit V, Pandian TK, Nfonsam VN. Frailty Predicts Morbidity after Colectomy for Ulcerative Colitis. *Am Surg* 2018; **84**(2): 225-9.
241. Traven SA, Horn RW, Reeves RA, Walton ZJ, Woolf SK, Slone HS. The 5-Factor Modified Frailty Index Predicts Complications, Hospital Admission, and Mortality Following Arthroscopic Rotator Cuff Repair. *Arthroscopy* 2020; **36**(2): 383-8.

For peer review only

242. Trung TN, Duoc NVT, Nhat LTH, et al. Functional outcome and muscle wasting in adults with tetanus. *Trans R Soc Trop Med Hyg* 2019; **113**(11): 706-13.
243. Tuttle LJ, Bittel DC, Bittel AJ, Sinacore DR. Early-Onset Physical Frailty in Adults With Diabesity and Peripheral Neuropathy. *Can J Diabetes* 2018; **42**(5): 478-83.
244. Van Pilsum Rasmussen S, Konel J, Warsame F, et al. Engaging clinicians and patients to assess and improve frailty measurement in adults with end stage renal disease. *BMC Nephrol* 2018; **19**(1): 8.
245. Vatanen A, Hou M, Huang T, et al. Clinical and biological markers of premature aging after autologous SCT in childhood cancer. *Bone Marrow Transplant* 2017; **52**(4): 600-5.
246. Venado A, McCulloch C, Greenland JR, et al. Frailty trajectories in adult lung transplantation: A cohort study. *J Heart Lung Transplant* 2019; **38**(7): 699-707.
247. Verheij E, Kirk GD, Wit FW. Frailty is associated with mortality and incident comorbidity among middle-aged HIV-positive and HIV-negative participants. *Care Management* 2020; **26**(1): 22-.
248. Verschoor CP, Tamim H. Frailty Is Inversely Related to Age at Menopause and Elevated in Women Who Have Had a Hysterectomy: An Analysis of the Canadian Longitudinal Study on Aging. *J Gerontol A Biol Sci Med Sci* 2019; **74**(5): 675-82.
249. Wade KF, Lee DM, McBeth J, et al. Chronic widespread pain is associated with worsening frailty in European men. *Age Ageing* 2016; **45**(2): 268-74.
250. Wallace LM, Theou O, Kirkland SA, et al. Accumulation of non-traditional risk factors for coronary heart disease is associated with incident coronary heart disease hospitalization and death. *PLoS One* 2014; **9**(3): e90475.
251. Wang CW, Lebsack A, Chau S, Lai JC. The Range and Reproducibility of the Liver Frailty Index. *Liver Transpl* 2019; **25**(6): 841-7.
252. Wang R, Shlipak MG, Ix JH, et al. Association of Fibroblast Growth Factor-23 (FGF-23) With Incident Frailty in HIV-Infected and HIV-Uninfected Individuals. *J Acquir Immune Defic Syndr* 2019; **80**(1): 118-25.
253. Warsame F, Ying H, Haugen CE, et al. Intradialytic Activities and Health-Related Quality of Life Among Hemodialysis Patients. *Am J Nephrol* 2018; **48**(3): 181-9.
254. Wen Y, Jabir MA, Dosokey EM, et al. Using Modified Frailty Index to Predict Safe Discharge Within 48 Hours of Ileostomy Closure. *Dis Colon Rectum* 2017; **60**(1): 76-80.
255. Wilkes JG, Evans JL, Prato BS, Hess SA, MacGillivray DC, Fitzgerald TL. Frailty Cost: Economic Impact of Frailty in the Elective Surgical Patient. *J Am Coll Surg* 2019; **228**(6): 861-70.
256. Williams DM, Jylhava J, Pedersen NL, Hagg S. A Frailty Index for UK Biobank Participants. *J Gerontol A Biol Sci Med Sci* 2019; **74**(4): 582-7.
257. Wilson CL, Chemaitilly W, Jones KE, et al. Modifiable Factors Associated With Aging Phenotypes Among Adult Survivors of Childhood Acute Lymphoblastic Leukemia. *J Clin Oncol* 2016; **34**(21): 2509-15.
258. Wilson JM, Holzgrefe RE, Staley CA, Schenker ML, Meals C. The Effect of Malnutrition on Postoperative Complications Following Surgery for Distal Radius Fractures. *J Hand Surg Am* 2019; **44**(9): 742-50.

For peer review only

259. Wilson JM, Lunati MP, Grabel ZJ, Staley CA, Schwartz AM, Schenker ML. Hypoalbuminemia Is an Independent Risk Factor for 30-Day Mortality, Postoperative Complications, Readmission, and Reoperation in the Operative Lower Extremity Orthopaedic Trauma Patient. *J Orthop Trauma* 2019; **33**(6): 284-91.
260. Wilson ME, Vakil AP, Kandel P, Undavalli C, Dunlay SM, Kennedy CC. Pretransplant frailty is associated with decreased survival after lung transplantation. *J Heart Lung Transplant* 2016; **35**(2): 173-8.
261. Womack JA, Goulet JL, Gibert C, et al. Physiologic frailty and fragility fracture in HIV-infected male veterans. *Clin Infect Dis* 2013; **56**(10): 1498-504.
262. Wulunggono W, Yunihastuti E, Shatri H, Wahyudi ER, Ophinni Y. Frailty among HIV-1 Infected Adults under Antiretroviral Therapy in Indonesia. *Curr HIV Res* 2019; **17**(3): 204-13.
263. Xu W, Zhang XM, Ke T, Cai HR, Gao X. Modified Frailty Index and Body Mass Index as Predictors of Adverse Surgical Outcomes in Degenerative Spinal Disease. *Turk Neurosurg* 2018; **28**(6): 897-903.
264. Yadla M, John JP, Mummadi M. A study of clinical assessment of frailty in patients on maintenance hemodialysis supported by cashless government scheme. *Saudi J Kidney Dis Transpl* 2017; **28**(1): 15-22.
265. Yagi M, Michikawa T, Hosogane N, et al. Treatment for Frailty Does Not Improve Complication Rates in Corrective Surgery for Adult Spinal Deformity. *Spine (Phila Pa 1976)* 2019; **44**(10): 723-31.
266. Yagi M, Michikawa T, Hosogane N, et al. The 5-Item Modified Frailty Index Is Predictive of Severe Adverse Events in Patients Undergoing Surgery for Adult Spinal Deformity. *Spine (Phila Pa 1976)* 2019; **44**(18): E1083-E91.
267. Yi C, Lin J, Cao P, et al. Prevalence and Prognosis of Coexisting Frailty and Cognitive Impairment in Patients on Continuous Ambulatory Peritoneal Dialysis. *Sci Rep* 2018; **8**(1): 17305.
268. Young AC, Glaser K, Spector TD, Steves CJ. The Identification of Hereditary and Environmental Determinants of Frailty in a Cohort of UK Twins. *Twin Res Hum Genet* 2016; **19**(6): 600-9.

For peer review only

PRISMA 2009 Checklist

Section/topic	#	Checklist item	Reported on page #
**TITLE**			
Title	1	Identify the report as a systematic review, meta-analysis, or both.	1
**ABSTRACT**			
Structured summary	2	Provide a structured summary including, as applicable: background; objectives; data sources; study eligibility criteria, participants, and interventions; study appraisal and synthesis methods; results; limitations; conclusions and implications of key findings; systematic review registration number.	2
**INTRODUCTION**			
Rationale	3	Describe the rationale for the review in the context of what is already known.	3-4
Objectives	4	Provide an explicit statement of questions being addressed with reference to participants, interventions, comparisons, outcomes, and study design (PICOS).	4
**METHODS**			
Protocol and registration	5	Indicate if a review protocol exists, if and where it can be accessed (e.g., Web address), and, if available, provide registration information including registration number.	NA
Eligibility criteria	6	Specify study characteristics (e.g., PICOS, length of follow-up) and report characteristics (e.g., years considered, language, publication status) used as criteria for eligibility, giving rationale.	5-6
Information sources	7	Describe all information sources (e.g., databases with dates of coverage, contact with study authors to identify additional studies) in the search and date last searched.	5
Search	8	Present full electronic search strategy for at least one database, including any limits used, such that it could be repeated.	Supplementary materials
Study selection	9	State the process for selecting studies (i.e., screening, eligibility, included in systematic review, and, if applicable, included in the meta-analysis).	6
Data collection process	10	Describe method of data extraction from reports (e.g., piloted forms, independently, in duplicate) and any processes for obtaining and confirming data from investigators.	6-7
Data items	11	List and define all variables for which data were sought (e.g., PICOS, funding sources) and any assumptions and simplifications made.	7
Risk of bias in individual studies	12	Describe methods used for assessing risk of bias of individual studies (including specification of whether this was done at the study or outcome level), and how this information is to be used in any data synthesis.	NA
Summary measures	13	State the principal summary measures (e.g., risk ratio, difference in means).	NA
Synthesis of results	14	Describe the methods of handling data and combining results of studies, if done, including measures of consistency (e.g., I^2) for each meta-analysis.	NA

For peer review only

For peer review only - http://bmjopen.bmj.com/site/about/guidelines.xhtml

PRISMA 2009 Checklist

Section/topic	#	Checklist item	Reported on page #
Risk of bias across studies	15	Specify any assessment of risk of bias that may affect the cumulative evidence (e.g., publication bias, selective reporting within studies).	NA
Additional analyses	16	Describe methods of additional analyses (e.g., sensitivity or subgroup analyses, meta-regression), if done, indicating which were pre-specified.	NA
**RESULTS**			
Study selection	17	Give numbers of studies screened, assessed for eligibility, and included in the review, with reasons for exclusions at each stage, ideally with a flow diagram.	8, and figure 1
Study characteristics	18	For each study, present characteristics for which data were extracted (e.g., study size, PICOS, follow-up period) and provide the citations.	Supplementary materials table S2
Risk of bias within studies	19	Present data on risk of bias of each study and, if available, any outcome level assessment (see item 12).	NA
Results of individual studies	20	For all outcomes considered (benefits or harms), present, for each study: (a) simple summary data for each intervention group (b) effect estimates and confidence intervals, ideally with a forest plot.	NA
Synthesis of results	21	Present results of each meta-analysis done, including confidence intervals and measures of consistency.	9-11, supplementary materials tables S3, S4, and figures S1 and S2
Risk of bias across studies	22	Present results of any assessment of risk of bias across studies (see Item 15).	NA
Additional analysis	23	Give results of additional analyses, if done (e.g., sensitivity or subgroup analyses, meta-regression [see Item 16]).	NA
**DISCUSSION**			
Summary of evidence	24	Summarize the main findings including the strength of evidence for each main outcome; consider their relevance to key groups (e.g., healthcare providers, users, and policy makers).	11-12
Limitations	25	Discuss limitations at study and outcome level (e.g., risk of bias), and at review-level (e.g., incomplete retrieval of identified research, reporting bias).	12-13
Conclusions	26	Provide a general interpretation of the results in the context of other evidence, and implications for future research.	11-12
**FUNDING**			

For peer review only

PRISMA 2009 Checklist

Funding	27	Describe sources of funding for the systematic review and other support (e.g., supply of data); role of funders for the systematic review.	Title page

From: Moher D, Liberati A, Tetzlaff J, Altman DG, The PRISMA Group (2009). Preferred Reporting Items for Systematic Reviews and Meta-Analyses: The PRISMA Statement. PLoS Med 6(7): e1000097. doi:10.1371/journal.pmed1000097

For more information, visit: **www.prisma-statement.org**.

Page 2 of 2

For peer review only
